# Photoredox-Catalyzed Lysine C(sp^3^)–H Functionalization for Peptide Editing

**DOI:** 10.1021/jacs.6c02967

**Published:** 2026-06-24

**Authors:** Christopher W. Lamartina, Paramjit S. Arora

**Affiliations:** Department of Chemistry, 5894 New York University, New York, New York 10003, United States

## Abstract

The selective chemical modification of lysine residues beyond its ε-amino group remains a long-standing challenge in peptide synthesis due to the conformational flexibility of the residue, the nucleophilicity of the amine group, and the inert C­(sp^3^)–H bonds of the aliphatic side chain. Herein we report a photoredox-catalyzed reaction for site-selective C­(sp^3^)–H functionalization of lysine and other primary amine residues through α-amino radical intermediates generated from trifluoroacetamide protecting groups. This transformation proceeds under mild conditions and exhibits broad tolerance to residue identity and alkene coupling partners. The technology is compatible with standard Fmoc-solid phase peptide synthesis and direct on-resin modification. The method facilitates both intermolecular alkylation and intramolecular macrocyclization, providing access to a diverse array of unnatural amino acid motifs and peptide architectures. Collectively, this work establishes redox-active amide protecting groups as versatile handles for radical generation in complex molecular settings and expands the toolbox for late-stage peptide diversification.

## Graphical abstract

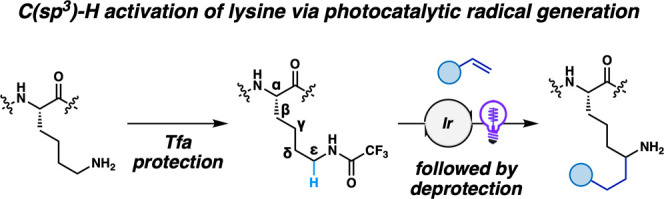

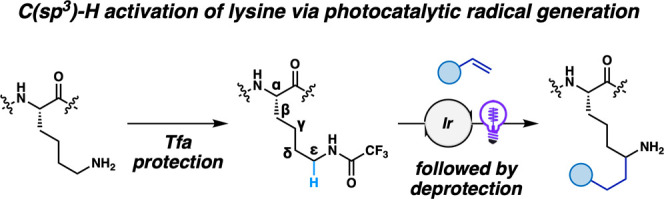

## Introduction

The design of peptide therapeutics is evolving to incorporate more sophisticated structural modifications to enhance their target engagement, metabolic stability, membrane permeability, and absorption capabilities.^1–4^ These goals require new chemistries for peptide backbone and side chain editing. There has been a growing interest in both the synthetic and biological communities in expanding the functionality available in peptides and proteins beyond the 20 canonical amino acids by incorporating unnatural amino acids (UAAs).^5–10^ One particularly effective strategy for UAA incorporation involves the divergent modification of native amino acid residues rather than de novo synthesis of fully unnatural building blocks.^11^ This approach enables streamlined technological advances wherein a common mechanistic logic can be leveraged to generate a wide array of distinct derivatives. However, the success of such strategies depends critically on the ability to selectively engage otherwise inert functional handles within complex peptide environments.

Among the canonical amino acids, lysine presents an attractive target for selective chemical modification.^12^ The ε-amino group of lysine is routinely employed as a reactive handle for derivatization due to its innate nucleophilicity.^13–15^ Lysine is commonly modified by amidation,^16^ alkylation via reductive amination,^17^ arylation via palladium complexes,^18^ and SuFEx chemistry.^19^ Additionally, the amino group can be activated and deaminated for cross coupling reactions (Scheme 1A).^20^

**Scheme 1:**
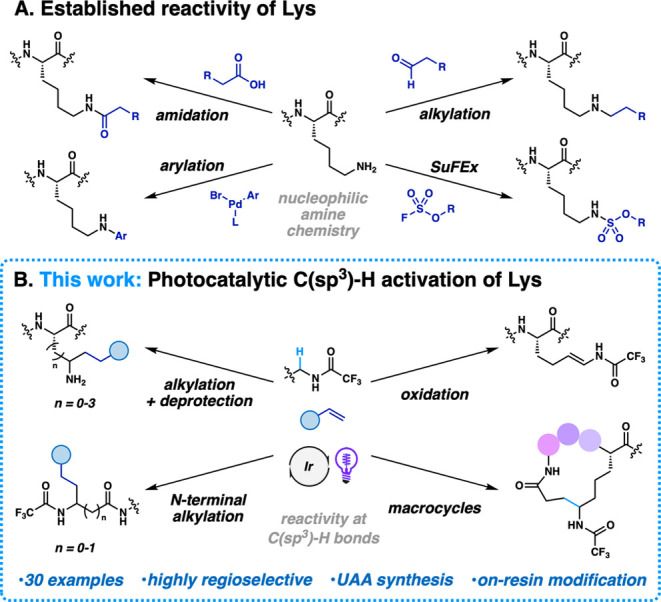
Modes of Reactivity for Lysine

However, the aliphatic C­(sp^3^)–H bonds of lysine have remained largely inaccessible for selective functionalization. For example, selective modifications at the β, γ, δ, and ε positions of lysine would afford difficult-to-generate branched aliphatic side chains to graft peptidomimetic ligands.^21^ Given the accessibility of shorter lysine analogs such as ornithine (Orn), 2,4-diaminobutyric acid (Dab), and 2,3-diaminopropionic acid (Dap), a range of branched derivatives may be accessed. The concept of C–C bond formation at the side chain is distinct from previous ε-amino modification methods due to the ability to keep the primary amino functionality of the residue, which can be further modified or retained to offer a cationic group under physiological conditions. C­(sp^3^)–H modifications on lysine are mechanistically challenging because the nitrogen heteroatom readily undergoes nucleophilic reactions, diverting reactivity away from adjacent aliphatic C–H bonds. Moreover, these C–H bonds possess high activation energies, minimal electronic bias, and significant conformational freedom, making chemoselective modifications a difficult undertaking.

Here we show that C­(sp^3^)–H modification of the lysine side chain can be facilitated by visible light-activated photocatalysis (Scheme 1B). Photocatalytic methods allow generation of reactive radical intermediates under mild conditions. Upon photoexcitation, metal-centered or organic photocatalysts access long-lived excited states capable of engaging a wide range of substrates through single-electron transfer processes.^22^ Photoredox catalysis enables the generation of diverse radical species in a site-selective manner with broad functional group tolerance.^23^ These attributes render photoredox catalysis particularly well suited for methodology development in complex biomolecular settings.

The intersection of photoredox catalysis and peptide modification has emerged as a distinct and rapidly developing area of research (Scheme 2A). Seminal contributions include a photochemically initiated C­(sp^2^)–H activation of tyrosine,^24^ a C­(sp^3^)–H activation reaction at the benzylic position of tryptophan,^25^ an iridium-catalyzed C–S bond cleavage of methionine,^26^ and a complementary lumiflavin-catalyzed C­(sp^3^)–H activation of the methyl group of methionine.^27^ Elegant examples that include a photochemical deoxygenative C–O cleavage protocol for serine alkylation^28^ and a decarboxylative radical approach to C-terminal peptide modification have also garnered significant attention.^29,30^

**Scheme 2:**
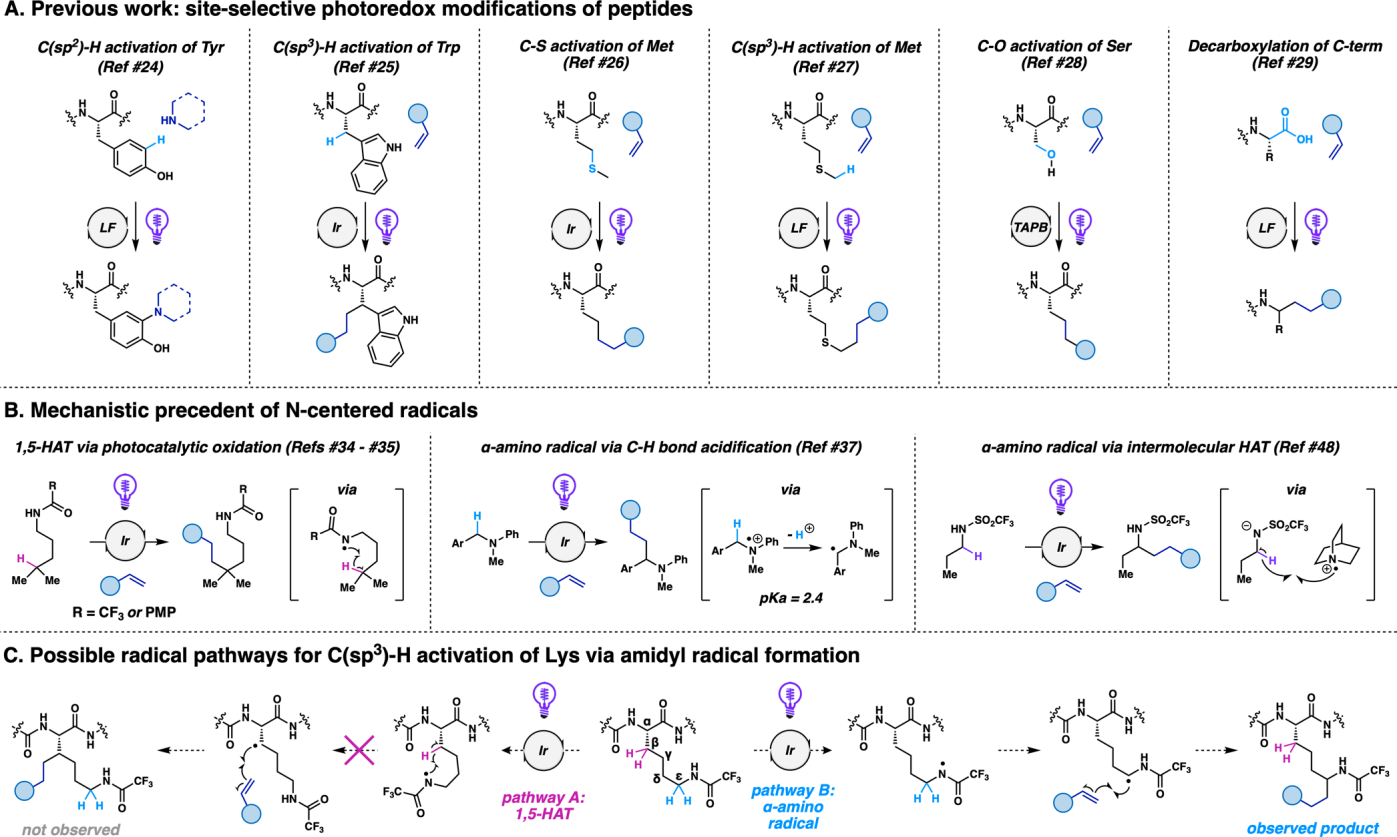
Literature Context and Mechanistic Background^a^

a (A) Representative examples of site-selective photoredox modifications of peptides reported to date, including residue-specific activation of Tyr, Met, Trp, Ser, and C-terminal motifs. (B) Mechanistic precedent for N-centered radical reactivity, including canonical 1,5-hydrogen atom transfer (HAT) processes and previously reported α-amino radical formation pathways. (C) Possible radical pathways for C­(sp^3^)–H activation of lysine via amidyl radical formation, illustrating both distal 1,5-HAT and proximal α-amino radical manifolds. The observed regioselective functionalization is consistent with α-amino radical generation under the reaction conditions. Legend: LF = lumiflavin; Ir = iridium; TAPB = 1,3,5-tris­(4-aminophenyl)­benzene; light bulb represents irradiation; blue ball on alkene handle represents an electron withdrawing group (EWG).

Application of photoredox catalysis to peptide modification has spanned a range of canonical amino acids, but the C­(sp^3^)–H bonds of lysine have remained elusive due to their resistance to radical generation and alkylation. A key challenge to accessing the C–H bonds on the lysine side chain is the high nucleophilicity of the ε-amino group. We reasoned that the nucleophilicity of the nitrogen atom may be reduced with a redox-active electron deficient protecting group. For this protecting group to be compatible with common peptide synthesis methods, i.e., Fmoc-solid phase peptide synthesis (SPPS),^31–33^ it would need to resist secondary amines used for Fmoc deprotection and acids required for the removal of side chain protecting groups. Moreover, the desired protecting group would need to be readily removed under mild conditions to reveal the free amine after the photoredox transformation.

The Rovis and Knowles groups have both independently reported 1,5-hydrogen atom transfer (HAT) methodologies,^34,35^ which use redox-active amides to access N-centered radicals via single-electron oxidation (Scheme 2B). The electrophilic nature of this amidyl radical could readily abstract a remote C­(sp^3^)–H bond to generate a distal nucleophilic carbon-centered radical. We initially hypothesized that placing a trifluoroacetamide (Tfa) protecting group on a Lys residue would generate an amidyl radical that is situated for 1,5-HAT to enable β-C­(sp^3^)–H activation and alkylation. To our surprise, we observed that using a trifluoroacetamide protecting group on Lys enables a pragmatic approach for ε-C­(sp^3^)–H activation and alkylation without the competing β-C­(sp^3^)–H alkylation, leading to a highly regioselective photocatalytic transformation (Scheme 2C).

If a 1,5-HAT pathway is not favorable due to conformational restraints or a high entropic penalty to adopt the necessary 6-membered transition state,^36^ then the other feasible pathway is generation of an α-amino radical from the proximal C­(sp^3^)–H bond of the amino group. Precedent from the Ready group has demonstrated that direct photocatalytic oxidation of tertiary amines to radical cations leads to facile α-amino radical formation and subsequent alkylation (Scheme 2B).^37^ The presence of a heteroatom-centered radical greatly acidifies the adjacent C–H bonds (p*K*_a_ decrease of >15 units) due to radical enhanced deprotonation.^38–42^ In practice, this phenomenon enables a deprotonation event and a 1,2-radical shift to form a stabilized C-centered radical.^43–45^ This nucleophilic α-amino radical can then engage with electron deficient olefins in Giese additions to enable C­(sp^3^)–C­(sp^3^) bond formation.^46,47^ An alternative mechanism for α-amino radical formation was also reported by the Rovis group, where trifluoromethanesulfonamides are used as radical precursors via intermolecular HAT with quinuclidine (Scheme 2B).^48^ We have probed the proposed mechanism and studied the substrate scope of the reaction. Our studies support redox-active amides as versatile handles for radical generation and diversification of the neighboring C–H bonds.

## Results and Discussion

We began our optimization studies by synthesizing a tetrapeptide that contains a Lys­(Tfa) residue. The combination of [Ir­(dFCF_3_ppy)_2_dtbbpy]­PF_6_ (4 mol %) as the photocatalyst, 3.0 equiv of methyl acrylate as the radical acceptor, 2.0 equiv of K_3_PO_4_ as the base, and 10 μL of water as an additive in DMF was irradiated with 427 nm light under inert atmosphere for 18 h (Table 1, entry 1) to furnish the desired alkylated product from activation and modification of the ε-C­(sp^3^)–H bond, in 40% yield. Removing water as an additive that partially solubilizes the base deteriorates the yield to 15% (entry 2). A solvent screen revealed DMSO as the optimal solvent, generating the product in 72% isolated yield (entry 3), while diluting the reaction medium drops the yield to 40% (entry 4). Changing the base to Na_3_PO_4_ reduces the yield to 30% (entry 5), while removing the base entirely leads to 0% yield (entry 6).

**Table 1:**
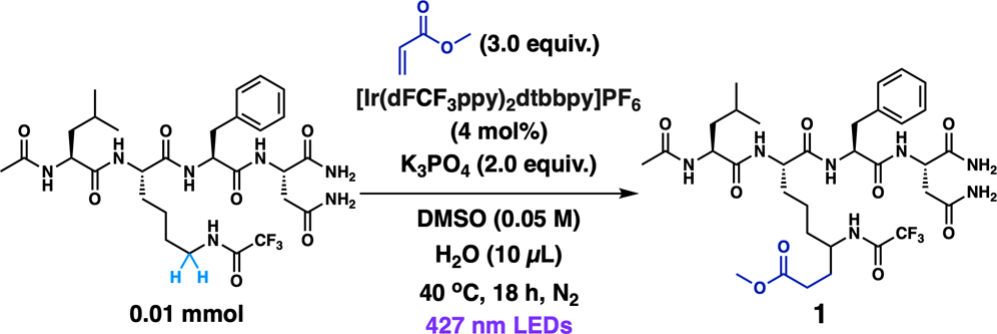
Optimization and Control Reactions

entry	deviation from standard conditions	yield **1** (%)^a^
1	DMF	40
2	DMF, no water	15
3	none	75 (72)^b^
4	0.01 M	40
5	Na_3_PO_4_	30
6	no base	0
7	[Ir(dFMeppy)_2_dtbbpy]PF_6_	35
8	4CzIPN	60
9	no photocatalyst	0
10	no photocatalyst, under air	10
11	*p*-methoxybenzamide as protecting group	0
12	tosyl as protecting group	0
13	acetamide as protecting group	0

a Determined by analysis of ^1^H NMR of the crude reaction mixture using 1,4-dinitrobenzene as an internal standard.

b Isolated yield.

A photocatalyst screen revealed that [Ir­(dFMeppy)_2_dtbbpy]­PF_6_ changes the product distribution by generating a higher amount of oligomerized byproducts (see the Supporting Information for full optimization studies), reducing the desired single adduct to 35% yield (entry 7). A further screen demonstrates that 4CzIPN also diminishes the yield of the desired single adduct to 60% (entry 8). Notably, removing the photocatalyst under an inert atmosphere shuts down the reaction (entry 9), while a 10% yield of the product is obtained in air even in the absence of the photocatalyst (entry 10), implying that singlet oxygen may act as an oxidant in this reaction. Changing the protecting group on the Lys residue from trifluoroacetamide to *p*-methoxybenzamide, tosyl, or acetamide leads to no reaction (entries 11–13). The Knowles group has previously demonstrated that *p*-methoxybenzamides undergo efficient amidyl radical formation and subsequent 1,5-HAT with a highly oxidizing iridium photocatalyst and a specialized dibutyl phosphate base.^35^ The reaction with *p*-methoxybenzamide was not observed under our milder conditions and peptide substrates.

With optimized conditions in hand, we investigated the scope of alkenes that may modify the lysine ε-position. In our preliminary studies, we found that two iridium photocatalysts led to notably different product distributions for methyl acrylate (see Supporting Information for full product distribution details). The two iridium catalysts differ in their redox potentials, reactive properties, and substrate compatibilities.^49^ Based on this result, we developed two complementary conditions and tested both of them across a range of olefin electronics and sterics (Scheme 3A). Condition A uses [Ir­(dFCF_3_ppy)_2_dtbbpy]­PF_6_ as the catalyst and condition B uses [Ir­(dFMeppy)_2_dtbbpy]­PF_6_ as the catalyst. We observed that condition A works best for more reactive electrophiles that are less sterically bulky at the β-carbon, such as unsubstituted acrylates (**1**, **2**), methyl methacrylate (**3**), ethyl vinyl ketone (**5**), dimethyl acrylamide (**7**), and 1,1-diphenyl ethylene (**10**). The reaction can also be scaled up to 0.1 mmol, affording the product (**2**) in 52% isolated yield. For more sterically bulky electrophiles or less electron deficient olefins, condition B provided relatively higher yields. Examples include ethyl crotonate (**4**), methylenenorcamphor (**6**), diethyl vinyl phosphonate (**8**), phenyl vinyl sulfone (**9**), and α-methyl styrene (**11**). In all cases, no diastereoselectivity is observed which is expected of radical Giese additions.^46^ These results suggest that the reaction efficiency depends on a balance between alkene electrophilicity and steric accessibility and that modulation of photocatalyst redox properties allows productive radical addition across a wider range of olefin classes.

**Scheme 3:**
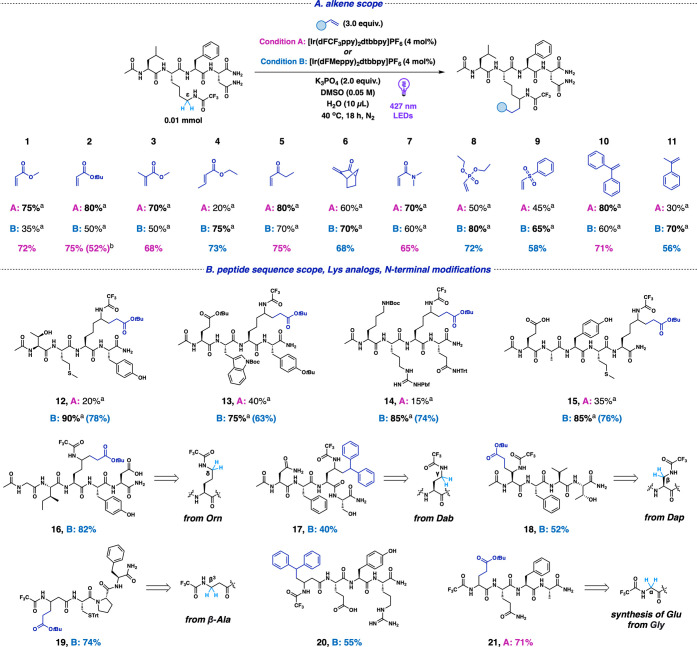
Alkene and Peptide Sequence Scope[Fn s3fn2]

^a^Determined by analysis of ^1^H NMR of the crude reaction mixture using 1,4-dinitrobenzene as an internal standard. ^b^Isolated yield from 0.1 mmol scale reaction, DMSO:H_2_O (19:1) (0.125 M).Isolated yields (bolded and colored) were obtained from either condition A or B, selected based on the higher ^1^H NMR yield.
c Isolated yields (bolded and colored) were obtained from either condition A or B, selected based on the higher ^1^H NMR yield.

Next, we tested the potential of the methodology to modify lysine residues on peptides containing diverse side-chain functional groups (Scheme 3B). The reaction is compatible with several unprotected nucleophilic or oxidizable residues such as Thr, Met, Tyr, and Glu (**12**, **15**). The reaction is also compatible with the most common acid-labile protecting groups used in Fmoc-SPPS, such as Glu­(O*t*Bu), Trp­(Boc), and Tyr­(*t*Bu) (**13**), Arg­(Pbf), and Gln­(Trt) (**14**). The reaction also maintains full selectivity for Lys­(Tfa) in the presence of other lysine protecting groups, i.e., Lys­(Boc) (**14**). This result is significant, as it allows site-selective modification of a single Lys residue in the presence of other Lys residues. In each case, condition B gives the higher yield, demonstrating that the [Ir­(dFMeppy)_2_dtbbpy]­PF_6_ catalyst is essential for productive reactivity when other competing oxidizable residues are present.

Importantly, the reaction is amendable to shorter Lys analogues. The scope includes modification of ornithine (Orn) (**16**) to a δ-branched UAA, diaminobutyric acid (Dab) to a γ-branched UAA (**17**), and diaminopropionic acid (Dap) to a β-branched UAA (**18**), respectively. The reaction also tolerates N-terminal residues that are Tfa-capped, which enables the generation of functionalized β^3^-amino acids at the terminus of peptides containing β-Ala (**19**, **20**). Remarkably, the reaction also works on N-terminal Tfa-Gly residues, which can facilitate α-functionalization of Gly. Example **21** in Scheme 3B illustrates synthesis of glutamic acid from glycine in a single transformation. These cases also show reaction tolerance to unprotected Asp, Ser, and Arg residues, as well as protected Cys­(Trt). This set of substrates effectively expands this chemistry beyond a Lys modification and turns it into a general platform for peptide editing via C­(sp^3^)–H activation at trifluoroacetamide protected amines.

To further expand the utility of this method, we next interrogated placement of the electrophile within the peptide and addition of a small molecule trifluoroacetamide fragment (Scheme 4A). Reaction of an N-terminal acrylamide-capped substrate (1.0 equiv) with *N*-ethyl-2,2,2-trifluoroacetamide (3.0 equiv) allows for the efficient synthesis of a substituted N-terminal γ-amino acid in one step (**22**). Similarly, transformation of a cysteine residue into an electrophilic dehydroalanine (Dha) residue leads to a functional handle that can be trapped to generate a methylated Dab UAA analog (**23**).

**Scheme 4:**
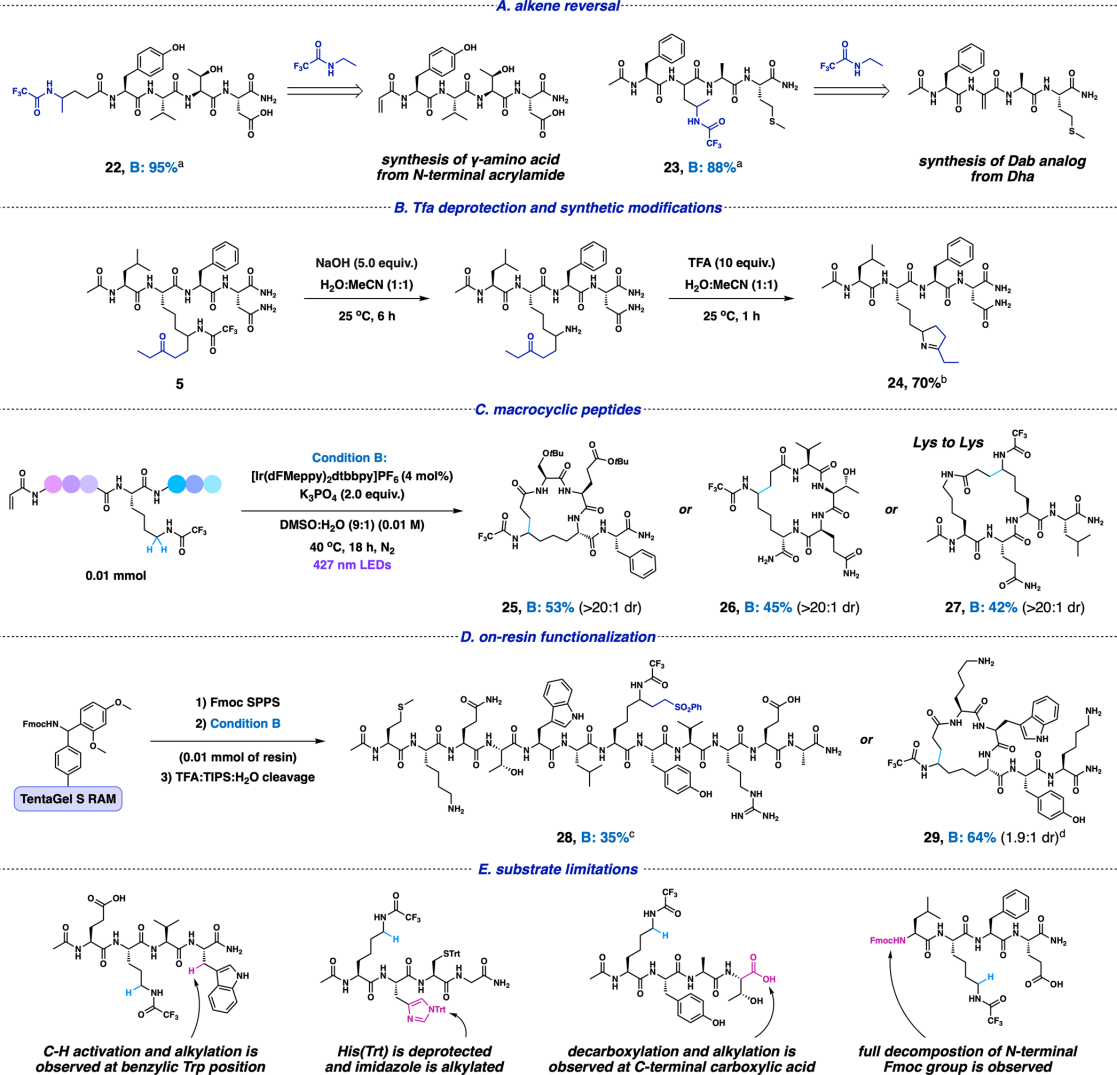
Scope for Alkene Reversal, Peptide Macrocyclization, On-Resin Modification, and Tfa Deprotection[Fn s4fn2]

a Isolated yields are in bold and colored. ^a^The stoichiometry was modified to 1.0 equiv of peptide and 3.0 equiv of trifluoroacetamide. ^b^Isolated yield over 2 step synthesis at 0.01 mmol scale. ^c^DMF (0.05 M) and 20 μL of H_2_O. ^d^DMSO:H_2_O (9:1) (0.01 M). Diastereoselectivity refers to the isolated compound. For on-resin examples, isolated yield includes steps for SPPS and photocatalytic reaction. For substrate limitations, each peptide was subjected to condition B with methyl acrylate.
e Isolated yields are in bold and colored.

The trifluoroacetamide group can be readily deprotected under mild alkaline conditions to reveal the free amine. Scheme 4B illustrates a two-step sequence of basic deprotection followed by acidic cyclization to convert the ketone adduct (**5**) into a heterocyclic imine derivative (**24**). This result demonstrates the practical postsynthetic modifications leading to new UAAs from the photomodified products.

We next assessed the potential of the methodology in an intramolecular context to generate macrocyclic products (Scheme 4C). Macrocyclic peptides have demonstrated promise as peptidomimetic therapeutics due to their high metabolic stability, resistance to proteases, and ability to inhibit protein–protein interactions.^50–55^ We generated a modified version of condition B that employs dilute reactants to prevent intermolecular oligomerization and aid macrocyclization. The reaction was applied to substrates with N-terminal acrylamides to generate head-to-side chain macrocycles, which were isolated as pure diastereomers (**25**, **26**). Use of a Lys­(acrylamide) residue allows generation of Lys to Lys side chain cross-linked macrocycle (**27**), again demonstrating that orthogonal protecting group strategies may be used to change the reactivity of individual Lys residues.

Finally, we focused on developing reaction conditions for the direct on-resin functionalization of peptides (Scheme 4D). Initial screening efforts using a standard Rink amide resin were unsuccessful, leading to no desired product. We discovered that TentaGel S RAM resin is amendable to the photocatalytic conditions, leading to fully protected peptides on bead to be alkylated. Notably, the Lys­(Tfa) residue is convenient to install during Fmoc-SPPS as the Fmoc-Lys­(Tfa)-OH building block is commercially available. The TentaGel resin bearing a 12-mer peptide was directly subjected to condition B with phenyl vinyl sulfone as the electrophile and DMF as the solvent. After irradiation with 427 nm light, the resin-bound peptide was deprotected and cleaved under acidic conditions and subsequently HPLC-purified to afford the functionalized product in 35% isolated yield (**27**). The reaction also works for on-resin macrocyclization. A pentapeptide substrate was first built on the TentaGel S RAM bead with one Lys­(Tfa), two Lys­(Boc) residues, and an N-terminal acrylamide and then subjected to the intramolecular reaction conditions. After cleavage and deprotection, the crude material was purified to give the desired macrocycle in 64% isolated yield and 1.9:1 diastereoselectivity, with full site selectivity of only the photoactive Lys residue (**29**).

Our studies revealed several incompatible functionalities that either do not survive photoredox conditions or outcompete the desired Lys reactivity (Scheme 4E). When unprotected Trp is included in a sequence, competitive benzylic C­(sp^3^)–H activation and alkylation is observed.^25^ The Trp­(Boc) protecting group is necessary to prevent competitive oxidation at the indole nitrogen. By contrast, we postulate that free Tyr residues are tolerated by the reaction conditions since oxidation forms electrophilic tyrosyl radicals which do not engage in Giese additions. Additionally, we observe that His­(Trt) residues are not tolerated since the Trt group gets deprotected and the imidazole undergoes competitive alkylation. The reaction also does not tolerate C-terminal carboxylic acids, as these groups are fully decarboxylated and alkylated under the reaction conditions.^29^ Since the reaction operates under basic conditions, N-terminal Fmoc groups are also prone to deprotection and other forms of decomposition.

The proposed mechanism is consistent with a radical pathway, based on the trends found in the reaction scope and the optimization studies (Scheme 5A). Precedent from the Rovis group has shown that amidyl radicals can be generated from trifluoroacetamides by deprotonation of the acidic N–H proton, followed by single-electron oxidation by an excited state Ir­(III) photocatalyst.^34^ This pathway is consistent with the Lys­(Tfa) system as they both use the same base and photocatalyst. In the Lys system, no product from the 1,5-HAT pathway is observed, potentially due to conformational constraints to achieve the 6-membered transition state for abstraction.^36^ Steric clash between the Tfa protecting group and the peptide backbone could be a possible factor that increases the entropic penalty to achieve the required transition state for the 1,5-HAT pathway.

**Scheme 5:**
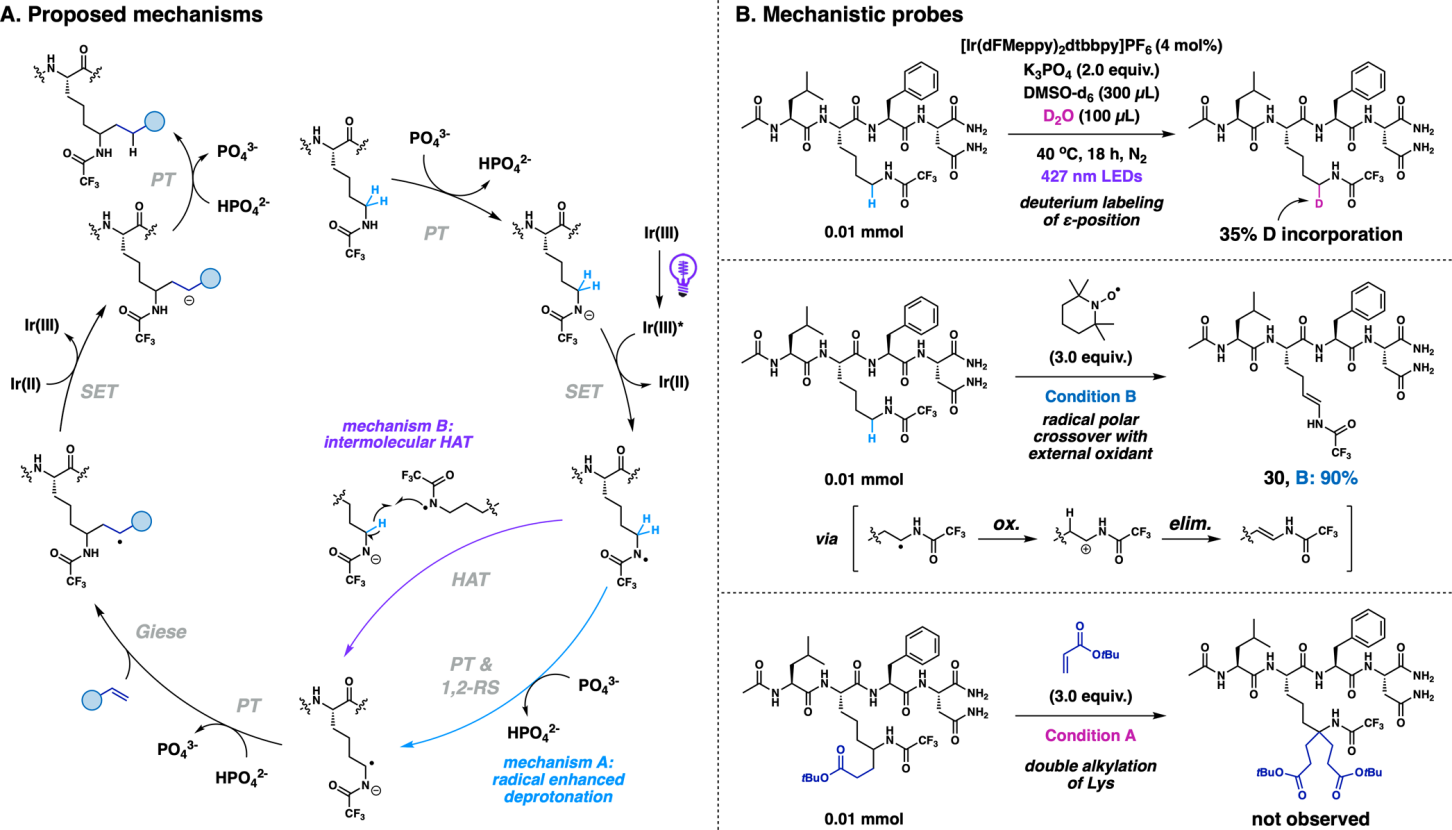
Plausible Catalytic Cycles^a^

a Deuterium incorporation percentage determined by ^1^H NMR analysis of recovered starting material after subjecting it to reaction conditions. For the radical polar crossover experiment, the trifluoroenamide product is not observed when the photocatalyst is removed. Legend: PT = proton transfer; SET = single-electron transfer; 1,2-RS = 1,2-radical shift; HAT = hydrogen atom transfer.

A plausible pathway, denoted as mechanism A, involves deprotonation of the adjacent C­(sp^3^)–H bond relative to the amidyl radical and a 1,2-radical shift to generate an α-amino radical.^38–43^ Based on radical polarity studies, this type of radical has been characterized as nucleophilic.^56^ The other feasible pathway for α-amino formation would be intermolecular HAT of the amidyl radical to abstract the C­(sp^3^)–H bond from another equivalent of trifluoroacetamide, denoted as mechanism B.^48^ We postulate that the generated radical can then participate in a Giese addition into an electron deficient olefin to generate another stabilized carbon-centered radical. Importantly, the newly formed stabilized radical can either be reduced by Ir­(II) to form an anion that can be quenched with a proton to form the single adduct product, or alternatively add into another equivalent of olefin to generate an oligomerized byproduct before reduction (see Supporting Information for proposed off-cycle mechanistic pathways). To our knowledge, while α-amino radicals have been generated via a variety of photoredox strategies,^37,44,48,57–59^ trifluoroacetamide substrates have not been previously exploited as direct precursors for α-amino radical formation under photoredox catalysis.

In order to further probe the reaction mechanism, we conducted a deuterium labeling experiment on peptide sequence used for optimization studies (Scheme 5B). We subjected the peptide to a modified version of condition B in dilute 3:1 mixture of DMSO-*d*_6_:D_2_O in the absence of an alkene. Under these conditions, a majority of the starting material was recovered and 35% deuterium incorporation was observed at the ε-C­(sp^3^)–H position. This result suggests an α-amino radical is generated adjacent to the trifluoroacetamide. The deuterium can potentially be incorporated by reduction of the radical to an anion by Ir­(II) followed by protonation by D_2_O. Alternatively, the incorporation could potentially occur via intermolecular HAT with the deuterated solvent.

Next, we treated the same peptide sequence with 3.0 equiv of TEMPO under condition B. Rather than observing the trapped TEMPO adduct, we isolated an oxidized trifluoroenamide (**30**) in 90% yield. We propose that TEMPO acts as an external sacrificial oxidant in this reaction to enable radical polar crossover to a carbocation from the α-amino radical, which can then eliminate under basic conditions to form the stable enamide. This product is not observed when photocatalyst is not added. This reaction enables the facile oxidation of the Lys side chain to an unsaturated UAA, which provides an attractive complementary method of enamide synthesis directly from amides compared to other existing methods.^60^ As a final mechanistic probe, the monoalkylated product (**2**) was resubjected to condition A and no double alkylation at the ε-C­(sp^3^)–H position was observed. This result suggests that the product cannot reenter the catalytic cycle, thereby ensuring high selectivity for monoalkylation. Multialkylated products are formed by radical chain propagation and oligomerization via an off-cycle pathway (see Supporting Information for extended mechanism).

Mechanistically, the phenomenon that is consistently observed across conditions A and B can be rationalized in terms of radical lifetime. We hypothesize that the varying product distributions of unreacted starting material, single adduct, and multiply oligomerized byproducts are determined by kinetic control. The relative rates of radical reduction by Ir­(II) after Giese addition vs propagation of the radical to add into another equivalent of alkene change the observed product distributions. We propose that [Ir­(dFCF_3_ppy)_2_dtbbpy]­PF_6_ leads to faster reduction of the radical to a carbanion, while [Ir­(dFMeppy)_2_dtbbpy]­PF_6_ is slower at reduction, leading to a longer-lived radical. This observation can potentially explain why condition B is accommodating of less electrophilic olefins and peptide sequences with residues that can compete for single-electron oxidation such as Met, Tyr, and Glu. For condition A, fast reduction is required for potent electrophiles, such as unsubstituted acrylates, to prevent rapid oligomerization before catalytic turnover.

## Conclusions

In summary, we have developed a photoredox-catalyzed peptide editing strategy that enables site-selective C­(sp^3^)–H functionalization of lysine and related primary amine residues through α-amino radical generation from trifluoroacetamides. By leveraging a redox-active protecting group that is fully compatible with standard Fmoc-based solid phase peptide synthesis, this method overcomes long-standing challenges associated with the inherent nucleophilicity and conformational flexibility of lysine side chains. The transformation operates under mild conditions, exhibits broad functional group tolerance, and functions across a diverse range of amino acid sequences and alkene coupling partners.

Beyond lysine modification, this chemistry generalizes to ornithine, diaminobutyric acid, diaminopropionic acid, β-alanine, and glycine, thereby providing streamlined access to a variety of branched and otherwise inaccessible unnatural amino acid motifs. The methodology further enables alkene-reversed reactivity for functionalization of acrylamides and dehydroalanine residues, intramolecular macrocyclization to generate cyclic peptides, direct on-resin modifications, and oxidation to unsaturated side chains. Collectively, these features highlight the versatility of this photoredox chemistry as a potentially powerful approach to peptide diversification. More broadly, this work establishes redox-active amide protecting groups as programmable elements for site-selective radical generation in complex peptide environments. We anticipate that the approach will facilitate rapid late-stage diversification of therapeutic peptides, enable new approaches to macrocyclization and conjugation, and lead to the development of additional redox-active functional handles for selective C–H editing in biomolecular settings.

## Supplementary Material



## Notes

Experimental materials, methods, characterization of new compounds, and NMR and HRMS spectra (PDF)
