# Supplementary material for: Photoredox-Catalyzed Lysine C(sp3)–H Functionalization for Peptide Editing
Source: J Am Chem Soc. 2026 Jun 24;148(26):27169–77. doi: 10.1021/jacs.6c02967 (PMC13352616; doi:10.1021/jacs.6c02967)
Supplement: Supplementary file 1 [file ja6c02967_si_001.pdf]

Supporting Information

Photoredox-Catalyzed Lysine C(sp^3)–H Functionalization for Peptide Editing

Christopher W. Lamartina and Paramjit S. Arora*

Department of Chemistry, New York University, New York, New York 10003, United States

Table of Contents

1. General information S2
2. Proposed mechanisms of photoredox-catalyzed C(sp^3)-H activation with off-cycle pathways S3
3. Extended optimization studies S4
4. Substrate limitations S7
5. Experimental methods 1: Starting material synthesis and characterization S8
6. Experimental methods 2: Product synthesis and characterization S15
7. Pictures of photoredox experimental setup S26
8. NMR and HRMS spectra S28
9. References S106

1. General information

Unless otherwise noted, all reactions were performed without precaution to air or moisture. Commercial reagents and HPLC grade solvents were purchased from Sigma-Aldrich, Fisher Scientific, and Ambeed. ^{1}H-NMR spectra were recorded on Bruker 500 or 600 MHz spectrometers equipped with cryoprobe at ambient temperature. Chemical shifts are reported in parts per million (ppm) in respective NMR solvent (DMSO-d_6 ref. @ 2.50 ppm) with multiplicity (s = singlet, d = doublet, t = triplet, q = quartet, and m = multiplet) and coupling constants (Hz). Each product spectrum is compared to its corresponding spectrum for direct comparison of diagnostic resonances and to confirm regioselectivity. High resolution mass spectra (HRMS) were obtained from Waters ACQUITY/Xevo G3 LC-QToF MS using the ESI+ ionization model. MALDI analysis was accomplished on Bruker AutoFlex Max MALDI-TOF instrument. High performance liquid chromatography (preparative) was performed on a Thermo Scientific UltiMate 3000 instrument with reversed-phase C18 column. High performance liquid chromatography (analytical) was performed on an Agilent 1260 instrument with reversed-phase C18 column. A Labconco lyophilizer was used to freeze-dry samples.

2. Proposed mechanisms of photoredox-catalyzed C(sp^3)-H activation with off-cycle pathways

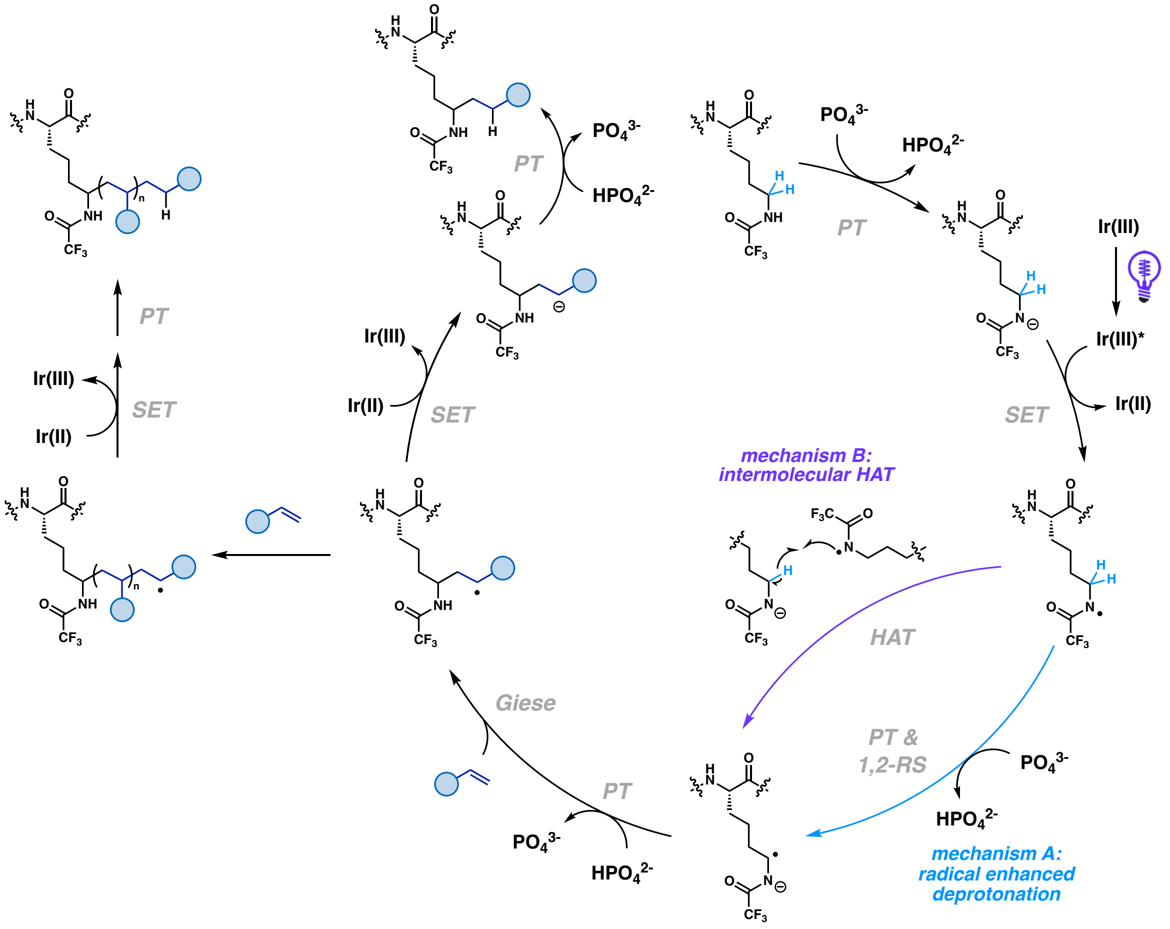

The proposed reaction mechanism in the main text is further elaborated on in this figure. The carbon-centered radical that is formed after the first Giese addition step can either be directly reduced by Ir(II) and subsequently protonated to form the single adduct product, or the radical can propagate to add into additional equivalents of alkene to oligomerize before reduction and catalytic turnover. This proposed mechanism explains the generation of byproducts that are observed by MALDI analysis of crude reaction mixtures. Our analysis suggests that the relative rates of radical reduction vs. rate of propagation to oligomerize is under kinetic control and varies based on the redox properties of the selected photocatalyst for a given substrate combination.

3. Extended optimization studies

Product yields of the single adduct are determined by ^{1}H-NMR analysis of crude reaction mixtures by using 1,4-dinitrobenzene as internal standard, product distribution ratios (unreacted starting material, single adduct, and oligomers) are determined by MALDI analysis of the crude reaction mixtures.

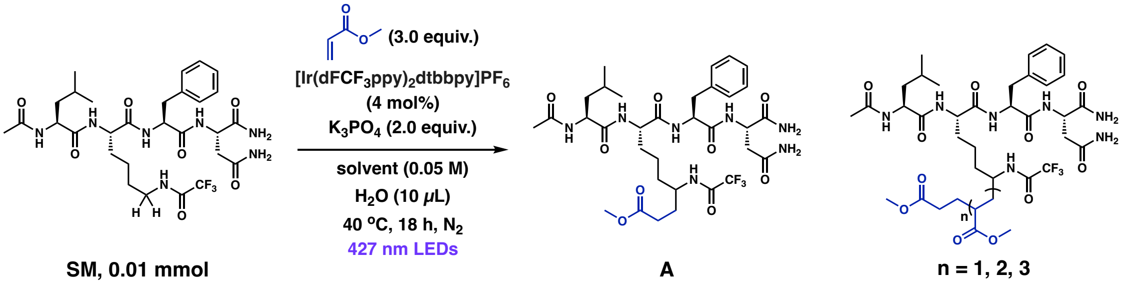

Solvent	Unreacted SM	A	n = 1	n = 2	n = 3
DMF	50%	40%	10%	0%	0%
DMSO	10%	75%	10%	5%	0%
DMSO:$PhCF_3$ (1:1)	85%	10%	5%	0%	0%
DMF:$PhCF_3$ (1:1)	95%	5%	0%	0%	0%
MeCN:$PhCF_3$ (1:1)	100%	0%	0%	0%	0%
MeCN	100%	0%	0%	0%	0%
DMSO:DMF (1:1)	60%	30%	10%	0%	0%

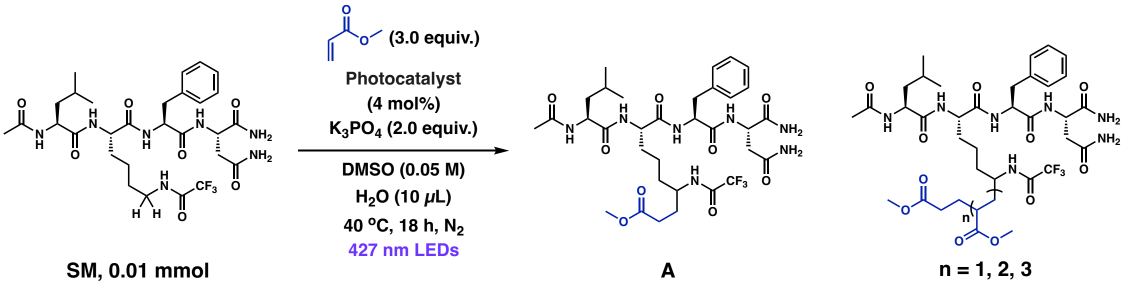

Photocatalyst	Unreacted SM	A	n = 1	n = 2	n = 3
$[Ir(dFCF_3ppy)_2dtbbpy]PF_6$	10%	75%	10%	5%	0%
$[Ir(dFMeppy)_2dtbbpy]PF_6$	0%	35%	30%	25%	10%
$Ir(ppy)_3$	100%	0%	0%	0%	0%
4CzIPN	20%	60%	15%	5%	0%
none	100%	0%	0%	0%	0%
none, under air	90%	10%	0%	0%	0%

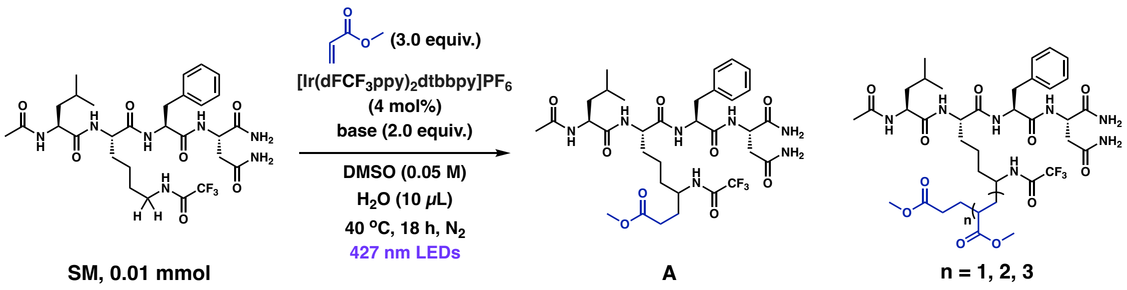

Base	Unreacted SM	A	n = 1	n = 2	n = 3
K_3PO_4	10%	75%	10%	5%	0%
K_2HPO_4	100%	0%	0%	0%	0%
Na_3PO_4	60%	30%	10%	0%	0%
K_2CO_3	85%	10%	5%	0%	0%
Na_2CO_3	80%	10%	10%	0%	0%
TMG	100%	0%	0%	0%	0%
BTMG	100%	0%	0%	0%	0%
quinuclidine	100%	0%	0%	0%	0%
none	100%	0%	0%	0%	0%

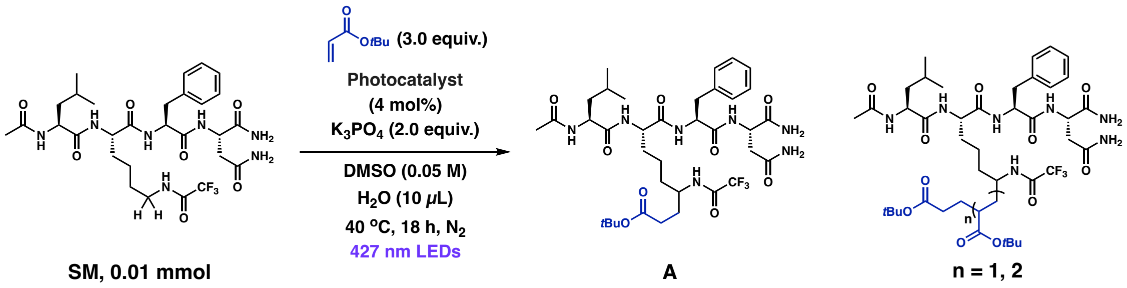

Photocatalyst	Unreacted SM	A	n = 1	n = 2
$[Ir(dFCF_3ppy)_2dtbbpy]PF_6$	10%	80%	10%	0%
$[Ir(dFMeppy)_2dtbbpy]PF_6$	0%	50%	40%	10%

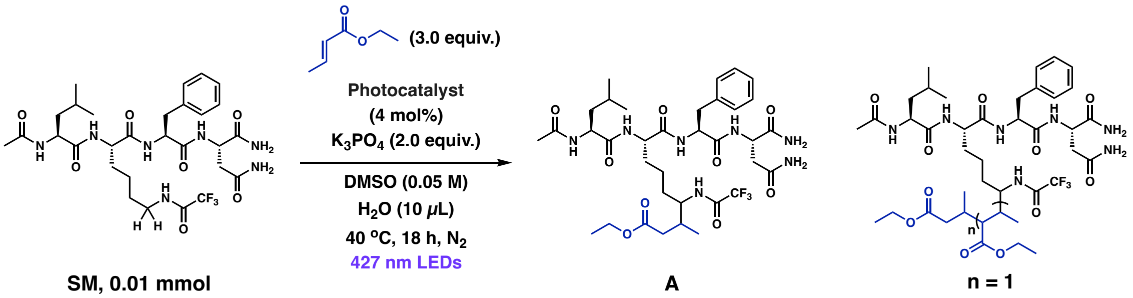

Photocatalyst	Unreacted SM	A	n = 1
[Ir(dFCF$_3$ppy)$_2$dtbbpy]PF$_6$	80%	20%	0%
[Ir(dFMeppy)$_2$dtbbpy]PF$_6$	25%	75%	0%

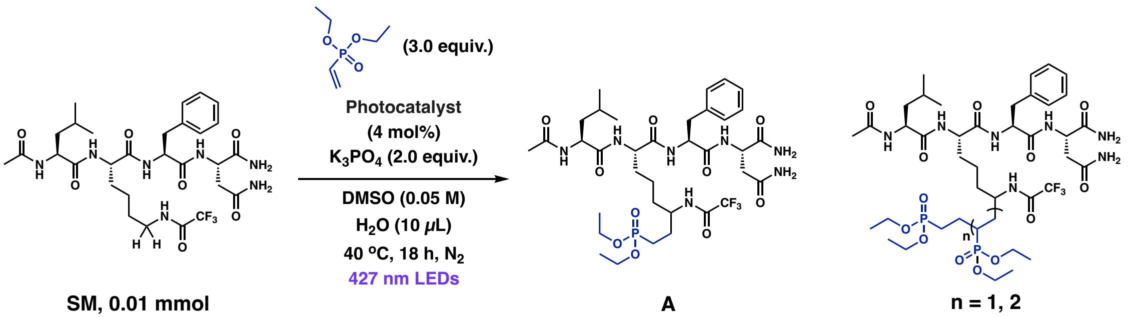

Photocatalyst	Unreacted SM	A	n = 1	n = 2
[Ir(dFCF$_3$ppy)$_2$dtbbpy]PF$_6$	40%	50%	10%	0%
[Ir(dFMeppy)$_2$dtbbpy]PF$_6$	5%	80%	10%	5%

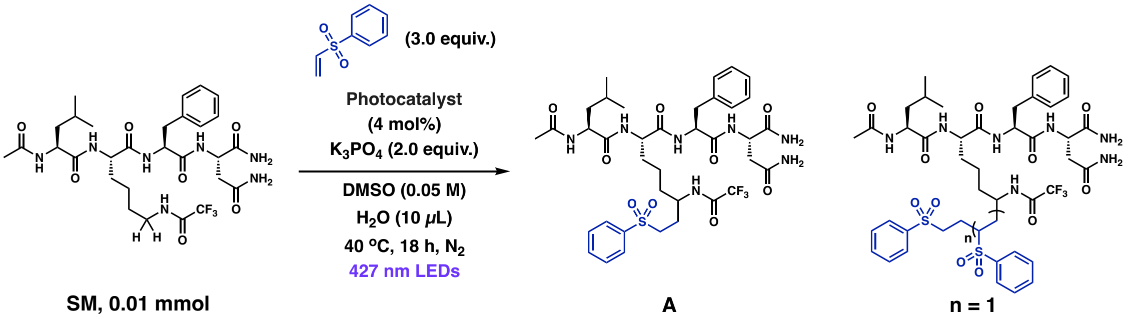

Photocatalyst	Unreacted SM	A	n = 1
[Ir(dFCF$_3$ppy)$_2$dtbbpy]PF$_6$	55%	45%	0%
[Ir(dFMeppy)$_2$dtbbpy]PF$_6$	35%	65%	0%

4. Substrate limitations

The following electrophiles did not work with the optimized conditions or with any photocatalyst. Styrenes require α-substitution to work effectively in the reaction conditions.

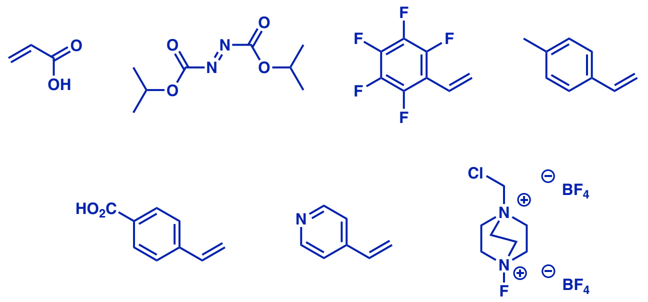

The following peptide sequences gave complex product mixtures. The substrate limitations are further elaborated on in the main text.

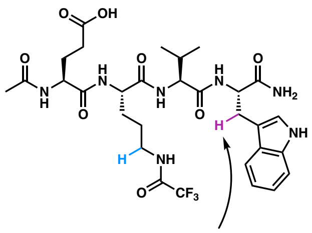

C-H activation and alkylation is observed at benzylic Trp position

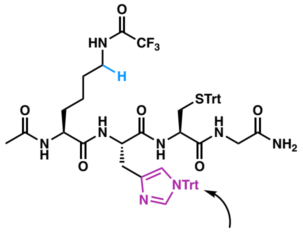

His(Trt) is deprotected and imidazole is alkylated

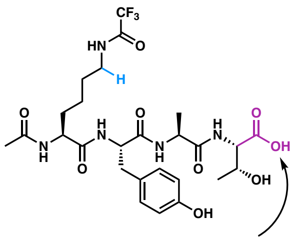

decarboxylation and alkylation is observed at C-terminal carboxylic acid

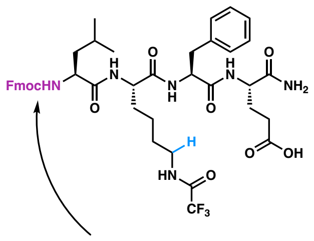

full decompostion of N-terminal Fmoc group is observed

5. Experimental methods 1: Starting material synthesis and characterization

General Procedure A – *Synthesis of starting materials via solid phase peptide synthesis (manual)*

Part 1: This protocol has been adapted from a previous report.[1] To a 50 mL solid phase peptide synthesis vessel equipped with a magnetic stir bar was added 0.3 mmol of Rink amide resin, Sieber amide resin, or TentaGel S RAM resin. The resin (1.0 equiv.) was swelled with DCM and stirred for 20 min, then the vessel was drained using house vacuum and waste collection flask. To deprotect the Fmoc of the resin, 20% piperidine in DMF was added to the vessel and then stirred for 20 min. The vessel was drained and the resin was flow washed x 2 with DMF, followed by DMF batch wash x 1. To couple an amino acid, Fmoc-AA (5.0 equiv.), HBTU or HATU (4.9 equiv.), and HOBt hydrate (4.9 equiv.) dissolved in DMF was added to vessel, DIPEA (10.0 equiv.) was added to activate the reagents, and then the mixture was stirred for 30 min. The vessel was drained and the resin was flow washed x 2 with DMF, followed by DMF batch wash x 1. This iterative deprotection/coupling process was repeated to furnish desired sequence until final deprotection of N-terminus. For Lys(Tfa) installation, the commercially available Fmoc-Lys(Tfa)-OH amino acid was implemented. Similarly, Fmoc-Orn(Tfa)-OH, Fmoc-Dab(Tfa)-OH, or Fmoc-Dap(Tfa)-OH were used to synthesize related analogs.

Part 2: For acetamide (Ac) capped N-termini, the final Fmoc-protected residue is deprotected followed by addition with Ac_2O (20.0 equiv.) and DIPEA (40.0 equiv.) in DMF and then stirred for 30 min. The resin was flow washed x 2 with DMF, followed by DMF batch wash x 1.

For N-terminal acryloyl capping, the deprotected resin was washed with DCM and then suspended in DCM. To this suspension was added triethylamine (2.0 equiv.) followed by slow addition of acryloyl chloride (2.0 equiv.) dissolved in DCM and then stirred for 30 min. The vessel was drained and the resin was flow washed x 2 with DCM, followed by DCM batch wash x 1.

For N-terminal trifluoroacetamide (Tfa) capping, the deprotected resin was treated with ethyl trifluoroacetate (20.0 equiv.) and DIPEA (40.0 equiv.) in DMF and then stirred for 30 min. The resin was flow washed x 2 with DMF, followed by DMF batch wash x 1.

Part 3: Acidic cleavage of the Rink amide resin or TentaGel S RAM resin was accomplished by utilizing a 95:2.5:2.5 mixture of TFA:TIPS:H_2O. The solution was added to capped resin in the vessel and then stirred for 3 h. The solution was directly drained from the vessel into a 20 mL scintillation vial, followed by multiple washes with DCM to ensure quantitative transfer. The volatiles were removed by rotary evaporator until highly concentrated and the crude residue was then added into diethyl ether in a 50 mL centrifuge tube to precipitate the peptide. The sample was then spun down in a centrifuge and the ether was decanted off to remove any scavengers. More ether was added to the precipitated peptide and then spun down again, this process was repeated 4 times. The crude material was then dissolved in MeCN:H_2O and purified via preparative HPLC reverse phase chromatography. The purified material was then flash frozen in liquid nitrogen and freeze dried using a lyophilizer instrument. This protocol is recommended when deprotection of acid-labile side chain protecting groups is necessary.

Acidic cleavage of the Sieber amide resin was accomplished by utilizing a 98:2 mixture of DCM:TFA. The solution was added to capped resin in the vessel and then stirred for 30 min. The solution was directly drained from the vessel into a round bottom flask, followed by multiple washes with DCM to ensure quantitative transfer. This process was repeated 4 times, and the combined cleavage solution was concentrated by rotary evaporator. The crude residue was then added into diethyl ether in a 50 mL centrifuge tube to precipitate the peptide. The sample was then spun down in a centrifuge and the ether was decanted off. The crude material was then dissolved in MeCN:H_2O and purified via preparative HPLC reverse phase chromatography. The purified material was then flash frozen in liquid nitrogen and freeze dried using a lyophilizer instrument. This protocol is recommended when acid-labile side chain protecting groups need to be kept intact. Note that heating the cleavage solution on the rotovap for too long can lead to partial deprotection of the side chains.

General Procedure B – *Synthesis of tetrapeptide containing a Dha residue*

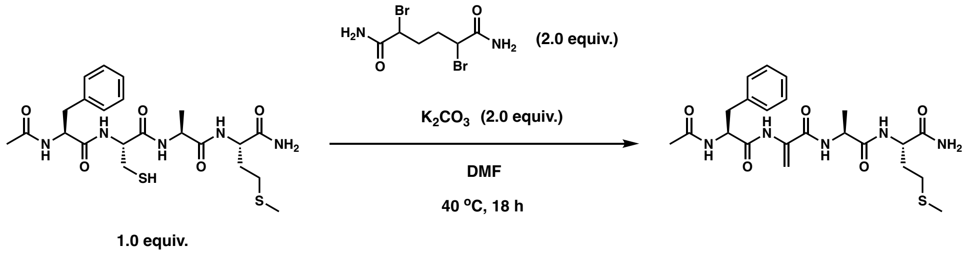

This protocol has been adapted from a previous report.[2] A tetrapeptide containing an unprotected Cys residue was synthesized using General Procedure A (Rink amide resin). A round bottom flask equipped with a magnetic stir bar was charged with the crude peptide (1.0 equiv) dissolved in DMF and then 2,5-dibromohexanediamide (2.0 equiv) and K_2CO_3 (2.0 equiv) were added to the solution. The reaction was stirred over night at 40 °C and then quenched with water and TFA (3.0 equiv). The crude solution was purified via preparative HPLC reverse phase chromatography. The purified material was then flash frozen in liquid nitrogen and freeze dried using a lyophilizer instrument.

General Procedure C – *Synthesis of tetrapeptide containing a Lys(acrylamide) residue*

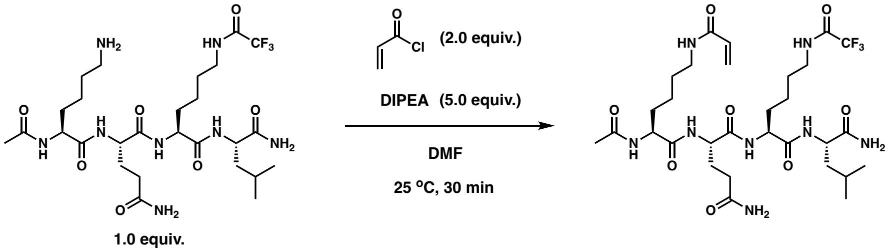

A tetrapeptide containing an unprotected Lys residue that was derived from Lys(Boc) and a protected Lys(Tfa) residue was synthesized using General Procedure A (Rink amide resin). A round bottom flask equipped with a magnetic stir bar was charged with the crude peptide (1.0 equiv) dissolved in DMF. The reaction mixture was charged with DIPEA (5.0 equiv.) and acryloyl chloride (2.0 equiv.) and stirred for 30 min at room temperature, and then quenched with water and TFA (3.0 equiv). The crude solution was purified via preparative HPLC reverse phase chromatography. The purified material was then flash frozen in liquid nitrogen and freeze dried using a lyophilizer instrument.

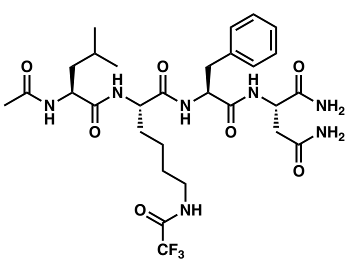

Ac-*L*-Leu-*L*-Lys(Tfa)-*L*-Phe-*L*-Asn-NH2 (Compound A1). Prepared according to General Procedure A from Rink amide resin (0.3 mmol). Purified via prep HPLC C18 reverse phase column (30% --> 60% MeCN/H_2O w/ 0.1% TFA). White solid (0.1498 g, 76% isolated yield). **^{1}H NMR** (600 MHz, DMSO) δ 9.38 (t, *J* = 5.7 Hz, 1H), 8.07 (d, *J* = 8.0 Hz, 1H), 7.95 (dd, *J* = 16.2, 7.9 Hz, 2H), 7.87 (d, *J* = 7.9 Hz, 1H), 7.32 – 7.28 (m, 1H), 7.26 – 7.15 (m, 5H), 7.04 (s, 1H), 6.93 – 6.89 (m, 1H), 6.87 – 6.83 (m, 1H), 4.49 – 4.38 (m, 2H), 4.25 (ddd, *J* = 9.5, 7.9, 5.5 Hz, 1H), 4.15 (ddd, *J* = 9.0, 7.8, 5.1 Hz, 1H), 3.13 (q, *J* = 7.0 Hz, 2H), 3.03 (dd, *J* = 14.0, 4.8 Hz, 1H), 2.81 (dd, *J* = 14.0, 9.2 Hz, 1H), 2.48 (d, *J* = 6.1 Hz, 1H), 2.42 (dd, *J* = 15.5, 6.8 Hz, 1H), 1.83 (s, 3H), 1.63 – 1.54 (m, 2H), 1.50 – 1.34 (m, 5H), 1.24 – 1.14 (m, 2H), 0.85 (dd, *J* = 25.1, 6.6 Hz, 6H). **HRMS** $C_{29}H_{42}F_3N_7O_7$: calculated (M+H)+ m/z 658.3176; found (M+H)+ m/z 658.3180.

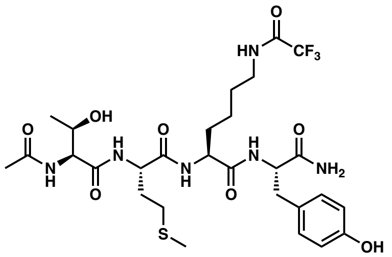

Ac-*L*-Thr-*L*-Met-*L*-Lys(Tfa)-*L*-Tyr-NH2 (Compound A2). Prepared according to General Procedure A from Rink amide resin (0.3 mmol). Purified via prep HPLC C18 reverse phase column (30% --> 60% MeCN/H2O w/ 0.1% TFA). White solid (0.1648 g, 81% isolated yield). **^{1}H NMR** (600 MHz, DMSO) δ 9.38 (t, *J* = 5.7 Hz, 1H), 9.12 (s, 1H), 7.91 (dd, *J* = 7.8, 5.7 Hz, 2H), 7.82 (d, *J* = 8.0 Hz, 1H), 7.71 (d, *J* = 8.1 Hz, 1H), 7.25 (d, *J* = 2.1 Hz, 1H), 7.01 (d, *J* = 2.2 Hz, 1H), 6.99 – 6.94 (m, 2H), 6.66 – 6.59 (m, 2H), 4.87 (s, 1H), 4.33 (dtd, *J* = 24.5, 8.3, 5.1 Hz, 2H), 4.20 – 4.09 (m, 2H), 4.00 – 3.90 (m, 1H), 3.13 (q, *J* = 6.9 Hz, 2H), 2.85 (dd, *J* = 13.9, 5.4 Hz, 1H), 2.70 (dd, *J* = 13.9, 8.2 Hz, 1H), 2.48 – 2.37 (m, 2H), 2.01 (s, 3H), 1.91 (s, 4H), 1.79 (dtd, *J* = 13.9, 9.5, 5.0 Hz, 1H), 1.59 (ddt, *J* = 13.3, 10.8, 5.6 Hz, 1H), 1.50 – 1.38 (m, 3H), 1.25 – 1.17 (m, 2H), 1.04 (d, *J* = 6.4 Hz, 3H). **HRMS** $C_{28}H_{41}F_3N_6O_8S$: calculated (M+H)+ m/z 679.2737; found (M+H)+ m/z 679.2738.

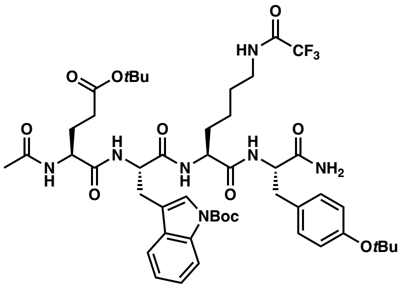

Ac - *L* - Glu(O*t*Bu) - *L* - Trp(Boc) - *L* - Lys(Tfa) - *L* - Tyr(*t*Bu) - NH2 (Compound A3). Prepared according to General Procedure A from Sieber amide resin (0.3 mmol). Purified via prep HPLC C18 reverse phase column (50% --> 80% MeCN/H2O w/ 0.1% TFA). White solid (0.1518 g, 52% isolated yield). **^{1}H NMR** (600 MHz, DMSO) δ 9.36 (t, *J* = 5.7 Hz, 1H), 8.06 – 7.91 (m, 4H), 7.70 (d, *J* = 8.3 Hz, 1H), 7.67 – 7.62 (m, 1H), 7.53 (s, 1H), 7.29 (ddd, *J* = 8.4, 7.1, 1.3 Hz, 1H), 7.25 – 7.17 (m, 2H), 7.12 – 7.06 (m, 3H), 6.82 – 6.77 (m, 2H), 4.56 (ddd, *J* = 9.6, 7.4, 4.0 Hz, 1H), 4.36 (td, *J* = 8.7, 4.9 Hz, 1H), 4.16 – 4.11 (m, 1H), 4.03 (ddd, *J* = 9.3, 7.4, 5.0 Hz, 1H), 3.12 – 3.04 (m, 3H), 3.01 – 2.92 (m, 2H), 2.75 (dd, *J* = 13.9, 9.1 Hz, 1H), 2.15 (t, *J* = 7.9 Hz, 2H), 1.80 (s, 4H), 1.70 – 1.63 (m, 1H), 1.60 (s, 9H), 1.54 – 1.47 (m, 1H), 1.45 – 1.37 (m, 3H), 1.34 (s, 9H), 1.20 (s, 9H), 1.12 – 1.01 (m, 2H). **HRMS** $C_{48}H_{66}F_3N_7O_{11}$: calculated (M+H)+ m/z 974.4851; found (M+H)+ m/z 974.4838.

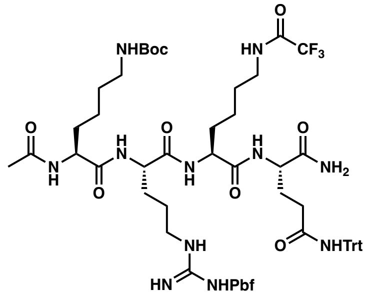

Ac - *L* - Lys(Boc) - *L* - Arg(Pbf) - *L* - Lys(Tfa) - *L* - Gln(Trt) - NH2 (Compound A4). Prepared according to General Procedure A from Sieber amide resin (0.3 mmol). Purified via prep HPLC C18 reverse phase column (50% --> 80% MeCN/H2O w/ 0.1% TFA). White solid (0.2320 g, 60% isolated yield). **^{1}H NMR** ^{1}H NMR (500 MHz, DMSO) δ 9.38 (t, *J* = 5.8 Hz, 1H), 8.61 (s, 1H), 7.97 (dd, *J* = 7.7, 4.1 Hz, 2H), 7.84 (d, *J* = 7.6 Hz, 1H), 7.76 (d, *J* = 7.9 Hz, 1H), 7.25 (t, *J* = 7.6 Hz, 6H), 7.22 – 7.09 (m, 10H), 7.03 (s, 1H), 6.71 (t, *J* = 5.7 Hz, 1H), 6.35 (s, 1H), 4.23 – 4.05 (m, 4H), 3.13 (q, *J* = 6.9 Hz, 2H), 3.00 (q, *J* = 6.5 Hz, 2H), 2.95 (s, 2H), 2.87 (dtd, *J* = 9.9, 7.1, 3.6 Hz, 2H), 2.47 (s, 3H), 2.42 (s, 3H), 2.36 – 2.23 (m, 2H), 2.00 (s, 3H), 1.81 (s, 4H), 1.68 (dq, *J* = 21.5, 8.4, 6.8 Hz, 3H), 1.62 – 1.44 (m, 6H), 1.40 (s, 9H), 1.36 (s, 9H), 1.35 – 1.16 (m, 7H). **HRMS** $C_{64}H_{86}F_3N_{11}O_{12}S$: calculated (M+H)+ m/z 1290.6209; found (M+H)+ m/z 1290.6207.

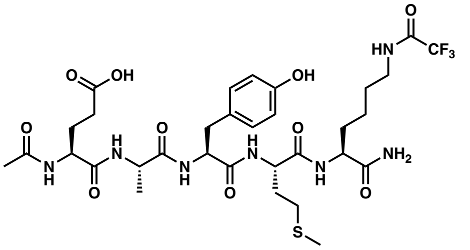

Ac-*L*-Glu-*L*-Ala-*L*-Tyr-*L*-Met-*L*-Lys(Tfa)-NH2 (Compound A5). Prepared according to General Procedure A from Rink amide resin (0.3 mmol). Purified via prep HPLC C18 reverse phase column (30% --> 60% MeCN/H2O w/ 0.1% TFA). White solid (0.1678 g, 72% isolated yield). **^{1}H NMR** (500 MHz, DMSO) δ 12.07 (s, 1H), 9.38 (t, *J* = 5.8 Hz, 1H), 9.12 (s, 1H), 8.03 (dd, *J* = 11.1, 7.2 Hz, 2H), 7.90 (dd, *J* = 7.9, 4.9 Hz, 2H), 7.75 (d, *J* = 7.9 Hz, 1H), 7.26 (s, 1H), 7.00 (d, *J* = 7.7 Hz, 3H), 6.63 (d, *J* = 8.1 Hz, 2H), 4.33 (dtd, *J* = 22.0, 8.3, 4.8 Hz, 2H), 4.24 – 4.08 (m, 3H), 3.15 (q, *J* = 6.8 Hz, 2H), 2.91 (dd, *J* = 14.1, 4.6 Hz, 1H), 2.72 (dd, *J* = 14.2, 9.1 Hz, 1H), 2.42 (td, *J* = 9.3, 5.8 Hz, 2H), 2.24 (t, *J* = 7.9 Hz, 2H), 2.03 (s, 3H), 1.95 – 1.76 (m, 6H), 1.75 – 1.61 (m, 2H), 1.55 – 1.44 (m,

3H), 1.29 – 1.23 (m, 2H), 1.15 (d, *J* = 7.1 Hz, 3H). **HRMS** $C_{32}H_{46}F_3N_7O_{10}S$: calculated (M+H)+ m/z 778.3057; found (M+H)+ m/z 778.3066.

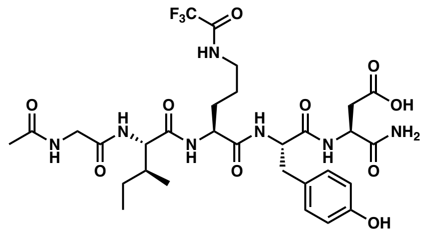

Ac-Gly-*L*-Ile-*L*-Orn(Tfa)-*L*-Tyr-*L*-Asp-NH_2 (Compound A6). Prepared according to General Procedure A from Rink amide resin (0.3 mmol). Purified via prep HPLC C18 reverse phase column (30% --> 60% $MeCN/H_2O$ w/ 0.1% TFA). White solid (0.0925 g, 43% isolated yield). **^{1}H NMR** (500 MHz, DMSO) δ 9.36 (t, *J* = 5.6 Hz, 1H), 8.19 (d, *J* = 7.8 Hz, 1H), 8.02 (t, *J* = 7.5 Hz, 2H), 7.81 (d, *J* = 7.8 Hz, 1H), 7.36 (s, 1H), 7.26 – 7.16 (m, 6H), 7.11 (s, 1H), 6.90 (s, 1H), 4.90 (s, 1H), 4.51 (q, *J* = 7.2 Hz, 1H), 4.42 (ddd, *J* = 9.3, 7.7, 4.2 Hz, 1H), 4.32 (td, *J* = 8.4, 5.7 Hz, 1H), 4.20 (dt, *J* = 7.9, 5.3 Hz, 1H), 3.63 (dd, *J* = 10.7, 5.6 Hz, 1H), 3.57 (dd, *J* = 10.8, 5.1 Hz, 1H), 3.26 – 3.20 (m, 2H), 3.06 (dd, *J* = 14.1, 4.2 Hz, 1H), 2.82 (dd, *J* = 14.1, 9.3 Hz, 1H), 2.48 – 2.44 (m, 1H), 2.32 (dd, *J* = 15.4, 7.4 Hz, 1H), 1.97 (dt, *J* = 13.9, 6.3 Hz, 1H), 1.79 (s, 4H). **HRMS** $C_{30}H_{42}F_3N_7O_{10}$: calculated (M+H)+ m/z 718.3024; found (M+H)+ m/z 718.3031.

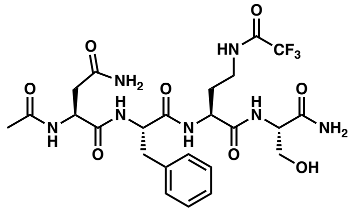

Ac-*L*-Asn-*L*-Phe-*L*-Dab(Tfa)-*L*-Ser-NH_2 (Compound A7). Prepared according to General Procedure A from Rink amide resin (0.3 mmol). Purified via prep HPLC C18 reverse phase column (30% --> 60% $MeCN/H_2O$ w/ 0.1% TFA). White solid (0.0868 g, 48% isolated yield). **^{1}H NMR** (500 MHz, DMSO) δ 9.36 (t, *J* = 5.6 Hz, 1H), 8.19 (d, *J* = 7.8 Hz, 1H), 8.02 (t, *J* = 7.5 Hz, 2H), 7.81 (d, *J* = 7.8 Hz, 1H), 7.36 (s, 1H), 7.26 – 7.16 (m, 6H), 7.11 (s, 1H), 6.90 (s, 1H), 4.90 (s, 1H), 4.51 (q, *J* = 7.2 Hz, 1H), 4.42 (ddd, *J* = 9.3, 7.7, 4.2 Hz, 1H), 4.32 (td, *J* = 8.4, 5.7 Hz, 1H), 4.20 (dt, *J* = 7.9, 5.3 Hz, 1H), 3.63 (dd, *J* = 10.7, 5.6 Hz, 1H), 3.57 (dd, *J* = 10.8, 5.1 Hz, 1H), 3.26 – 3.20 (m, 2H), 3.06 (dd, *J* = 14.1, 4.2 Hz, 1H), 2.82 (dd, *J* = 14.1, 9.3 Hz, 1H), 2.48 – 2.44 (m, 1H), 2.32 (dd, *J* = 15.4, 7.4 Hz, 1H), 1.97 (dt, *J* = 13.9, 6.3 Hz, 1H), 1.79 (s, 4H). **HRMS** $C_{24}H_{32}F_3N_7O_8$: calculated (M+H)+ m/z 604.2343; found (M+H)+ m/z 604.1962.

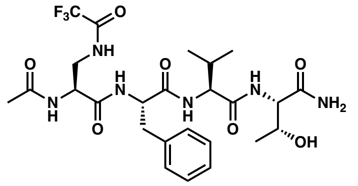

Ac-*L*-Dap(Tfa)-*L*-Phe-*L*-Val-*L*-Thr-NH_2 (Compound A8). Prepared according to General Procedure A from Rink amide resin (0.3 mmol). Purified via prep HPLC C18 reverse phase column (30% --> 60% $MeCN/H_2O$ w/ 0.1% TFA). White solid (0.1111 g, 63% isolated yield). **^{1}H NMR** (600 MHz, DMSO) δ 9.27 (t, *J* = 5.8 Hz, 1H), 8.21 (d, *J* = 8.7 Hz, 1H), 8.02 (dd, *J* = 25.0, 8.1 Hz, 2H), 7.67 (d, *J* = 8.6 Hz, 1H), 7.27 – 7.23 (m, 4H), 7.21 – 7.16 (m, 1H), 7.08 (d, *J* = 14.1 Hz, 2H), 4.90 (d, *J* = 5.2 Hz, 1H), 4.62 (ddd, *J* = 9.6, 7.8, 4.1 Hz, 1H), 4.47 (ddd, *J* = 8.4, 7.5, 6.3 Hz, 1H), 4.24 (dd, *J* = 8.7, 6.9 Hz, 1H), 4.15 (dd, *J* = 8.7, 3.5 Hz, 1H), 4.02 (dtd, *J* = 7.1, 5.9, 3.6 Hz, 1H), 3.37 – 3.33 (m, 2H), 3.05 (dd, *J* = 14.1, 4.1 Hz, 1H), 2.80 (dd, *J* = 14.1, 9.6 Hz, 1H), 2.08 – 1.98 (m, 1H), 1.82 (s, 3H), 1.03 (d, *J* = 6.3 Hz, 3H), 0.88 (dd, *J* = 8.5, 6.8 Hz, 6H). **HRMS** $C_{25}H_{35}F_3N_6O_7$: calculated (M+H)+ m/z 589.2598; found (M+H)+ m/z 589.2606.

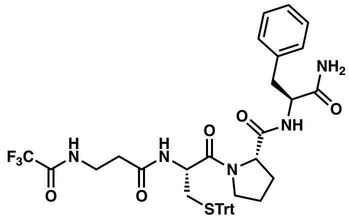

Tfa-β-Ala-*L*-Cys(Trt)-*L*-Pro-*L*-Phe-NH_2 (Compound A9). Prepared according to General Procedure A from Sieber amide resin (0.3 mmol). Purified via prep HPLC C18 reverse phase column (40% --> 70% $MeCN/H_2O$ w/ 0.1% TFA). White solid (0.1275 g, 55% isolated yield). **^{1}H NMR** (500 MHz, DMSO) δ 9.40 (t, *J* = 5.6 Hz, 1H), 8.43 (d, *J* = 8.4 Hz, 1H), 7.58 (d, *J* = 8.3 Hz, 1H), 7.32 (d, *J* = 4.4 Hz, 11H), 7.28 – 7.12 (m, 10H), 7.02 (s, 1H), 4.43 (td, *J* = 8.8, 5.6 Hz, 1H), 4.34 (td, *J* = 8.7, 5.1 Hz, 1H), 4.16 (dd, *J* = 8.7, 3.2 Hz, 1H), 3.35 (q, *J* = 6.8 Hz, 2H), 3.24 (dt, *J* = 9.7, 7.3 Hz, 1H), 2.99 (dd, *J* = 13.7, 5.1 Hz, 2H), 2.69 (dd, *J* = 13.9, 9.1 Hz, 1H), 2.49 – 2.34 (m, 4H), 1.84 (ddt, *J* = 13.3, 10.5, 6.8 Hz, 1H), 1.72 – 1.50 (m, 3H). **HRMS** $C_{41}H_{42}F_3N_5O_5S$: calculated (M+H)+ m/z 774.2937; found (M+H)+ m/z 774.2950.

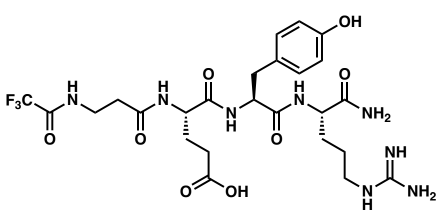

Tfa-β-Ala-*L*-Glu-*L*-Tyr-*L*-Arg-NH2 (Compound A10). Prepared according to General Procedure A from Rink amide resin (0.3 mmol). Purified via prep HPLC C18 reverse phase column (20% --> 40% MeCN/H2O w/ 0.1% TFA). White solid (0.1422 g, 75% isolated yield). **^{1}H NMR** (500 MHz, DMSO) δ 12.07 (s, 1H), 9.38 (t, *J* = 5.8 Hz, 1H), 9.12 (s, 1H), 8.03 (dd, *J* = 11.1, 7.2 Hz, 2H), 7.90 (dd, *J* = 7.9, 4.9 Hz, 2H), 7.75 (d, *J* = 7.9 Hz, 1H), 7.26 (s, 1H), 7.00 (d, *J* = 7.7 Hz, 3H), 6.63 (d, *J* = 8.1 Hz, 2H), 4.33 (dtd, *J* = 22.0, 8.3, 4.8 Hz, 2H), 4.24 – 4.08 (m, 3H), 3.15 (q, *J* = 6.8 Hz, 2H), 2.91 (dd, *J* = 14.1, 4.6 Hz, 1H), 2.72 (dd, *J* = 14.2, 9.1 Hz, 1H), 2.42 (td, *J* = 9.3, 5.8 Hz, 2H), 2.24 (t, *J* = 7.9 Hz, 2H), 2.03 (s, 3H), 1.95 – 1.76 (m, 6H), 1.75 – 1.61 (m, 2H), 1.55 – 1.44 (m, 3H), 1.29 – 1.23 (m, 2H), 1.15 (d, *J* = 7.1 Hz, 3H). **HRMS** $C_{25}H_{35}F_3N_8O_8$: calculated (M+H)+ m/z 633.2608; found (M+H)+ m/z 633.2626.

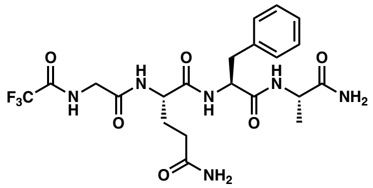

Tfa-Gly-*L*-Gln-*L*-Phe-*L*-Ala-NH2 (Compound A11). Prepared according to General Procedure A from Rink amide resin (0.3 mmol). Purified via prep HPLC C18 reverse phase column (30% --> 60% MeCN/H2O w/ 0.1% TFA). White solid (0.1053 g, 68% isolated yield). **^{1}H NMR** (500 MHz, DMSO) δ 9.59 (t, *J* = 6.0 Hz, 1H), 8.24 (d, *J* = 7.8 Hz, 1H), 8.10 (d, *J* = 8.1 Hz, 1H), 7.92 (d, *J* = 7.5 Hz, 1H), 7.31 – 7.08 (m, 7H), 7.00 (s, 1H), 6.74 (s, 1H), 4.47 (td, *J* = 8.8, 4.6 Hz, 1H), 4.20 (dq, *J* = 25.1, 7.3 Hz, 2H), 3.90 – 3.79 (m, 2H), 3.05 (dd, *J* = 14.0, 4.7 Hz, 1H), 2.80 (dd, *J* = 13.9, 9.5 Hz, 1H), 2.11 – 1.96 (m, 2H), 1.79 (qd, *J* = 15.1, 14.3, 7.5 Hz, 1H), 1.71 – 1.62 (m, 1H), 1.21 (d, *J* = 7.1 Hz, 3H). **HRMS** $C_{21}H_{27}F_3N_6O_6$: calculated (M+H)+ m/z 517.2022; found (M+H)+ m/z 517.2026.

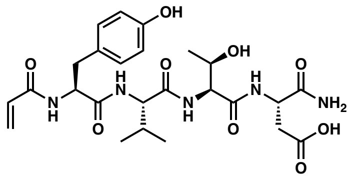

acryloyl-*L*-Tyr-*L*-Val-*L*-Thr-*L*-Asp-NH2 (Compound A12). Prepared according to General Procedure A from Rink amide resin (0.3 mmol). Purified via prep HPLC C18 reverse phase column (20% --> 40% MeCN/H2O w/ 0.1% TFA). White solid (0.1350 g, 82% isolated yield). **^{1}H NMR** (500 MHz, DMSO) δ 12.29 (s, 1H), 9.12 (s, 1H), 8.25 (d, *J* = 8.5 Hz, 1H), 8.04 (dd, *J* = 8.4, 5.1 Hz, 2H), 7.78 (d, *J* = 8.1 Hz, 1H), 7.16 (d, *J* = 19.4 Hz, 2H), 7.04 (d, *J* = 8.2 Hz, 2H), 6.61 (d, *J* = 8.2 Hz, 2H), 6.25 (dd, *J* = 17.1, 10.2 Hz, 1H), 6.00 (dd, *J* = 17.1, 2.1 Hz, 1H), 5.54 (dd, *J* = 10.2, 2.2 Hz, 1H), 5.06 (d, *J* = 5.0 Hz, 1H), 4.61 (ddd, *J* = 10.2, 8.4, 3.9 Hz, 1H), 4.49 (q, *J* = 7.2 Hz, 1H), 4.31 – 4.20 (m, 2H), 4.06 – 3.95 (m, 1H), 2.92 (dd, *J* = 14.1, 3.9 Hz, 1H), 2.72 – 2.53 (m, 3H), 2.01 (p, *J* = 6.8 Hz, 1H), 1.04 (d, *J* = 6.3 Hz, 3H), 0.86 (t, *J* = 7.0 Hz, 6H). **HRMS** $C_{25}H_{35}N_5O_9$: calculated (M+H)+ m/z 550.2513; found (M+H)+ m/z 550.2515.

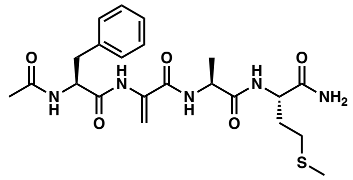

Ac-*L*-Phe-Dha-*L*-Ala-*L*-Met-NH2 (Compound A13). Prepared according to General Procedure B from Rink amide resin (0.3 mmol). Purified via prep HPLC C18 reverse phase column (30% --> 60% MeCN/H2O w/ 0.1% TFA). White solid (0.0644 g, 45% isolated yield). **^{1}H NMR** (500 MHz, DMSO) δ 9.30 (s, 1H), 8.40 (d, *J* = 7.0 Hz, 1H), 8.30 (d, *J* = 7.9 Hz, 1H), 7.90 (d, *J* = 8.2 Hz, 1H), 7.29 – 7.14 (m, 6H), 7.05 (s, 1H), 6.03 (s, 1H), 5.59 (s, 1H), 4.56 (ddd, *J* = 10.3, 7.8, 4.6 Hz, 1H), 4.31 (q, *J* = 7.1 Hz, 1H), 4.24 (td, *J* = 8.6, 4.6 Hz, 1H), 3.07 (dd, *J* = 13.9, 4.5 Hz, 1H), 2.81 – 2.71 (m, 1H), 2.42 (ddd, *J* = 22.8, 11.6, 5.7 Hz, 2H), 2.03 (s, 3H), 1.94 (ddd, *J* = 17.3, 10.9, 6.6 Hz, 1H), 1.84 – 1.74 (m, 4H), 1.29 (d, *J* = 7.1 Hz, 3H). **HRMS** $C_{22}H_{31}N_5O_5S$: calculated (M+H)+ m/z 478.2124; found (M+H)+ m/z 478.2118.

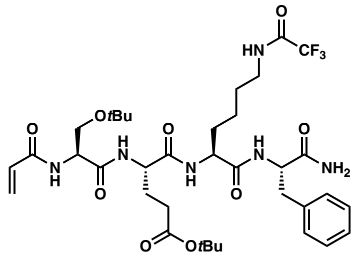

acryloyl - *L* - Ser(*t*Bu) - *L* - Glu(O*t*Bu) - *L* - Lys(Tfa) - *L* - Phe - NH_2 (Compound A14). Prepared according to General Procedure A from Sieber amide resin (0.3 mmol). Purified via prep HPLC C18 reverse phase column (50% --> 80% MeCN/H_2O w/ 0.1% TFA). White solid (0.1132 g, 49% isolated yield). **^{1}H NMR** (600 MHz, DMSO) δ 9.37 (t, *J* = 5.7 Hz, 1H), 8.22 (d, *J* = 7.7 Hz, 1H), 7.93 – 7.82 (m, 3H), 7.30 (d, *J* = 2.1 Hz, 1H), 7.28 – 7.15 (m, 5H), 7.04 (d, *J* = 2.1 Hz, 1H), 6.43 (dd, *J* = 17.1, 10.2 Hz, 1H), 6.11 (dd, *J* = 17.1, 2.1 Hz, 1H), 5.62 (dd, *J* = 10.2, 2.1 Hz, 1H), 4.42 (dq, *J* = 7.5, 5.0 Hz, 2H), 4.31 (td, *J* = 8.4, 4.9 Hz, 1H), 4.15 (td, *J* = 8.2, 5.4 Hz, 1H), 3.54 (dd, *J* = 9.2, 5.3 Hz, 1H), 3.46 (s, 1H), 3.11 (q, *J* = 7.2 Hz, 2H), 3.01 (dd, *J* = 13.9, 5.2 Hz, 1H), 2.82 (dd, *J* = 13.8, 8.7 Hz, 1H), 2.21 (qdd, *J* = 16.5, 10.7, 5.6 Hz, 2H), 1.89 (ddt, *J* = 13.6, 10.8, 5.5 Hz, 1H), 1.72 (dddd, *J* = 13.9, 10.7, 8.8, 5.4 Hz, 1H), 1.57 (ddt, *J* = 13.4, 11.0, 5.8 Hz, 1H), 1.50 – 1.40 (m, 3H), 1.38 (s, 9H), 1.23 – 1.14 (m, 2H), 1.11 (s, 9H). **HRMS** $C_{36}H_{53}F_3N_6O_9$: calculated (M+H)+ m/z 771.3904; found (M+H)+ m/z 771.3920.

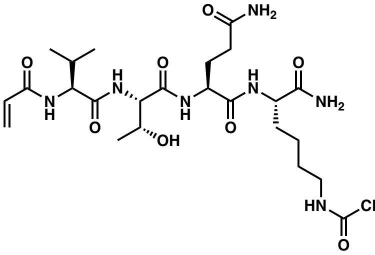

acryloyl-*L*-Val-*L*-Thr-*L*-Gln-*L*-Lys(Tfa)-NH_2 (Compound A15). Prepared according to General Procedure A from Rink amide resin (0.3 mmol). Purified via prep HPLC C18 reverse phase column (20% --> 40% MeCN/H_2O w/ 0.1% TFA). White solid (0.1121 g, 66% isolated yield). **^{1}H NMR** (500 MHz, DMSO) δ 9.39 (t, *J* = 5.5 Hz, 1H), 8.19 (d, *J* = 8.6 Hz, 1H), 7.92 – 7.75 (m, 3H), 7.22 (d, *J* = 18.9 Hz, 2H), 7.07 – 6.98 (m, 1H), 6.79 – 6.69 (m, 1H), 6.43 (dd, *J* = 17.1, 10.2 Hz, 1H), 6.11 (dd, *J* = 17.1, 2.2 Hz, 1H), 5.60 (dd, *J* = 10.2, 2.2 Hz, 1H), 4.94 (d, *J* = 5.2 Hz, 1H), 4.32 (dd, *J* = 8.6, 6.9 Hz, 1H), 4.23 (ddd, *J* = 17.0, 8.1, 4.8 Hz, 2H), 4.12 (td, *J* = 8.5, 5.0 Hz, 1H), 4.00 (q, *J* = 5.3 Hz, 1H), 3.14 (q, *J* = 6.8 Hz, 2H), 2.10 (t, *J* = 7.7 Hz, 2H), 2.02 (dt, *J* = 13.6, 6.8 Hz, 1H), 1.91 (dq, *J* = 13.9, 7.5, 6.7 Hz, 1H), 1.75 (dq, *J* = 15.2, 8.1 Hz, 1H), 1.69 – 1.61 (m, 1H), 1.47 (tt, *J* = 14.8, 8.3 Hz, 3H), 1.30 (s, 2H), 1.03 (d, *J* = 6.3 Hz, 3H), 0.87 (dd, *J* = 12.5, 6.8 Hz, 6H). **HRMS** $C_{25}H_{40}F_3N_7O_8$: calculated (M+H)+ m/z 624.2969; found (M+H)+ m/z 624.2621.

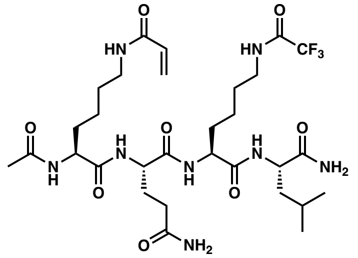

Ac-*L*-Lys(acryloyl)-*L*-Gln-*L*-Lys(Tfa)-*L*-Leu-NH_2 (Compound A16). Prepared according to General Procedure C from Rink amide resin (0.3 mmol). Purified via prep HPLC C18 reverse phase column (30% --> 60% MeCN/H_2O w/ 0.1% TFA). White solid (0.0911 g, 43% isolated yield). **^{1}H NMR** (500 MHz, DMSO) δ 9.39 (t, *J* = 5.7 Hz, 1H), 8.18 (d, *J* = 7.3 Hz, 1H), 8.06 (d, *J* = 7.5 Hz, 2H), 7.85 (d, *J* = 7.6 Hz, 1H), 7.69 (d, *J* = 8.2 Hz, 1H), 7.27 (s, 1H), 7.18 (s, 1H), 6.98 (s, 1H), 6.78 (s, 1H), 6.20 (dd, *J* = 17.1, 10.1 Hz, 1H), 6.05 (dd, *J* = 17.2, 2.3 Hz, 1H), 5.55 (dd, *J* = 10.1, 2.3 Hz, 1H), 4.23 – 4.08 (m, 4H), 3.12 (dq, *J* = 19.8, 6.7 Hz, 4H), 2.10 (h, *J* = 8.4 Hz, 2H), 1.86 (s, 4H), 1.76 (dt, *J* = 14.3, 7.4 Hz, 1H), 1.65 – 1.40 (m, 10H), 1.34 – 1.20 (m, 5H), 0.84 (dd, *J* = 26.9, 6.5 Hz, 6H). **HRMS** $C_{30}H_{49}F_3N_8O_8$: calculated (M+H)+ m/z 707.3704; found (M+H)+ m/z 707.3719.

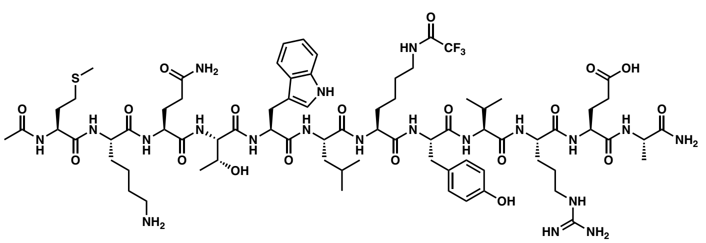

Ac - *L* - Met - *L* - Lys - *L* - Gln - *L* - Thr - *L* - Trp - *L* - Leu - *L* - Lys(Tfa) - *L* - Tyr - *L* - Val - *L* - Arg - *L* - Glu - *L* - Ala - NH_2 (Compound A17). Prepared according to General Procedure A from TentaGel S RAM amide resin (0.3 mmol). Purified 0.05 mmol of resin via prep HPLC C18 reverse phase column (30% --> 60% MeCN/H_2O w/ 0.1% TFA). White solid (0.0675 g, 80% isolated yield). Analytical HPLC trace obtained using analytical HPLC C18 reverse phase column (0% --> 100% MeCN/H_2O w/ 0.1% TFA). **HRMS** $C_{75}H_{115}F_3N_{20}O_{19}S$: calculated (M+H)+ m/z 1689.8398; found (M+H)+ m/z 1689.8391.

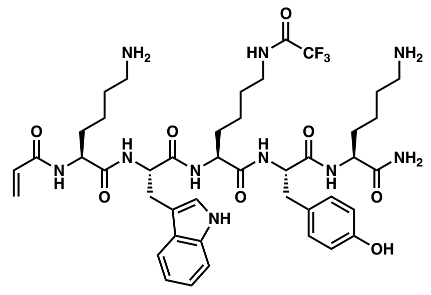

acryloyl-*L*-Lys-*L*-Trp-*L*-Lys(Tfa)-*L*-Tyr-*L*-Lys-NH_2 (Compound A18). Prepared according to General Procedure A from TentaGel S RAM amide resin (0.3 mmol). Purified 0.05 mmol of resin via prep HPLC C18 reverse phase column (25% --> 55% MeCN/H_2O w/ 0.1% TFA). White solid (0.0369 g, 82% isolated yield). **^{1}H NMR** (500 MHz, DMSO) δ 10.76 (d, *J* = 2.5 Hz, 1H), 9.40 (t, *J* = 5.8 Hz, 1H), 9.16 (s, 1H), 8.25 (d, *J* = 7.7 Hz, 1H), 8.01 – 7.77 (m, 4H), 7.65 (q, *J* = 10.4, 5.6 Hz, 4H), 7.56 (d, *J* = 7.9 Hz, 1H), 7.30 (d, *J* = 8.1 Hz, 1H), 7.19 (s, 1H), 7.12 – 6.98 (m, 5H), 6.93 (t, *J* = 7.4 Hz, 1H), 6.63 (d, *J* = 8.1 Hz, 2H), 6.31 (dd, *J* = 17.1, 10.2 Hz, 1H), 6.08 (dd, *J* = 17.1, 2.2 Hz, 1H), 5.61 (dd, *J* = 10.1, 2.2 Hz, 1H), 4.53 (td, *J* = 8.2, 4.3 Hz, 1H), 4.42 (td, *J* = 8.2, 5.0 Hz, 1H), 4.26 (td, *J* = 8.4, 5.1 Hz, 1H), 4.22 – 4.09 (m, 2H), 3.16 – 3.05 (m, 3H), 2.95 (ddd, *J* = 19.4, 14.5, 6.9 Hz, 2H), 2.73 (dtd, *J* = 24.6, 13.8, 13.3, 6.2 Hz, 5H), 1.69 – 1.39 (m, 12H), 1.24 (ddt, *J* = 26.8, 15.6, 7.7 Hz, 6H). **HRMS** $C_{43}H_{59}F_3N_{10}O_8$: calculated (M+H)+ m/z 901.4548; found (M+H)+ m/z 901.4568.

6. Experimental methods 1: Product synthesis and characterization

General Procedure D – *Photoredox-catalyzed C(sp³)-H activation and intermolecular alkylation or intramolecular macrocyclization of trifluoroacetamide-protected peptides*

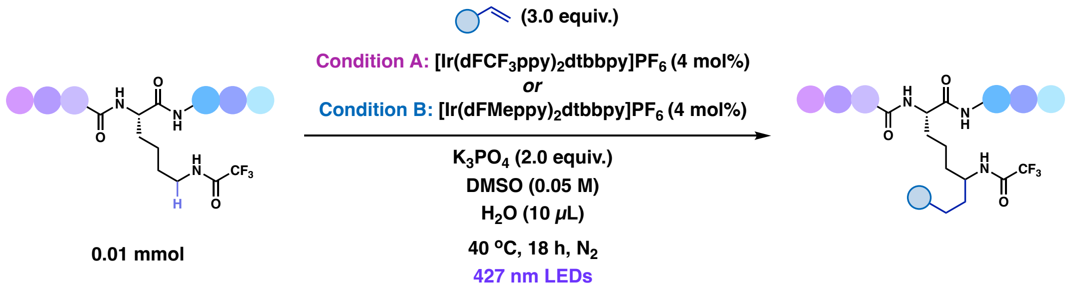

General Procedure D1: This protocol is for intermolecular alkylation for solution phase peptides. To a 1.0-dram vial was added either: $[Ir(dFCF_3ppy)_2dtbbpy]PF_6$ (4 mol%) (**Condition A**) *or* $[Ir(dFMeppy)_2dtbbpy]PF_6$ (4 mol%) (**Condition B**) via a stock solution in DCM, and then the volatiles removed under reduced pressure via rotary evaporator to coat the inside of the flask with catalyst. The vial was then charged with a new and unused micro stir bar. To the vial was added K_3PO_4 (2.0 equiv.) as a solid, followed by addition of peptide starting material (1.0 equiv., 0.01 mmol) as a stock solution in DMSO (0.05 M, 200 μL). Water (10 μL) and alkene electrophile (3.0 equiv.) *or* trifluoroacetamide nucleophile (3.0 equiv.) were added to the reaction mixture. Alkene electrophile is used if the peptide starting material has a Tfa-protected residue *or* trifluoroacetamide nucleophile is used if the peptide starting material has an electrophilic alkene functionality (i.e. acrylamide or Dha residue). The vial was then sealed with an air-tight PTFE green cap and the reaction mixture was briefly sonicated. The reaction mixture was then sparged with inert nitrogen gas using an inlet needle and an outlet needle for 1 min. The reaction vial was then placed inside of a lightbox containing a magnetic stir plate, aluminum foil insulation, an electric fan attached to cool down the box, and two 427 nm Kessil LED lamps that are each situated approximately 3 cm away from either side of the vial. The bottom of each individual vial was taped to the stir plate using a small piece of double-sided tape to prevent the fan from blowing it over on its side. The reaction vial was then irradiated with both lamps on maximum intensity setting, with both the stirring and the fan on for 18 h. For analysis of crude mixture by ^1H-NMR, a solvent mixture of DMSO-d_6 and D_2O is used instead, such that the mixture is diluted with more DMSO-d_6 after the reaction is finished and directly added to an NMR tube. For MALDI analysis, 1 μL of crude reaction mixture is spotted with 1 μL of matrix solution on a MALDI plate and then dried and directly analyzed on the instrument. The crude reaction mixture was then dissolved in MeCN:H_2O and purified via preparative HPLC reverse phase chromatography. The purified material was then flash frozen in liquid nitrogen and freeze dried using a lyophilizer instrument.

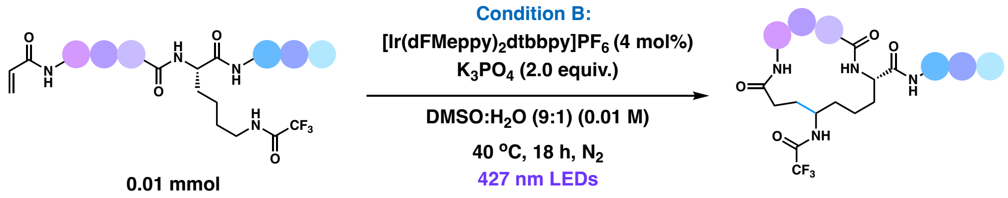

General Procedure D2: This protocol is for intramolecular macrocyclization for solution phase peptides. To a 1.0-dram vial was added $[Ir(dFMeppy)_2dtbbpy]PF_6$ (4 mol%) (**Condition B**) via a stock solution in DCM, and then the volatiles removed under reduced pressure via rotary evaporator to coat the inside of the flask with catalyst. The vial was then charged with a new and unused micro stir bar. To the vial was added K_3PO_4 (2.0 equiv.) as a solid, followed by addition of peptide starting material (1.0 equiv., 0.01 mmol) as a stock solution in DMSO (900 μL). Water (100 μL) was added to the reaction mixture to make the cosolvent mixture concentration as 0.01 M. The vial was then sealed with an air-tight PTFE green cap and the reaction mixture was briefly sonicated. The reaction mixture was then sparged with inert nitrogen gas

using an inlet needle and an outlet needle for 1 min. The reaction vial was then placed inside of a lightbox containing a magnetic stir plate, aluminum foil insulation, an electric fan attached to cool down the box, and two 427 nm Kessil LED lamps that are each situated approximately 3 cm away from either side of the vial. The bottom of each individual vial was taped to the stir plate using a small piece of double-sided tape to prevent the fan from blowing it over on its side. The reaction vial was then irradiated with both lamps on maximum intensity setting, with both the stirring and the fan on for 18 h. For analysis of crude mixture by ^1H-NMR, a solvent mixture of DMSO-d_6 and D_2O is used instead. After the reaction is finished, this mixture is directly added to an NMR tube. For MALDI analysis, 1 μL of crude reaction mixture is spotted with 1 μL of matrix solution on a MALDI plate and then dried and directly analyzed on the instrument. The crude reaction mixture was then dissolved in MeCN:H_2O and purified via preparative HPLC reverse phase chromatography. The purified material was then flash frozen in liquid nitrogen and freeze dried using a lyophilizer instrument.

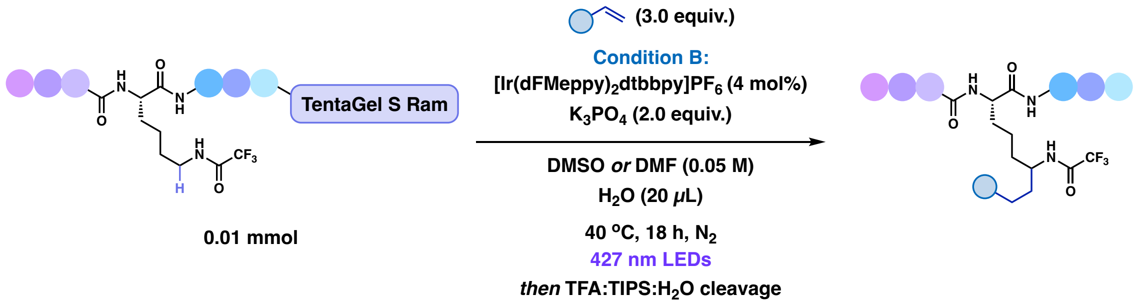

General Procedure D3: This protocol is for intermolecular alkylation for solid phase peptides that are attached on-resin. To a 1.0-dram vial was added [Ir(dFMeppy)$_2$dtbbpy]PF_6 (4 mol%) (**Condition B**) via a stock solution in DCM, and then the volatiles removed under reduced pressure via rotary evaporator to coat the inside of the flask with catalyst. The vial was then charged with a new and unused micro stir bar. To the vial was added K_3PO_4 (2.0 equiv.) as a solid, followed by addition of vacuum-dried TentaGel S RAM resin that is appended with fully protected peptide sequence (1.0 equiv., 0.01 mmol). DMSO *or* DMF (0.05 M, 200 μL), water (20 μL), and alkene electrophile (3.0 equiv.) were added to the reaction mixture. The vial was then sealed with an air-tight PTFE green cap and the reaction mixture was briefly sonicated. The reaction mixture was then sparged with inert nitrogen gas using an inlet needle and an outlet needle for 1 min. The reaction vial was then placed inside of a lightbox containing a magnetic stir plate, aluminum foil insulation, an electric fan attached to cool down the box, and two 427 nm Kessil LED lamps that are each situated approximately 3 cm away from either side of the vial. The bottom of each individual vial was taped to the stir plate using a small piece of double-sided tape to prevent the fan from blowing it over on its side. The reaction vial was then irradiated with both lamps on maximum intensity setting, with both the stirring and the fan on for 18 h.

After the reaction is finished, the heterogenous reaction mixture is quantitatively solvent transferred from the vial to a 20 mL solid phase peptide synthesis vessel by using multiple solvent washes with DMF. The resin is then flow washed x 2 with DCM, followed by DCM batch wash x 1. The cleavage and global deprotection of the TentaGel S RAM resin was accomplished by utilizing a 95:2.5:2.5 mixture of TFA:TIPS:H_2O. The solution was added to the resin in the vessel and then stirred for 3 h. The solution was directly drained from the vessel into a 20 mL scintillation vial, followed by multiple washes with DCM to ensure quantitative transfer. The volatiles were removed by rotary evaporator until highly concentrated. The crude residue is dissolved in DMSO-d_6 for ^1H-NMR analysis. For MALDI analysis, 1 μL of crude reaction mixture is spotted with 1 μL of matrix solution on a MALDI plate and then dried and directly analyzed on the instrument. The crude reaction mixture was then dissolved in MeCN:H_2O and purified via preparative HPLC reverse phase chromatography. The purified material was then flash frozen in liquid nitrogen and freeze dried using a lyophilizer instrument.

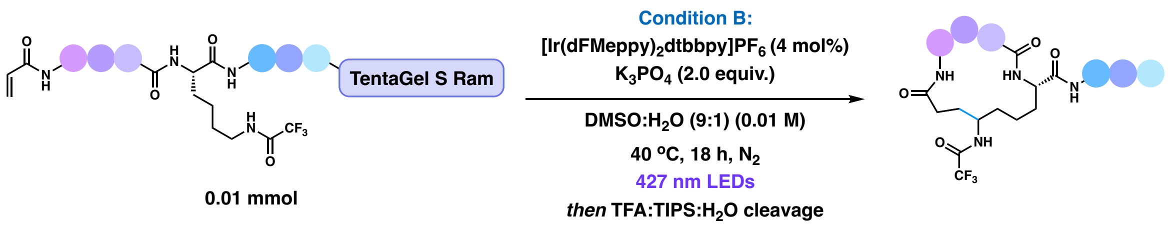

General Procedure D4: This protocol is for intramolecular macrocyclization for solid phase peptides that are attached on-resin. To a 1.0-dram vial was added $[Ir(dFMeppy)_2dtbbpy]PF_6$ (4 mol%) (**Condition B**) via a stock solution in DCM, and then the volatiles removed under reduced pressure via rotary evaporator to coat the inside of the flask with catalyst. The vial was then charged with a new and unused micro stir bar. To the vial was added K_3PO_4 (2.0 equiv.) as a solid, followed by addition of vacuum-dried TentaGel S RAM resin that is appended with fully protected peptide sequence (1.0 equiv., 0.01 mmol). DMSO (900 μL) and water (100 μL) were added to the reaction mixture to make the cosolvent mixture concentration as 0.01 M. The vial was then sealed with an air-tight PTFE green cap and the reaction mixture was briefly sonicated. The reaction mixture was then sparged with inert nitrogen gas using an inlet needle and an outlet needle for 1 min. The reaction vial was then placed inside of a lightbox containing a magnetic stir plate, aluminum foil insulation, an electric fan attached to cool down the box, and two 427 nm Kessil LED lamps that are each situated approximately 3 cm away from either side of the vial. The bottom of each individual vial was taped to the stir plate using a small piece of double-sided tape to prevent the fan from blowing it over on its side. The reaction vial was then irradiated with both lamps on maximum intensity setting, with both the stirring and the fan on for 18 h.

After the reaction is finished, the heterogenous reaction mixture is quantitatively solvent transferred from the vial to a 20 mL solid phase peptide synthesis vessel by using multiple solvent washes with DMF. The resin is then flow washed x 2 with DCM, followed by DCM batch wash x 1. The cleavage and global deprotection of the TentaGel S RAM resin was accomplished by utilizing a 95:2.5:2.5 mixture of $TFA:TIPS:H_2O$. The solution was added to the resin in the vessel and then stirred for 3 h. The solution was directly drained from the vessel into a 20 mL scintillation vial, followed by multiple washes with DCM to ensure quantitative transfer. The volatiles were removed by rotary evaporator until highly concentrated. The crude residue is dissolved in DMSO-d_6 for ^{1}H-NMR analysis. For MALDI analysis, 1 μL of crude reaction mixture is spotted with 1 μL of matrix solution on a MALDI plate and then dried and directly analyzed on the instrument. The crude reaction mixture was then dissolved in $MeCN:H_2O$ and purified via preparative HPLC reverse phase chromatography. The purified material was then flash frozen in liquid nitrogen and freeze dried using a lyophilizer instrument.

General Procedure E – *Basic deprotection of trifluoroacetamide-protected residues and intramolecular imine cyclization*

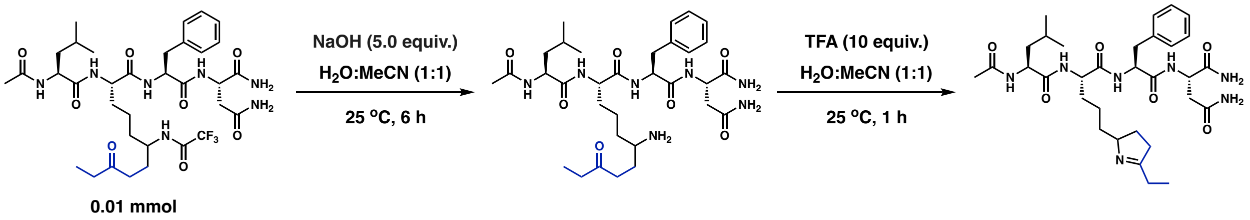

To a 1.0-dram vial was added equipped with stir bar was added Tfa-protected peptide (1.0 equiv., 0.01 mmol) as a stock solution in $MeCN:H_2O$ (1:1, 5 mL). NaOH (5.0 equiv.) was added to the reaction mixture and the solution was stirred at room temperature for 6 h. The reaction was then quenched with TFA (10.0 equiv.) and stirred for 1 h. The crude reaction mixture purified via preparative HPLC reverse phase chromatography. The purified material was then flash frozen in liquid nitrogen and freeze dried using a lyophilizer instrument.

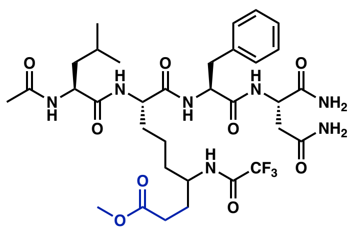

Ac-*L*-Leu-*L*-Lys(Tfa)-*L*-Phe-*L*-Asn-NH2 and methyl acrylate adduct (Compound 1). Prepared according to General Procedure D1, Condition A from Compound A1 (0.01 mmol) and methyl acrylate (0.03 mmol). Purified via prep HPLC C18 reverse phase column (25% --> 55% MeCN/H_2O w/ 0.1% TFA). White solid (0.0053 g, 72% isolated yield). **^{1}H NMR** (500 MHz, DMSO) δ 9.12 (dd, *J* = 11.9, 8.6 Hz, 1H), 8.19 – 7.79 (m, 4H), 7.30 – 7.15 (m, 6H), 7.07 – 6.77 (m, 3H), 4.49 – 4.37 (m, 2H), 4.29 – 4.19 (m, 1H), 4.14 (td, *J* = 9.1, 8.6, 4.3 Hz, 1H), 3.72 (s, 1H), 3.57 (s, 3H), 3.02 (dd, *J* = 14.1, 4.8 Hz, 1H), 2.81 (dd, *J* = 14.0, 9.1 Hz, 1H), 2.49 – 2.37 (m, 2H), 2.27 (t, *J* = 7.6 Hz, 2H), 1.83 (d, *J* = 2.3 Hz, 3H), 1.77 (dt, *J* = 13.1, 5.2 Hz, 1H), 1.67 (dtd, *J* = 14.2, 7.3, 3.5 Hz, 1H), 1.56 (h, *J* = 5.5 Hz, 2H), 1.50 – 1.33 (m, 5H), 1.19 (q, *J* = 6.5, 5.2 Hz, 2H), 0.85 (dd, *J* = 20.9, 6.6 Hz, 6H). **HRMS-ESI** $C_{33}H_{48}F_3N_7O_9$: calculated (M+H)+ m/z 744.3544; found (M+H)+ m/z 744.3552.

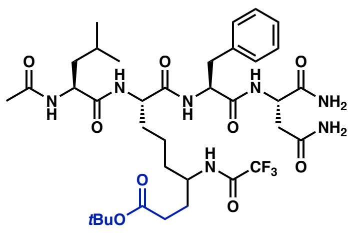

Ac-*L*-Leu-*L*-Lys(Tfa)-*L*-Phe-*L*-Asn-NH2 and *tert*-butyl acrylate adduct (Compound 2). Prepared according to General Procedure D1, Condition A from Compound A1 (0.01 mmol) and *tert*-butyl acrylate (0.03 mmol). Purified via prep HPLC C18 reverse phase column (30% --> 60% MeCN/H_2O w/ 0.1% TFA). White solid (0.0059 g, 75% isolated yield). **^{1}H NMR** (600 MHz, DMSO) δ 9.10 (dd, *J* = 14.5, 8.7 Hz, 1H), 8.06 (t, *J* = 7.1 Hz, 1H), 8.01 – 7.81 (m, 3H), 7.31 – 7.26 (m, 1H), 7.25 – 7.13 (m, 5H), 7.03 (s, 1H), 6.86 (d, *J* = 30.3 Hz, 2H), 4.43 (qd, *J* = 6.9, 3.0 Hz, 2H), 4.30 – 4.19 (m, 1H), 4.14 (qd, *J* = 7.9, 4.7 Hz, 1H), 3.71 (dt, *J* = 9.7, 5.1 Hz, 1H), 3.02 (dd, *J* = 13.9, 4.9 Hz, 1H), 2.80 (dd, *J* = 14.0, 9.0 Hz, 1H), 2.49 – 2.37 (m, 2H), 2.19 – 2.10 (m, 2H), 1.83 (d, *J* = 2.0 Hz, 3H), 1.77 – 1.69 (m, 1H), 1.58 (dh, *J* = 20.3, 7.0, 6.1 Hz, 3H), 1.44 (dd, *J* = 14.6, 6.2 Hz, 2H), 1.38 (s, 12H), 1.24 – 1.17 (m, 2H), 0.84 (dd, *J* = 25.1, 6.6 Hz, 6H). **HRMS-ESI** $C_{36}H_{54}F_3N_7O_9$: calculated (M+H)+ m/z 786.4013; found (M+H)+ m/z 786.4017.

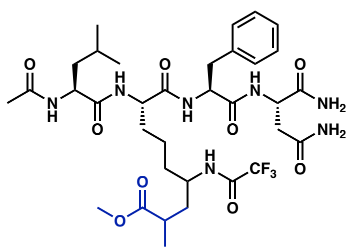

Ac-*L*-Leu-*L*-Lys(Tfa)-*L*-Phe-*L*-Asn-NH2 and methyl methacrylate adduct (Compound 3). Prepared according to General Procedure D1, Condition A from Compound A1 (0.01 mmol) and methyl methacrylate (0.03 mmol). Purified via prep HPLC C18 reverse phase column (25% --> 55% MeCN/H_2O w/ 0.1% TFA). White solid (0.0051 g, 68% isolated yield). **^{1}H NMR** (600 MHz, DMSO) δ 9.18 – 9.04 (m, 1H), 8.08 – 7.82 (m, 4H), 7.29 (s, 1H), 7.24 – 7.20 (m, 4H), 7.18 – 7.15 (m, 1H), 7.02 (s, 1H), 6.89 (s, 1H), 6.84 (s, 1H), 4.42 (q, *J* = 6.9 Hz, 2H), 4.23 (dq, *J* = 9.0, 4.6 Hz, 1H), 4.14 (td, *J* = 8.7, 4.9 Hz, 1H), 3.73 (d, *J* = 64.0 Hz, 1H), 3.57 (d, *J* = 10.0 Hz, 3H), 3.02 (dd, *J* = 14.1, 5.0 Hz, 1H), 2.80 (dd, *J* = 14.1, 9.0 Hz, 1H), 2.37 (ddd, *J* = 42.5, 14.6, 6.7 Hz, 3H), 1.83 (q, *J* = 3.7 Hz, 4H), 1.55 (td, *J* = 17.5, 15.5, 7.9 Hz, 3H), 1.45 – 1.35 (m, 5H), 1.21 – 1.14 (m, 2H), 1.09 – 1.05 (m, 3H), 0.87 – 0.82 (m, 6H). **HRMS-ESI** $C_{34}H_{50}F_3N_7O_9$: calculated (M+H)+ m/z 758.3700; found (M+H)+ m/z 758.3702.

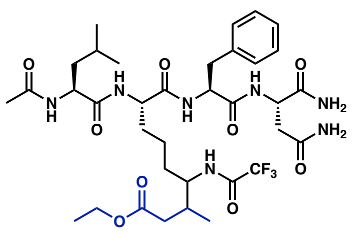

Ac-*L*-Leu-*L*-Lys(Tfa)-*L*-Phe-*L*-Asn-NH2 and ethyl crotonate adduct (Compound 4). Prepared according to General Procedure D1, Condition B from Compound A1 (0.01 mmol) and ethyl crotonate (0.03 mmol). Purified via prep HPLC C18 reverse phase column (25% --> 55% MeCN/H_2O w/ 0.1% TFA). White solid (0.0056 g, 73% isolated yield). **^{1}H NMR** (600 MHz, DMSO) δ 9.09 (ddd, *J* = 35.3, 23.2, 8.9 Hz, 1H), 8.17 – 7.79 (m, 4H), 7.30 (s, 1H), 7.26 – 7.16 (m, 5H), 7.03 (s, 1H), 6.96 – 6.81 (m, 2H), 4.49 – 4.40 (m, 2H), 4.28 – 4.12 (m, 2H), 4.06 (qd, *J* = 6.9, 2.0 Hz, 2H), 3.71 – 3.59 (m, 1H), 3.03 (ddd, *J* = 13.9, 4.9, 2.3 Hz, 1H), 2.85 – 2.76 (m, 1H), 2.45 – 2.32 (m, 2H), 2.04 (tdd, *J* = 9.9, 6.7, 2.9 Hz, 2H), 1.84 (dd, *J* = 5.6, 3.5 Hz, 3H), 1.63 – 1.54 (m, 2H), 1.51 – 1.33 (m, 5H), 1.29 – 1.08 (m, 6H), 0.91 – 0.79 (m, 9H). **HRMS-ESI** $C_{35}H_{52}F_3N_7O_9$: calculated (M+H)+ m/z 772.3857; found (M+H)+ m/z 772.3859.

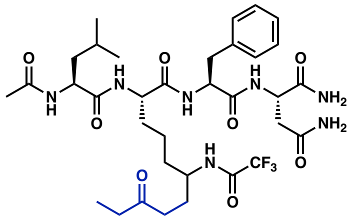

Ac-*L*-Leu-*L*-Lys(Tfa)-*L*-Phe-*L*-Asn-NH2 and ethyl vinyl ketone adduct (Compound 5). Prepared according to General Procedure D1, Condition A from Compound A1 (0.01 mmol) and ethyl vinyl ketone (0.03 mmol). Purified via prep HPLC C18 reverse phase column (25% --> 55% MeCN/H_2O w/ 0.1% TFA). White solid (0.0056 g, 75% isolated yield). **^{1}H NMR** (500 MHz, DMSO) δ 9.08 (dd, *J* = 12.8, 8.7 Hz, 1H), 8.13 – 7.81 (m, 4H), 7.29 (s, 1H), 7.25 – 7.17 (m, 5H), 7.03 (s, 1H), 6.87 (d, *J* = 25.6 Hz, 2H), 4.43 (h, *J* = 6.4 Hz, 2H), 4.23 (td, *J* = 8.8, 4.5 Hz, 1H), 4.18 – 4.06 (m, 1H), 3.72 – 3.58 (m, 1H), 3.02 (dd, *J* = 14.0, 4.8 Hz, 1H), 2.80 (dd, *J* = 13.9, 9.1 Hz, 1H), 2.47 – 2.31 (m, 6H), 1.83 (d, *J* = 2.6 Hz, 3H), 1.75 – 1.68 (m, 1H), 1.60 – 1.52 (m, 3H), 1.46 – 1.34 (m, 5H), 1.18 (q, *J* = 7.9 Hz, 2H), 0.89 (t, *J* = 7.2 Hz, 3H), 0.84 (dd, *J* = 20.9, 6.6 Hz, 6H). **HRMS-ESI** $C_{34}H_{50}F_3N_7O_8$: calculated (M+H)+ m/z 742.3751; found (M+H)+ m/z 742.3758.

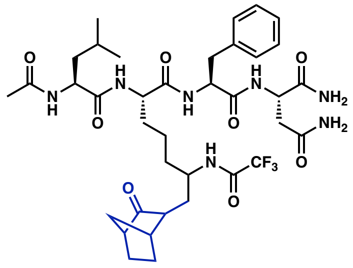

Ac-*L*-Leu-*L*-Lys(Tfa)-*L*-Phe-*L*-Asn-NH2 and 3-methylene-2-norbornanone adduct (Compound 6). Prepared according to General Procedure D1, Condition B from Compound A1 (0.01 mmol) and 3-methylene-2-norbornanone (0.03 mmol). Purified via prep HPLC C18 reverse phase column (30% --> 60% MeCN/H_2O w/ 0.1% TFA). White solid (0.0053 g, 68% isolated yield). **^{1}H NMR** (600 MHz, DMSO) δ 9.21 – 9.06 (m, 1H), 8.11 – 7.81 (m, 4H), 7.28 (s, 1H), 7.26 – 7.13 (m, 5H), 7.02 (s, 1H), 6.86 (d, *J* = 28.8 Hz, 2H), 4.43 (qdd, *J* = 7.6, 6.0, 5.6, 2.3 Hz, 2H), 4.22 (tq, *J* = 9.3, 4.8 Hz, 1H), 4.18 – 4.09 (m, 1H), 3.83 – 3.64 (m, 1H), 3.02 (dd, *J* = 14.0, 4.8 Hz, 1H), 2.80 (dd, *J* = 14.0, 9.0 Hz, 1H), 2.41 (dd, *J* = 15.5, 6.9 Hz, 2H), 2.06 – 1.92 (m, 1H), 1.85 – 1.81 (m, 3H), 1.80 – 1.71 (m, 2H), 1.70 – 1.61 (m, 2H), 1.60 – 1.32 (m, 13H), 1.22 – 1.11 (m, 2H), 0.84 (dd, *J* = 25.1, 6.6 Hz, 6H). **HRMS-ESI** $C_{37}H_{52}F_3N_7O_8$: calculated (M+H)+ m/z 780.3908; found (M+H)+ m/z 780.3906.

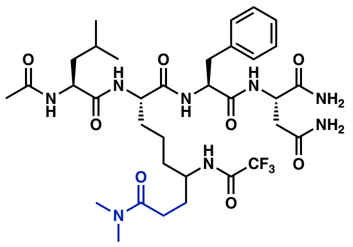

Ac-*L*-Leu-*L*-Lys(Tfa)-*L*-Phe-*L*-Asn-NH2 and *N,N*-dimethylacrylamide adduct (Compound 7). Prepared according to General Procedure D1, Condition A from Compound A1 (0.01 mmol) and *N,N*-dimethylacrylamide (0.03 mmol). Purified via prep HPLC C18 reverse phase column (25% --> 55% MeCN/H_2O w/ 0.1% TFA). White solid (0.0049 g, 65% isolated yield). **^{1}H NMR** (500 MHz, DMSO) δ 9.12 (t, *J* = 8.3 Hz, 1H), 8.24 – 7.73 (m, 4H), 7.32 – 7.13 (m, 6H), 7.04 (s, 1H), 6.88 (d, *J* = 22.8 Hz, 2H), 4.43 (p, *J* = 7.1 Hz, 2H), 4.24 (d, *J* = 8.3 Hz, 1H), 4.14 (td, *J* = 8.8, 4.6 Hz, 1H), 3.78 – 3.63 (m, 1H), 3.02 (dd, *J* = 14.0, 4.9 Hz, 1H), 2.92 (d, *J* = 1.4 Hz, 3H), 2.82 – 2.77 (m, 4H), 2.49 – 2.38 (m, 2H), 2.31 – 2.15 (m, 2H), 1.99 (dt, *J* = 16.0, 7.0 Hz, 1H), 1.87 – 1.80 (m, 3H), 1.73 (s, 1H), 1.61 (dt, *J* = 35.3, 6.8 Hz, 3H), 1.40 (dq, *J* = 20.3, 6.5 Hz, 4H), 1.19 (t, *J* = 8.2 Hz, 2H), 0.85 (dd, *J* = 20.8, 6.6 Hz, 6H). **HRMS-ESI** $C_{34}H_{51}F_3N_8O_8$: calculated (M+H)+ m/z 757.3860; found (M+H)+ m/z 757.3862.

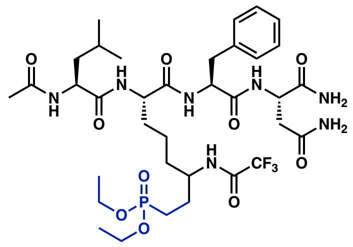

Ac-*L*-Leu-*L*-Lys(Tfa)-*L*-Phe-*L*-Asn-NH2 and diethyl vinylphosphonate adduct (Compound 8). Prepared according to General Procedure D1, Condition B from Compound A1 (0.01 mmol) and diethyl vinylphosphonate (0.03 mmol). Purified via prep HPLC C18 reverse phase column (30% --> 60% MeCN/H_2O w/ 0.1% TFA). White solid (0.0059 g, 72% isolated yield). **^{1}H NMR** (500 MHz, DMSO) δ 9.17 (dd, *J* = 13.6, 8.6 Hz, 1H), 8.19 – 7.69 (m, 4H), 7.32 – 7.28 (m, 1H), 7.24 – 7.15 (m, 5H), 7.04 (d, *J* = 8.7 Hz, 1H), 6.92 – 6.81 (m, 2H), 4.43 (h, *J* = 5.9 Hz, 2H), 4.27 – 4.20 (m, 1H), 4.15 (dq, *J* = 8.6, 4.7 Hz, 1H), 3.97 (ddt, *J* = 14.3, 7.1, 3.3 Hz, 4H), 3.73 (t, *J* = 10.0 Hz, 1H), 3.02 (dd, *J* = 14.0, 4.7 Hz, 1H), 2.85 – 2.75 (m, 1H), 2.47 – 2.35 (m, 2H), 1.83 (s, 3H), 1.71 – 1.52 (m, 6H), 1.50 – 1.34 (m, 5H), 1.22 (t, *J* = 7.1 Hz, 8H), 0.84 (dd, *J* = 21.2, 6.5 Hz, 6H). **HRMS-ESI** $C_{35}H_{55}F_3N_7O_{10}P$: calculated (M+H)+ m/z 822.3778; found (M+H)+ m/z 822.3769.

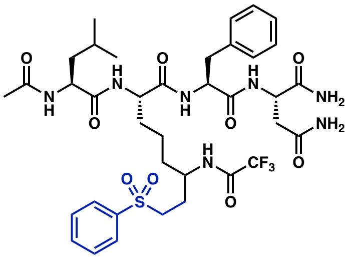

Ac-*L*-Leu-*L*-Lys(Tfa)-*L*-Phe-*L*-Asn-NH₂ and phenyl vinyl sulfone adduct (Compound 9). Prepared according to General Procedure D1, Condition B from Compound A1 (0.01 mmol) and phenyl vinyl sulfone (0.03 mmol). Purified via prep HPLC C18 reverse phase column (30% --> 60% MeCN/H_2O w/ 0.1% TFA). White solid (0.0048 g, 58% isolated yield). **^{1}H NMR** (500 MHz, DMSO) δ 9.16 (dd, *J* = 14.2, 8.5 Hz, 1H), 8.07 (dd, *J* = 8.0, 4.8 Hz, 1H), 7.96 (dd, *J* = 13.5, 7.8 Hz, 1H), 7.92 – 7.84 (m, 4H), 7.79 – 7.73 (m, 1H), 7.67 (t, *J* = 7.7 Hz, 2H), 7.29 (s, 1H), 7.24 – 7.12 (m, 5H), 7.03 (s, 1H), 6.87 (d, *J* = 25.6 Hz, 2H), 4.42 (q, *J* = 6.7 Hz, 2H), 4.22 (t, *J* = 8.5 Hz, 1H), 4.12 (dd, *J* = 10.8, 6.1 Hz, 1H), 3.77 (s, 1H), 3.20 (d, *J* = 5.9 Hz, 2H), 3.02 (dd, *J* = 14.3, 4.9 Hz, 1H), 2.80 (dd, *J* = 14.0, 9.0 Hz, 1H), 2.41 (dd, *J* = 15.5, 6.9 Hz, 2H), 1.82 (d, *J* = 1.5 Hz, 3H), 1.75 (dd, *J* = 10.5, 4.9 Hz, 2H), 1.56 (d, *J* = 7.1 Hz, 2H), 1.45 – 1.30 (m, 5H), 1.14 (d, *J* = 8.8 Hz, 2H), 0.84 (dd, *J* = 21.5, 6.6 Hz, 6H). **HRMS-ESI** $C_{37}H_{50}F_3N_7O_9S$: calculated (M+H)+ m/z 826.3421; found (M+H)+ m/z 826.3420.

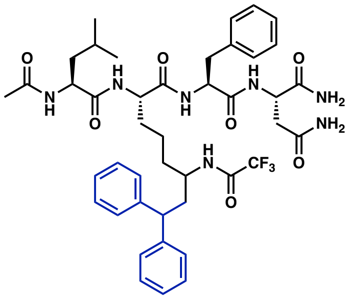

Ac-*L*-Leu-*L*-Lys(Tfa)-*L*-Phe-*L*-Asn-NH₂ and 1,1-diphenylethylene adduct (Compound 10). Prepared according to General Procedure D1, Condition B from Compound A1 (0.01 mmol) and 1,1-diphenylethylene (0.03 mmol). Purified via prep HPLC C18 reverse phase column (50% --> 80% MeCN/H_2O w/ 0.1% TFA). White solid (0.0060 g, 71% isolated yield). **^{1}H NMR** (600 MHz, DMSO) δ 9.17 (t, *J* = 8.8 Hz, 1H), 8.14 – 7.79 (m, 4H), 7.31 – 7.23 (m, 10H), 7.21 – 7.15 (m, 6H), 7.03 (d, *J* = 8.9 Hz, 1H), 6.92 – 6.82 (m, 2H), 4.48 – 4.38 (m, 2H), 4.23 (dtd, *J* = 9.8, 7.9, 5.3 Hz, 1H), 4.11 (dtd, *J* = 10.0, 7.4, 4.7 Hz, 1H), 3.94 – 3.86 (m, 1H), 3.54 – 3.48 (m, 1H), 3.03 – 2.99 (m, 1H), 2.83 – 2.76 (m, 1H), 2.47 – 2.28 (m, 3H), 2.17 (ddt, *J* = 13.7, 7.7, 2.4 Hz, 1H), 1.84 – 1.80 (m, 3H), 1.60 – 1.46 (m, 3H), 1.44 – 1.33 (m, 4H), 1.23 – 1.06 (m, 2H), 0.87 – 0.79 (m, 6H). **HRMS-ESI** $C_{43}H_{54}F_3N_7O_7$: calculated (M+H)+ m/z 838.4115; found (M+H)+ m/z 838.4115.

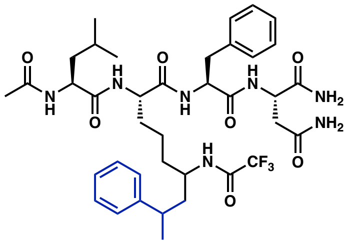

Ac-*L*-Leu-*L*-Lys(Tfa)-*L*-Phe-*L*-Asn-NH₂ and α-methylstyrene adduct (Compound 11). Prepared according to General Procedure D1, Condition B from Compound A1 (0.01 mmol) and α-methylstyrene (0.03 mmol). Purified via prep HPLC C18 reverse phase column (40% --> 70% MeCN/H_2O w/ 0.1% TFA). White solid (0.0043 g, 56% isolated yield). **^{1}H NMR** (500 MHz, DMSO) δ 9.11 (q, *J* = 10.5, 8.9 Hz, 1H), 8.15 – 7.79 (m, 4H), 7.29 – 7.16 (m, 11H), 7.03 (s, 1H), 6.87 (d, *J* = 23.5 Hz, 2H), 4.49 – 4.34 (m, 2H), 4.24 (d, *J* = 12.6 Hz, 1H), 4.17 – 4.02 (m, 1H), 3.75 (s, 1H), 3.06 – 2.94 (m, 1H), 2.80 (ddd, *J* = 14.2, 8.9, 5.5 Hz, 1H), 2.66 – 2.61 (m, 1H), 2.45 – 2.35 (m, 2H), 1.85 – 1.80 (m, 3H), 1.79 – 1.67 (m, 2H), 1.56 (d, *J* = 6.7 Hz, 2H), 1.46 – 1.33 (m, 5H), 1.17 (dd, *J* = 6.9, 3.2 Hz, 5H), 0.87 – 0.81 (m, 6H). **HRMS-ESI** $C_{38}H_{52}F_3N_7O_7$: calculated (M+H)+ m/z 776.3959; found (M+H)+ m/z 776.3973.

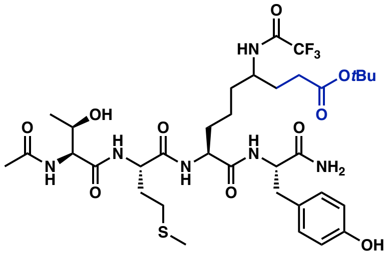

Ac-*L*-Thr-*L*-Met-*L*-Lys(Tfa)-*L*-Tyr-NH₂ and *tert*-butyl acrylate adduct (Compound 12). Prepared according to General Procedure D1, Condition B from Compound A2 (0.01 mmol) and *tert*-butyl acrylate (0.03 mmol). Purified via prep HPLC C18 reverse phase column (30% --> 60% MeCN/H_2O w/ 0.1% TFA). White solid (0.0063 g, 78% isolated yield). **^{1}H NMR** (500 MHz, DMSO) δ 9.16 – 9.02 (m, 2H), 8.07 – 7.58 (m, 4H), 7.25 (s, 1H), 6.98 (dt, *J* = 8.6, 2.9 Hz, 3H), 6.66 – 6.57 (m, 2H), 4.87 (s, 1H), 4.30 (td, *J* = 7.9, 4.4 Hz, 2H), 4.23 – 4.05 (m, 2H), 3.96 (s, 1H), 3.72 (s, 1H), 2.84 (dd, *J* = 13.7, 5.5 Hz, 1H), 2.71 (dd, *J* = 13.9, 8.0 Hz, 1H), 2.46 – 2.37 (m, 2H), 2.20 – 2.10 (m, 1H), 2.07 (s, 5H), 2.01 (s, 3H), 1.89 – 1.69 (m, 4H), 1.59 (dd, *J* = 24.3, 9.3 Hz, 2H), 1.38 (s, 12H), 1.11 – 0.98 (m, 3H). **HRMS-ESI** $C_{35}H_{53}F_3N_6O_{10}S$: calculated (M+H)+ m/z 807.3574; found (M+H)+ m/z 807.3558.

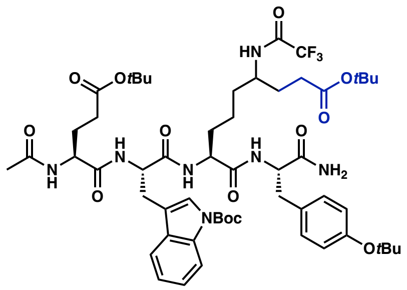

Ac - *L* - Glu(O*t*Bu) - *L* - Trp(Boc) - *L* - Lys(Tfa) - *L* - Tyr(*t*Bu) - NH_2 and *tert*-butyl acrylate adduct (Compound 13). Prepared according to General Procedure D1, Condition B from Compound A3 (0.01 mmol) and *tert*-butyl acrylate (0.03 mmol). Purified via prep HPLC C18 reverse phase column (50% --> 80% $MeCN/H_2O$ w/ 0.1% TFA). White solid (0.0069 g, 63% isolated yield). **^{1}H NMR** (600 MHz, DMSO) δ 9.07 (d, *J* = 8.6 Hz, 1H), 8.06 – 7.94 (m, 4H), 7.73 – 7.63 (m, 2H), 7.51 (d, *J* = 5.3 Hz, 1H), 7.29 – 7.19 (m, 3H), 7.12 – 7.06 (m, 3H), 6.80 (dd, *J* = 8.3, 3.8 Hz, 2H), 4.61 – 4.53 (m, 1H), 4.38 (q, *J* = 8.0 Hz, 1H), 4.09 (dq, *J* = 53.0, 7.1 Hz, 2H), 3.70 (d, *J* = 17.3 Hz, 1H), 3.11 – 3.08 (m, 1H), 2.97 (td, *J* = 15.3, 5.4 Hz, 2H), 2.76 (dd, *J* = 14.0, 8.9 Hz, 1H), 2.21 – 2.07 (m, 4H), 1.82 – 1.79 (m, 3H), 1.61 (s, 11H), 1.38 (d, *J* = 8.3 Hz, 14H), 1.36 (d, *J* = 10.4 Hz, 12H), 1.21 (d, *J* = 4.1 Hz, 9H). **HRMS-ESI** $C_{55}H_{78}F_3N_7O_{13}$: calculated (M+H)+ m/z 1102.5688; found (M+H)+ m/z 1102.5669.

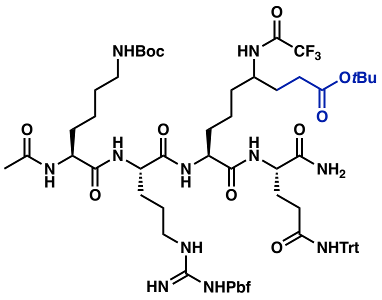

Ac - *L* - Lys(Boc) - *L* - Arg(Pbf) - *L* - Lys(Tfa) - *L* - Gln(Trt) - NH_2 and *tert*-butyl acrylate adduct (Compound 14). Prepared according to General Procedure D1, Condition B from Compound A4 (0.01 mmol) and *tert*-butyl acrylate (0.03 mmol). Purified via prep HPLC C18 reverse phase column (60% --> 90% $MeCN/H_2O$ w/ 0.1% TFA). White solid (0.0104 g, 74% isolated yield). **^{1}H NMR** (600 MHz, DMSO) δ 9.09 (d, *J* = 8.6 Hz, 1H), 8.60 (s, 1H), 7.95 (dt, *J* = 8.4, 4.1 Hz, 2H), 7.84 (t, *J* = 7.1 Hz, 1H), 7.74 (t, *J* = 7.4 Hz, 1H), 7.26 (t, *J* = 7.6 Hz, 6H), 7.21 – 7.14 (m, 10H), 7.05 – 6.97 (m, 1H), 6.71 (d, *J* = 6.1 Hz, 1H), 6.35 (s, 1H), 4.23 – 4.05 (m, 4H), 3.73 (s, 1H), 2.98 (d, *J* = 28.2 Hz, 4H), 2.87 (dd, *J* = 11.6, 5.9 Hz, 2H), 2.47 (s, 3H), 2.42 (s, 3H), 2.29 (qd, *J* = 8.8, 7.6, 4.2 Hz, 2H), 2.20 – 2.11 (m, 2H), 2.00 (s, 3H), 1.85 – 1.78 (m, 4H), 1.75 – 1.57 (m, 6H), 1.49 – 1.34 (m, 39H). **HRMS-ESI** $C_{71}H_{98}F_3N_{11}O_{14}S$: calculated (M+H)+ m/z 1418.7046; found (M+H)+ m/z 1418.7008.

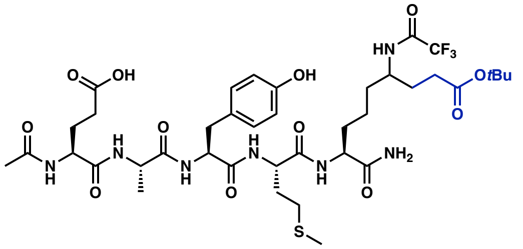

Ac-*L*-Glu-*L*-Ala-*L*-Tyr-*L*-Met-*L*-Lys(Tfa)-NH_2 and *tert*-butyl acrylate adduct (Compound 15). Prepared according to General Procedure D1, Condition B from Compound A5 (0.01 mmol) and *tert*-butyl acrylate (0.03 mmol). Purified via prep HPLC C18 reverse phase column (30% --> 60% $MeCN/H_2O$ w/ 0.1% TFA). White solid (0.0069 g, 76% isolated yield). **^{1}H NMR** (500 MHz, DMSO) δ 12.06 (s, 1H), 9.20 – 8.98 (m, 2H), 8.02 (dd, *J* = 17.2, 7.3 Hz, 2H), 7.90 (dd, *J* = 8.0, 5.0 Hz, 2H), 7.74 (dd, *J* = 8.0, 2.4 Hz, 1H), 7.26 (d, *J* = 12.2 Hz, 1H), 7.01 (d, *J* = 8.4 Hz, 3H), 6.66 – 6.55 (m, 2H), 4.33 (ddd, *J* = 24.7, 10.7, 6.8 Hz, 2H), 4.24 – 4.07 (m, 3H), 3.73 (t, *J* = 4.6 Hz, 1H), 2.90 (dd, *J* = 14.2, 4.5 Hz, 1H), 2.71 (dd, *J* = 14.2, 9.2 Hz, 1H), 2.41 (td, *J* = 9.7, 9.1, 5.4 Hz, 2H), 2.23 (t, *J* = 7.9 Hz, 2H), 2.17 – 2.10 (m, 1H), 2.02 (s, 4H), 1.93 – 1.79 (m, 6H), 1.76 – 1.66 (m, 2H), 1.61 (s, 2H), 1.53 – 1.41 (m, 3H), 1.37 (d, *J* = 1.5 Hz, 9H), 1.24 (d, *J* = 5.6 Hz, 2H), 1.18 – 1.11 (m, 3H). **HRMS-ESI** $C_{39}H_{58}F_3N_7O_{12}S$: calculated (M+H)+ m/z 906.3895; found (M+H)+ m/z 906.3889.

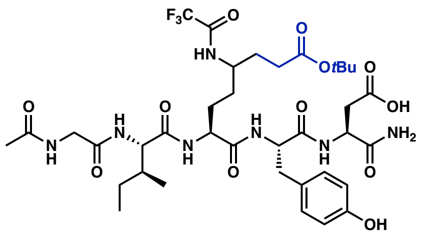

Ac-Gly-*L*-Ile-*L*-Orn(Tfa)-*L*-Tyr-*L*-Asp-NH_2 and *tert*-butyl acrylate adduct (Compound 16). Prepared according to General Procedure D1, Condition B from Compound A6 (0.01 mmol) and *tert*-butyl acrylate (0.03 mmol). Purified via prep HPLC C18 reverse phase column (30% --> 60% $MeCN/H_2O$ w/ 0.1% TFA). White solid (0.0069 g, 82% isolated yield). **^{1}H NMR** (500 MHz, DMSO) δ 12.24 (s, 1H), 9.27 – 8.98 (m, 2H), 8.07 (ddq, *J* = 27.7, 14.2, 7.8, 6.2 Hz, 3H), 7.86 (t, *J* = 8.7 Hz, 1H), 7.77 (dd, *J* = 7.6, 2.5 Hz, 1H), 7.09 (d, *J* = 6.1 Hz, 1H), 7.02 – 6.91 (m, 3H), 6.66 – 6.54 (m, 2H), 4.44 (p, *J* = 7.7, 7.0 Hz, 1H), 4.35 (td, *J* = 8.1, 4.2 Hz, 1H), 4.16 (dd, *J* = 9.6, 6.0 Hz, 2H), 3.72 (q, *J* = 6.2 Hz, 3H), 2.87 (dt, *J* = 13.9, 4.5 Hz, 1H), 2.75 – 2.61 (m, 2H), 2.45 (s, 1H), 2.20 – 2.11 (m, 1H), 1.84 (s, 3H), 1.70 (p, *J* = 6.7, 6.3 Hz, 2H), 1.58 (dq, *J* = 19.7, 7.0, 6.0 Hz,

2H), 1.38 (s, 14H), 1.08 – 0.99 (m, 1H), 0.81 – 0.74 (m, 6H). **HRMS-ESI** $C_{37}H_{54}F_3N_7O_{12}$: calculated (M+H)+ m/z 846.3861; found (M+H)+ m/z 846.3870.

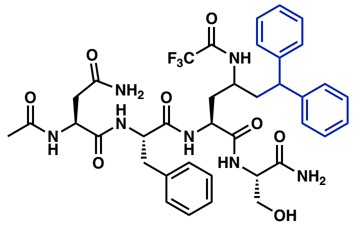

Ac-*L*-Asn-*L*-Phe-*L*-Dab(Tfa)-*L*-Ser-NH2 and 1,1-diphenylethylene adduct (Compound 17). Prepared according to General Procedure D1, Condition B from Compound A7 (0.01 mmol) and 1,1-diphenylethylene (0.03 mmol). Purified via prep HPLC C18 reverse phase column (40% --> 70% MeCN/H2O w/ 0.1% TFA). White solid (0.0031 g, 40% isolated yield). **^{1}H NMR** (500 MHz, DMSO) δ 9.19 (dd, *J* = 71.6, 8.6 Hz, 1H), 8.25 (dd, *J* = 57.4, 8.2 Hz, 1H), 8.02 – 7.81 (m, 2H), 7.53 – 7.44 (m, 1H), 7.29 – 7.09 (m, 18H), 7.00 (d, *J* = 26.0 Hz, 1H), 4.85 (s, 1H), 4.62 – 4.34 (m, 2H), 4.28 – 4.09 (m, 2H), 3.95 – 3.76 (m, 1H), 3.67 – 3.50 (m, 2H), 2.97 (ddd, *J* = 18.7, 14.1, 4.2 Hz, 1H), 2.73 (ddd, *J* = 43.1, 14.2, 8.9 Hz, 1H), 2.46 – 2.14 (m, 4H), 2.05 – 1.91 (m, 3H), 1.79 (d, *J* = 5.1 Hz, 3H). **HRMS-ESI** $C_{38}H_{44}F_3N_7O_8$: calculated (M+H)+ m/z 784.3282; found (M+H)+ m/z 784.3282.

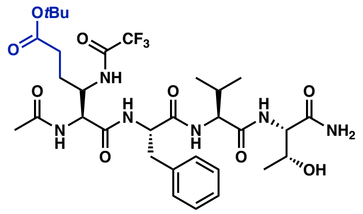

Ac-*L*-Dap(Tfa)-*L*-Phe-*L*-Val-*L*-Thr-NH2 and *tert*-butyl acrylate adduct (Compound 18). Prepared according to General Procedure D1, Condition B from Compound A8 (0.01 mmol) and *tert*-butyl acrylate (0.03 mmol). Purified via prep HPLC C18 reverse phase column (30% --> 60% MeCN/H2O w/ 0.1% TFA). White solid (0.0037 g, 52% isolated yield). **^{1}H NMR** (500 MHz, DMSO) δ 9.12 (d, *J* = 9.5 Hz, 1H), 8.33 (d, *J* = 8.8 Hz, 1H), 8.26 (d, *J* = 7.1 Hz, 1H), 7.98 (s, 1H), 7.68 (d, *J* = 8.6 Hz, 1H), 7.25 (d, *J* = 6.9 Hz, 4H), 7.18 (q, *J* = 6.4, 5.0 Hz, 1H), 7.07 (d, *J* = 12.2 Hz, 2H), 4.90 (d, *J* = 5.0 Hz, 1H), 4.71 – 4.62 (m, 1H), 4.32 – 3.96 (m, 5H), 3.02 (dd, *J* = 14.2, 4.4 Hz, 1H), 2.74 (dd, *J* = 14.3, 9.7 Hz, 1H), 2.01 (dq, *J* = 14.5, 7.7, 7.2 Hz, 2H), 1.82 (s, 3H), 1.74 – 1.66 (m, 1H), 1.48 (q, *J* = 6.9, 6.0 Hz, 1H), 1.38 (s, 9H), 1.02 (d, *J* = 6.3 Hz, 3H), 0.85 (d, *J* = 6.5 Hz, 6H). **HRMS-ESI** $C_{32}H_{47}F_3N_6O_9$: calculated (M+H)+ m/z 717.3435; found (M+H)+ m/z 717.3425.

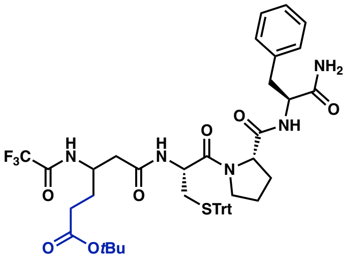

Tfa-β-Ala-*L*-Cys(Trt)-*L*-Pro-*L*-Phe-NH2 and *tert*-butyl acrylate adduct (Compound 19). Prepared according to General Procedure D1, Condition B from Compound A9 (0.01 mmol) and *tert*-butyl acrylate (0.03 mmol). Purified via prep HPLC C18 reverse phase column (40% --> 70% MeCN/H2O w/ 0.1% TFA). White solid (0.0067 g, 74% isolated yield). **^{1}H NMR** (500 MHz, DMSO) δ 9.17 (dd, *J* = 8.7, 2.7 Hz, 1H), 8.44 (t, *J* = 9.4 Hz, 1H), 7.57 (t, *J* = 7.6 Hz, 1H), 7.39 – 7.06 (m, 21H), 7.01 (d, *J* = 8.0 Hz, 1H), 4.49 – 4.32 (m, 2H), 4.15 (ddt, *J* = 18.9, 10.7, 5.2 Hz, 2H), 3.28 – 3.21 (m, 1H), 3.10 – 2.94 (m, 2H), 2.68 (dd, *J* = 14.0, 9.2 Hz, 1H), 2.39 (dt, *J* = 27.3, 6.6 Hz, 3H), 2.14 (dt, *J* = 11.8, 5.8 Hz, 1H), 1.99 (dt, *J* = 15.8, 6.9 Hz, 1H), 1.88 – 1.73 (m, 2H), 1.62 (dddd, *J* = 36.3, 21.7, 11.4, 6.5 Hz, 4H), 1.38 – 1.34 (m, 10H). **HRMS-ESI** $C_{48}H_{54}F_3N_5O_7S$: calculated (M+H)+ m/z 902.3774; found (M+H)+ m/z 902.3782.

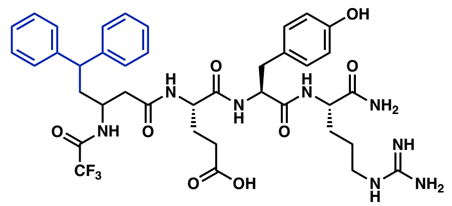

Tfa-β-Ala-*L*-Glu-*L*-Tyr-*L*-Arg-NH2 and 1,1-diphenylethylene adduct (Compound 20). Prepared according to General Procedure D1, Condition B from Compound A10 (0.01 mmol) and 1,1-diphenylethylene (0.03 mmol). Purified via prep HPLC C18 reverse phase column (40% --> 70% MeCN/H2O w/ 0.1% TFA). White solid (0.0045 g, 55% isolated yield). **^{1}H NMR** (500 MHz, DMSO) δ 12.08 (s, 1H), 9.26 (dd, *J* = 24.7, 8.4 Hz, 1H), 9.15 (d, *J* = 9.6 Hz, 1H), 8.14 – 7.76 (m, 3H), 7.51 – 7.05 (m, 16H), 7.03 – 6.96 (m, 2H), 6.61 (dd, *J* = 8.4, 6.5 Hz, 2H), 4.53 – 4.36 (m, 1H), 4.31 – 4.11 (m, 2H), 3.98 – 3.84 (m, 1H), 3.37 – 3.33 (m, 1H), 3.08 (q, *J* = 6.7 Hz, 2H), 2.91 (ddd, *J* = 14.5, 10.4, 4.7 Hz, 1H), 2.76 – 2.65 (m, 1H), 2.46 – 2.29 (m, 3H), 2.27 – 2.11 (m, 3H), 1.83 (dd, *J* = 12.2, 6.7 Hz, 1H), 1.67 (p, *J* = 7.2 Hz, 2H), 1.48 (dd, *J* = 33.6, 8.4 Hz, 3H). **HRMS-ESI** $C_{39}H_{47}F_3N_8O_8$: calculated (M+H)+ m/z 813.3547; found (M+H)+ m/z 813.3549.

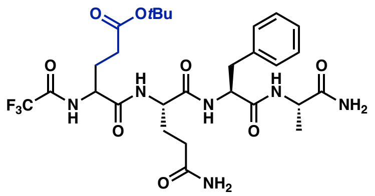

Tfa-Gly-*L*-Gln-*L*-Phe-*L*-Ala-NH2 and *tert*-butyl acrylate adduct (Compound 21). Prepared according to General Procedure D1, Condition A from Compound A11 (0.01 mmol) and *tert*-butyl acrylate (0.03 mmol). Purified via prep HPLC C18 reverse phase column (30% --> 60% MeCN/H_2O w/ 0.1% TFA). White solid (0.0046 g, 71% isolated yield). ^{1}**H NMR** (600 MHz, DMSO) δ 9.58 – 9.44 (m, 1H), 8.38 – 7.85 (m, 3H), 7.25 – 7.16 (m, 6H), 7.00 (s, 1H), 6.75 (s, 1H), 6.51 (s, 1H), 4.74 – 3.97 (m, 4H), 3.04 (ddd, *J* = 14.0, 7.4, 4.7 Hz, 1H), 2.84 – 2.76 (m, 1H), 2.20 (ddd, *J* = 16.5, 10.0, 5.8 Hz, 1H), 2.11 – 1.90 (m, 4H), 1.87 – 1.75 (m, 2H), 1.70 (dd, *J* = 9.6, 5.3 Hz, 1H), 1.39 (d, *J* = 1.1 Hz, 9H), 1.20 (dd, *J* = 7.1, 3.9 Hz, 3H). **HRMS-ESI** $C_{28}H_{39}F_3N_6O_8$: calculated (M+H)+ m/z 645.2860; found (M+H)+ m/z 645.2855.

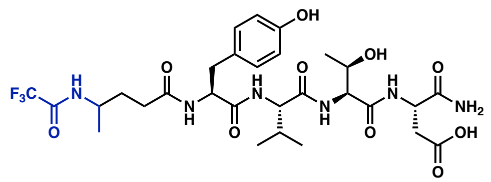

acryloyl-*L*-Tyr-*L*-Val-*L*-Thr-*L*-Asp-NH2 and *N*-ethyl-2,2,2-trifluoroacetamide adduct (Compound 22). Prepared according to General Procedure D1, Condition B from Compound A12 (0.01 mmol) and *N*-ethyl-2,2,2-trifluoroacetamide (0.03 mmol). Purified via prep HPLC C18 reverse phase column (30% --> 60% MeCN/H_2O w/ 0.1% TFA). White solid (0.0066 g, 95% isolated yield). ^{1}**H NMR** (500 MHz, DMSO) δ 12.29 (s, 1H), 9.20 (dd, *J* = 8.2, 3.1 Hz, 1H), 9.11 (s, 1H), 8.08 – 7.87 (m, 3H), 7.77 (dd, *J* = 8.0, 2.2 Hz, 1H), 7.16 (d, *J* = 20.3 Hz, 2H), 7.02 (d, *J* = 8.1 Hz, 2H), 6.61 (d, *J* = 8.2 Hz, 2H), 5.06 (d, *J* = 5.1 Hz, 1H), 4.49 (td, *J* = 8.0, 7.6, 4.1 Hz, 2H), 4.25 (ddd, *J* = 15.0, 8.3, 5.5 Hz, 2H), 4.01 (q, *J* = 5.3 Hz, 1H), 3.76 (p, *J* = 7.0 Hz, 1H), 2.88 (dt, *J* = 14.1, 4.3 Hz, 1H), 2.71 – 2.51 (m, 4H), 2.05 – 1.97 (m, 2H), 1.70 – 1.46 (m, 2H), 1.11 – 0.96 (m, 6H), 0.85 (dd, *J* = 9.1, 6.8 Hz, 6H). **HRMS-ESI** $C_{29}H_{41}F_3N_6O_{10}$: calculated (M+H)+ m/z 691.2915; found (M+H)+ m/z 691.2928.

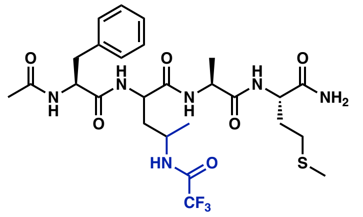

Ac-*L*-Phe-Dha-*L*-Ala-*L*-Met-NH2 and *N*-ethyl-2,2,2-trifluoroacetamide adduct (Compound 23). Prepared according to General Procedure D1, Condition B from Compound A13 (0.01 mmol) and *N*-ethyl-2,2,2-trifluoroacetamide (0.03 mmol). Purified via prep HPLC C18 reverse phase column (30% --> 60% MeCN/H_2O w/ 0.1% TFA). White solid (0.0054 g, 88% isolated yield). ^{1}**H NMR** (500 MHz, DMSO) δ 9.37 – 9.09 (m, 1H), 8.65 – 8.26 (m, 1H), 8.25 – 7.97 (m, 2H), 7.89 – 7.74 (m, 1H), 7.28 – 7.14 (m, 6H), 7.04 (d, *J* = 15.8 Hz, 1H), 4.53 – 4.38 (m, 1H), 4.23 (dtd, *J* = 15.5, 12.0, 10.0, 6.0 Hz, 2H), 4.01 – 3.77 (m, 2H), 2.97 (ddd, *J* = 19.7, 13.5, 5.0 Hz, 1H), 2.84 – 2.64 (m, 1H), 2.48 – 2.31 (m, 2H), 2.13 – 1.90 (m, 5H), 1.86 – 1.71 (m, 5H), 1.28 – 0.96 (m, 6H). **HRMS-ESI** $C_{26}H_{37}F_3N_6O_6S$: calculated (M+H)+ m/z 619.2526; found (M+H)+ m/z 619.2529.

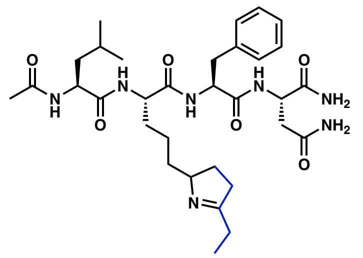

Ac-*L*-Leu-*L*-Lys(ethyl-dihydropyrrole)-*L*-Phe-*L*-Asn-NH2 (Compound 24). Prepared according to General Procedure E from Compound 5 (0.01 mmol). Purified via prep HPLC C18 reverse phase column (25% --> 55% MeCN/H_2O w/ 0.1% TFA). White solid (0.0044 g, 70% isolated yield). ^{1}**H NMR** (500 MHz, DMSO) δ 8.30 (q, *J* = 9.9, 8.8 Hz, 1H), 8.10 – 7.76 (m, 3H), 7.38 (d, *J* = 23.2 Hz, 1H), 7.26 – 7.10 (m, 6H), 7.01 – 6.87 (m, 2H), 4.57 – 4.52 (m, 1H), 4.23 (dt, *J* = 14.5, 7.1 Hz, 3H), 3.12 – 2.95 (m, 3H), 2.75 (dq, *J* = 14.9, 8.5, 7.5 Hz, 3H), 2.59 – 2.50 (m, 2H), 2.28 (d, *J* = 11.3 Hz, 1H), 1.82 (d, *J* = 4.3 Hz, 3H), 1.77 – 1.44 (m, 6H), 1.43 – 1.21 (m, 5H), 1.17 (t, *J* = 7.4 Hz, 3H), 0.84 (dd, *J* = 23.2, 6.5 Hz, 6H). **HRMS-ESI** $C_{32}H_{49}N_7O_6$: calculated (M+H)+ m/z 628.3823; found (M+H)+ m/z 629.3674.

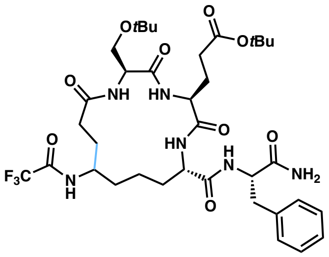

cyclo[acryloyl - *L* - Ser(*t*Bu) - *L* - Glu(O*t*Bu) - *L* - Lys(Tfa) - *L* - Phe - NH_2] (Compound 25). Prepared according to General Procedure D2, Condition B from Compound A14 (0.01 mmol). Purified via prep HPLC C18 reverse phase column (40% --> 70% MeCN/H_2O w/ 0.1% TFA). White solid (0.0041 g, 53% isolated yield). Isolated as >20:1 mixture of diastereomers. **^{1}H NMR** (500 MHz, DMSO) δ 9.04 (d, *J* = 8.2 Hz, 1H), 8.05 (d, *J* = 6.1 Hz, 1H), 7.83 (d, *J* = 8.7 Hz, 1H), 7.68 (d, *J* = 8.4 Hz, 1H), 7.39 (dd, *J* = 8.1, 4.5 Hz, 1H), 7.28 – 7.20 (m, 5H), 7.18 – 7.15 (m, 1H), 7.06 (s, 1H), 4.35 (td, *J* = 8.6, 4.9 Hz, 1H), 4.22 (td, *J* = 9.8, 9.2, 4.0 Hz, 2H), 4.14 (dd, *J* = 6.3, 3.4 Hz, 1H), 4.07 (t, *J* = 9.8 Hz, 1H), 3.64 (dd, *J* = 9.3, 4.0 Hz, 1H), 3.46 (dd, *J* = 9.3, 3.7 Hz, 1H), 2.98 (dd, *J* = 13.7, 4.8 Hz, 1H), 2.77 (dd, *J* = 13.7, 8.9 Hz, 1H), 2.31 (ddd, *J* = 15.9, 10.6, 4.8 Hz, 1H), 2.18 (ddd, *J* = 16.7, 10.3, 6.4 Hz, 1H), 2.04 – 1.94 (m, 2H), 1.89 (t, *J* = 11.7 Hz, 1H), 1.79 – 1.58 (m, 3H), 1.49 – 1.43 (m, 2H), 1.38 (s, 11H), 1.27 (d, *J* = 8.5 Hz, 2H), 1.15 (s, 9H). **HRMS-ESI** $C_{36}H_{53}F_3N_6O_9$: calculated (M+H)+ m/z 771.3904; found (M+H)+ m/z 771.3899.

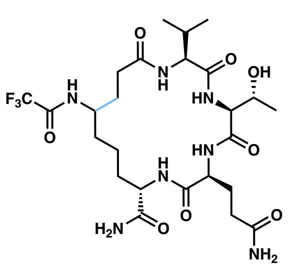

cyclo[acryloyl-*L*-Val-*L*-Thr-*L*-Gln-*L*-Lys(Tfa)-NH_2] (Compound 26). Prepared according to General Procedure D2, Condition B from Compound A15 (0.01 mmol). Purified via prep HPLC C18 reverse phase column (20% --> 40% MeCN/H_2O w/ 0.1% TFA). White solid (0.0028 g, 45% isolated yield). Isolated as >20:1 mixture of diastereomers. **^{1}H NMR** (500 MHz, DMSO) δ 9.27 (d, *J* = 8.8 Hz, 1H), 8.22 – 7.98 (m, 3H), 7.31 (d, *J* = 35.9 Hz, 2H), 7.11 (d, *J* = 8.2 Hz, 1H), 6.96 (d, *J* = 16.4 Hz, 1H), 6.80 (s, 1H), 4.89 (s, 1H), 4.31 (dd, *J* = 8.4, 3.1 Hz, 1H), 4.27 – 4.18 (m, 2H), 4.13 (dd, *J* = 9.4, 4.7 Hz, 1H), 4.01 (dt, *J* = 8.2, 5.6 Hz, 1H), 3.79 (s, 1H), 2.32 – 2.06 (m, 5H), 2.01 – 1.95 (m, 1H), 1.86 – 1.65 (m, 5H), 1.46 (d, *J* = 12.2 Hz, 1H), 1.36 (q, *J* = 12.0, 11.4 Hz, 3H), 1.03 (d, *J* = 6.3 Hz, 3H), 0.84 (dd, *J* = 6.9, 3.9 Hz, 6H). **HRMS-ESI** $C_{25}H_{40}F_3N_7O_8$: calculated (M+H)+ m/z 624.2969; found (M+H)+ m/z 624.2970.

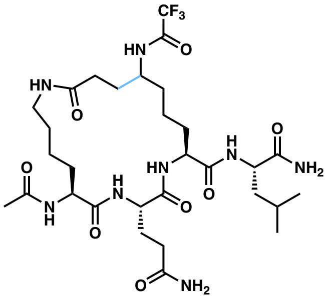

cyclo[Ac - *L* - Lys(acryloyl) - *L* - Gln - *L* - Lys(Tfa) - *L* - Leu - NH_2] (Compound 27). Prepared according to General Procedure D2, Condition B from Compound A16 (0.01 mmol). Purified via prep HPLC C18 reverse phase column (20% --> 40% MeCN/H_2O w/ 0.1% TFA). White solid (0.0030 g, 42% isolated yield). Isolated as >20:1 mixture of diastereomers. **^{1}H NMR** (500 MHz, DMSO) δ 9.20 (d, *J* = 8.5 Hz, 1H), 8.06 – 7.58 (m, 5H), 7.26 (d, *J* = 39.0 Hz, 2H), 6.97 (s, 1H), 6.75 (d, *J* = 19.8 Hz, 1H), 4.37 – 4.06 (m, 4H), 3.64 (d, *J* = 8.4 Hz, 1H), 3.24 – 3.18 (m, 1H), 2.86 (dd, *J* = 13.1, 5.6 Hz, 1H), 2.08 – 1.97 (m, 3H), 1.83 (s, 4H), 1.74 – 1.54 (m, 6H), 1.48 – 1.33 (m, 8H), 1.31 – 1.21 (m, 5H), 0.85 (dd, *J* = 24.5, 6.5 Hz, 6H). **HRMS-ESI** $C_{30}H_{49}F_3N_8O_8$: calculated (M+H)+ m/z 707.3704; found (M+H)+ m/z 707.3708.

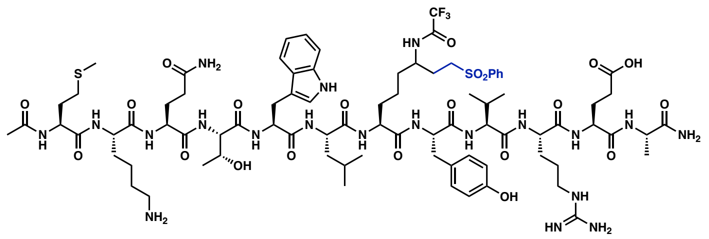

Ac - *L* - Met - *L* - Lys - *L* - Gln - *L* - Thr - *L* - Trp - *L* - Leu - *L* - Lys(Tfa) - *L* - Tyr - *L* - Val - *L* - Arg - *L* - Glu - *L* - Ala - NH_2 and phenyl vinyl sulfone adduct (Compound 28). Prepared according to General Procedure D3, Condition B from Compound A17 on TentaGel S RAM resin (protected and attached to bead) (0.01 mmol) and phenyl vinyl sulfone (0.03 mmol). Purified via prep HPLC C18 reverse phase column (30% --> 60% MeCN/H_2O w/ 0.1% TFA). White solid (0.0065 g, 35% isolated yield). **HRMS-ESI** $C_{83}H_{123}F_3N_{20}O_{21}S_2$: calculated (M+H)+ m/z 1857.8643; found (M+H)+ m/z 1857.8658.

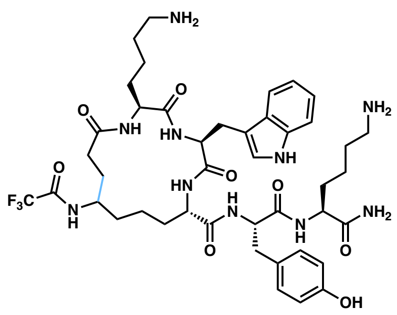

cyclo[acryloyl - *L* - Lys - *L* - Trp - *L* - Lys(Tfa) - *L* - Tyr - *L* - Lys - NH_2] (Compound 29). Prepared according to General Procedure D4, Condition B from Compound A18 on TentaGel S RAM resin (protected and attached to bead) (0.01 mmol). Purified via prep HPLC C18 reverse phase column (25% --> 55% MeCN/H_2O w/ 0.1% TFA). White solid (0.0058 g, 64% isolated yield). Isolated as 1.9:1 mixture of diastereomers. **^{1}H NMR** (600 MHz, DMSO) δ 10.89 – 10.64 (m, 1H), 9.49 – 8.96 (m, 2H), 8.23 (dd, *J* = 58.2, 5.7 Hz, 1H), 7.94 – 7.80 (m, 2H), 7.65 – 7.49 (m, 7H), 7.31 (t, *J* = 7.3 Hz, 1H), 7.20 – 7.12 (m, 2H), 7.08 – 6.97 (m, 5H), 6.65 – 6.61 (m, 2H), 4.60 – 3.70 (m, 6H), 3.20 – 3.06 (m, 2H), 2.99 – 2.63 (m, 6H), 2.22 – 2.14 (m, 1H), 2.03 – 1.93 (m, 2H), 1.66 (s, 1H), 1.56 – 1.36 (m, 12H), 1.33 – 1.25 (m, 6H). **HRMS-ESI** $C_{43}H_{59}F_3N_{10}O_8$: calculated (M+H)+ m/z 901.4548; found (M+H)+ m/z 901.4559.

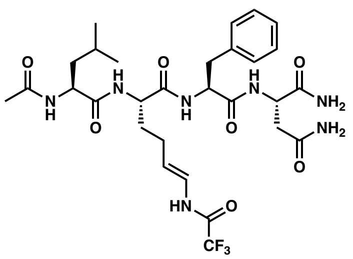

Ac-*L*-Leu-*L*-Lys(trifluoroenamide)-*L*-Phe-*L*-Asn-NH_2 (Compound 30). Prepared according to General Procedure D1, Condition B from Compound A1 (0.01 mmol) and TEMPO (0.03 mmol). Purified via prep HPLC C18 reverse phase column (30% --> 60% MeCN/H_2O w/ 0.1% TFA). White solid (0.0056 g, 90% isolated yield). **^{1}H NMR** (500 MHz, DMSO) δ 11.03 (d, *J* = 6.2 Hz, 1H), 8.85 (d, *J* = 8.0 Hz, 1H), 8.27 (d, *J* = 7.7 Hz, 1H), 8.19 (d, *J* = 7.9 Hz, 1H), 7.30 (s, 1H), 7.27 – 7.16 (m, 6H), 7.05 (s, 1H), 6.94 (s, 1H), 6.86 (s, 1H), 6.28 (s, 1H), 5.18 (d, *J* = 7.2 Hz, 1H), 4.82 – 4.25 (m, 4H), 3.03 – 2.99 (m, 1H), 2.81 (dd, *J* = 14.1, 8.9 Hz, 1H), 2.41 (dd, *J* = 15.8, 6.6 Hz, 2H), 2.06 – 2.00 (m, 1H), 1.81 (s, 4H), 1.65 – 1.59 (m, 1H), 1.52 – 1.49 (m, 2H), 1.40 (s, 1H), 1.04 (dd, *J* = 11.2, 6.7 Hz, 1H), 0.74 (dd, *J* = 55.8, 6.3 Hz, 6H). **HRMS** $C_{29}H_{40}F_3N_7O_7$: calculated (M+H)+ m/z 656.3020; found (M+H)+ m/z 656.3010.

7. Pictures of photoredox experimental setup

The following photos are to assist with reproducibility of General Procedure D.

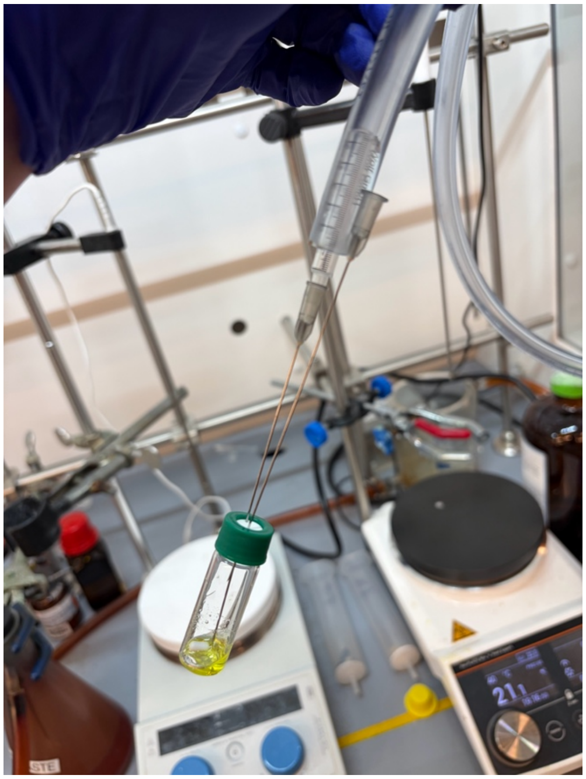

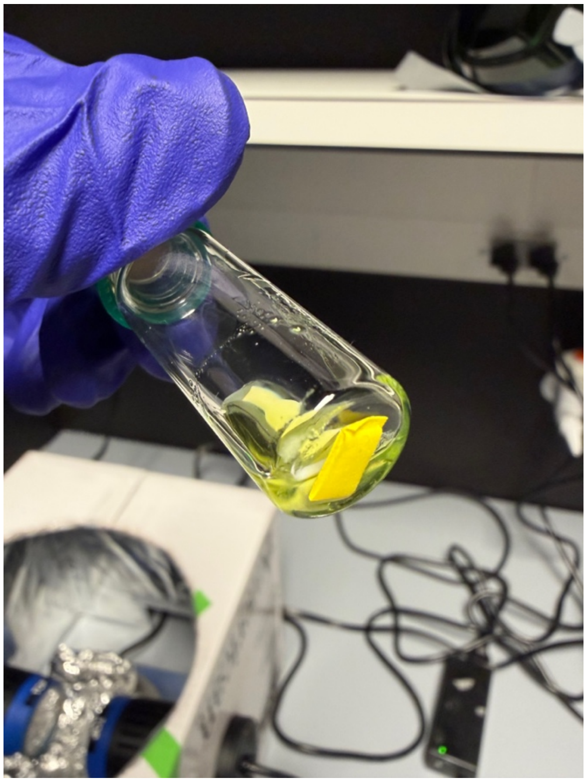

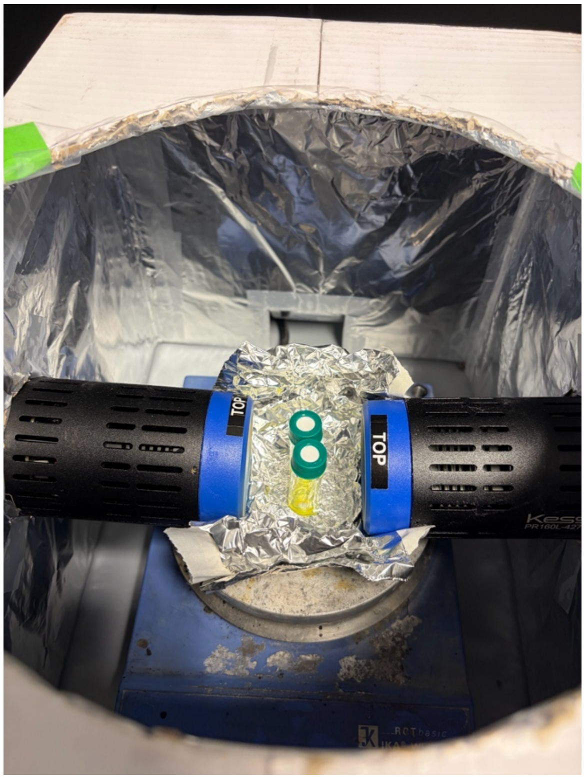

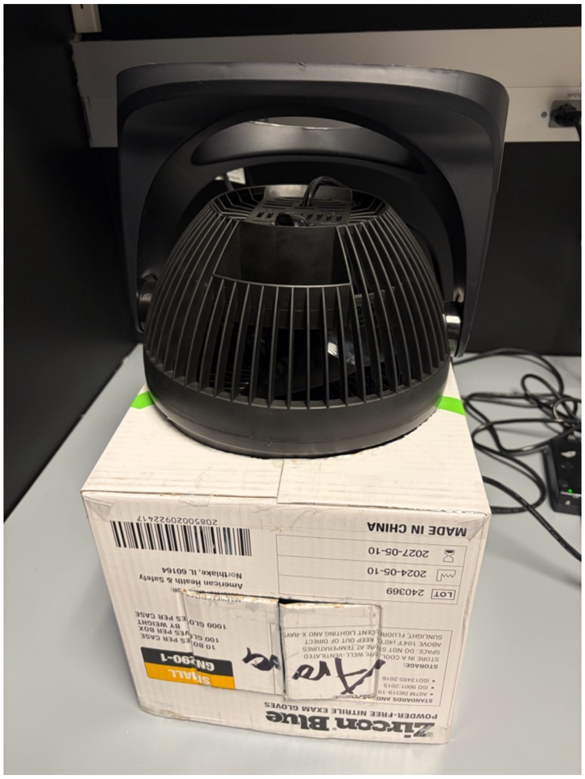

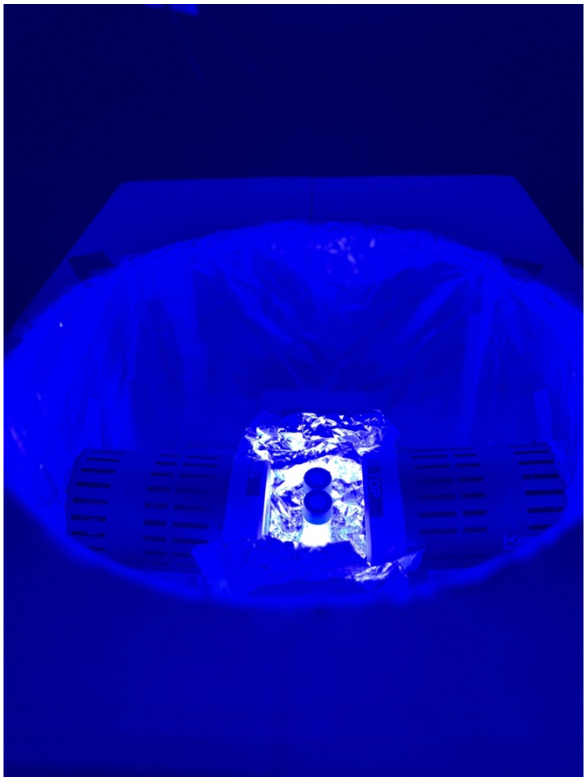

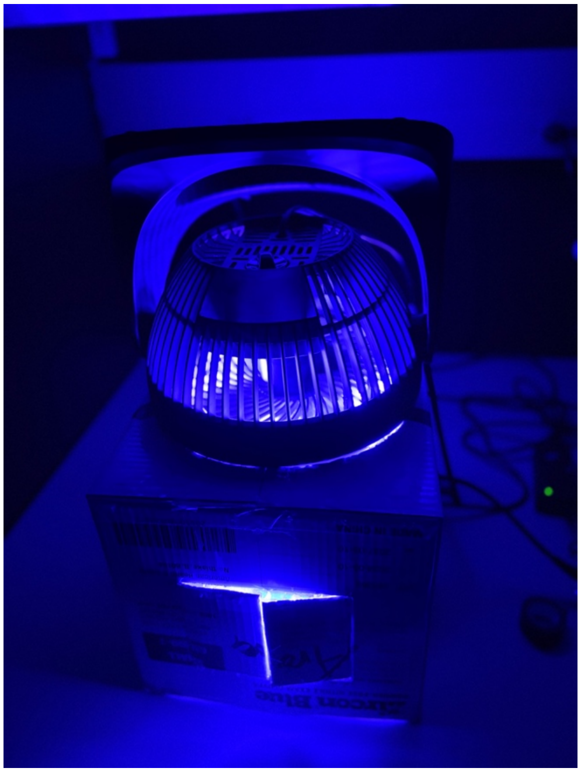

8. NMR and HRMS spectra

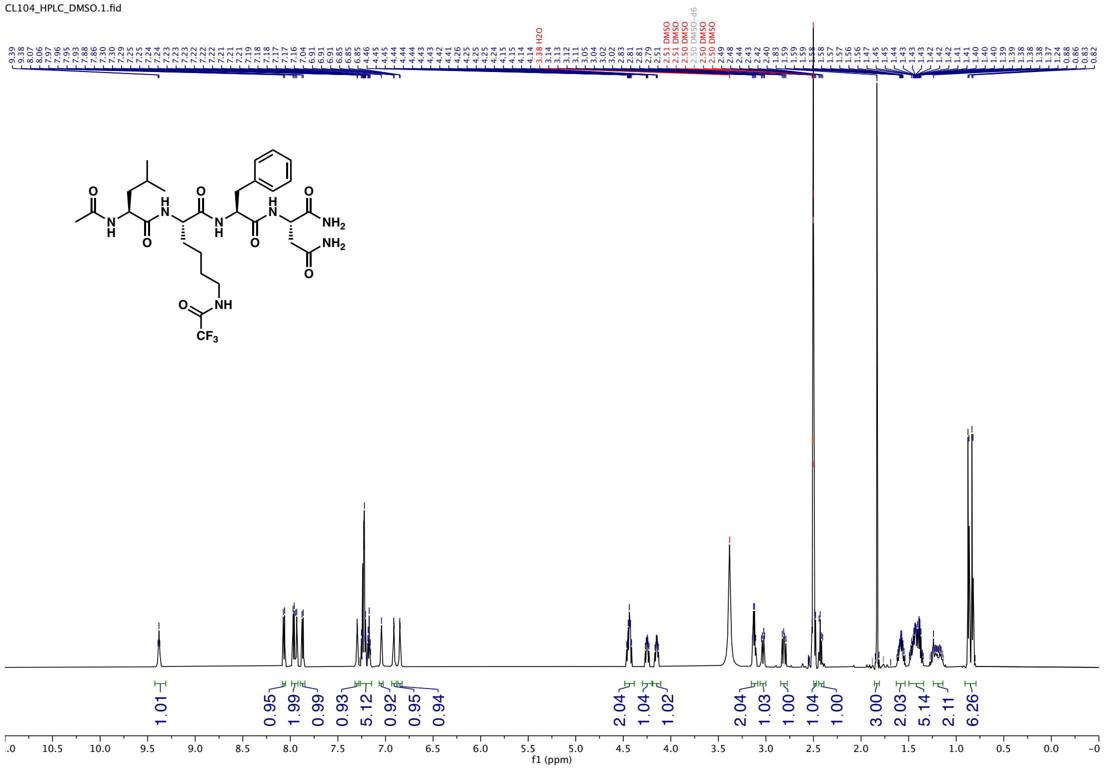

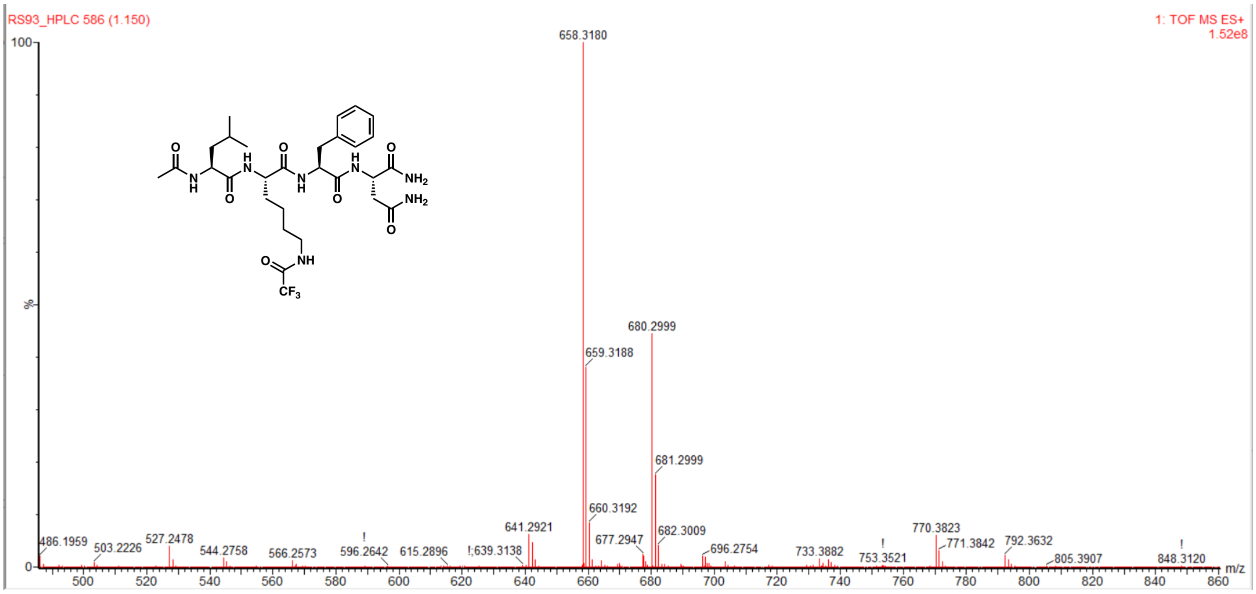

Deuterium labeling study

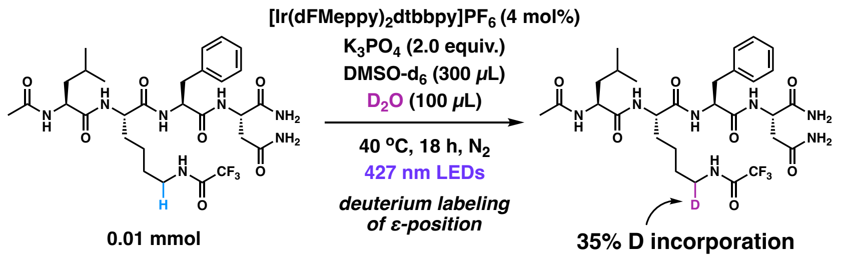

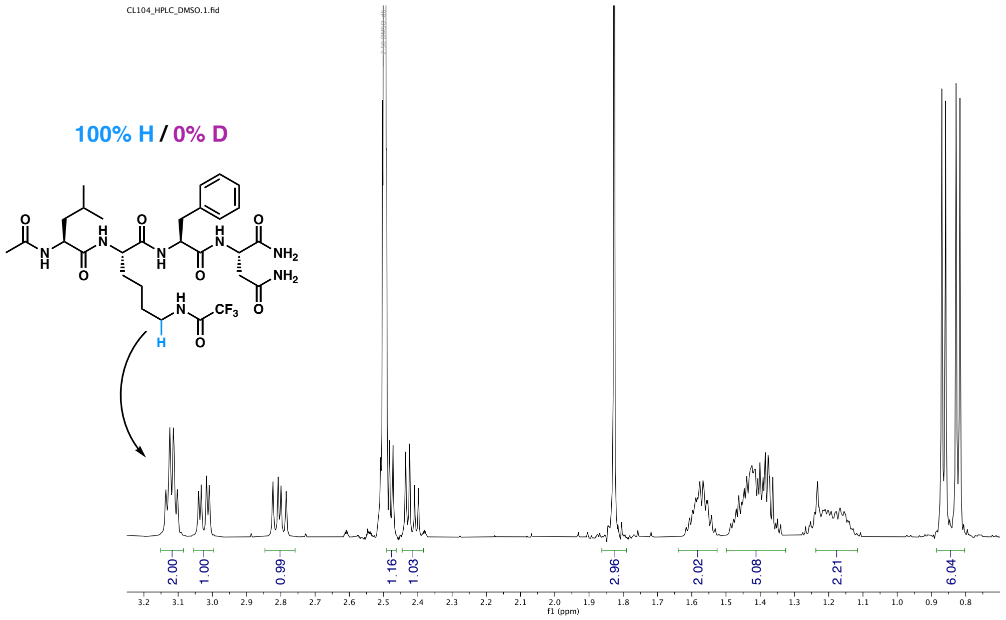

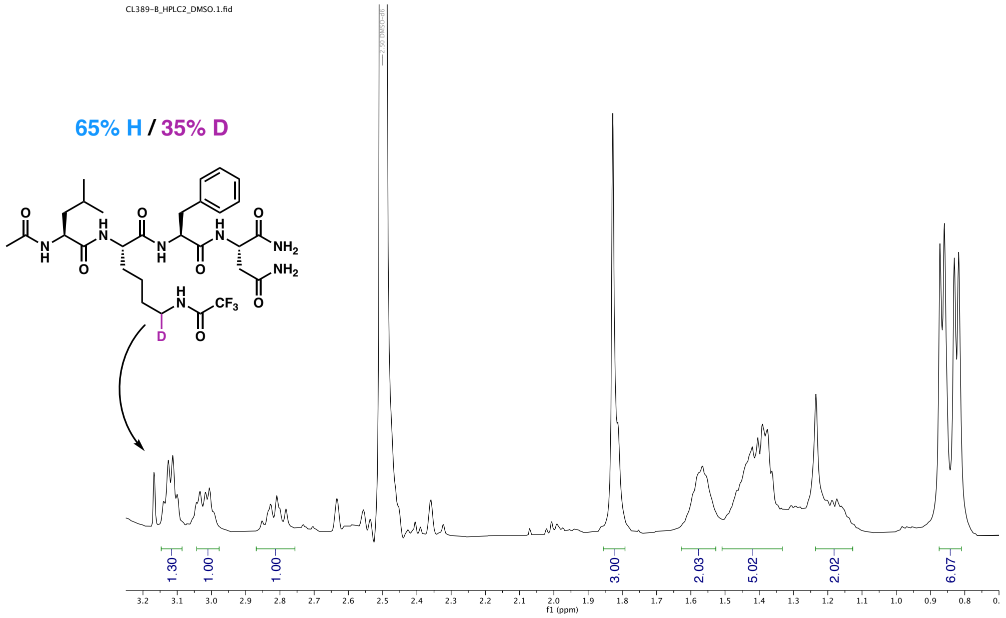

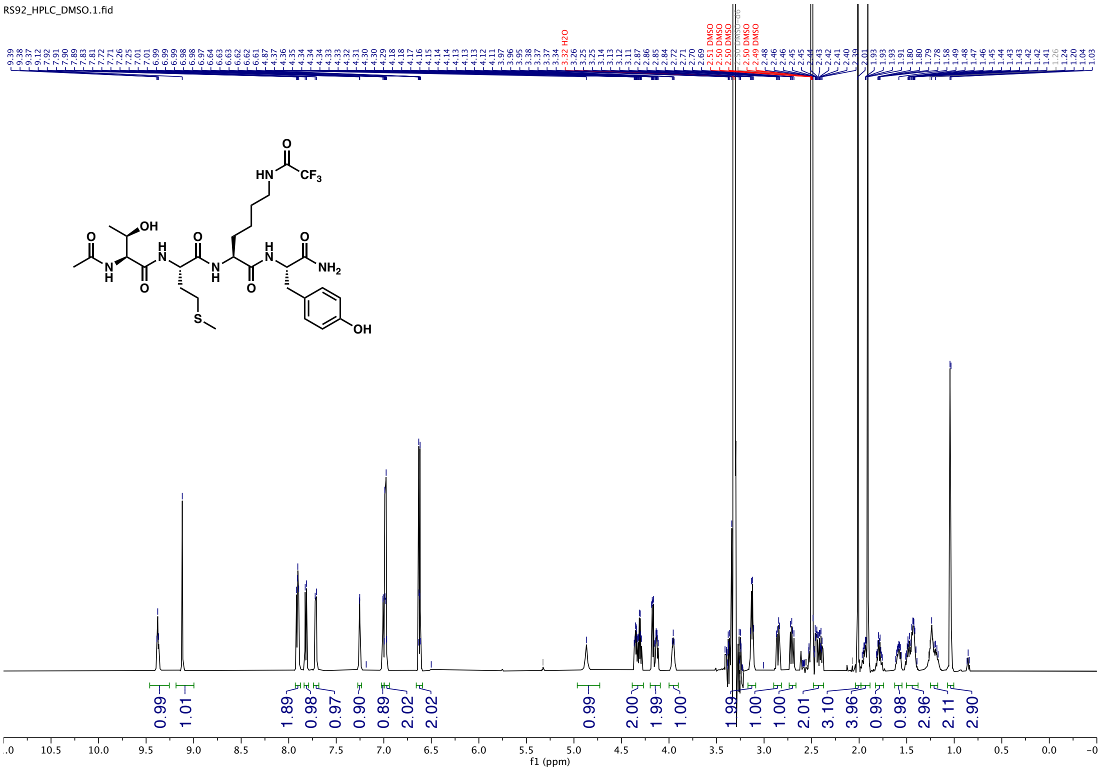
RS92_HPLC_DMSO.1.fid
f1 (ppm)
HN
CF3
OH
NH2
S
OH
0.99
1.01
1.89
0.98
0.97
0.90
0.89
2.02
2.02
0.99
2.00
1.99
1.00
1.99
1.00
1.00
2.01
3.10
3.96
0.99
0.98
2.96
2.11
2.90

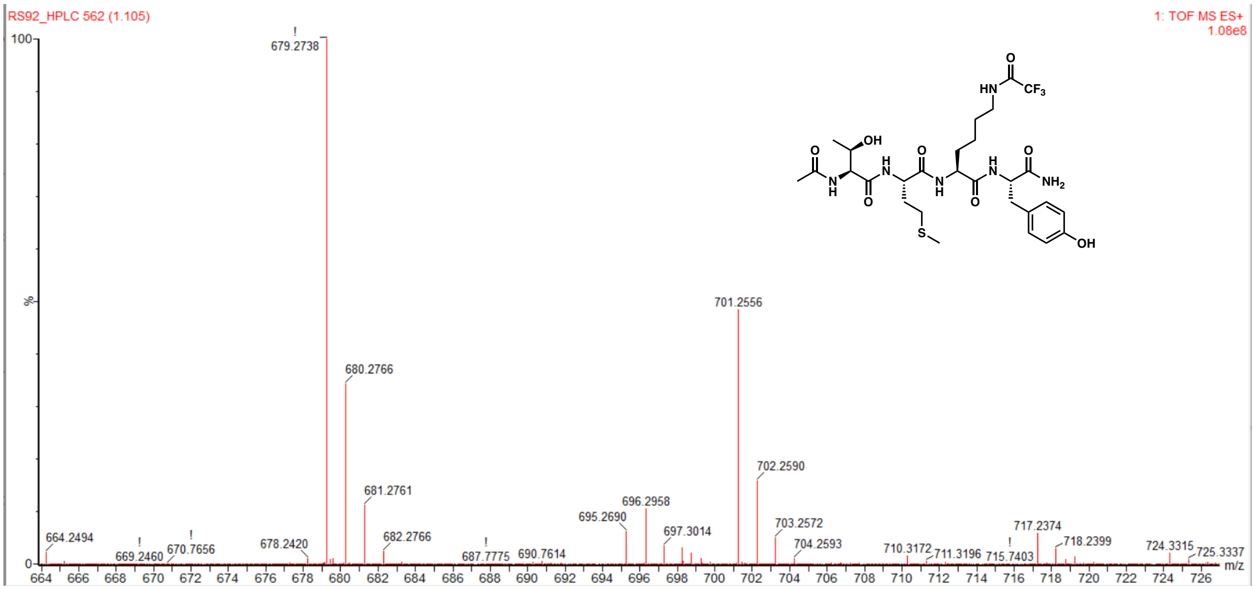
RS92_HPLC 562 (1.105)
1: TOF MS ES+
1.08e8
679.2738
701.2556
680.2766
702.2590
681.2761
696.2958
695.2690
697.3014
703.2572
717.2374
718.2399
664.2494
669.2460
670.7656
678.2420
682.2766
687.7775
690.7614
704.2593
710.3172
711.3196
715.7403
724.3315
725.3337
m/z
%

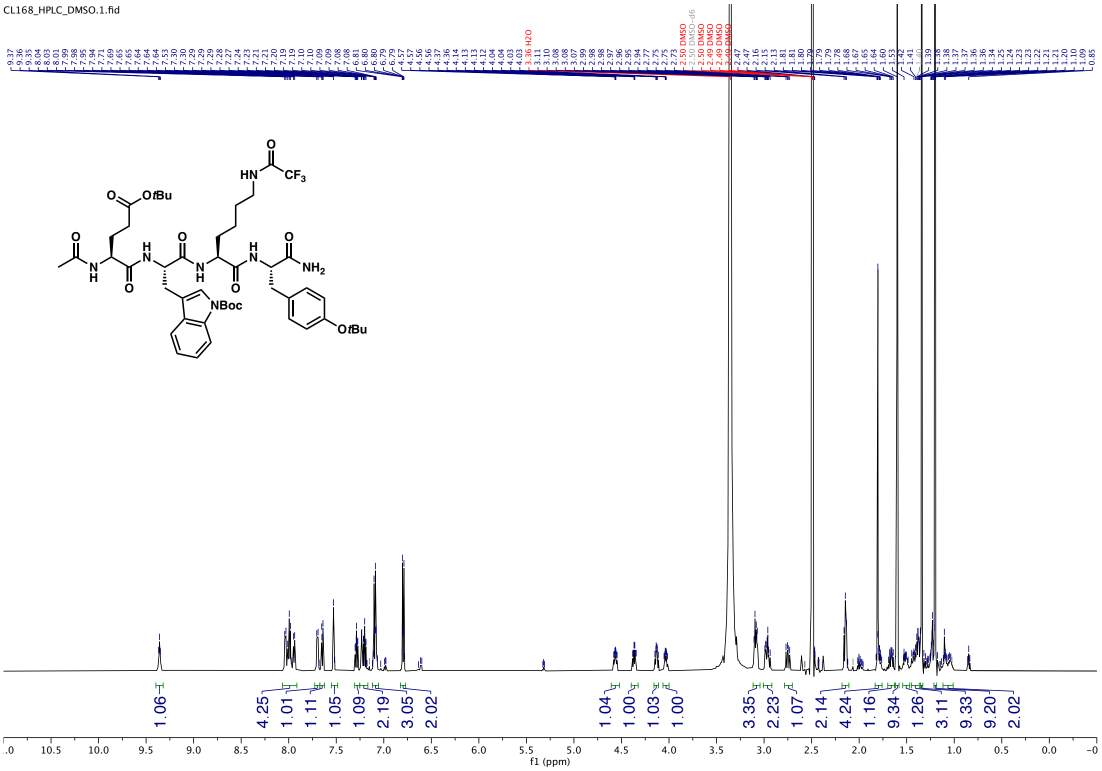
CL168_HPLC_DMSO.1.fid
HN
CF3
O
OtBu
NBoc
NH2
OtBu
1.06
4.25
1.01
1.11
1.05
1.09
2.19
3.05
2.02
1.04
1.00
1.03
1.00
3.35
2.23
1.07
2.14
4.24
1.16
9.34
1.26
3.11
9.33
9.20
2.02
f1 (ppm)

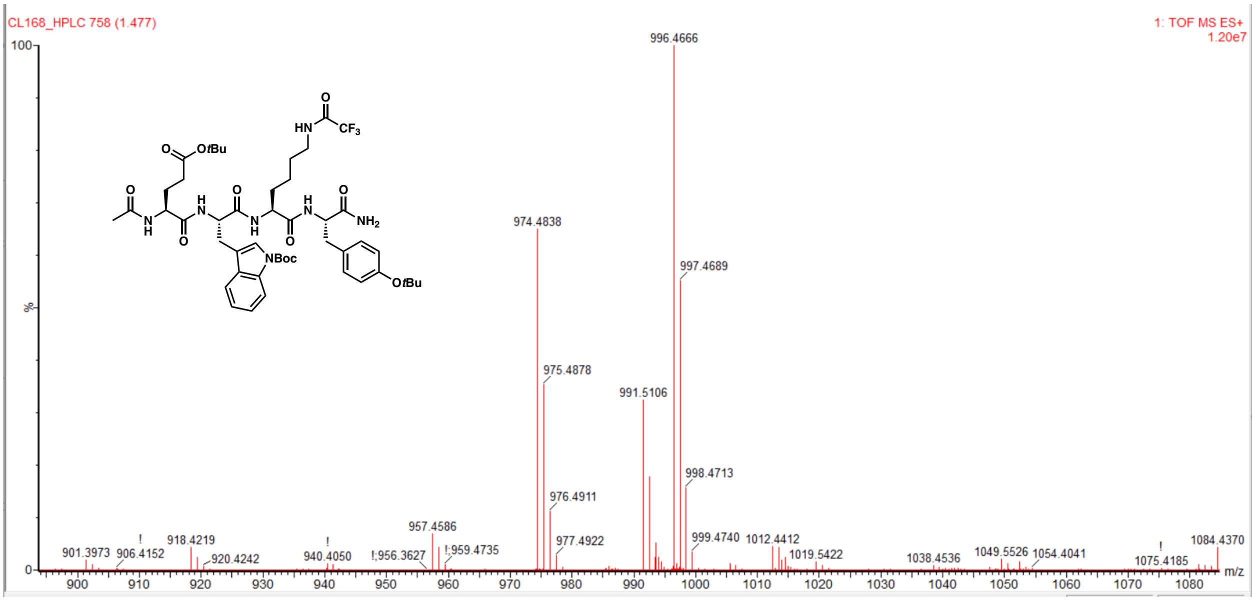
CL168_HPLC 758 (1.477)
1: TOF MS ES+
1.20e7
996.4666
974.4838
997.4689
975.4878
991.5106
998.4713
976.4911
977.4922
957.4586
999.4740
1012.4412
1019.5422
901.3973
906.4152
918.4219
920.4242
940.4050
956.3627
959.4735
1038.4536
1049.5526
1054.4041
1075.4185
1084.4370
%
m/z

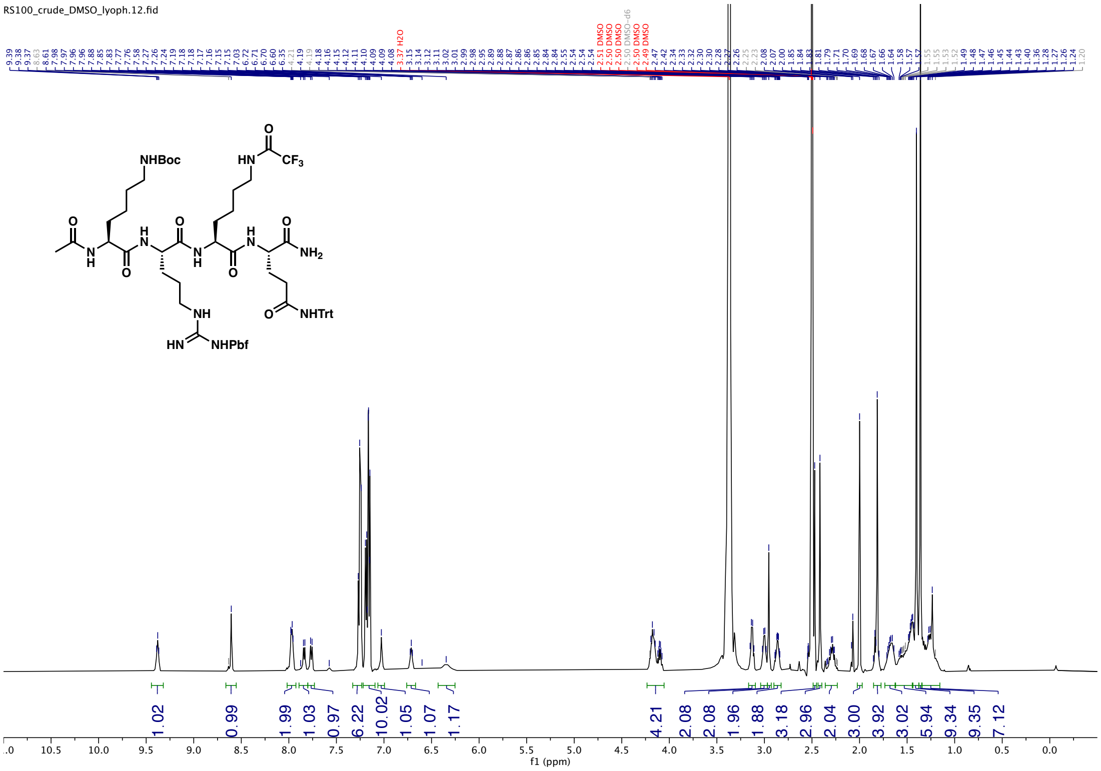

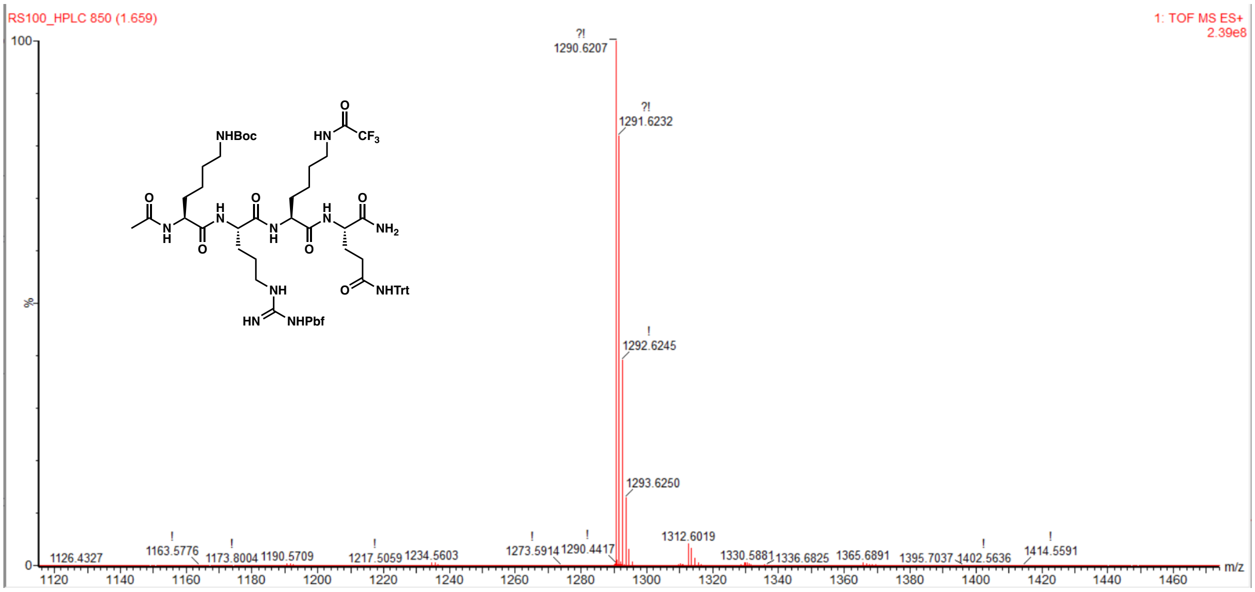

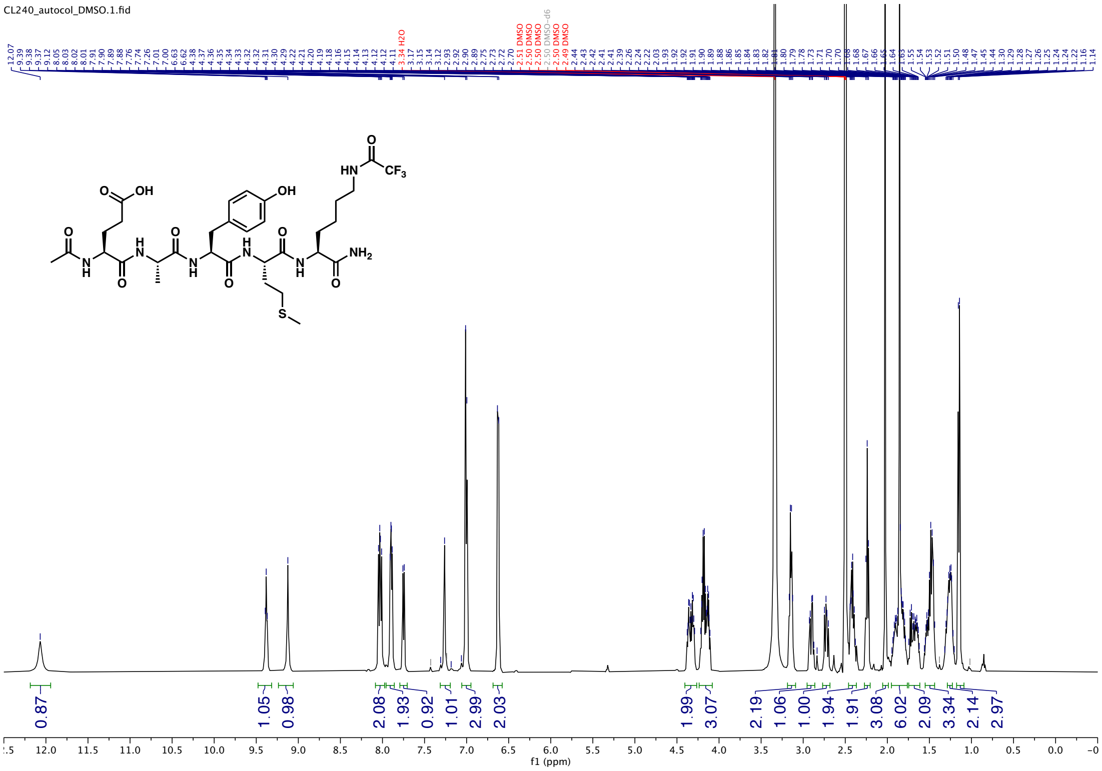
CL240_autocol_DMSO.1.fid
0.87
1.05
0.98
2.08
1.93
0.92
1.01
2.99
2.03
1.99
3.07
2.19
1.06
1.00
1.94
1.91
3.08
6.02
2.09
3.34
2.14
2.97
f1 (ppm)

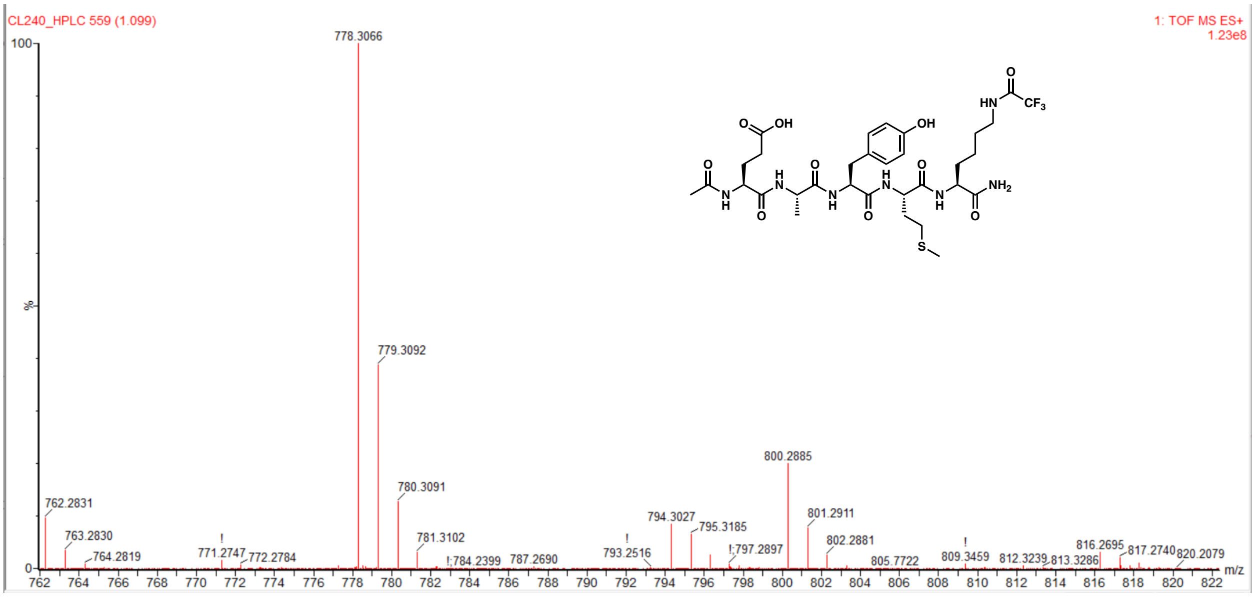
CL240_HPLC 559 (1.099)
1: TOF MS ES+
1.23e8
778.3066
779.3092
780.3091
781.3102
762.2831
763.2830
764.2819
771.2747
772.2784
784.2399
787.2690
793.2516
794.3027
795.3185
797.2897
800.2885
801.2911
802.2881
805.7722
809.3459
812.3239
813.3286
816.2695
817.2740
820.2079
%
m/z

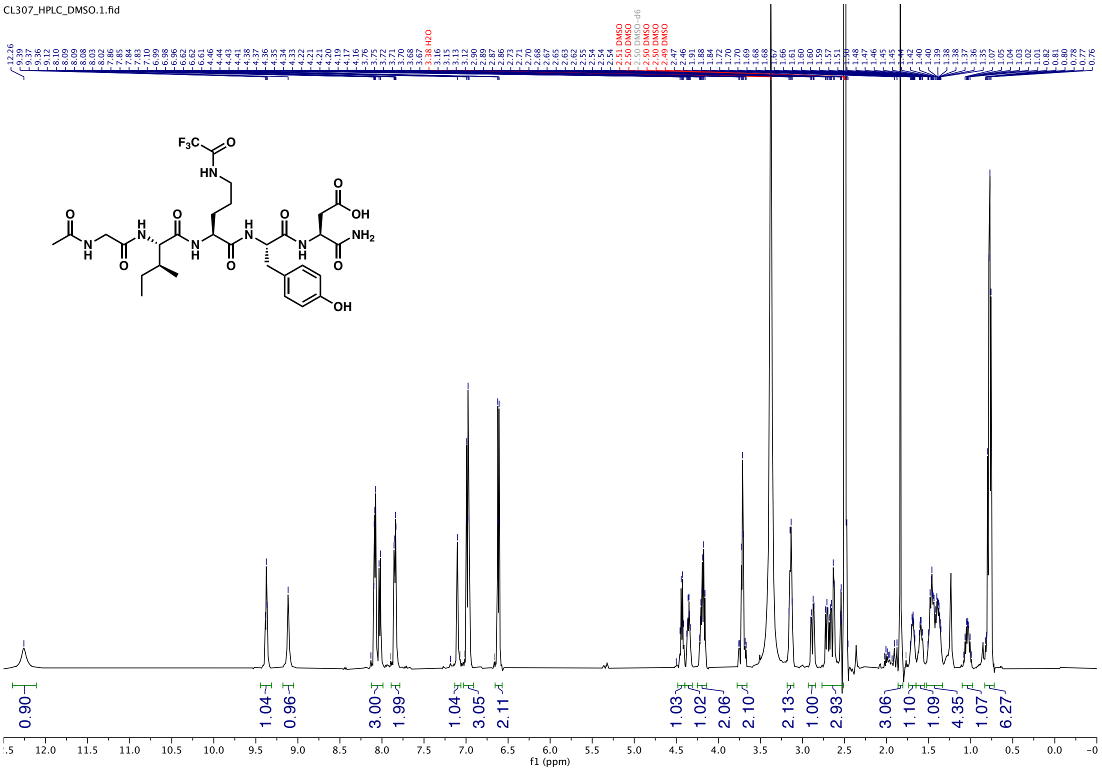
CL307_HPLC_DMSO.1.fid
0.90
1.04
0.96
3.00
1.99
1.04
3.05
2.11
1.03
1.02
2.06
2.10
2.13
1.00
2.93
3.06
1.10
1.09
4.35
1.07
6.27
f1 (ppm)

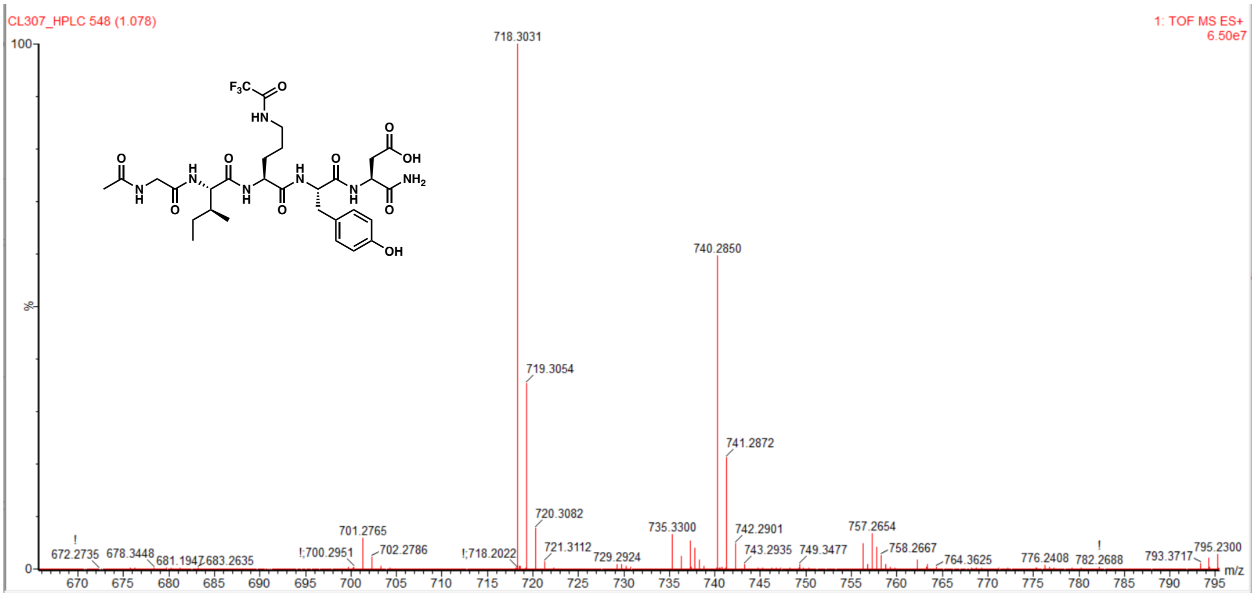
CL307_HPLC 548 (1.078)
1: TOF MS ES+
6.50e7
718.3031
740.2850
719.3054
741.2872
720.3082
735.3300
742.2901
757.2654
701.2765
702.2786
795.2300
m/z
%

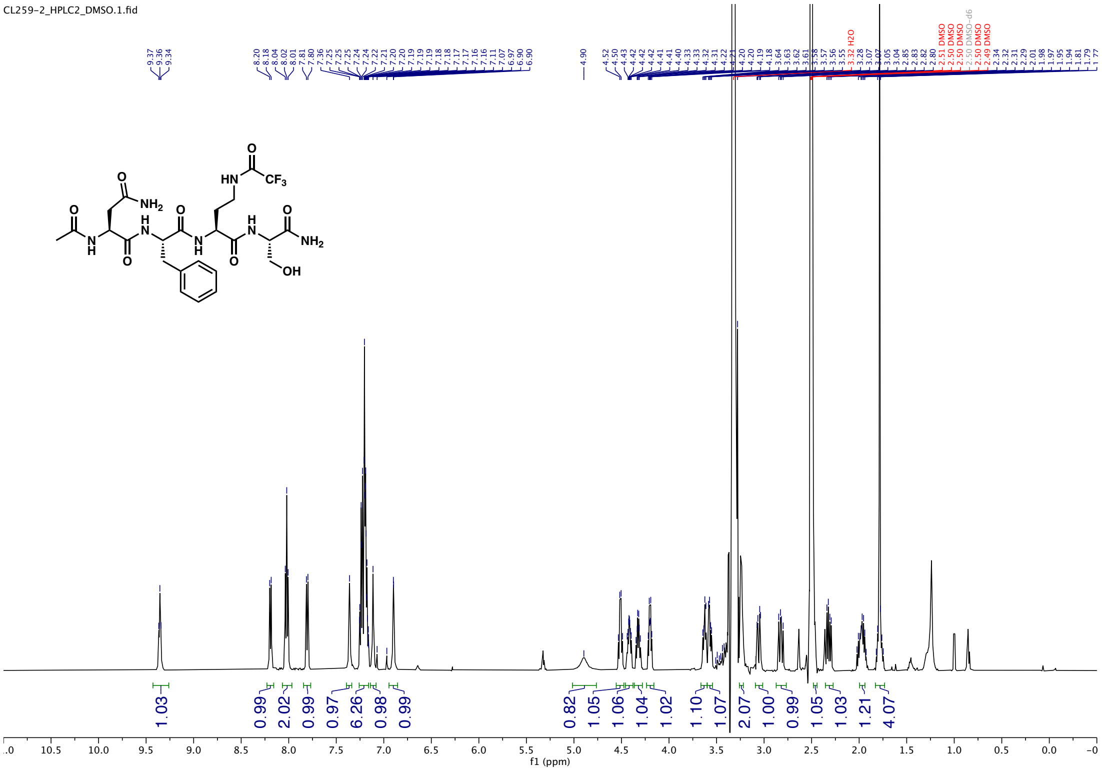
CL259-2_HPLC2_DMSO.1.fid
f1 (ppm)

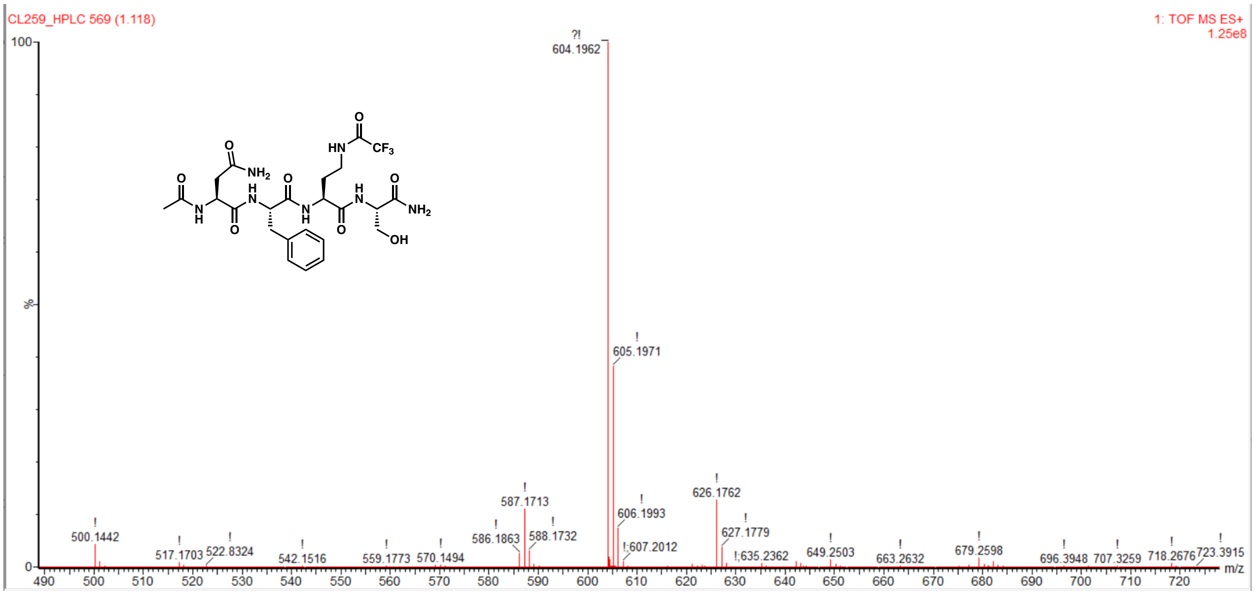
CL259_HPLC 569 (1.118)
1: TOF MS ES+
1.25e8
604.1962
605.1971
626.1762
587.1713
606.1993
627.1779
586.1863
588.1732
500.1442
607.2012
679.2598
635.2362
649.2503
522.8324
517.1703
542.1516
559.1773
570.1494
663.2632
696.3948
707.3259
718.2676
723.3915
m/z

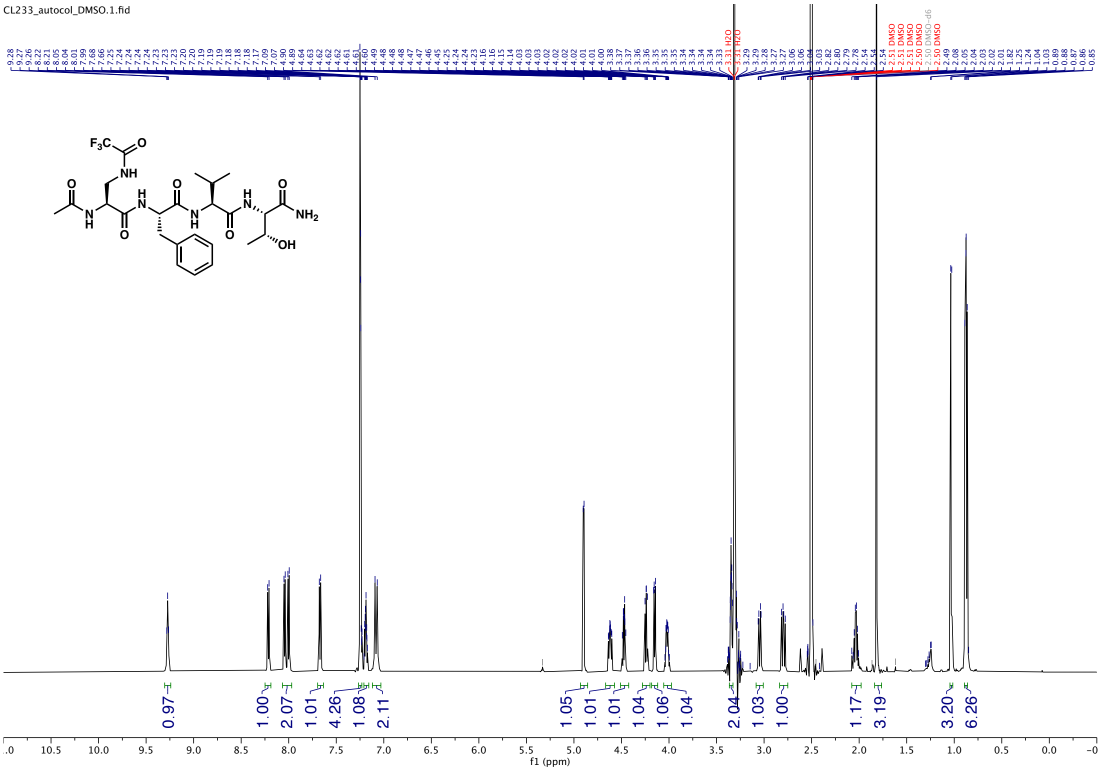
CL233_autocol_DMSO.1.fid
f1 (ppm)
F3C
O
NH
NH2
OH

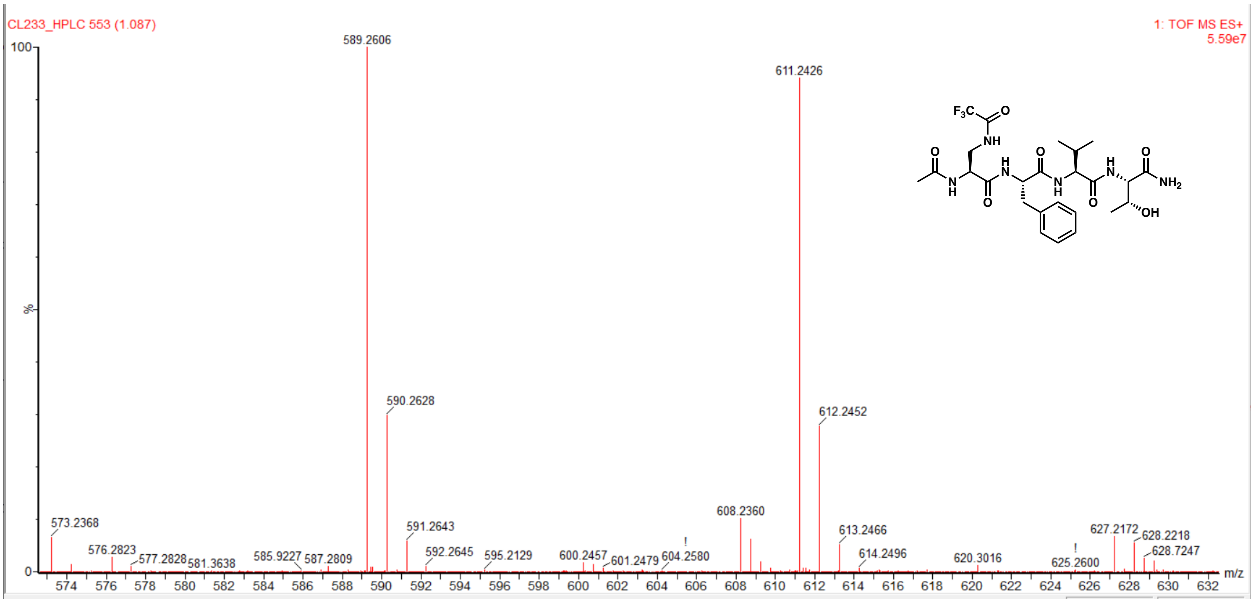
CL233_HPLC 553 (1.087)
1: TOF MS ES+
5.59e7
589.2606
611.2426
590.2628
612.2452
608.2360
573.2368
591.2643
613.2466
627.2172
628.2218
628.7247
m/z
%

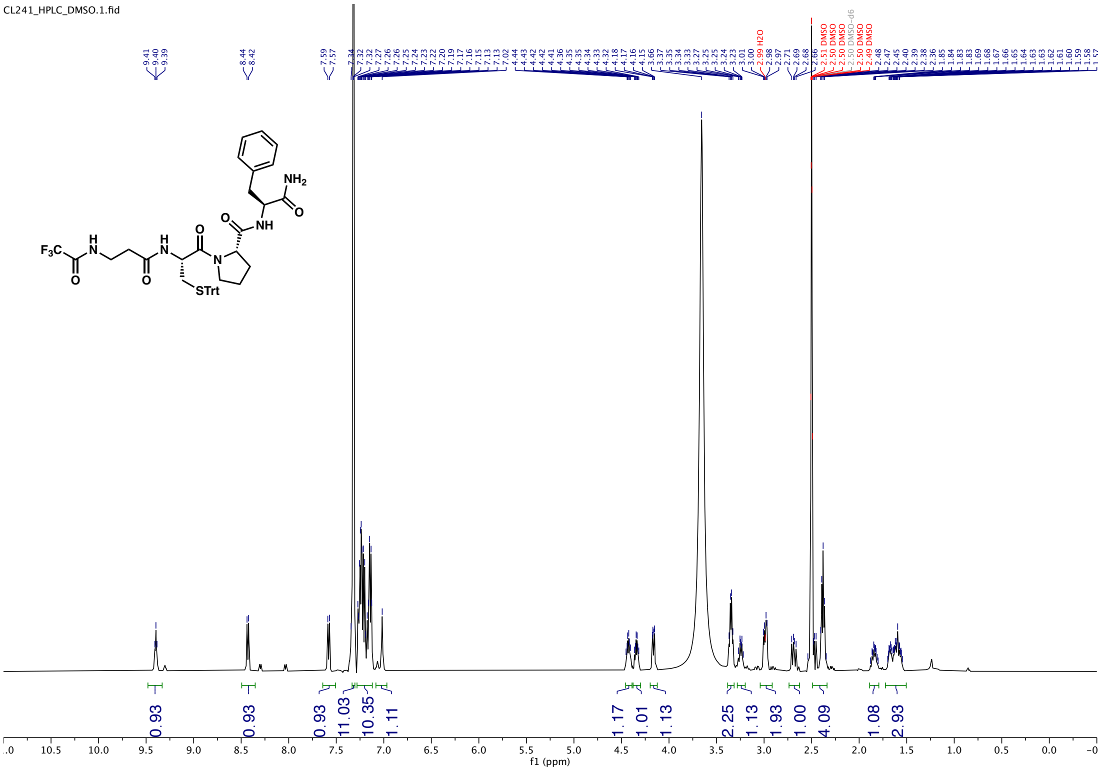
CL241_HPLC_DMSO.1.fid
f1 (ppm)

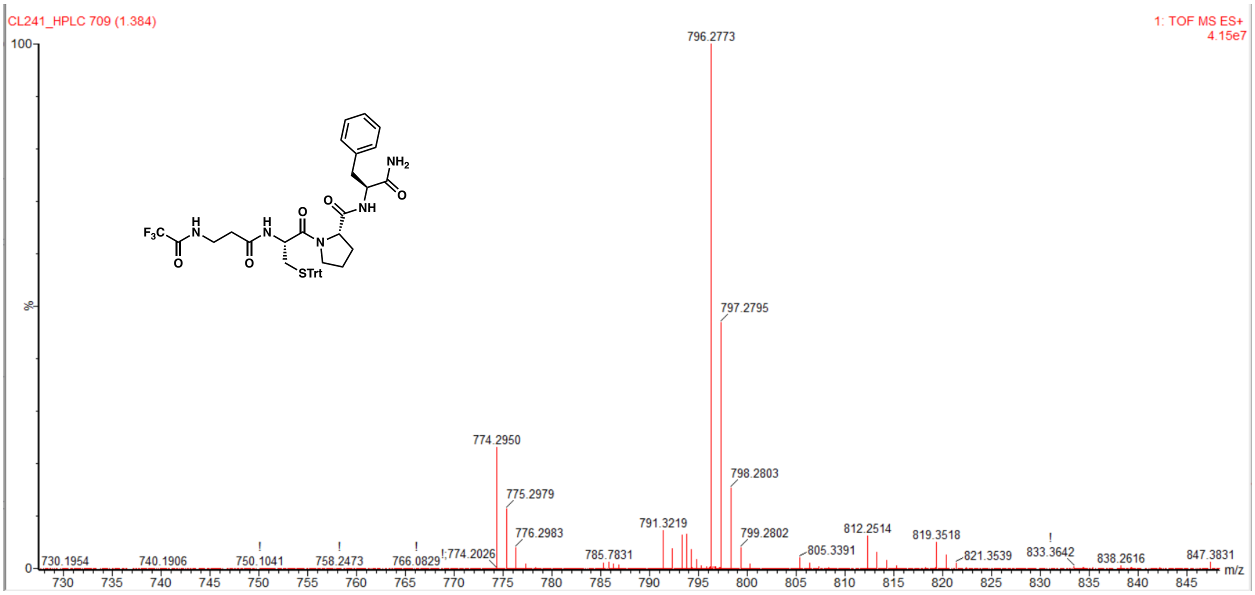
CL241_HPLC 709 (1.384)
1: TOF MS ES+
4.15e7
796.2773
797.2795
774.2950
798.2803
775.2979
m/z

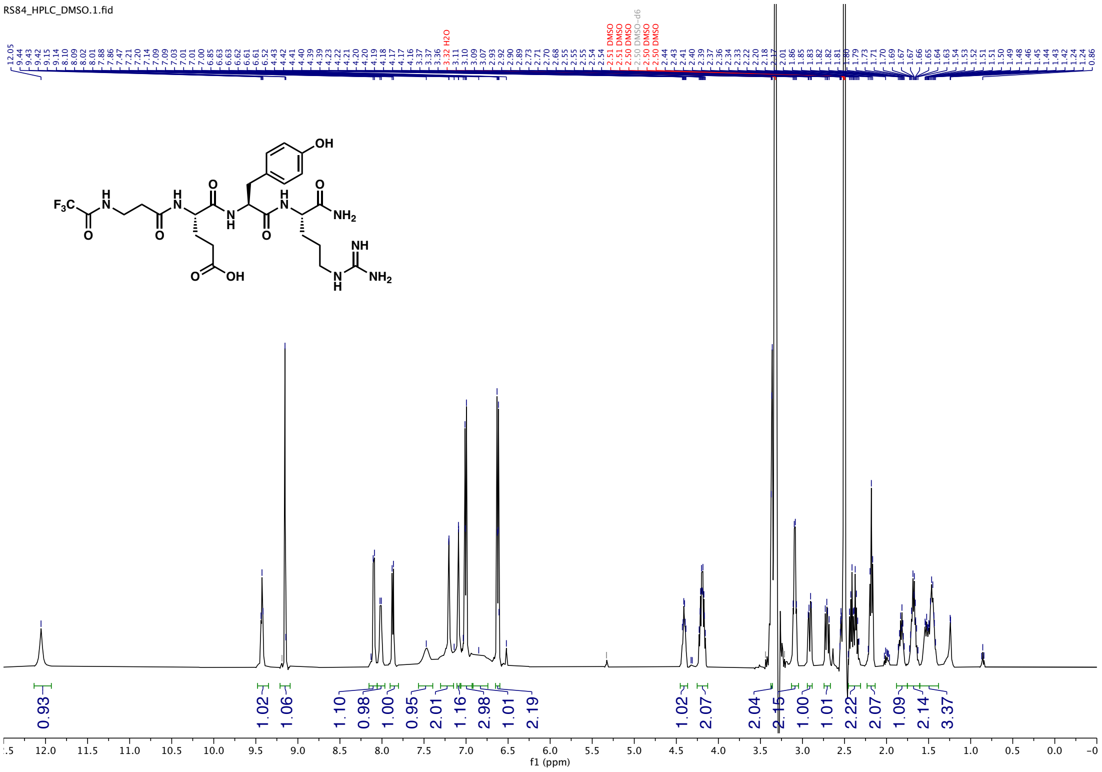

RS84_HPLC_DMSO.1.fid
F3C
NH2
OH
NH
0.93
1.02
1.06
1.10
0.98
1.00
0.95
2.01
1.16
2.98
1.01
2.19
1.02
2.07
2.04
2.15
1.00
1.01
2.22
2.07
1.09
2.14
3.37
f1 (ppm)

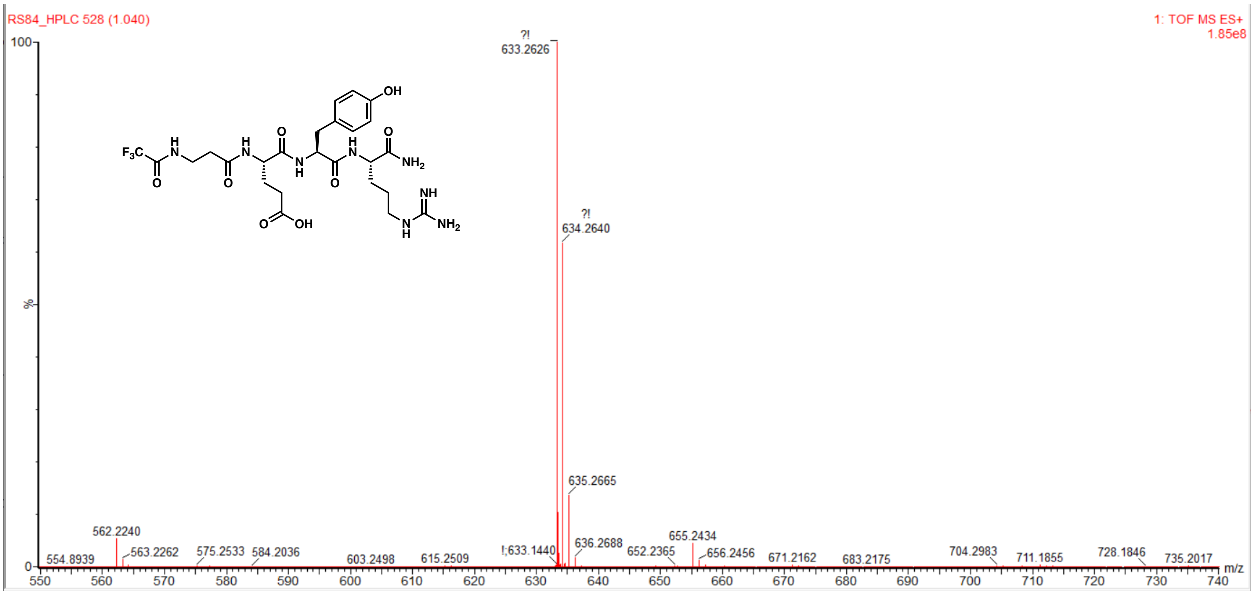

RS84_HPLC 528 (1.040)
1: TOF MS ES+
1.85e8
633.2626
634.2640
635.2665
562.2240
655.2434
%
m/z

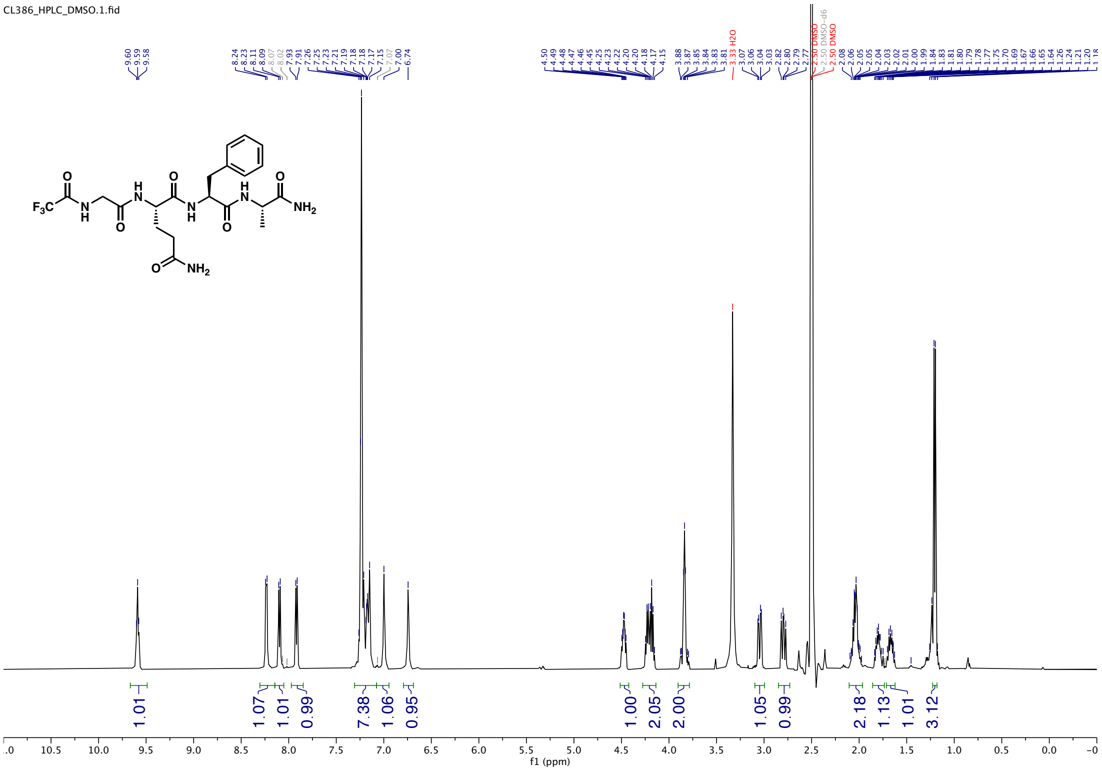
CL386_HPLC_DMSO.1.fid
f1 (ppm)
1.01
1.07
1.01
0.99
7.38
1.06
0.95
1.00
2.05
2.00
1.05
0.99
2.18
1.13
1.01
3.12
3.33 H2O
2.50 DMSO
NH2
F3C

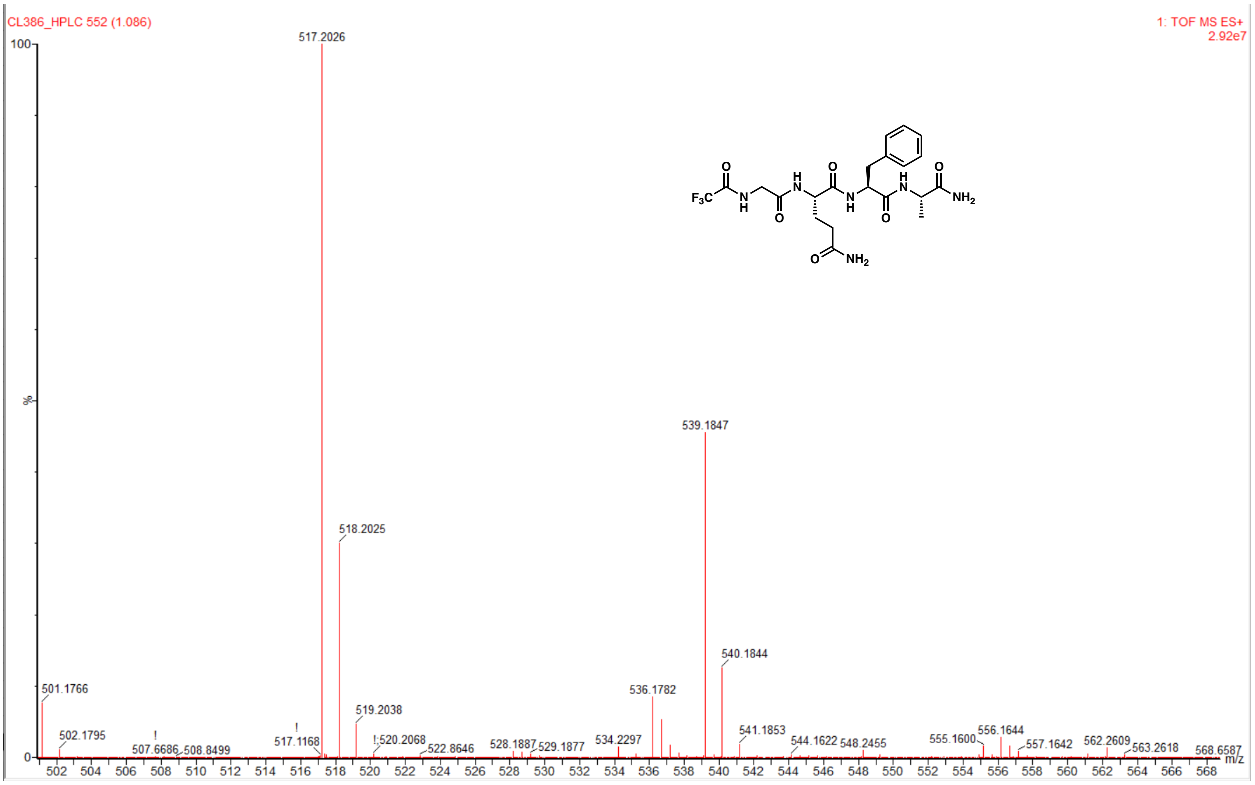
CL386_HPLC 552 (1.086)
1: TOF MS ES+
2.92e7
517.2026
539.1847
518.2025
540.1844
536.1782
501.1766
519.2038
541.1853
502.1795
556.1644
m/z
%
NH2
F3C

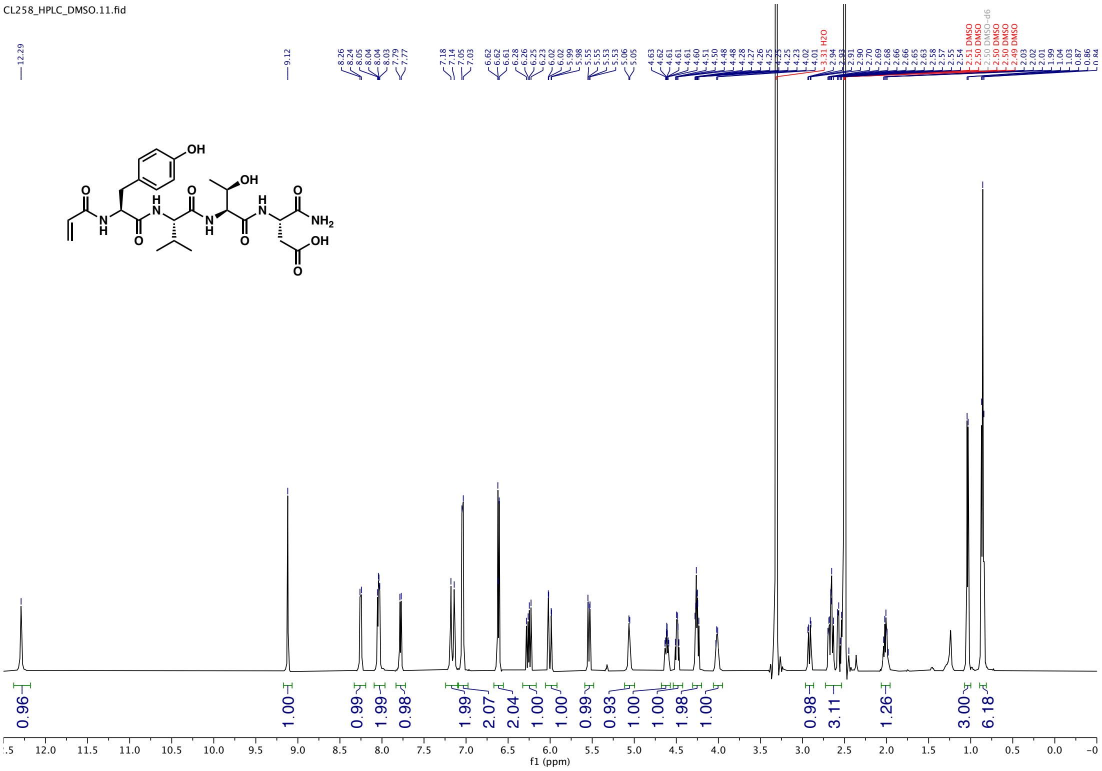
CL258_HPLC_DMSO.11.fid
f1 (ppm)

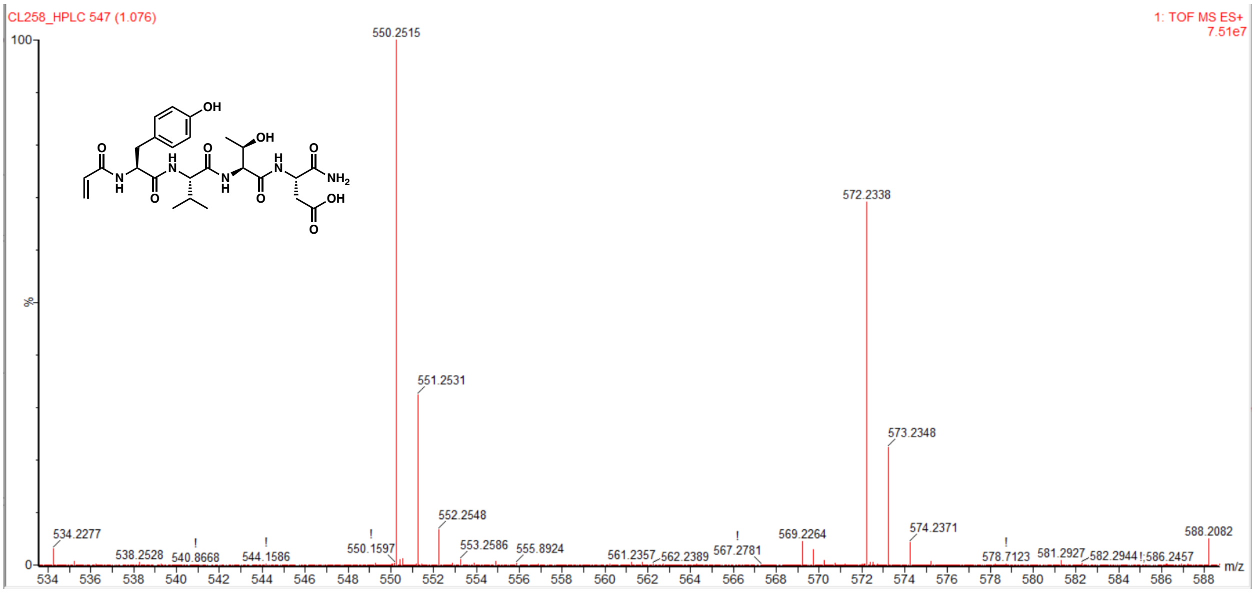
CL258_HPLC 547 (1.076)
1: TOF MS ES+
7.51e7
550.2515
572.2338
551.2531
573.2348
m/z

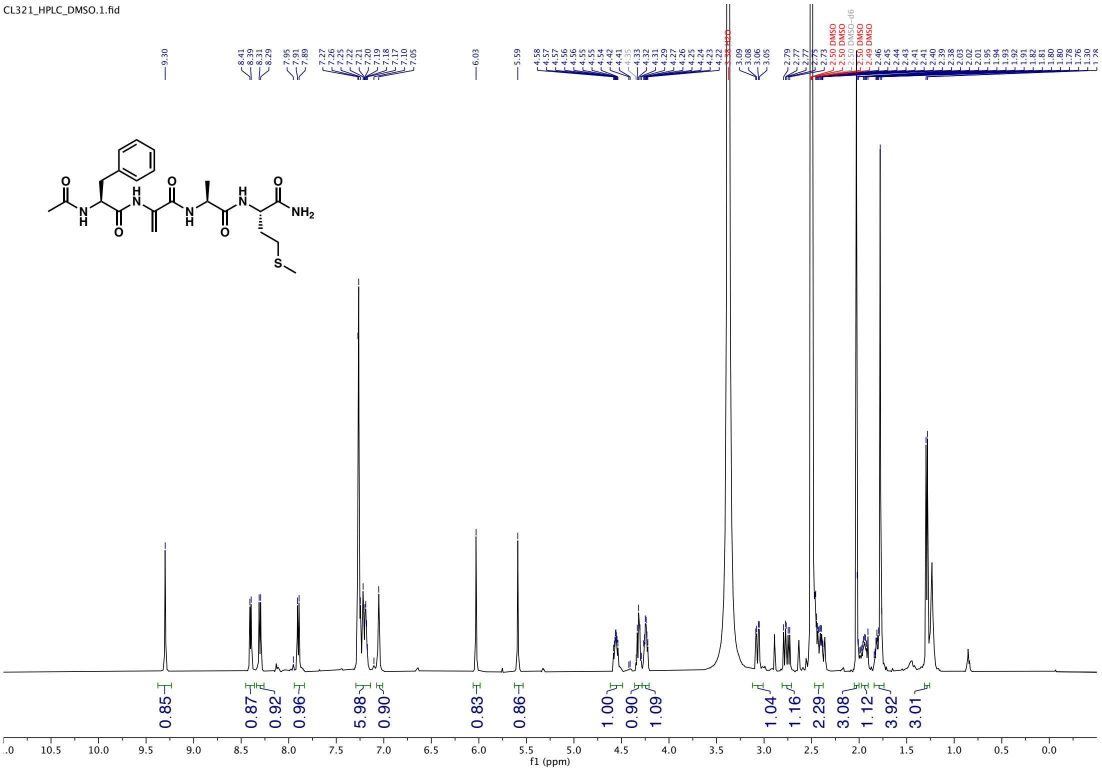
CL321_HPLC_DMSO.1.fid
0.85
0.87
0.92
0.96
5.98
0.90
0.83
0.86
1.00
0.90
1.09
1.04
1.16
2.29
3.08
1.12
3.92
3.01
f1 (ppm)

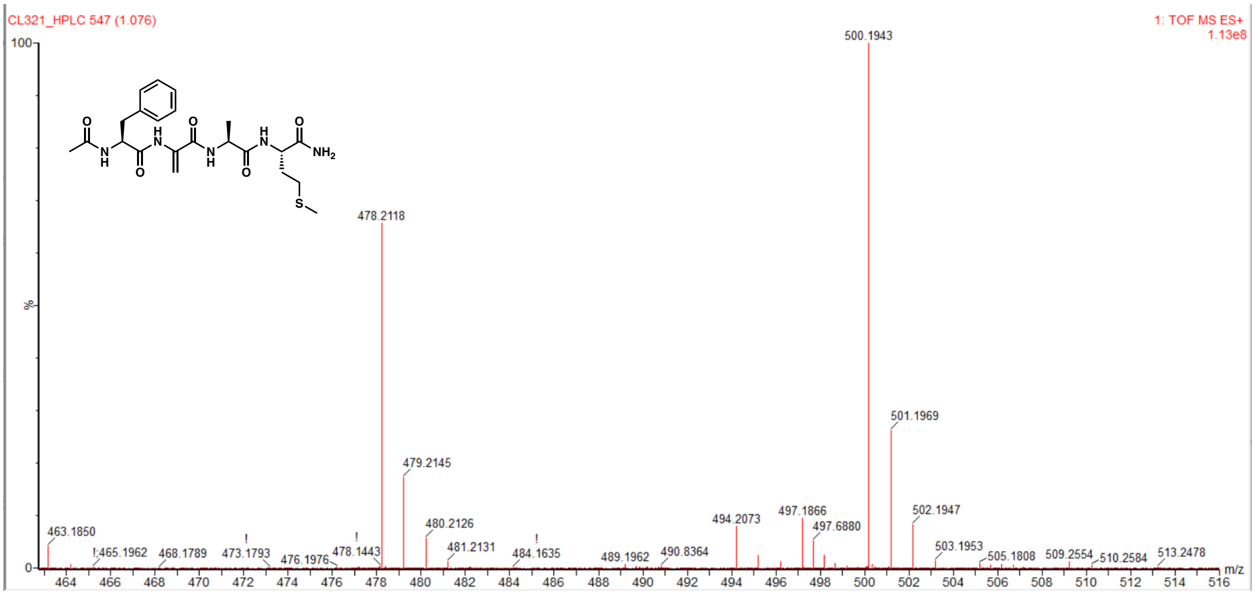
CL321_HPLC 547 (1.076)
1: TOF MS ES+
1.13e8
500.1943
478.2118
501.1969
479.2145
502.1947
497.1866
494.2073
497.6880
480.2126
463.1850
503.1953
481.2131
505.1808
509.2554
510.2584
513.2478
465.1962
468.1789
473.1793
476.1976
478.1443
484.1635
489.1962
490.8364
%
m/z

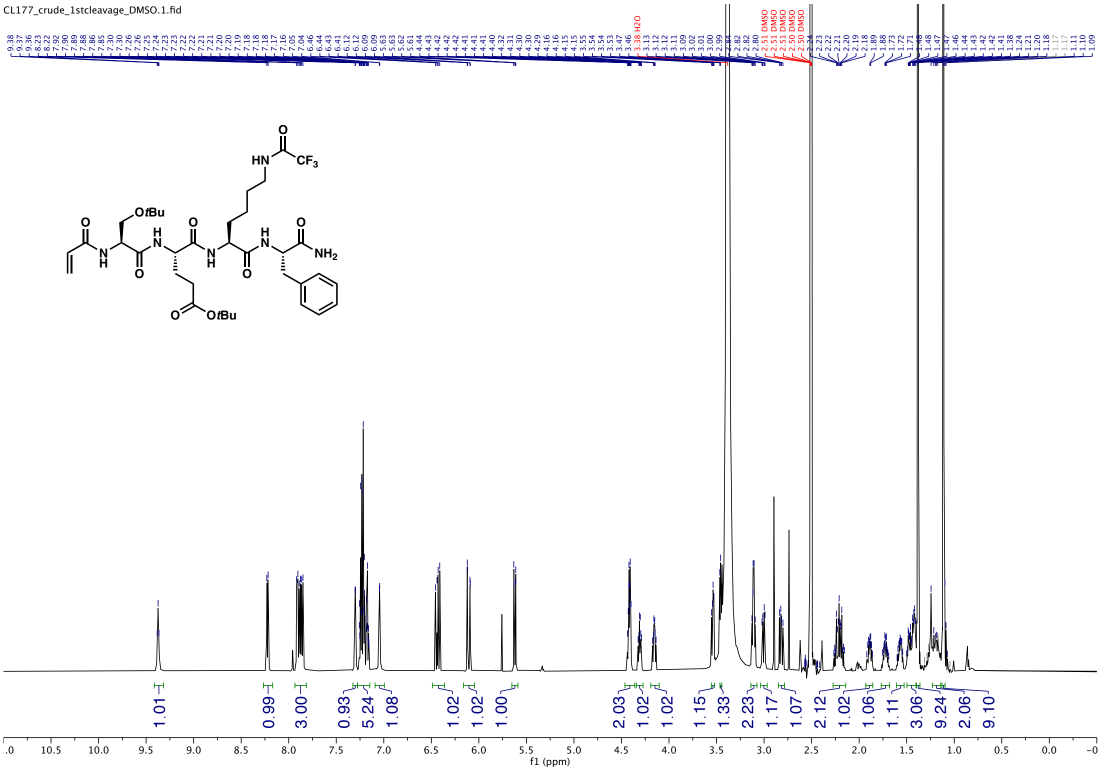
CL177_crude_1stcleavage_DMSO.1.fid
HN CF3 O
OtBu
NH2
OtBu
1.01
0.99
3.00
0.93
5.24
1.08
1.02
1.02
1.00
2.03
1.02
1.02
1.15
1.33
2.23
1.17
1.07
2.12
1.02
1.06
1.11
3.06
9.24
2.06
9.10
f1 (ppm)

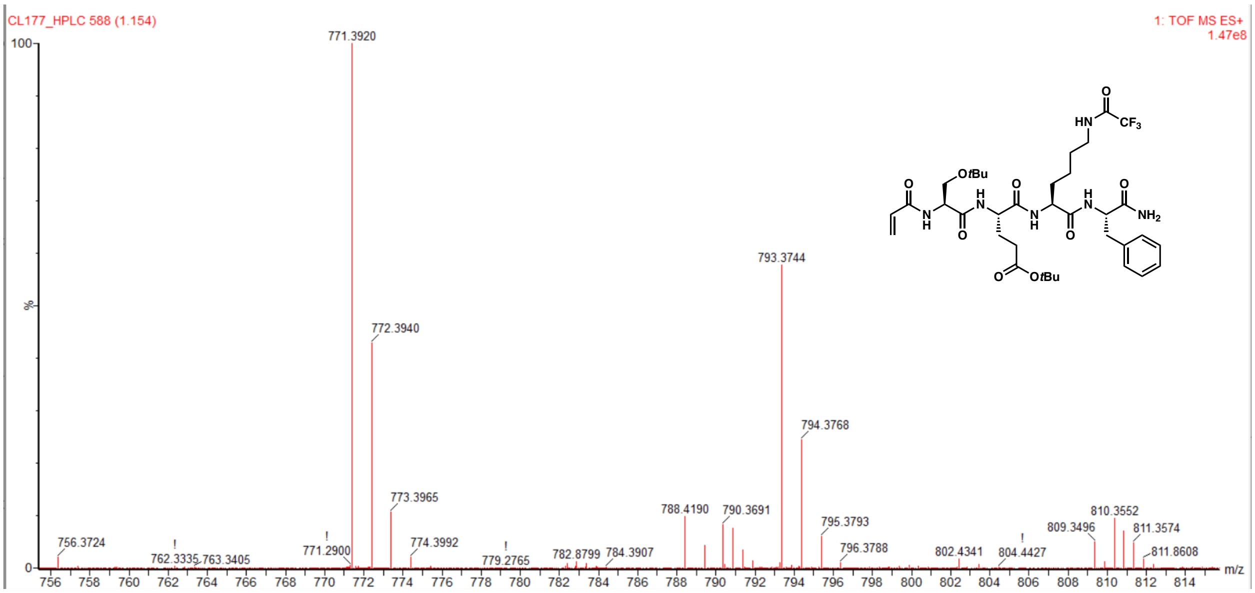
CL177_HPLC 588 (1.154)
1: TOF MS ES+
1.47e8
%
771.3920
772.3940
773.3965
774.3992
771.2900
756.3724
762.3335
763.3405
779.2765
782.8799
784.3907
788.4190
790.3691
793.3744
794.3768
795.3793
796.3788
802.4341
804.4427
809.3496
810.3552
811.3574
811.8608
m/z

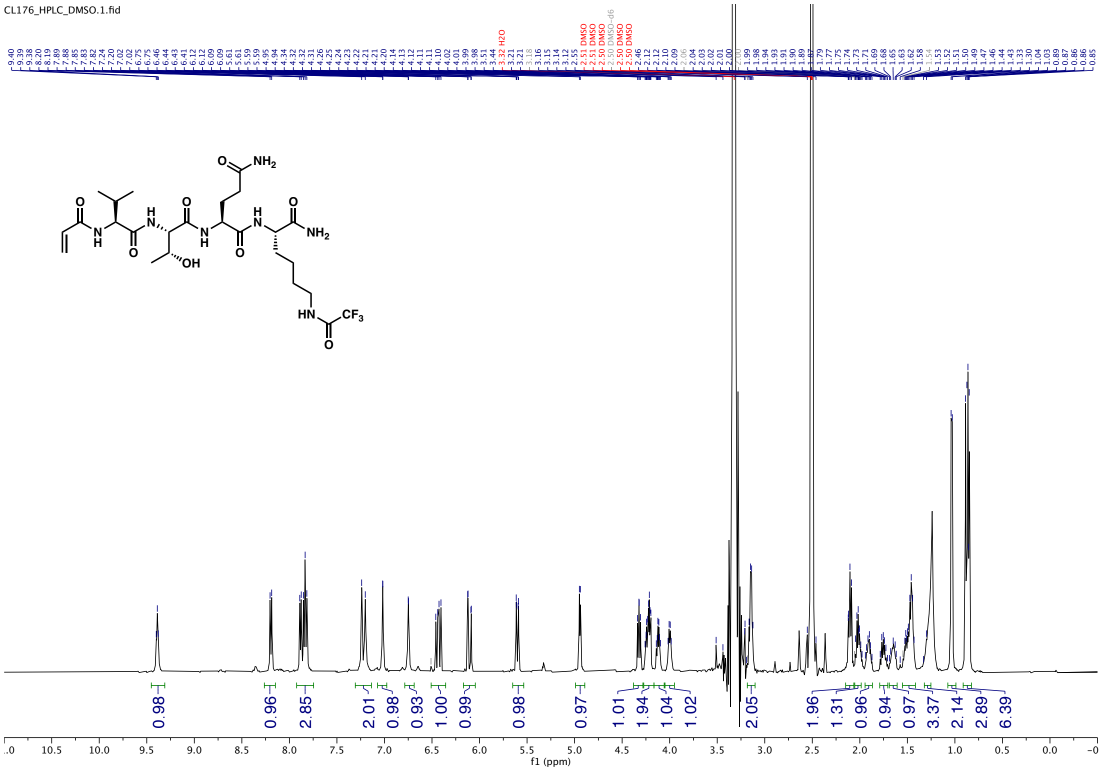
CL176_HPLC_DMSO.1.fid
f1 (ppm)

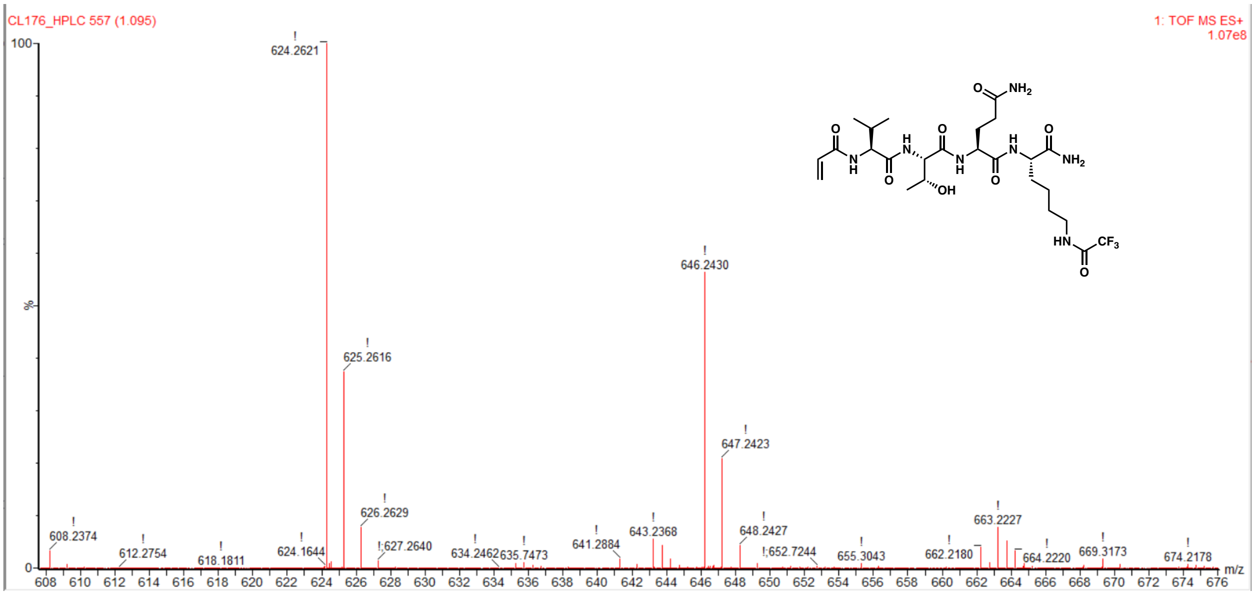
CL176_HPLC 557 (1.095)
1: TOF MS ES+
1.07e8
624.2621
646.2430
625.2616
647.2423
626.2629
608.2374
663.2227
643.2368
648.2427
m/z

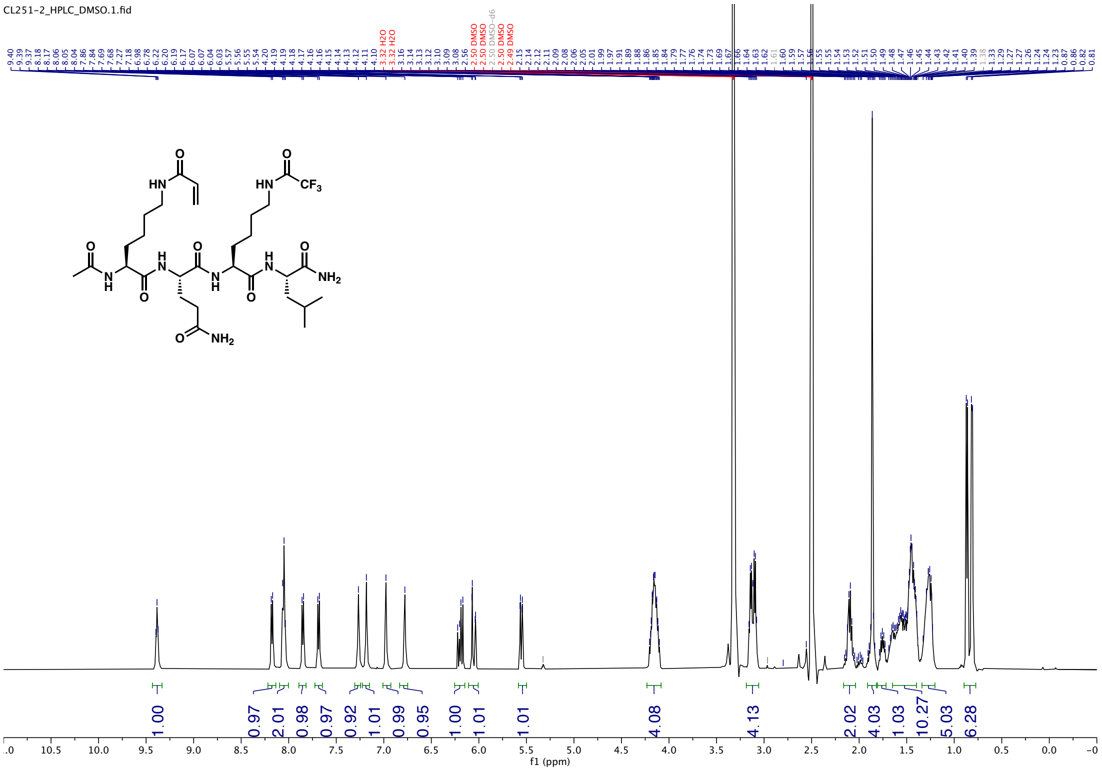
CL251-2_HPLC_DMSO.1.fid
HN
O
HN
O
CF3
O
N
H
O
H
N
O
N
H
O
H
N
O
NH2
O
NH2
1.00
0.97
2.01
0.98
0.97
0.92
1.01
0.99
0.95
1.00
1.01
1.01
4.08
4.13
2.02
4.03
1.03
10.27
5.03
6.28
f1 (ppm)

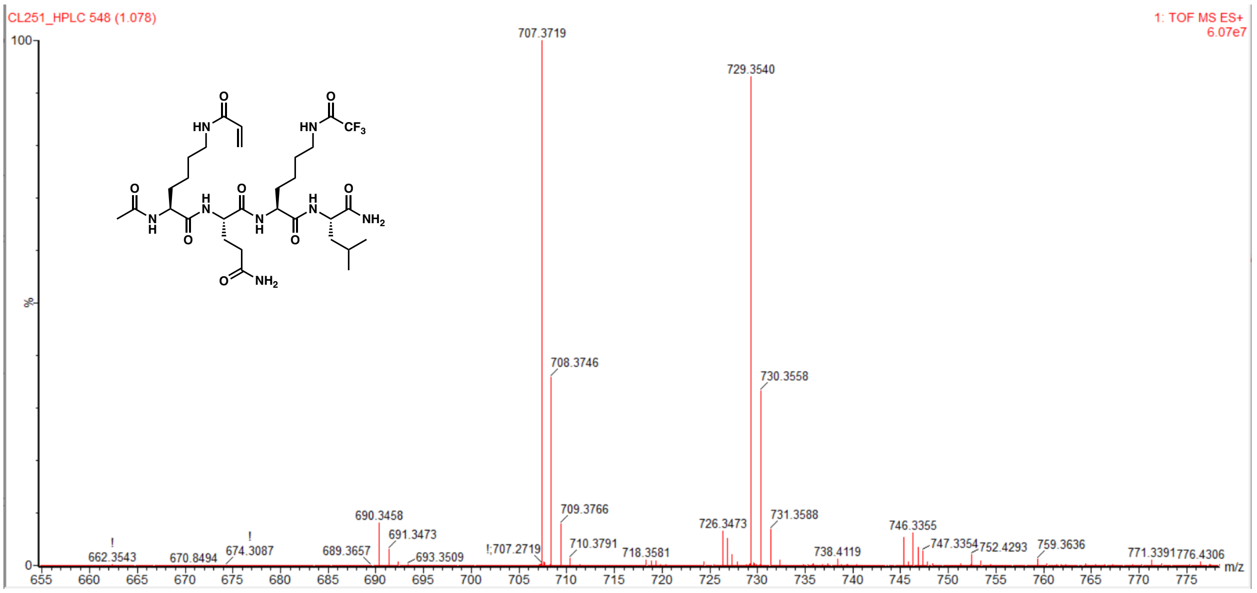
CL251_HPLC 548 (1.078)
1: TOF MS ES+
6.07e7
100
%
0
707.3719
729.3540
708.3746
730.3558
709.3766
726.3473
731.3588
690.3458
691.3473
746.3355
747.3354
752.4293
759.3636
771.3391
776.4306
662.3543
670.8494
674.3087
689.3657
693.3509
707.2719
710.3791
718.3581
738.4119
m/z

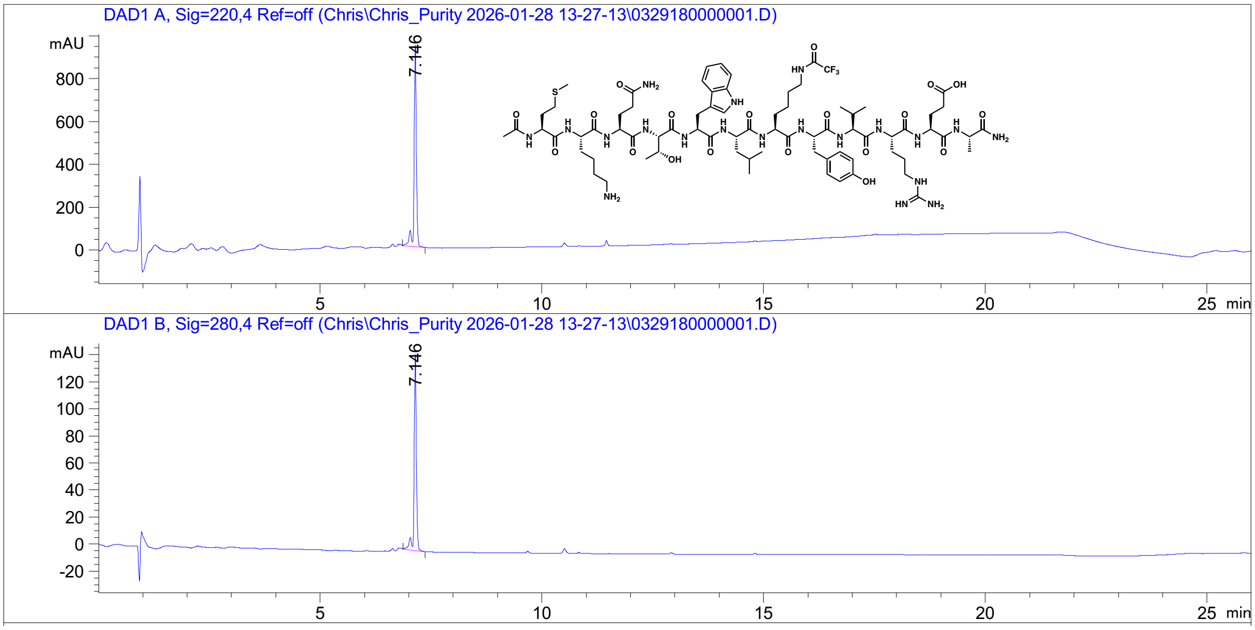

DAD1 A, Sig=220,4 Ref=off (Chris\Chris_Purity 2026-01-28 13-27-13\0329180000001.D)
mAU
7.146
min
DAD1 B, Sig=280,4 Ref=off (Chris\Chris_Purity 2026-01-28 13-27-13\0329180000001.D)
mAU
7.146
min

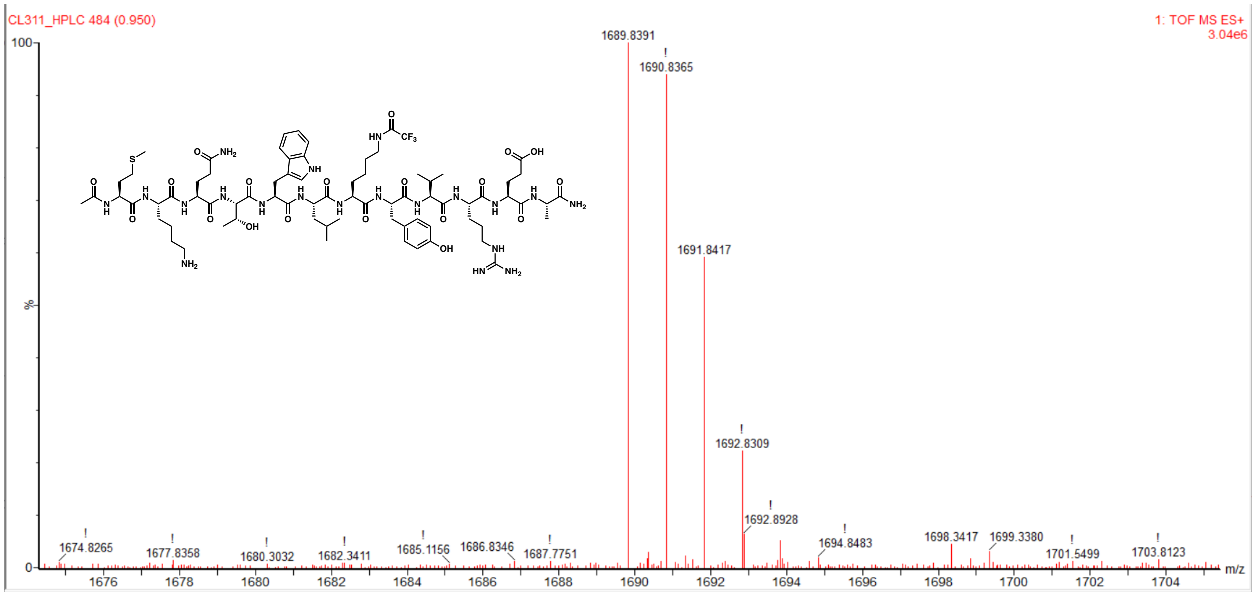

CL311_HPLC 484 (0.950)
1: TOF MS ES+
3.04e6
1689.8391
1690.8365
1691.8417
1692.8309
1692.8928
1694.8483
1698.3417
1699.3380
1701.5499
1703.8123
1674.8265
1677.8358
1680.3032
1682.3411
1685.1156
1686.8346
1687.7751
%
m/z

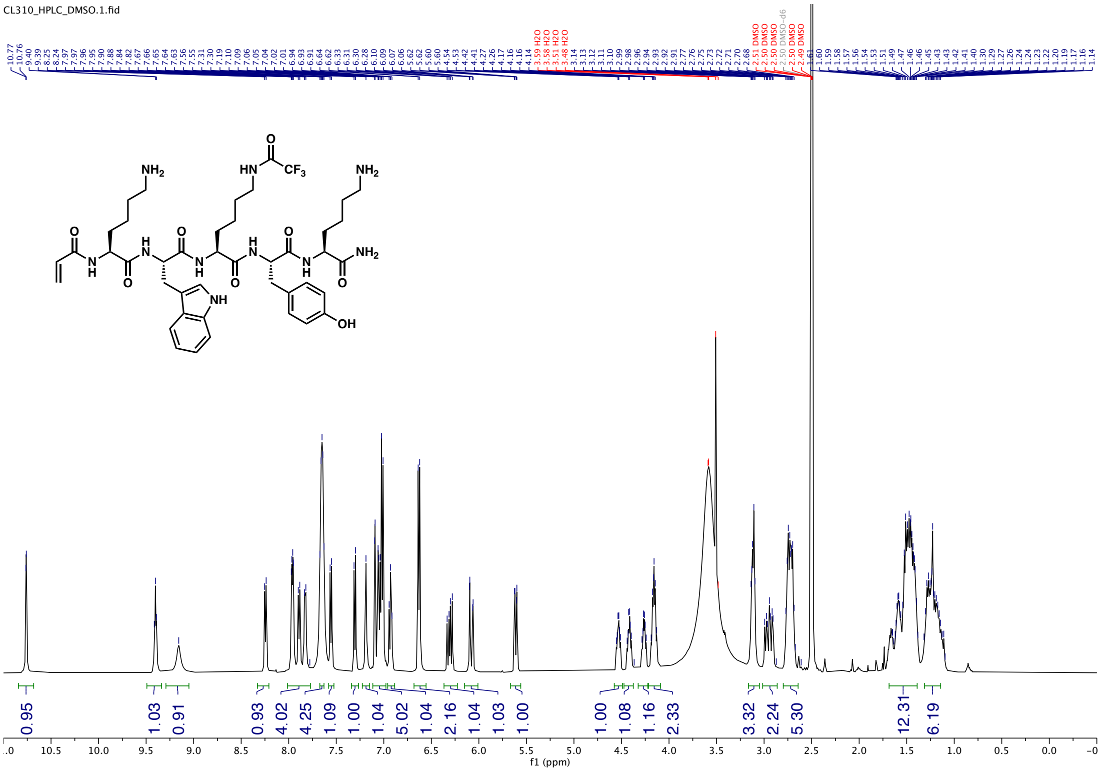
CL310_HPLC_DMSO.1.fid
f1 (ppm)

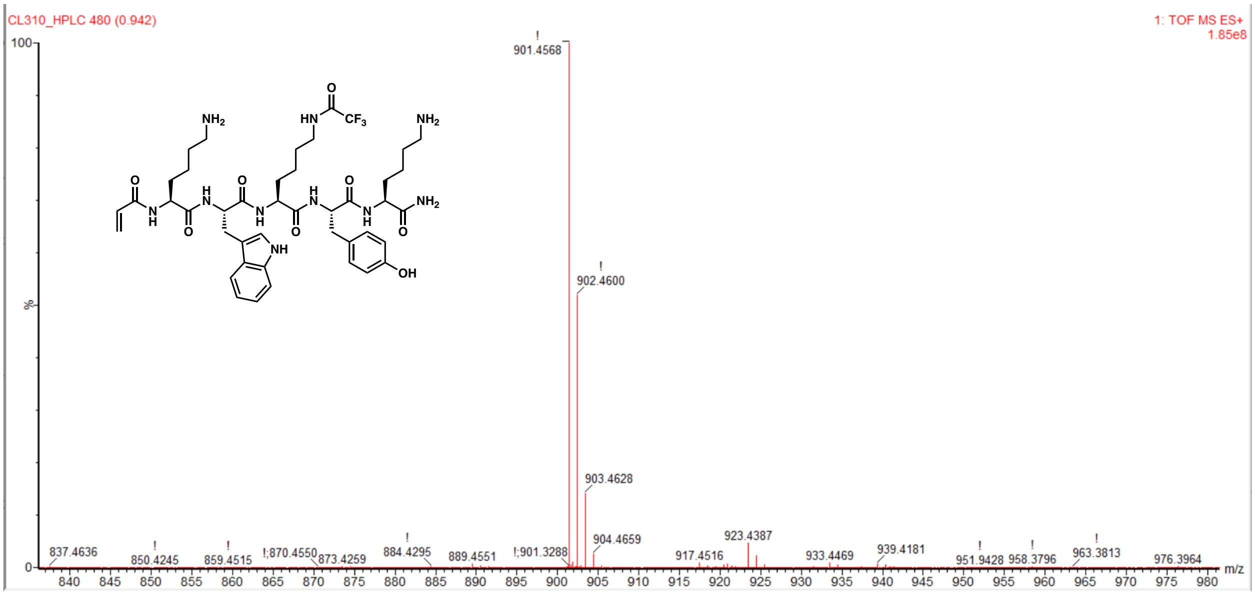
CL310_HPLC 480 (0.942)
1: TOF MS ES+
1.85e8
901.4568
902.4600
903.4628
904.4659
923.4387
939.4181
963.3813
976.3964
m/z

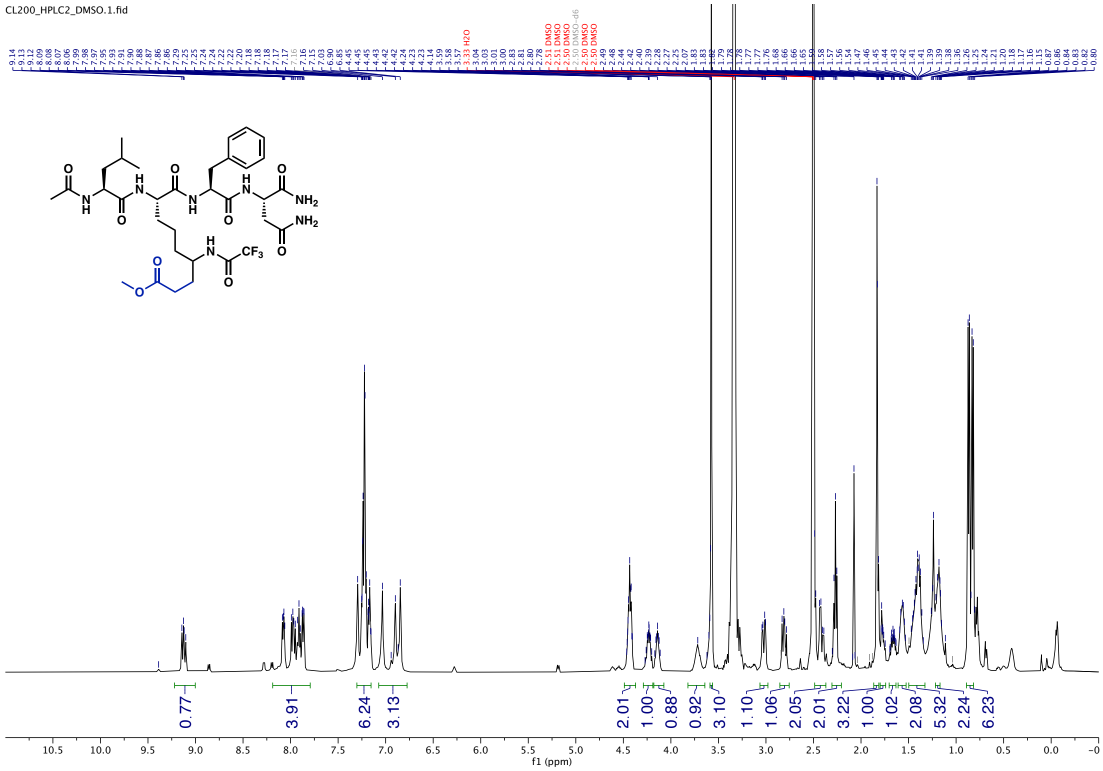
CL200_HPLC2_DMSO.1.fid
NH2
NH2
CF3
0.77
3.91
6.24
3.13
2.01
1.00
0.88
0.92
3.10
1.10
1.06
2.05
2.01
3.22
1.00
1.02
2.08
5.32
2.24
6.23
f1 (ppm)

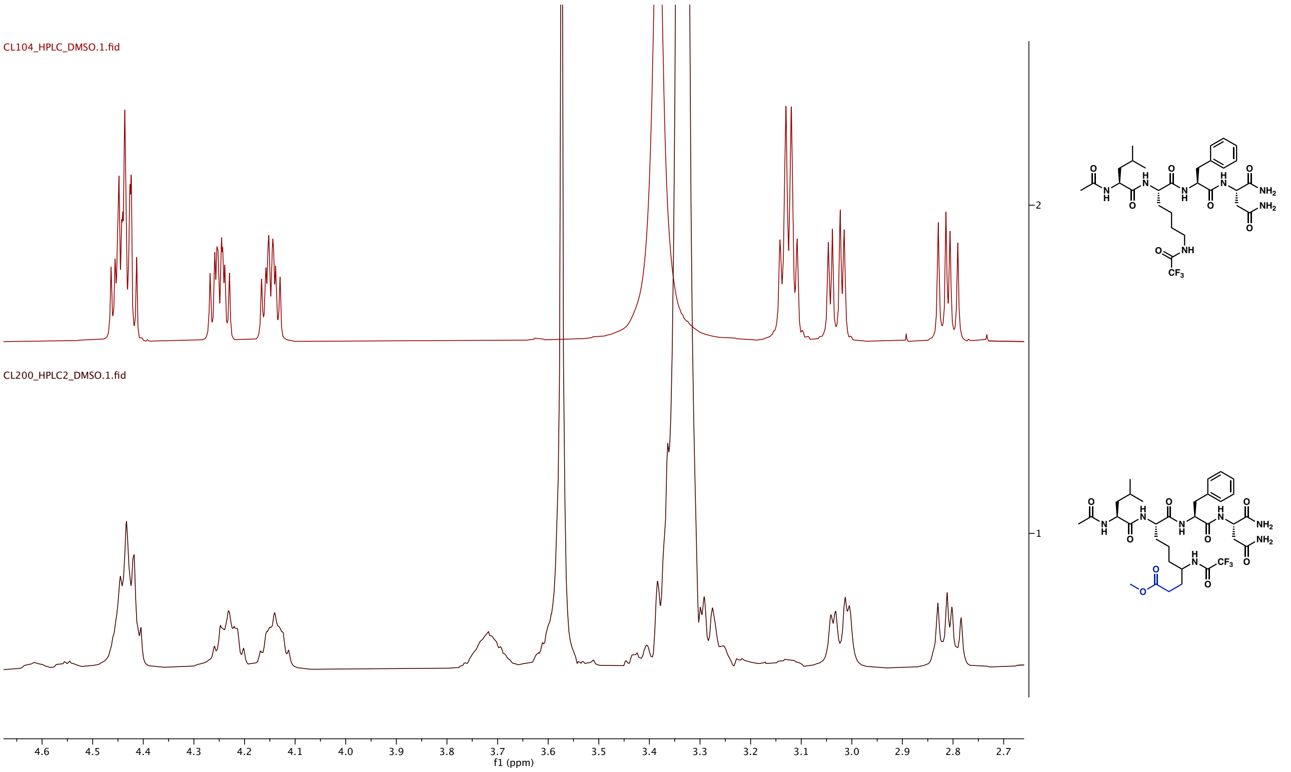
CL104_HPLC_DMSO.1.fid
CL200_HPLC2_DMSO.1.fid
NH2
NH2
CF3
f1 (ppm)

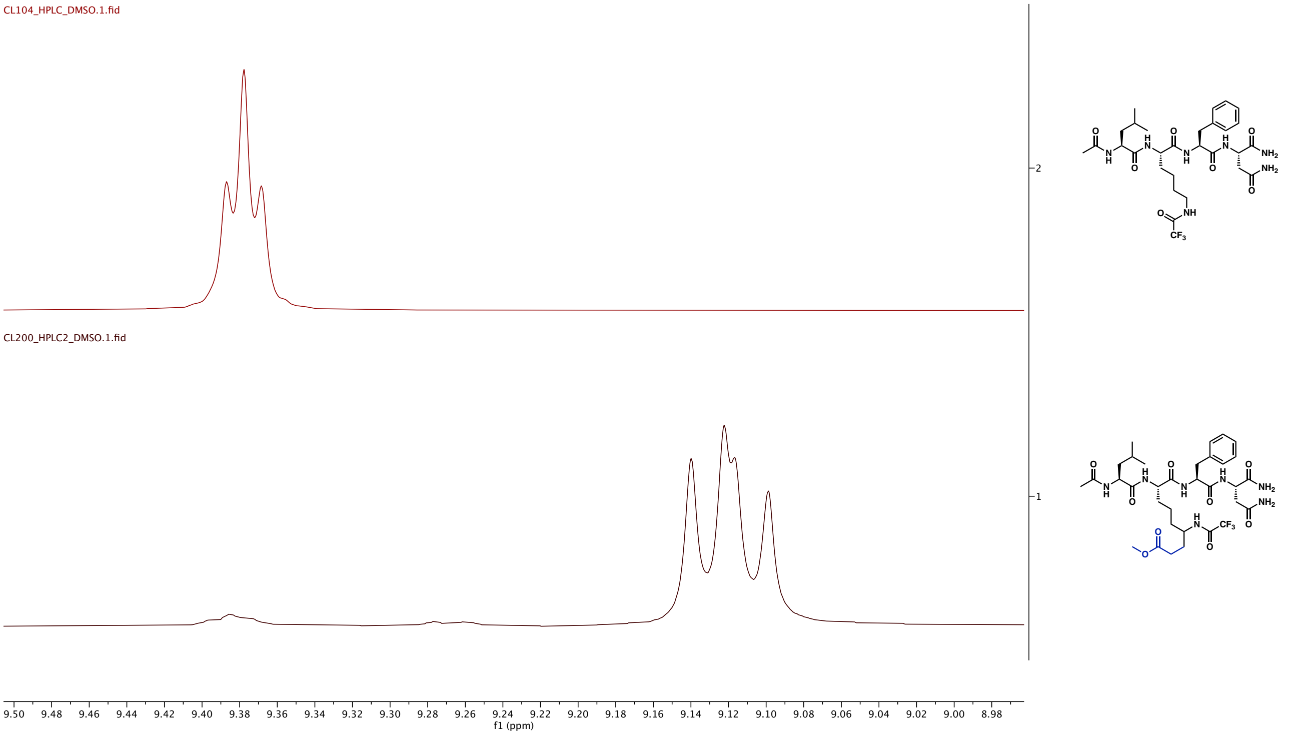

CL104_HPLC_DMSO.1.fid
CL200_HPLC2_DMSO.1.fid
9.50 9.48 9.46 9.44 9.42 9.40 9.38 9.36 9.34 9.32 9.30 9.28 9.26 9.24 9.22 9.20 9.18 9.16 9.14 9.12 9.10 9.08 9.06 9.04 9.02 9.00 8.98
f1 (ppm)

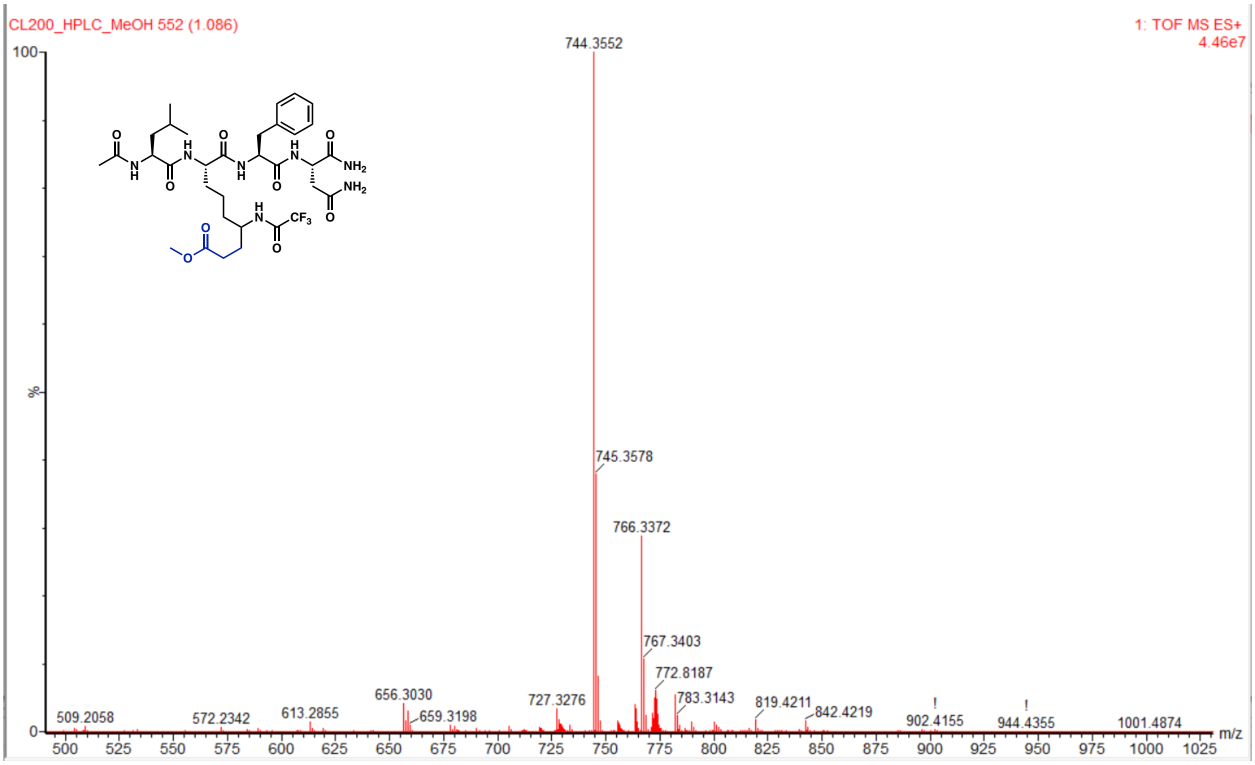

CL200_HPLC_MeOH 552 (1.086)
1: TOF MS ES+
4.46e7
744.3552
745.3578
766.3372
767.3403
772.8187
783.3143
819.4211
842.4219
902.4155
944.4355
1001.4874
727.3276
656.3030
659.3198
613.2855
572.2342
509.2058
m/z
%

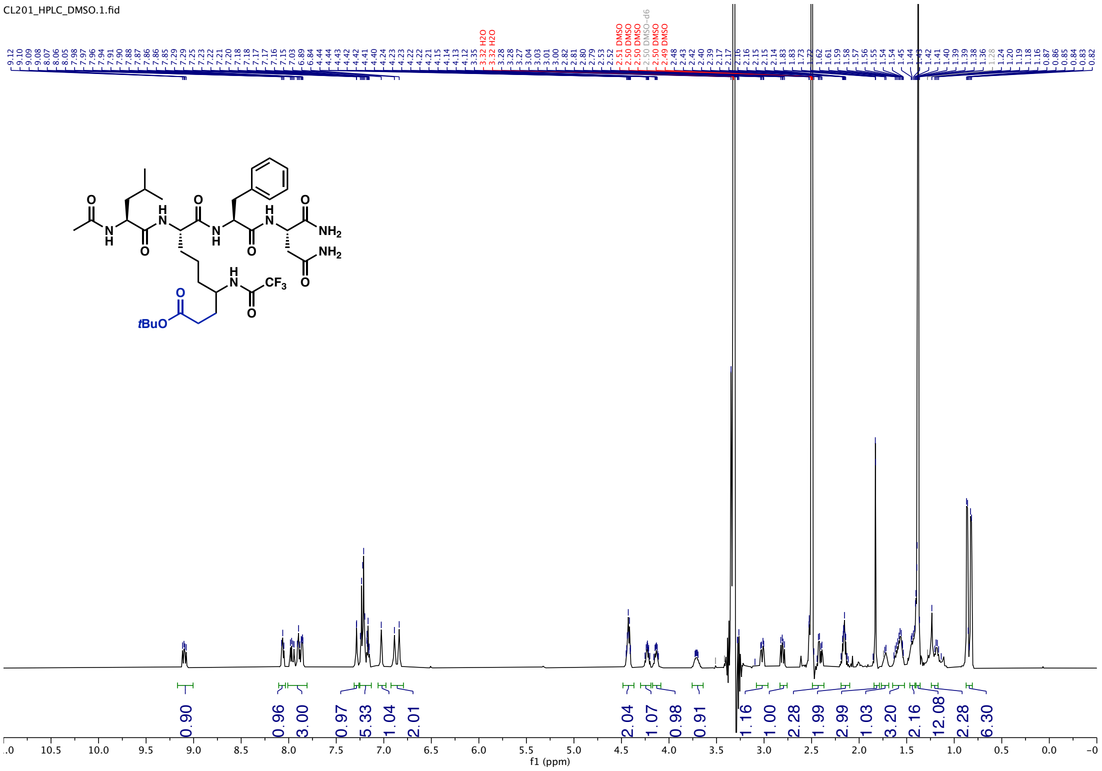
CL201_HPLC_DMSO.1.fid
tBuO
f1 (ppm)

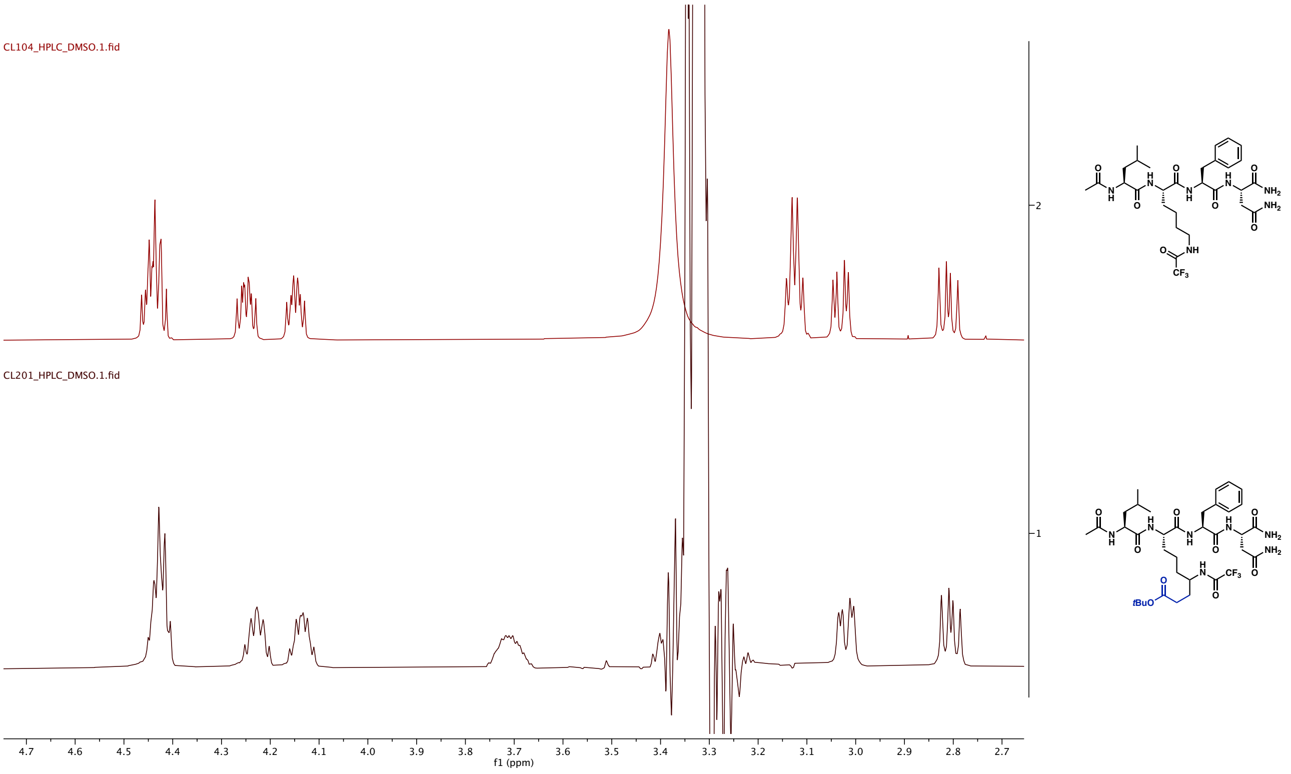
CL104_HPLC_DMSO.1.fid
CL201_HPLC_DMSO.1.fid
tBuO
f1 (ppm)

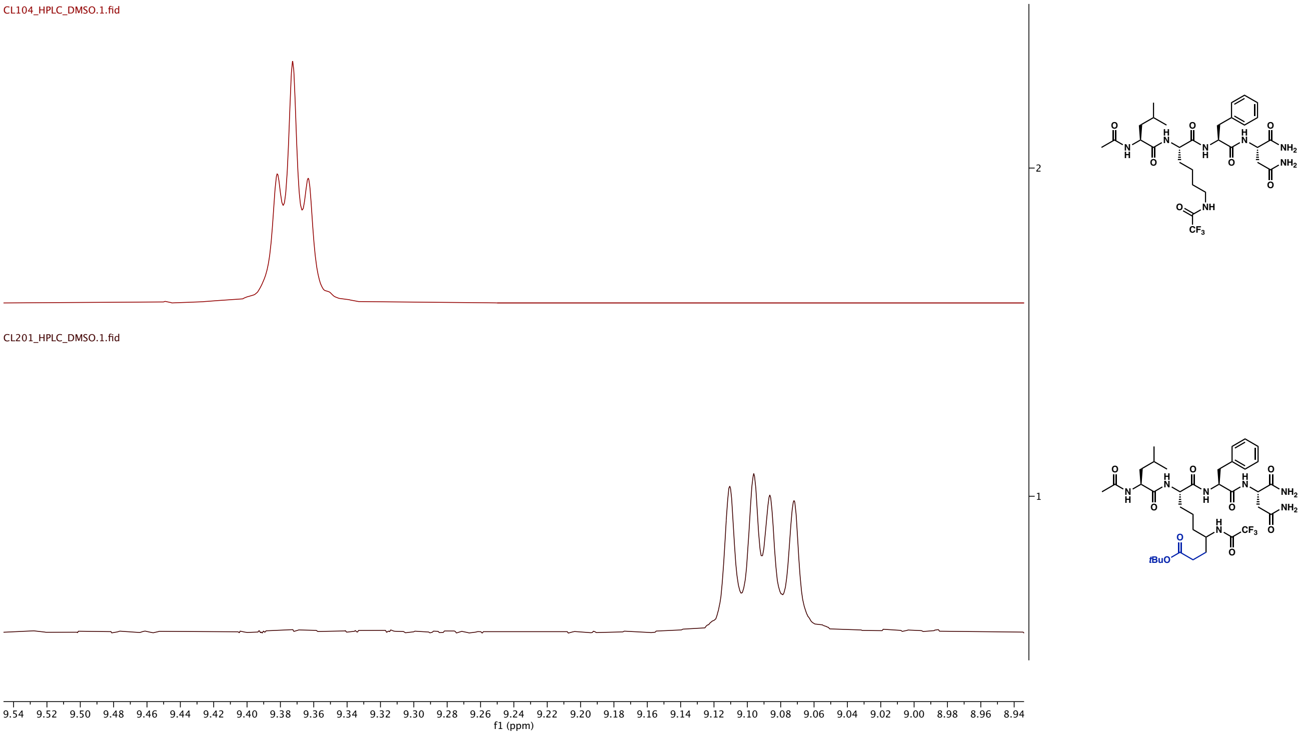
CL104_HPLC_DMSO.1.fid
CL201_HPLC_DMSO.1.fid
f1 (ppm)

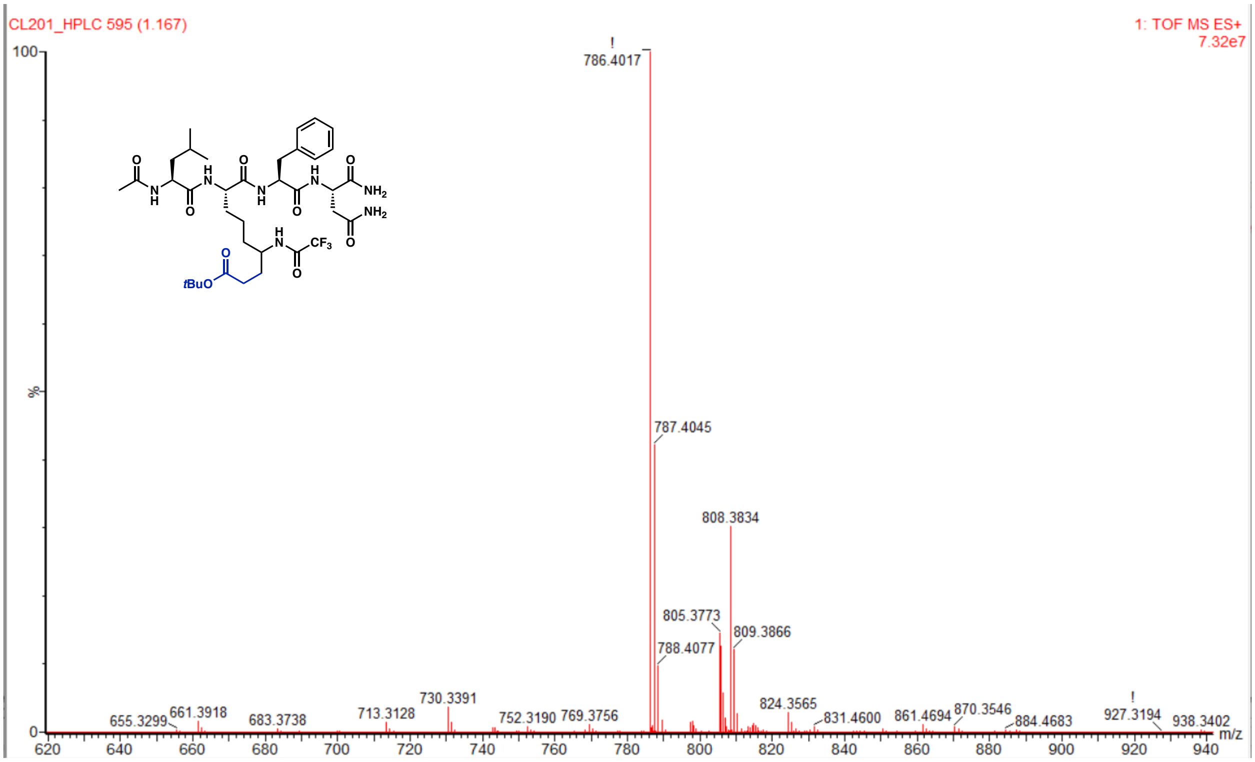
CL201_HPLC 595 (1.167)
1: TOF MS ES+
7.32e7
786.4017
787.4045
808.3834
805.3773
809.3866
788.4077
730.3391
824.3565
m/z

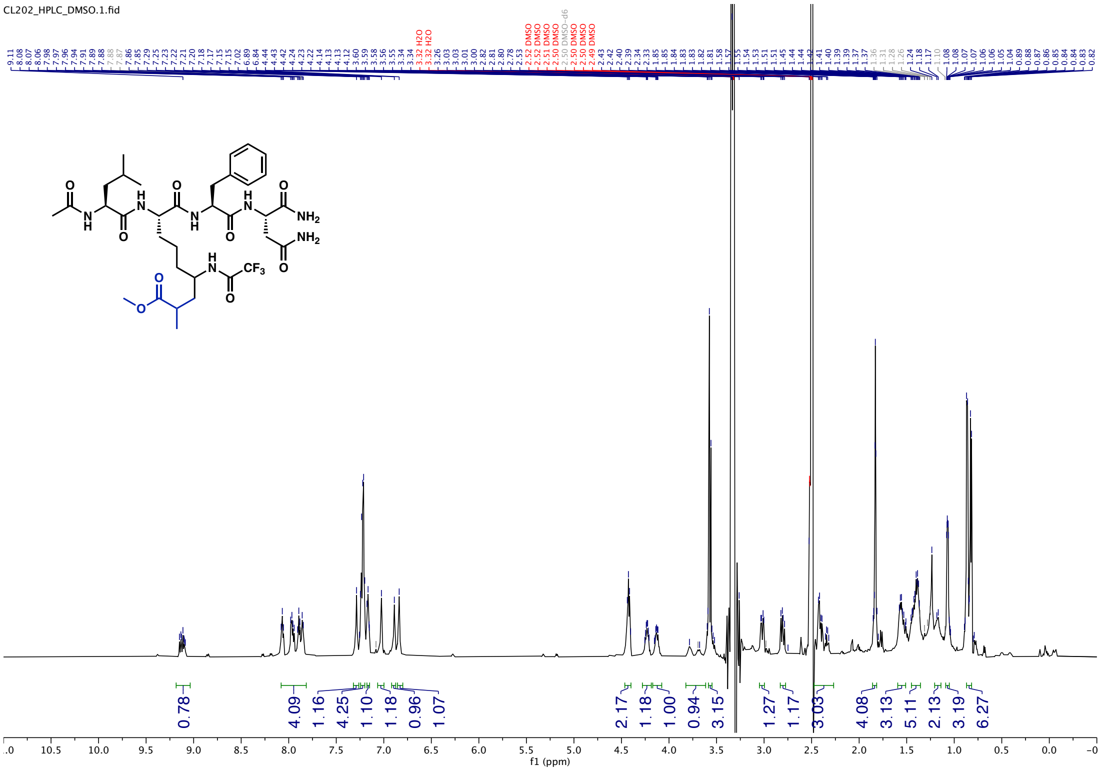

CL202_HPLC_DMSO.1.fid
NH2
NH2
CF3
0.78
4.09
1.16
4.25
1.10
1.18
0.96
1.07
2.17
1.18
1.00
0.94
3.15
1.27
1.17
3.03
4.08
3.13
5.11
2.13
3.19
6.27
f1 (ppm)

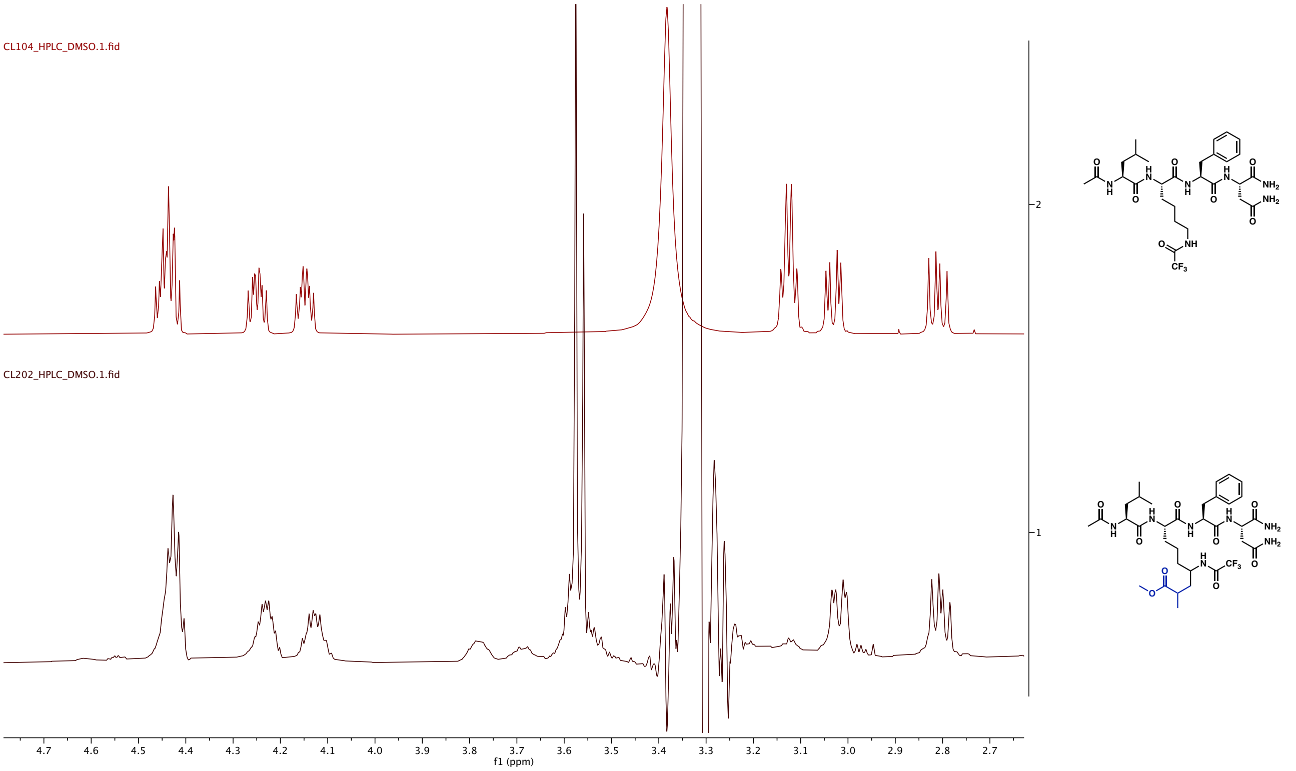

CL104_HPLC_DMSO.1.fid
CL202_HPLC_DMSO.1.fid
f1 (ppm)

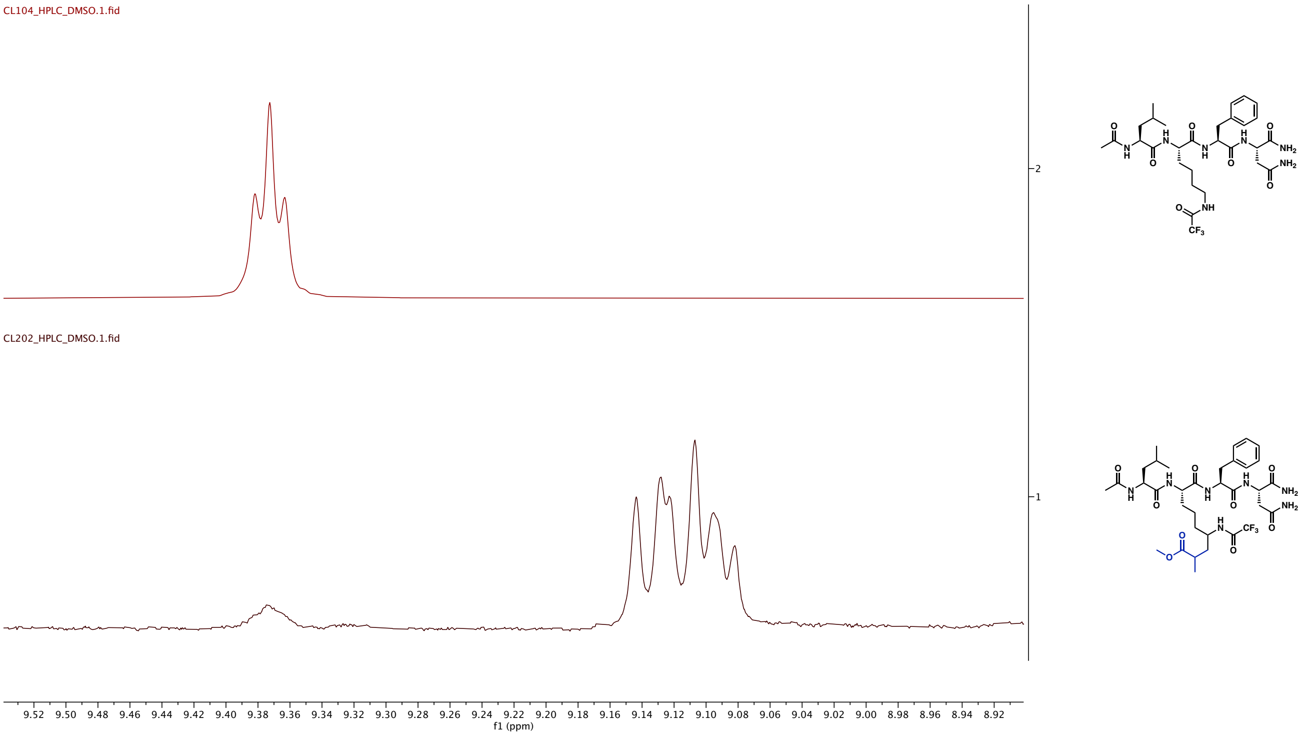

CL104_HPLC_DMSO.1.fid
CL202_HPLC_DMSO.1.fid
9.52 9.50 9.48 9.46 9.44 9.42 9.40 9.38 9.36 9.34 9.32 9.30 9.28 9.26 9.24 9.22 9.20 9.18 9.16 9.14 9.12 9.10 9.08 9.06 9.04 9.02 9.00 8.98 8.96 8.94 8.92
f1 (ppm)
2
1

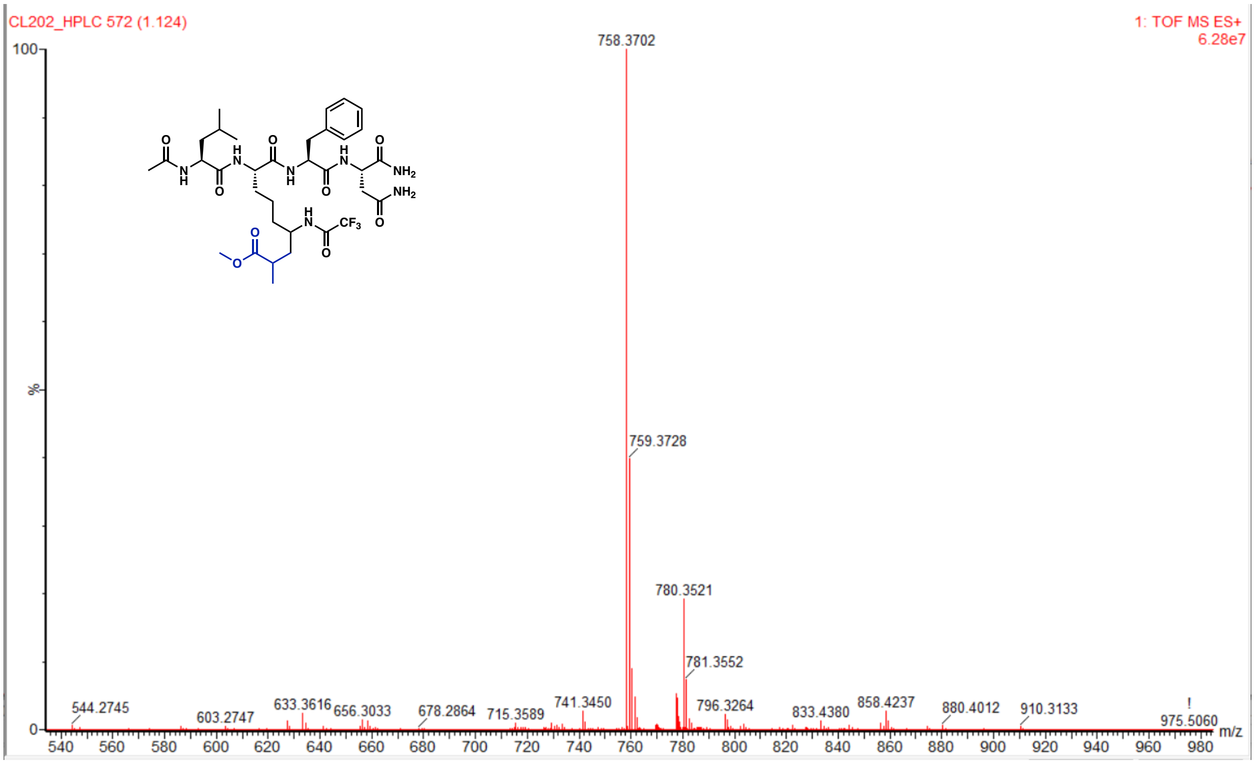

CL202_HPLC 572 (1.124)
1: TOF MS ES+
6.28e7
100
%
0
758.3702
759.3728
780.3521
781.3552
544.2745
603.2747
633.3616
656.3033
678.2864
715.3589
741.3450
796.3264
833.4380
858.4237
880.4012
910.3133
975.5060
540 560 580 600 620 640 660 680 700 720 740 760 780 800 820 840 860 880 900 920 940 960 980
m/z

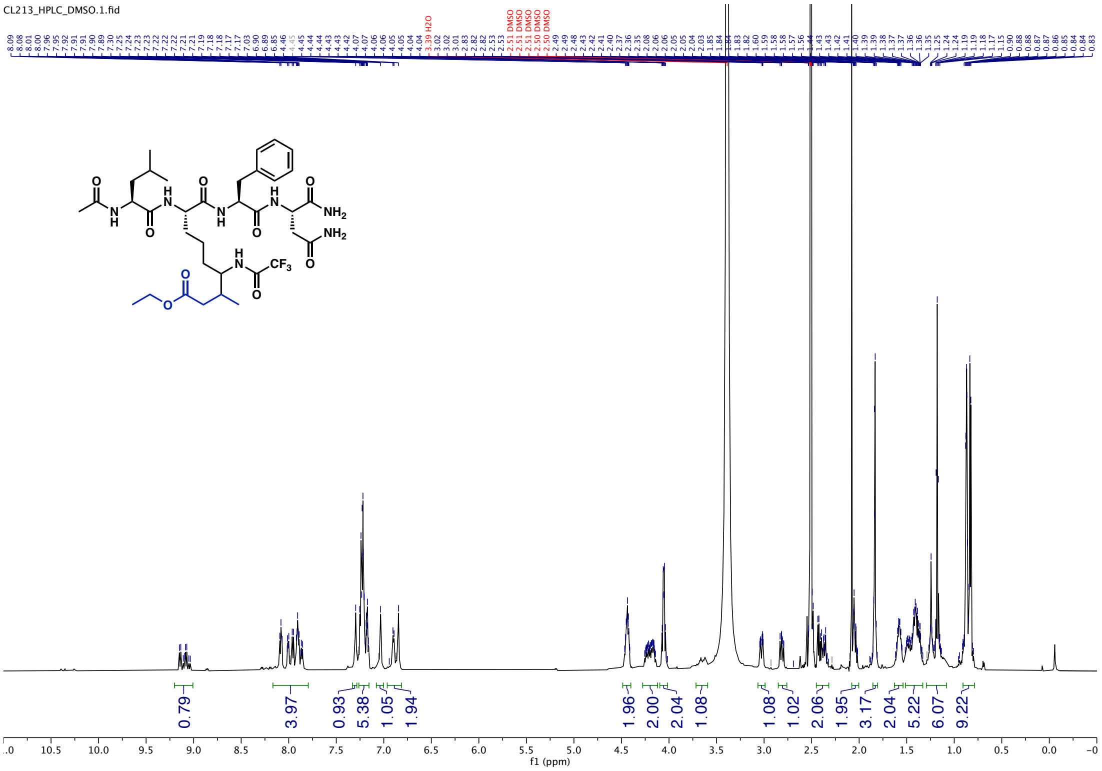
CL213_HPLC_DMSO.1.fid
0.79
3.97
0.93
5.38
1.05
1.94
1.96
2.00
2.04
1.08
1.08
1.02
2.06
1.95
3.17
2.04
5.22
6.07
9.22
f1 (ppm)

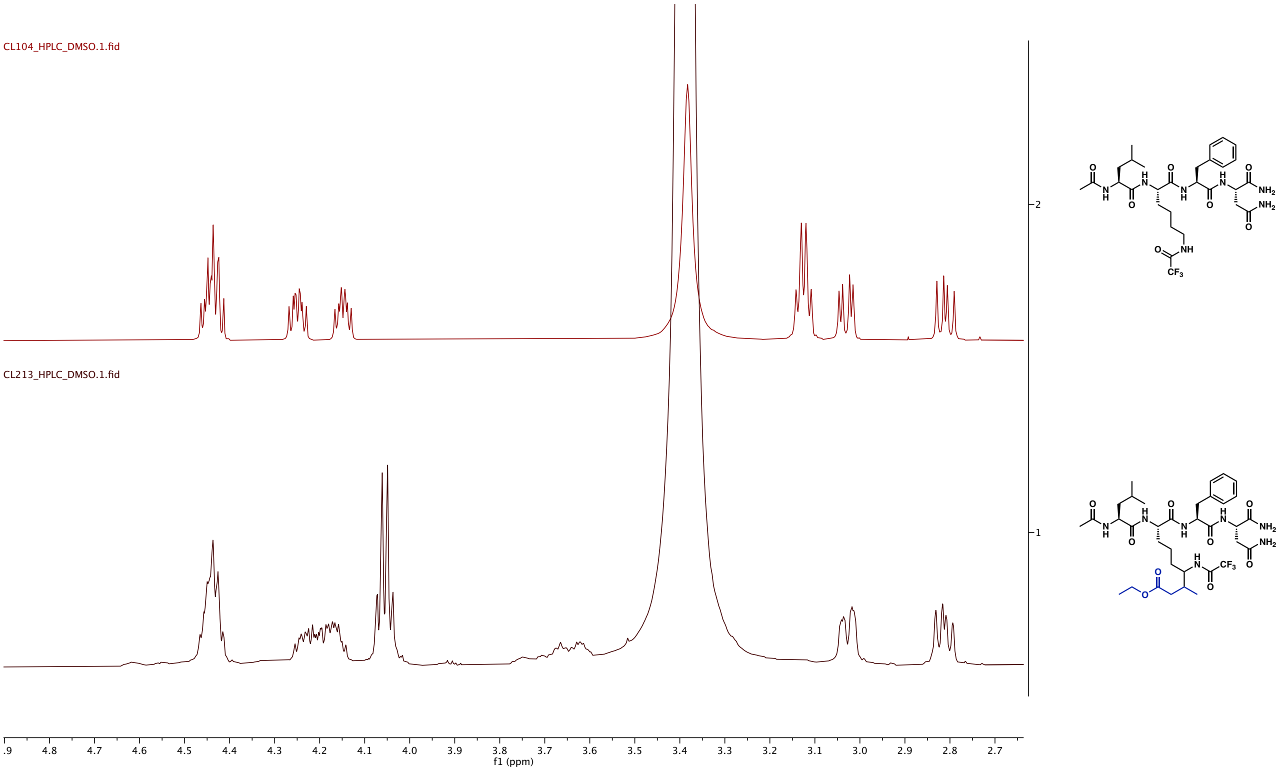
CL104_HPLC_DMSO.1.fid
CL213_HPLC_DMSO.1.fid
f1 (ppm)

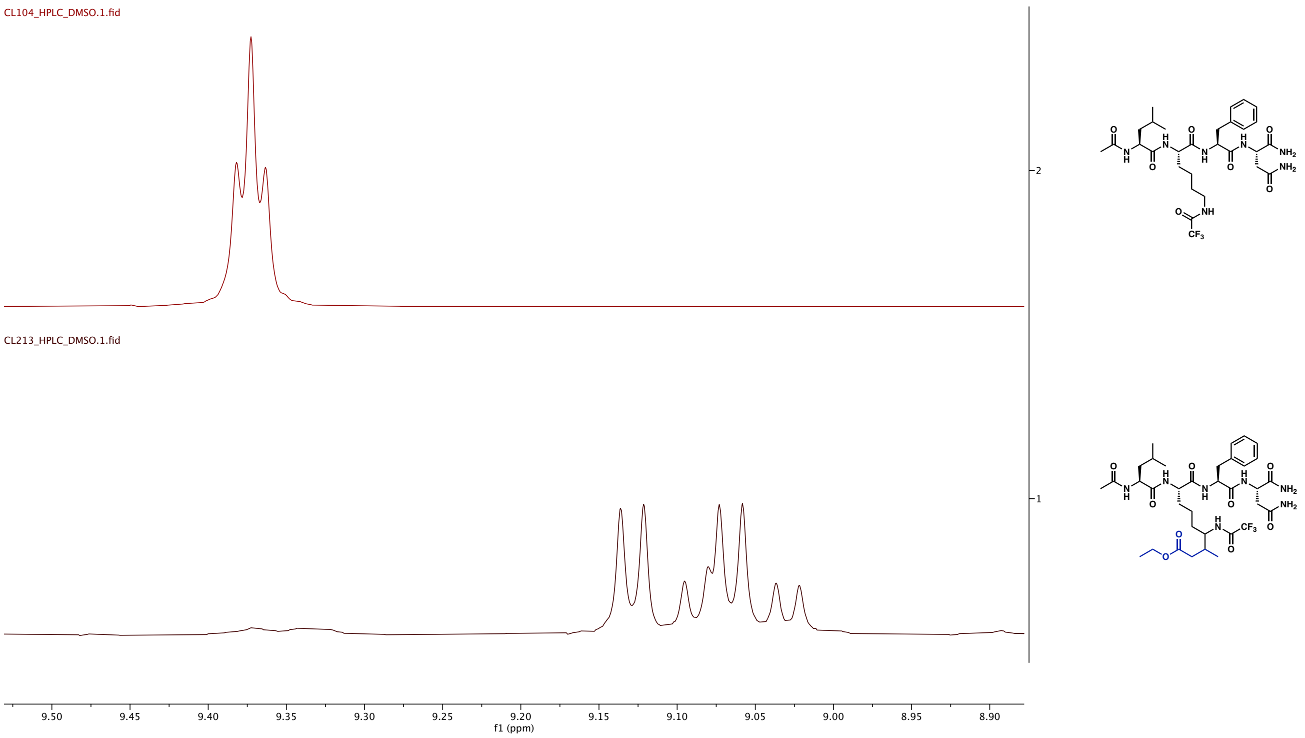

CL104_HPLC_DMSO.1.fid
CL213_HPLC_DMSO.1.fid
9.50
9.45
9.40
9.35
9.30
9.25
9.20
9.15
9.10
9.05
9.00
8.95
8.90
f1 (ppm)

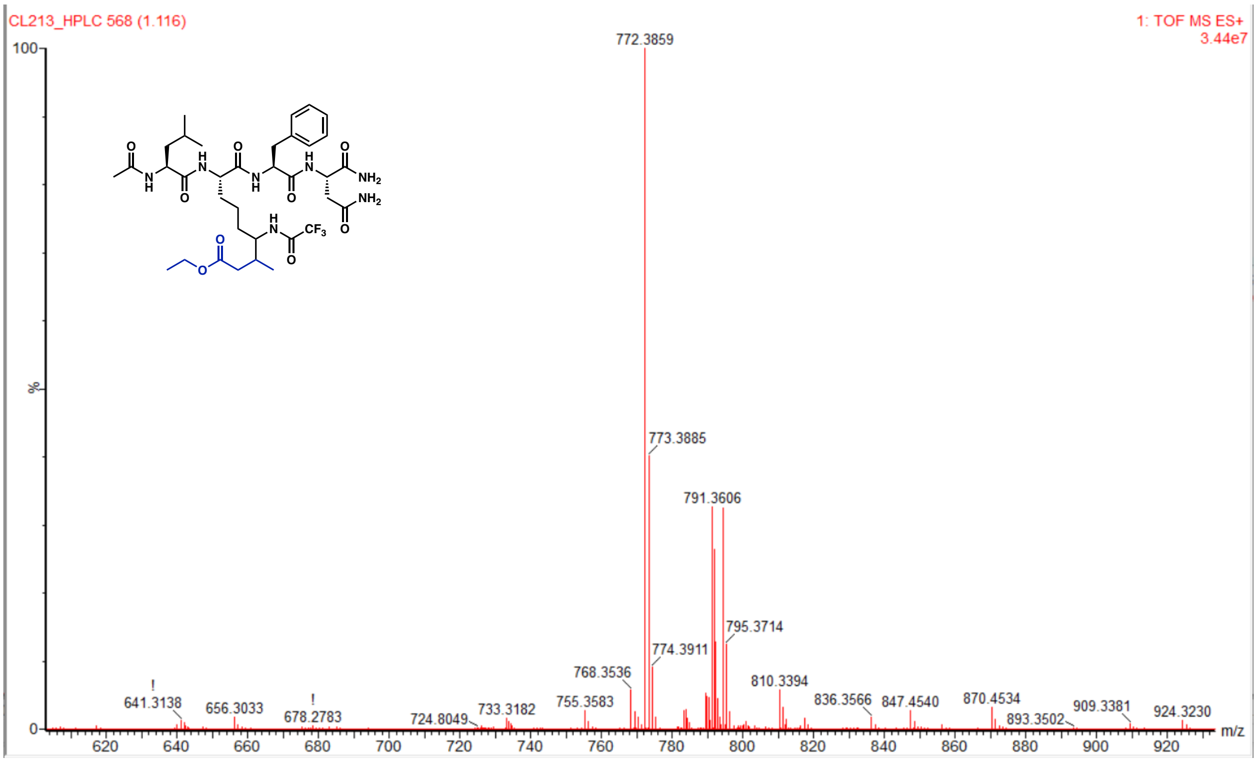

CL213_HPLC 568 (1.116)
1: TOF MS ES+
3.44e7
772.3859
773.3885
791.3606
795.3714
774.3911
768.3536
810.3394
755.3583
733.3182
724.8049
678.2783
656.3033
641.3138
836.3566
847.4540
870.4534
893.3502
909.3381
924.3230
m/z
%

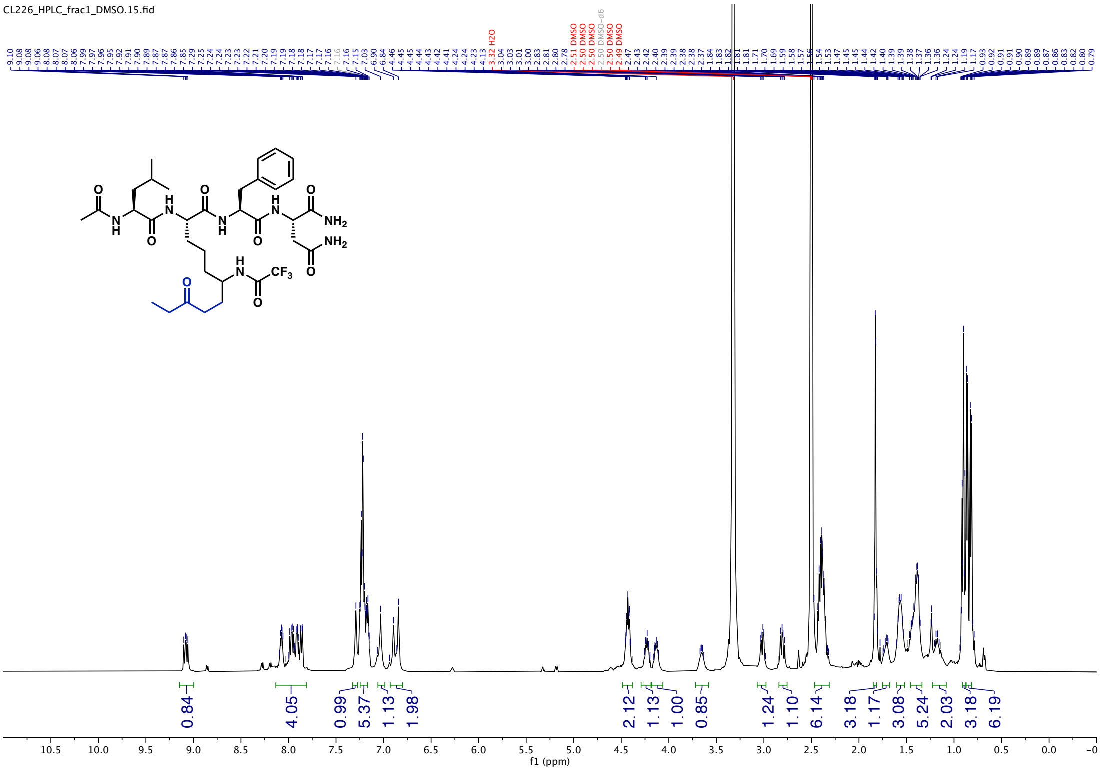
CL226_HPLC_frac1_DMSO.15.fid
f1 (ppm)

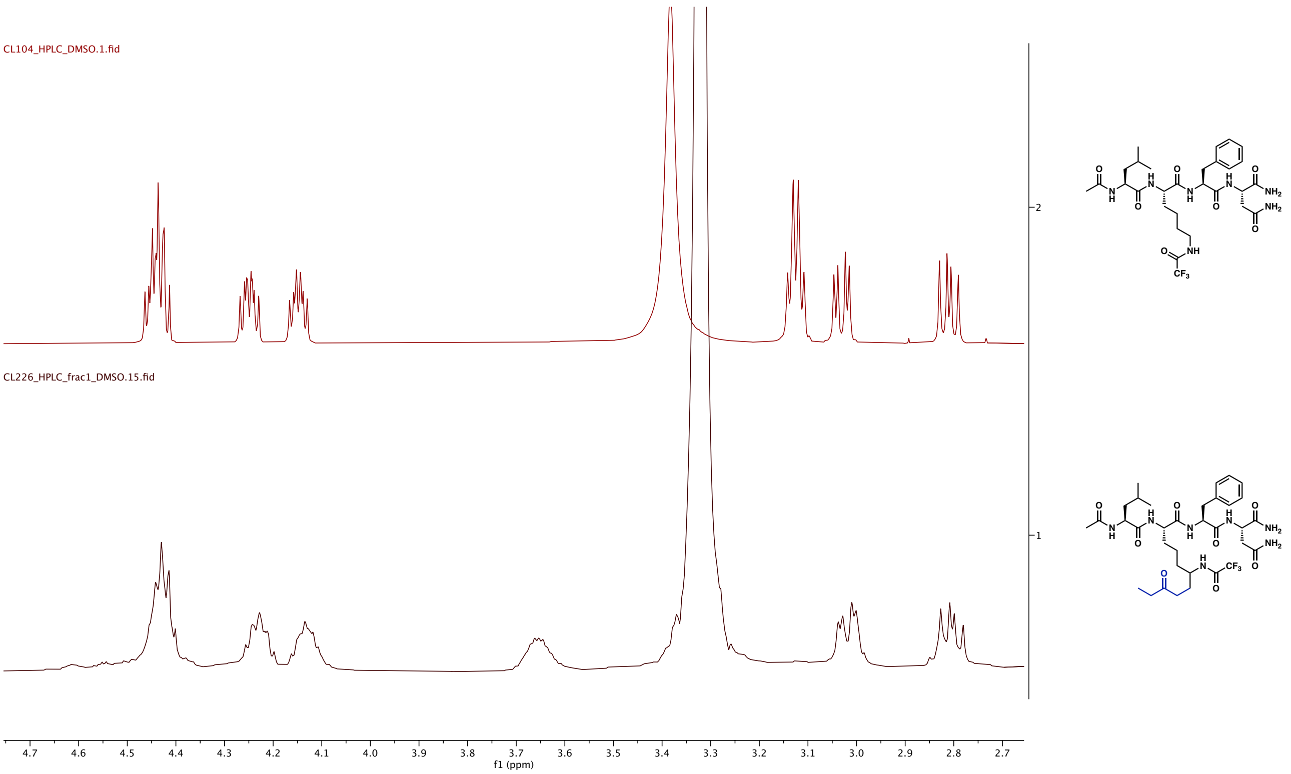
CL104_HPLC_DMSO.1.fid
CL226_HPLC_frac1_DMSO.15.fid
f1 (ppm)

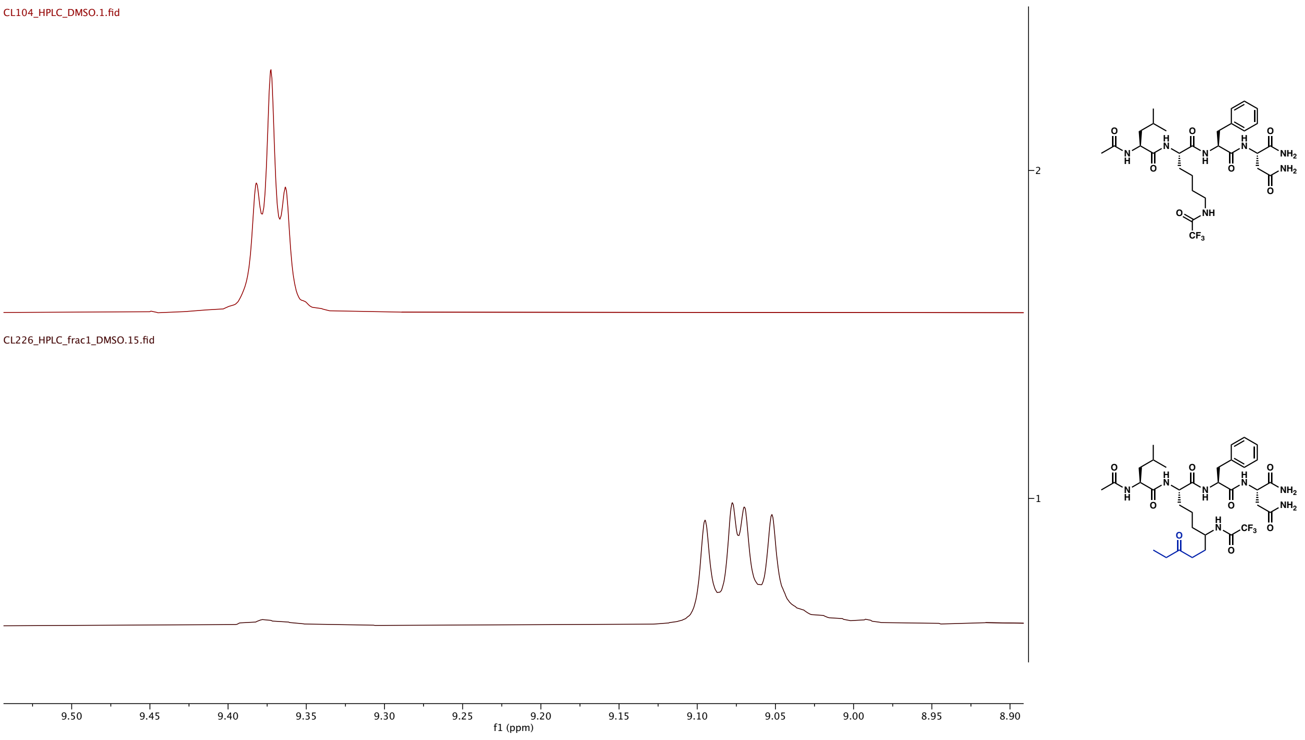
CL104_HPLC_DMSO.1.fid
CL226_HPLC_frac1_DMSO.15.fid
9.50 9.45 9.40 9.35 9.30 9.25 9.20 9.15 9.10 9.05 9.00 8.95 8.90
f1 (ppm)
2
1

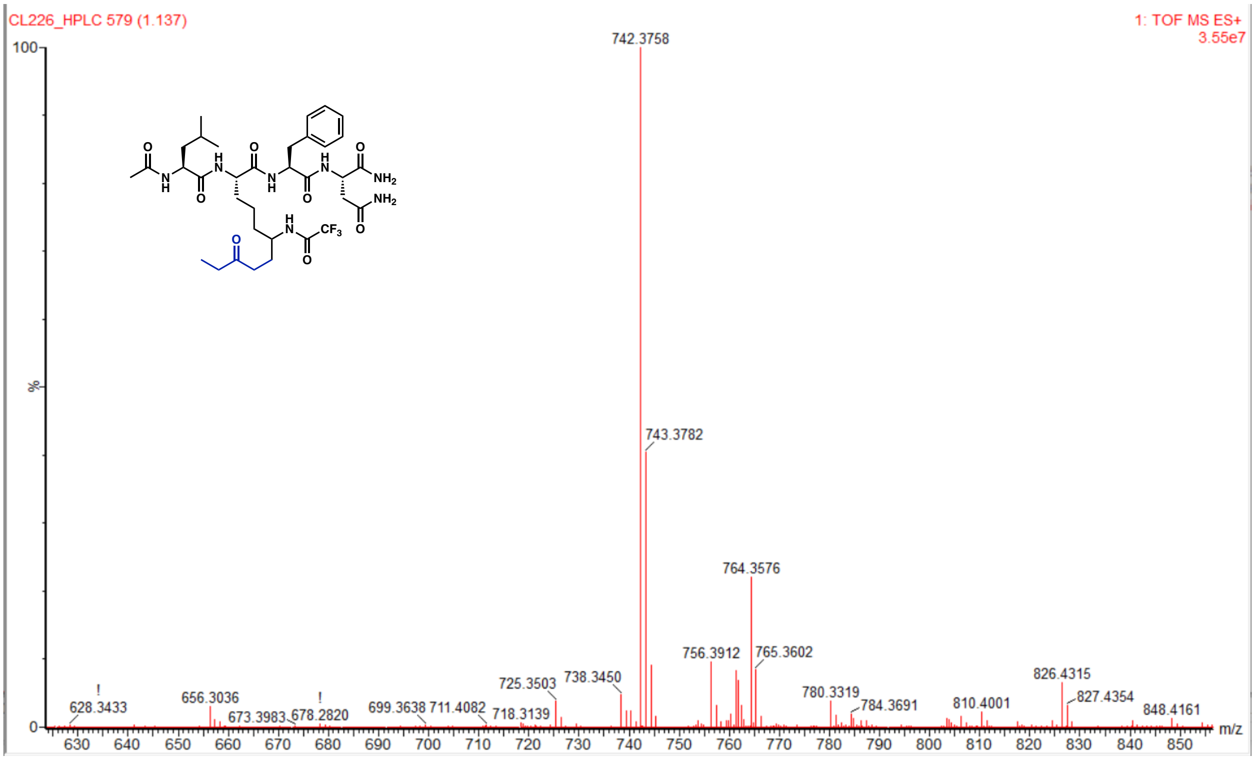
CL226_HPLC 579 (1.137)
1: TOF MS ES+
3.55e7
100
%
0
742.3758
743.3782
764.3576
756.3912
765.3602
738.3450
725.3503
780.3319
784.3691
810.4001
826.4315
827.4354
848.4161
628.3433
656.3036
673.3983
678.2820
699.3638
711.4082
718.3139
630 640 650 660 670 680 690 700 710 720 730 740 750 760 770 780 790 800 810 820 830 840 850
m/z

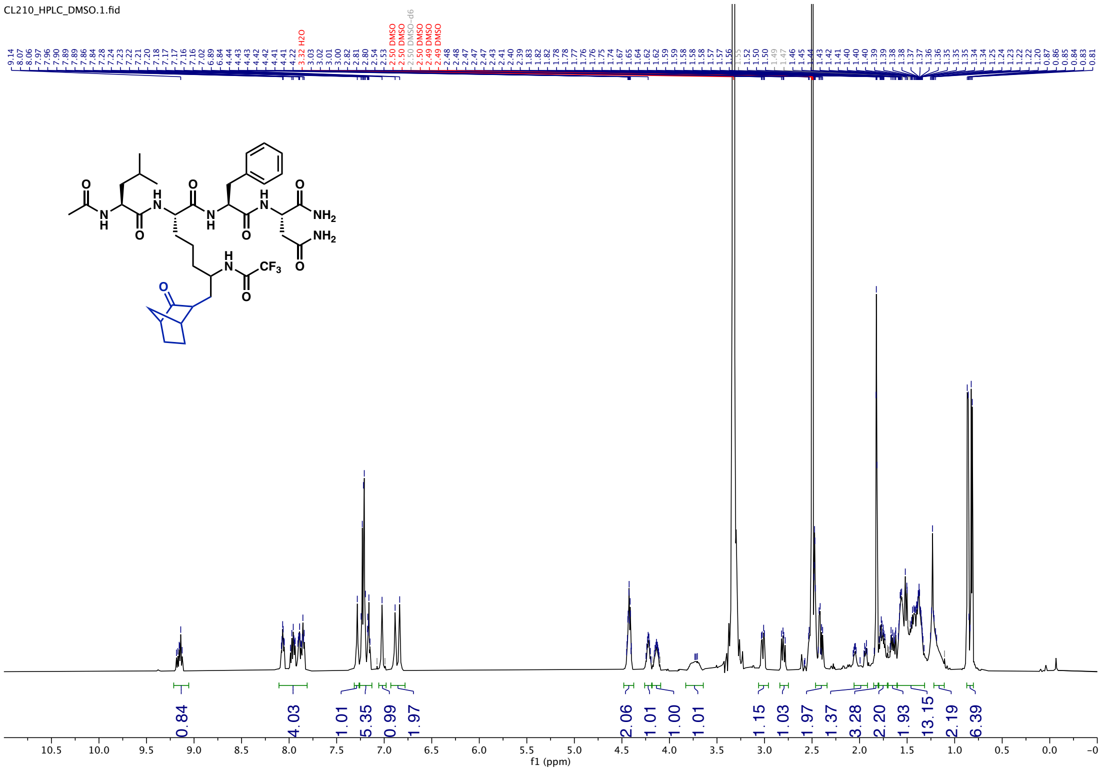
CL210_HPLC_DMSO.1.fid
3.32 H2O
2.50 DMSO
2.50 DMSO-d6
NH2
CF3
0.84
4.03
1.01
5.35
0.99
1.97
2.06
1.01
1.00
1.01
1.15
1.03
1.97
1.37
3.28
2.20
1.93
13.15
2.19
6.39
f1 (ppm)

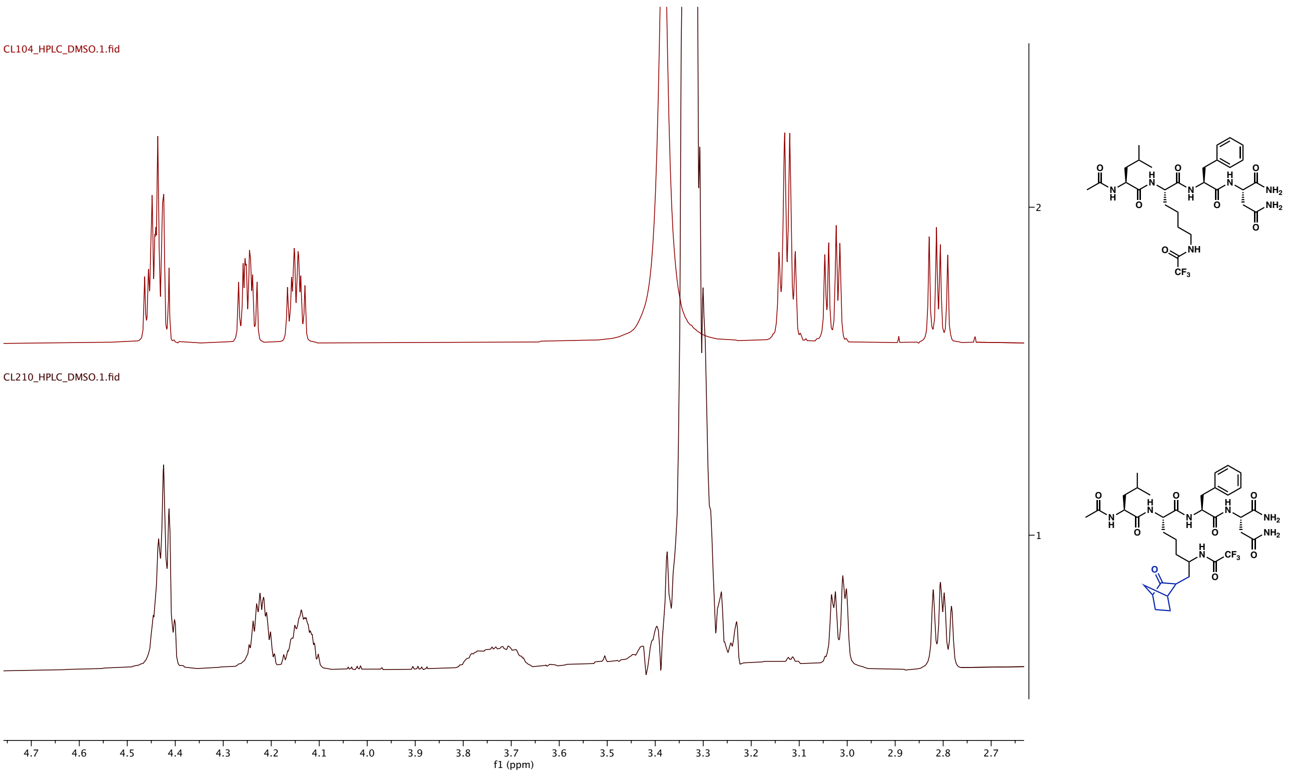
CL104_HPLC_DMSO.1.fid
CL210_HPLC_DMSO.1.fid
NH2
CF3
f1 (ppm)

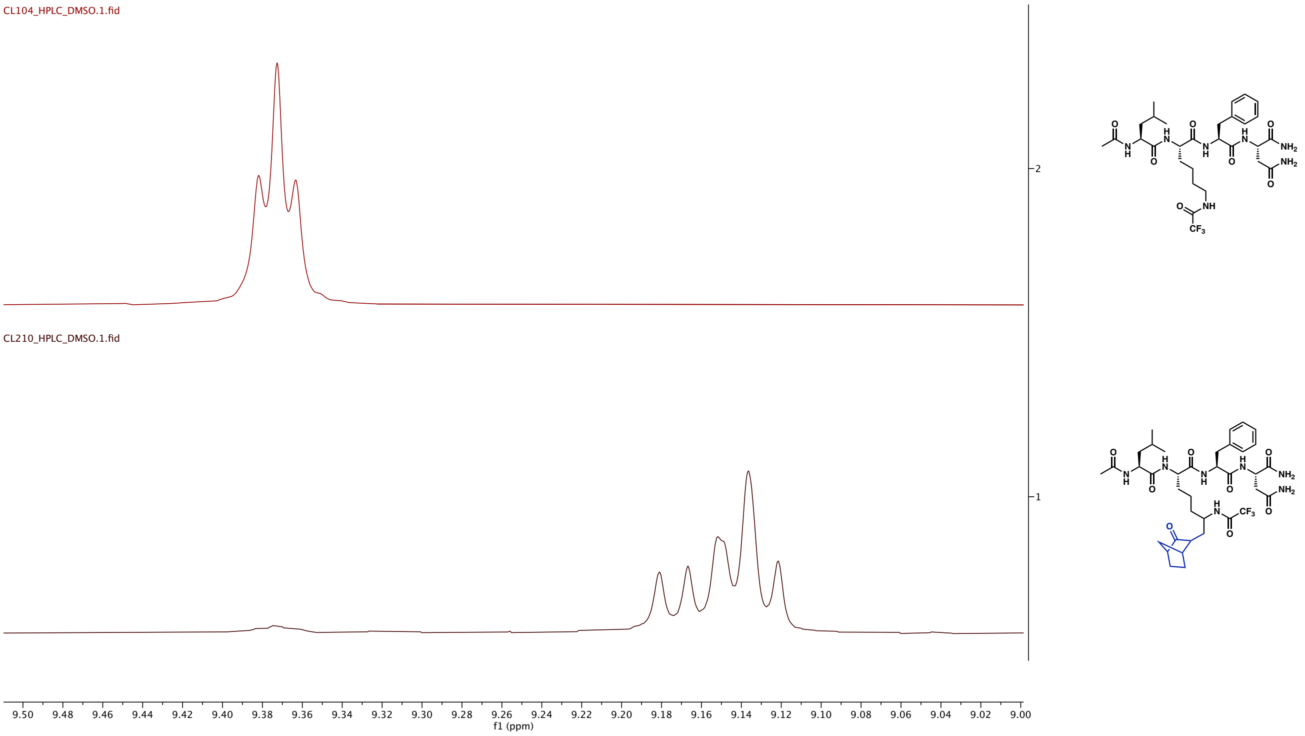
CL104_HPLC_DMSO.1.fid
CL210_HPLC_DMSO.1.fid
2
1
9.50 9.48 9.46 9.44 9.42 9.40 9.38 9.36 9.34 9.32 9.30 9.28 9.26 9.24 9.22 9.20 9.18 9.16 9.14 9.12 9.10 9.08 9.06 9.04 9.02 9.00
f1 (ppm)

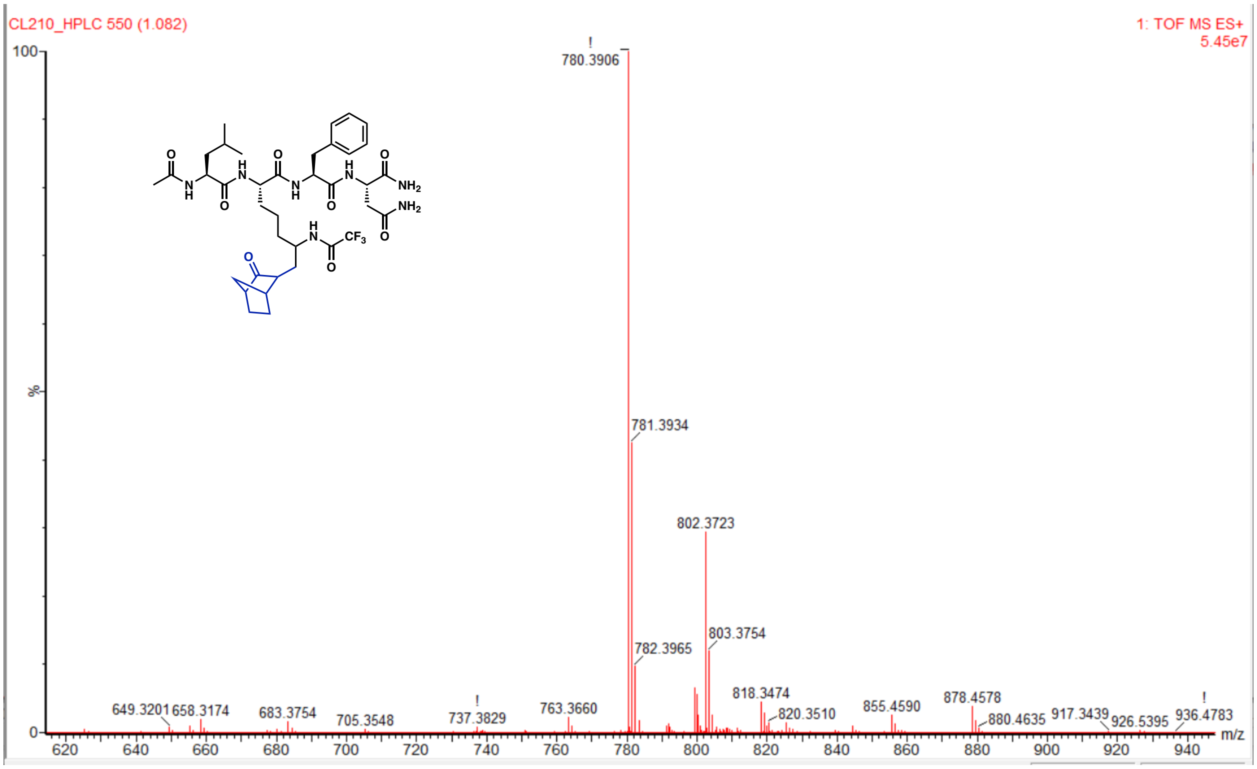
CL210_HPLC 550 (1.082)
1: TOF MS ES+
5.45e7
100
%
0
780.3906
781.3934
802.3723
782.3965
803.3754
818.3474
820.3510
855.4590
878.4578
880.4635
917.3439
926.5395
936.4783
649.3201
658.3174
683.3754
705.3548
737.3829
763.3660
620 640 660 680 700 720 740 760 780 800 820 840 860 880 900 920 940
m/z

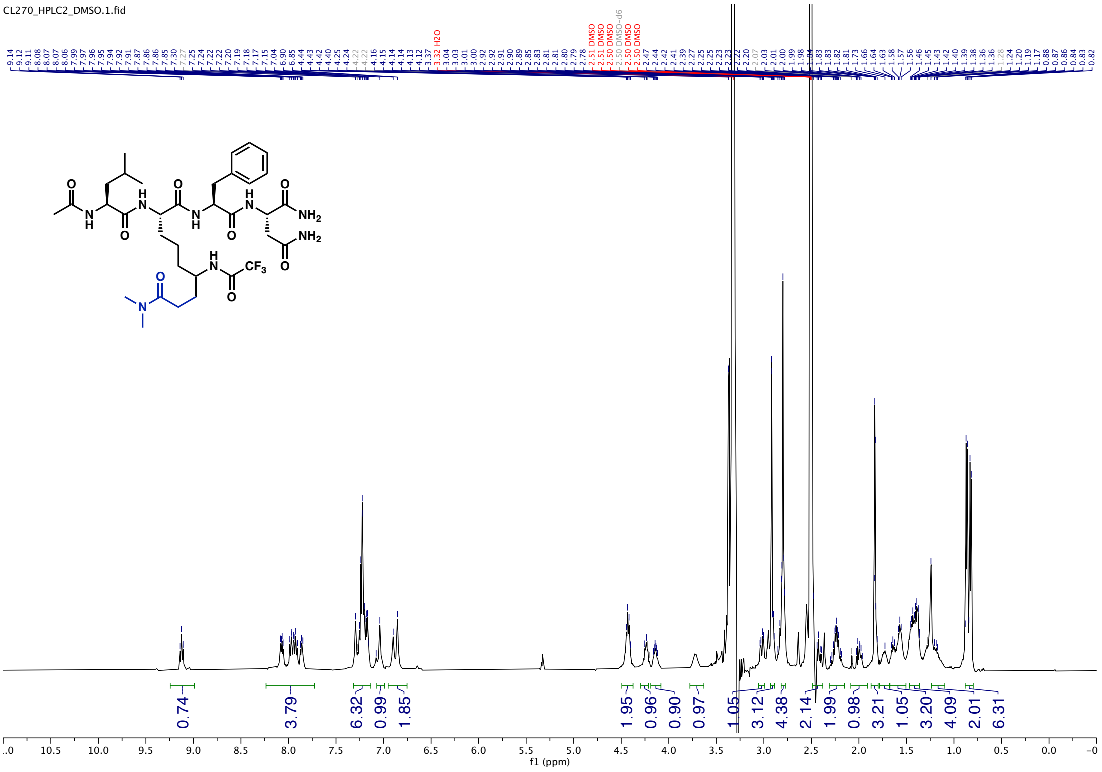
CL270_HPLC2_DMSO.1.fid
0.74
3.79
6.32
0.99
1.85
1.95
0.96
0.90
0.97
1.05
3.12
4.38
2.14
1.99
0.98
3.21
1.05
3.20
4.09
2.01
6.31
f1 (ppm)

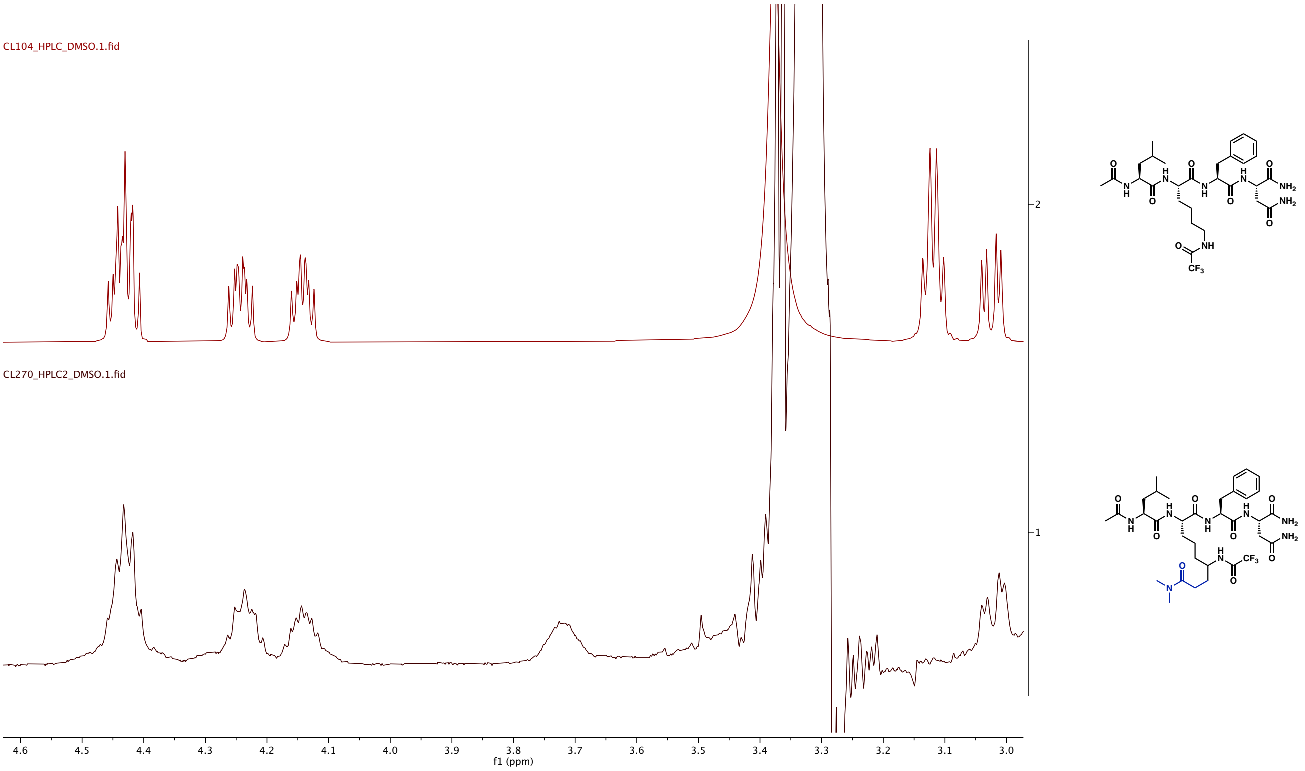
CL104_HPLC_DMSO.1.fid
CL270_HPLC2_DMSO.1.fid
f1 (ppm)

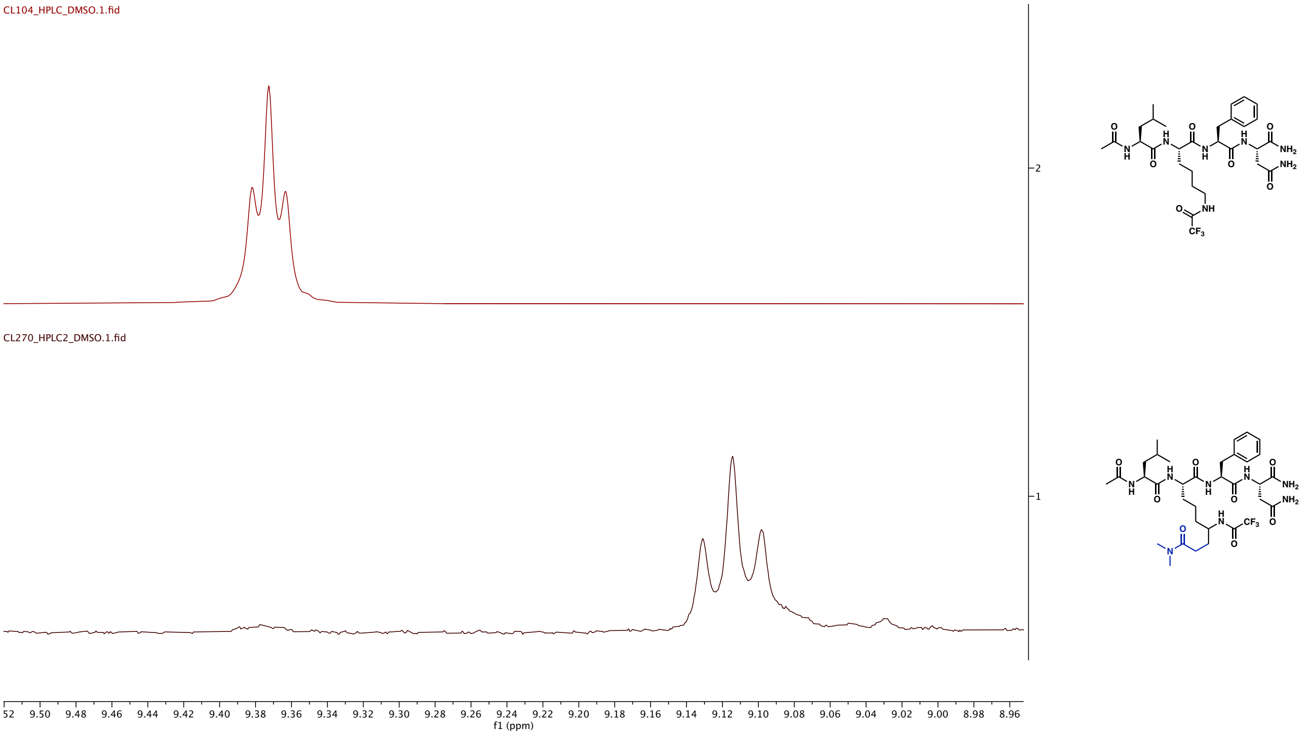
CL104_HPLC_DMSO.1.fid
CL270_HPLC2_DMSO.1.fid
52 9.50 9.48 9.46 9.44 9.42 9.40 9.38 9.36 9.34 9.32 9.30 9.28 9.26 9.24 9.22 9.20 9.18 9.16 9.14 9.12 9.10 9.08 9.06 9.04 9.02 9.00 8.98 8.96
f1 (ppm)

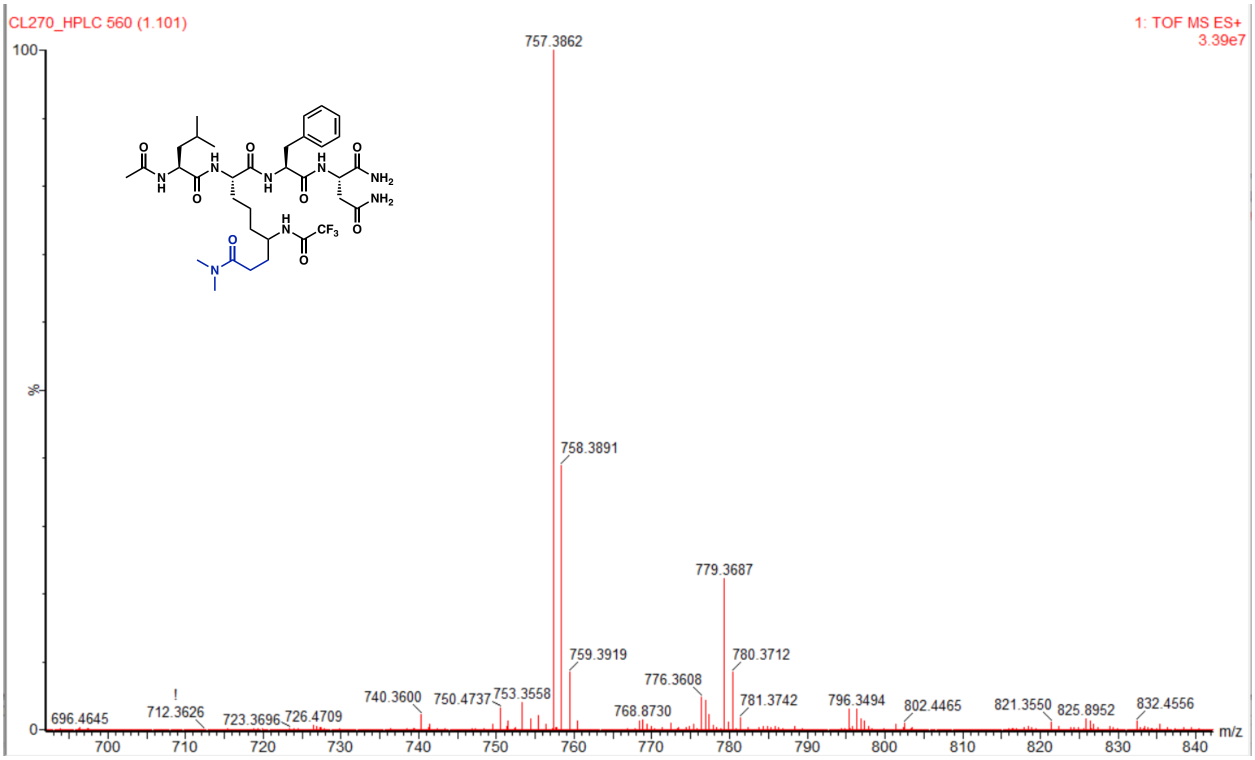
CL270_HPLC 560 (1.101)
1: TOF MS ES+
3.39e7
757.3862
758.3891
779.3687
759.3919
780.3712
776.3608
781.3742
796.3494
802.4465
821.3550
825.8952
832.4556
768.8730
753.3558
750.4737
740.3600
726.4709
723.3696
712.3626
696.4645
%
m/z
700 710 720 730 740 750 760 770 780 790 800 810 820 830 840

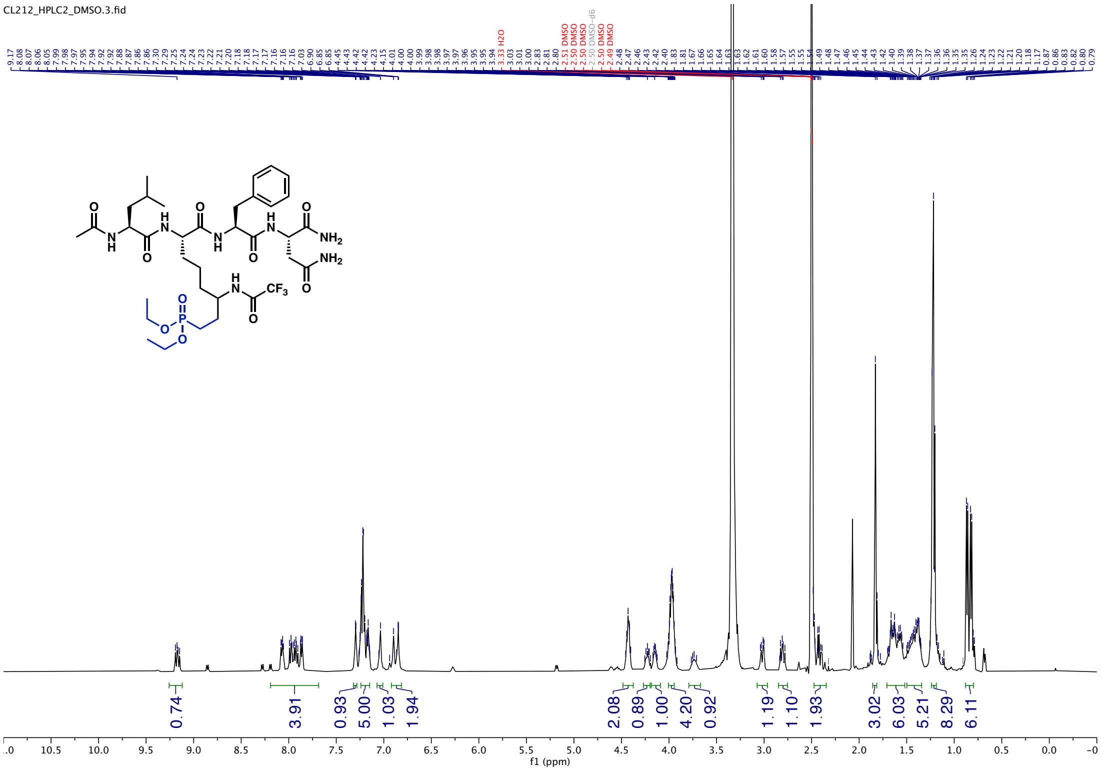
CL212_HPLC2_DMSO.3.fid
0.74
3.91
0.93
5.00
1.03
1.94
2.08
0.89
1.00
4.20
0.92
1.19
1.10
1.93
3.02
6.03
5.21
8.29
6.11
f1 (ppm)

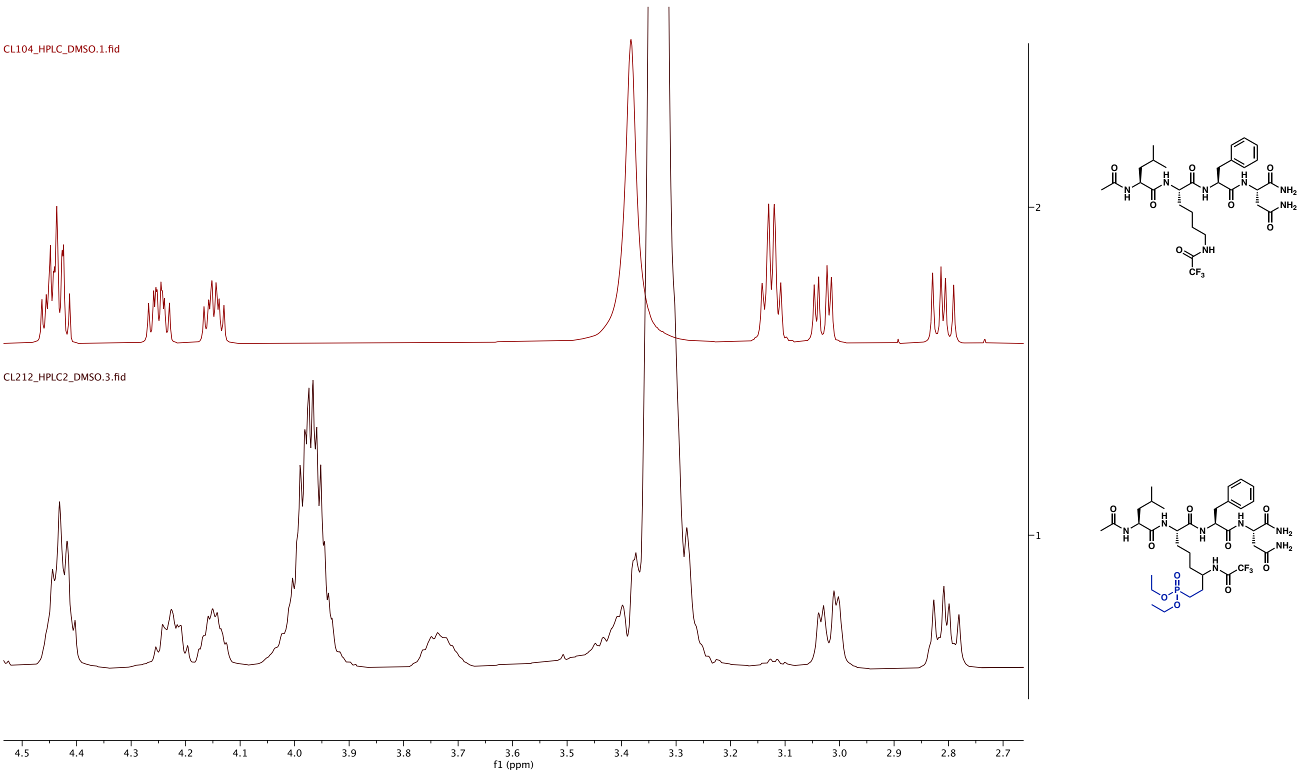
CL104_HPLC_DMSO.1.fid
CL212_HPLC2_DMSO.3.fid
f1 (ppm)

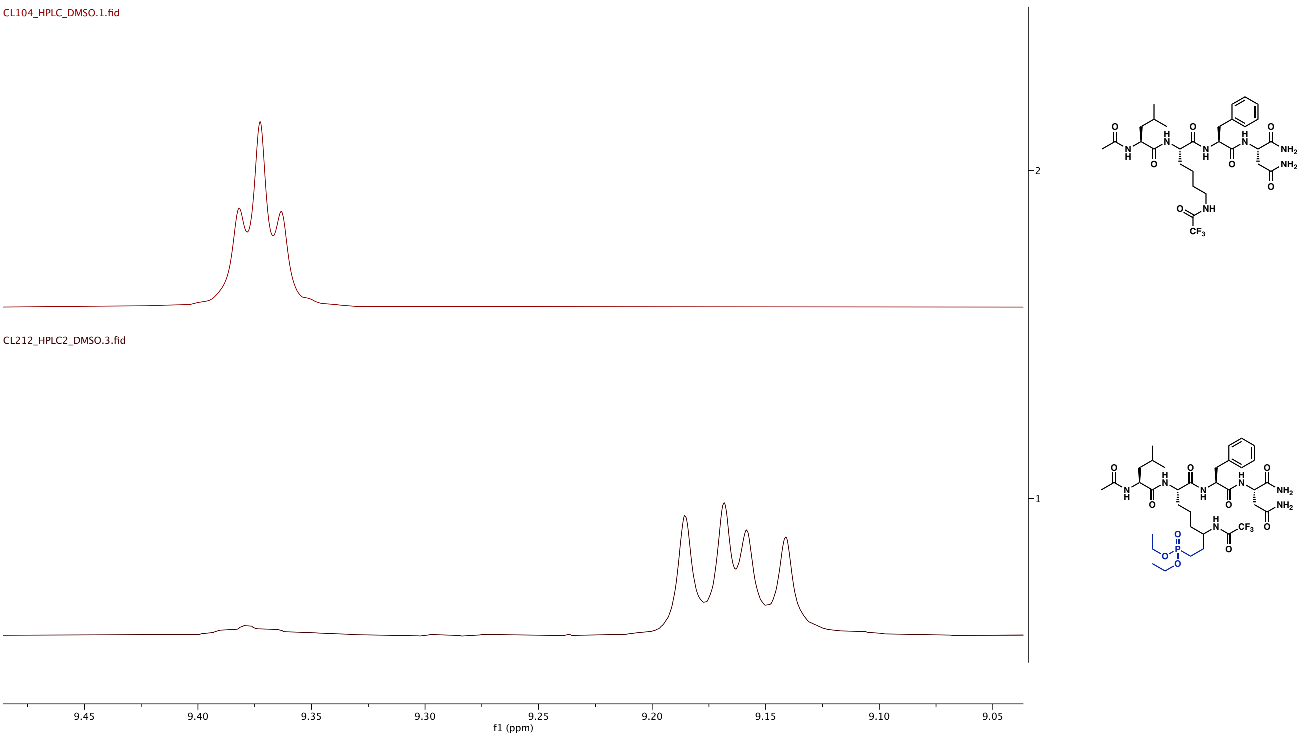

CL104_HPLC_DMSO.1.fid
CL212_HPLC2_DMSO.3.fid
2
1
9.45
9.40
9.35
9.30
9.25
9.20
9.15
9.10
9.05
f1 (ppm)

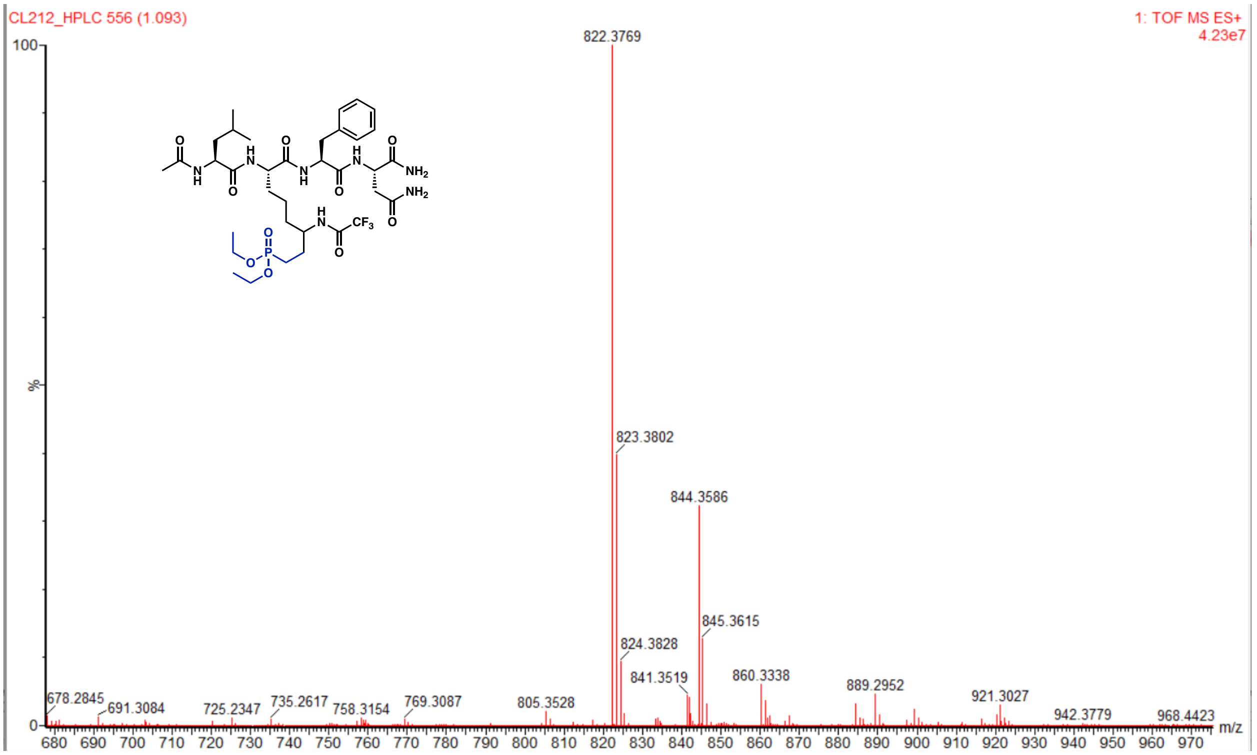

CL212_HPLC 556 (1.093)
1: TOF MS ES+
4.23e7
100
%
0
822.3769
823.3802
844.3586
845.3615
824.3828
841.3519
860.3338
889.2952
921.3027
678.2845
691.3084
725.2347
735.2617
758.3154
769.3087
805.3528
942.3779
968.4423
m/z
680 690 700 710 720 730 740 750 760 770 780 790 800 810 820 830 840 850 860 870 880 890 900 910 920 930 940 950 960 970

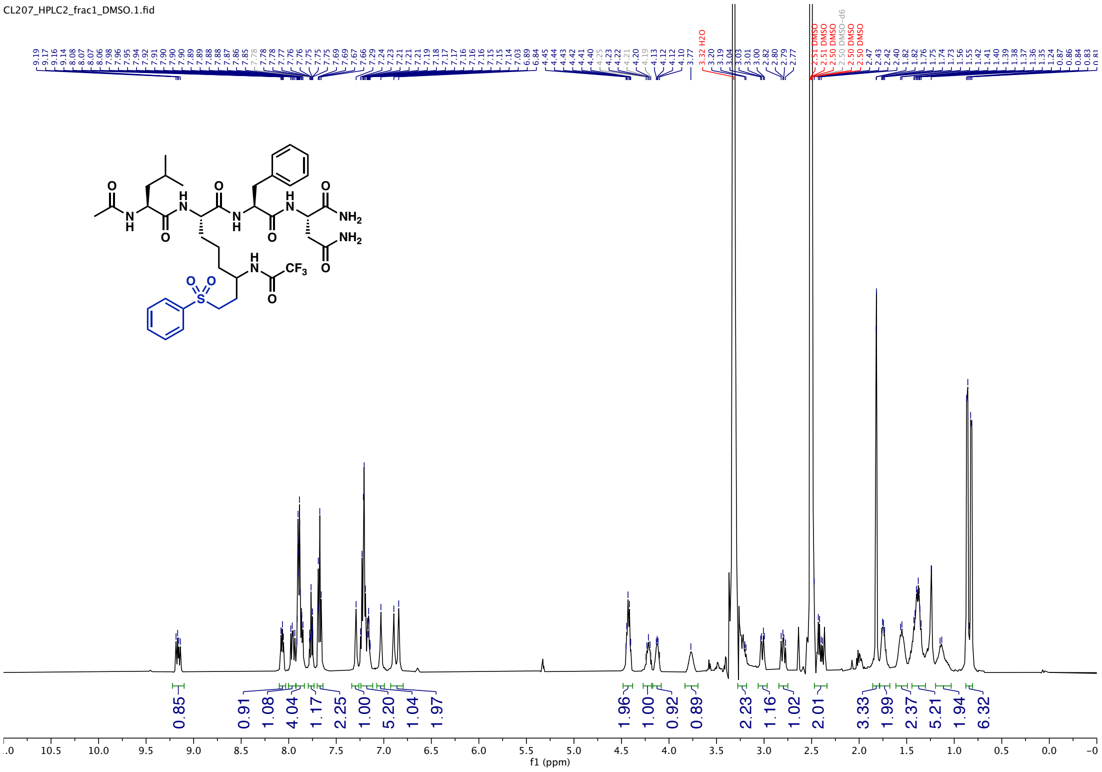
CL207_HPLC2_frac1_DMSO.1.fid
f1 (ppm)

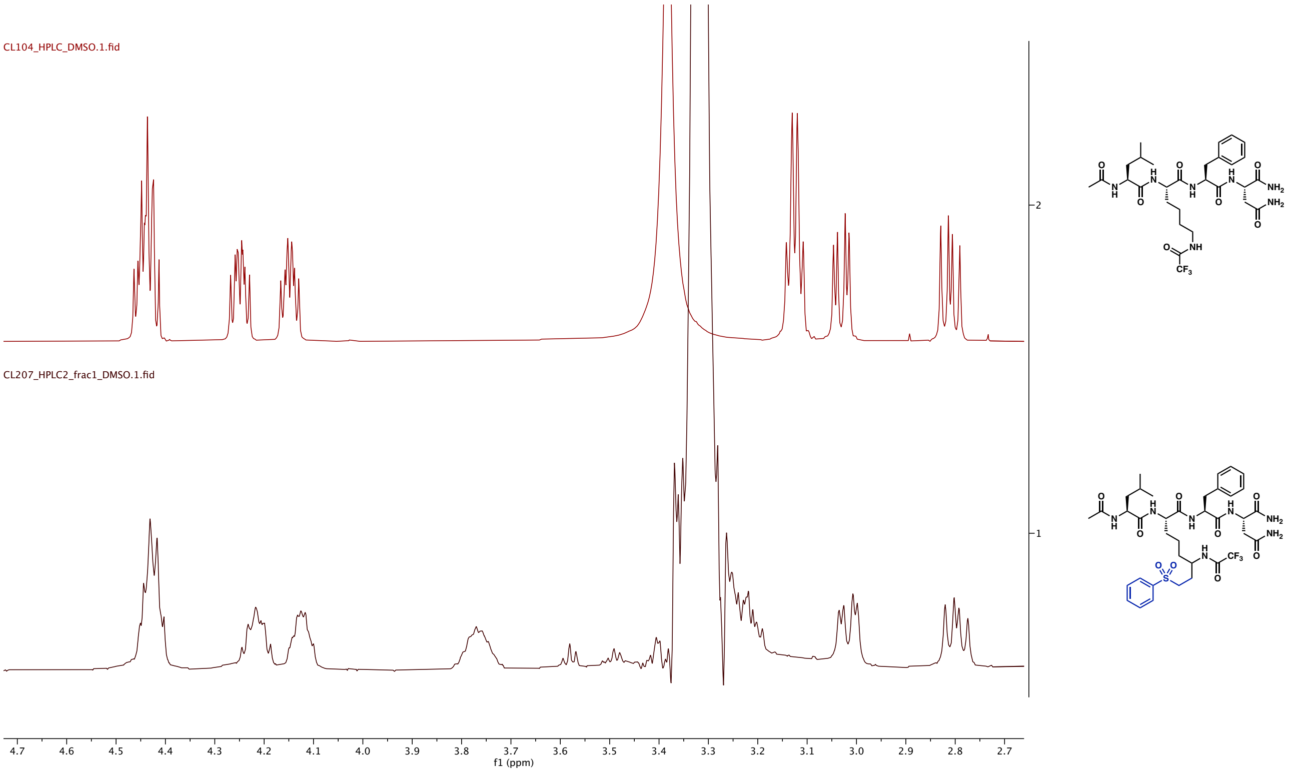
CL104_HPLC_DMSO.1.fid
CL207_HPLC2_frac1_DMSO.1.fid
f1 (ppm)

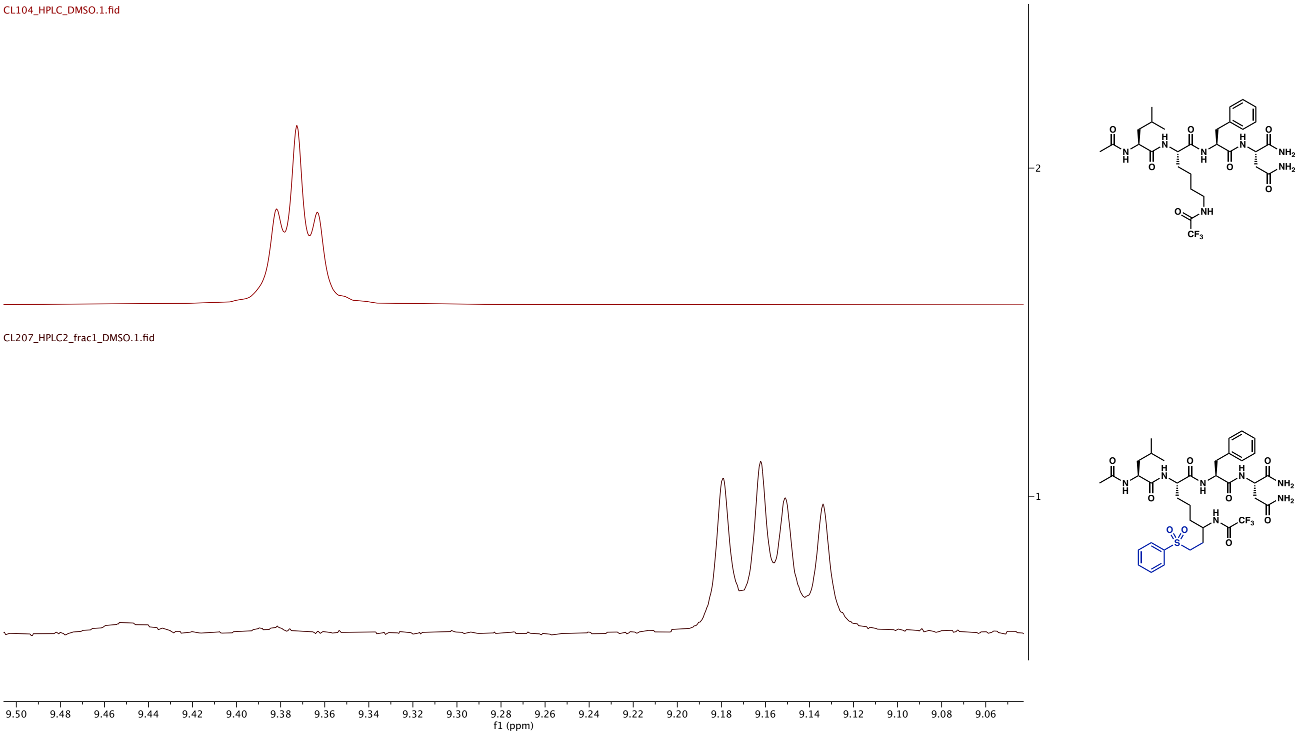
CL104_HPLC_DMSO.1.fid
CL207_HPLC2_frac1_DMSO.1.fid
9.50 9.48 9.46 9.44 9.42 9.40 9.38 9.36 9.34 9.32 9.30 9.28 9.26 9.24 9.22 9.20 9.18 9.16 9.14 9.12 9.10 9.08 9.06
f1 (ppm)

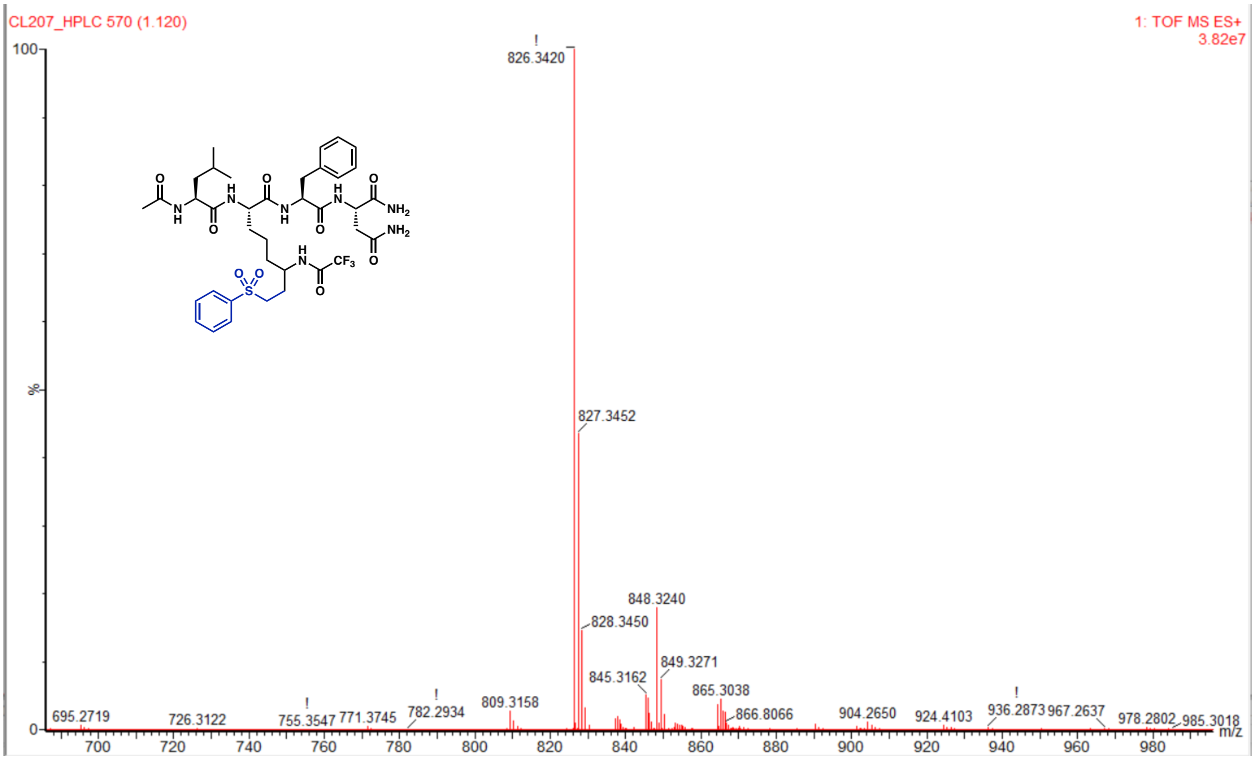
CL207_HPLC 570 (1.120)
1: TOF MS ES+
3.82e7
826.3420
827.3452
848.3240
828.3450
849.3271
845.3162
865.3038
809.3158
866.8066
904.2650
924.4103
936.2873
967.2637
978.2802
985.3018
695.2719
726.3122
755.3547
771.3745
782.2934
m/z

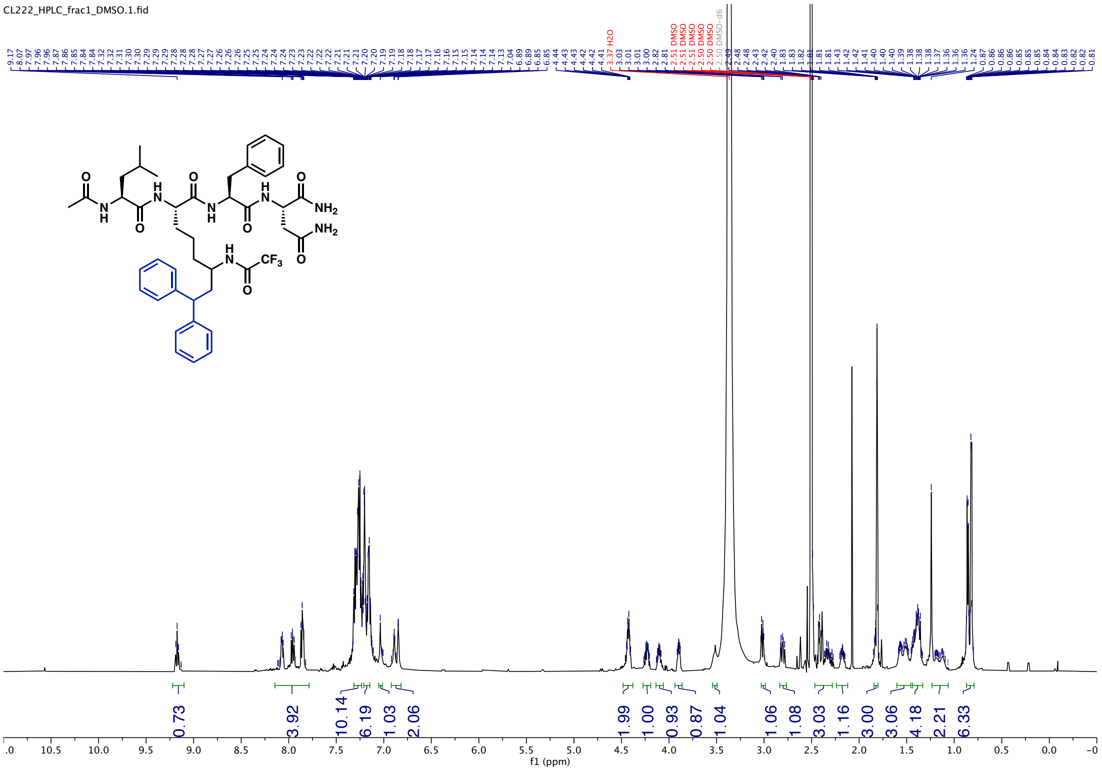
CL222_HPLC_frac1_DMSO.1.fid
f1 (ppm)

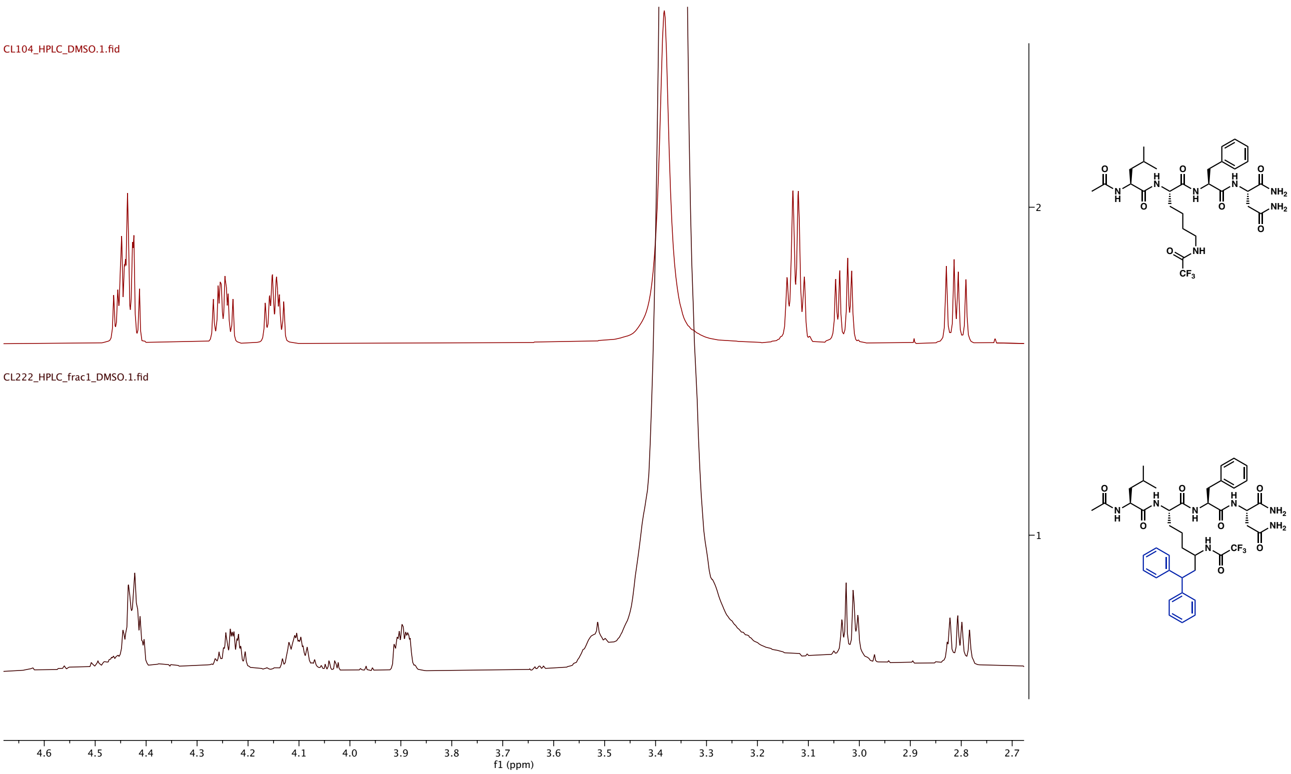
CL104_HPLC_DMSO.1.fid
CL222_HPLC_frac1_DMSO.1.fid
f1 (ppm)

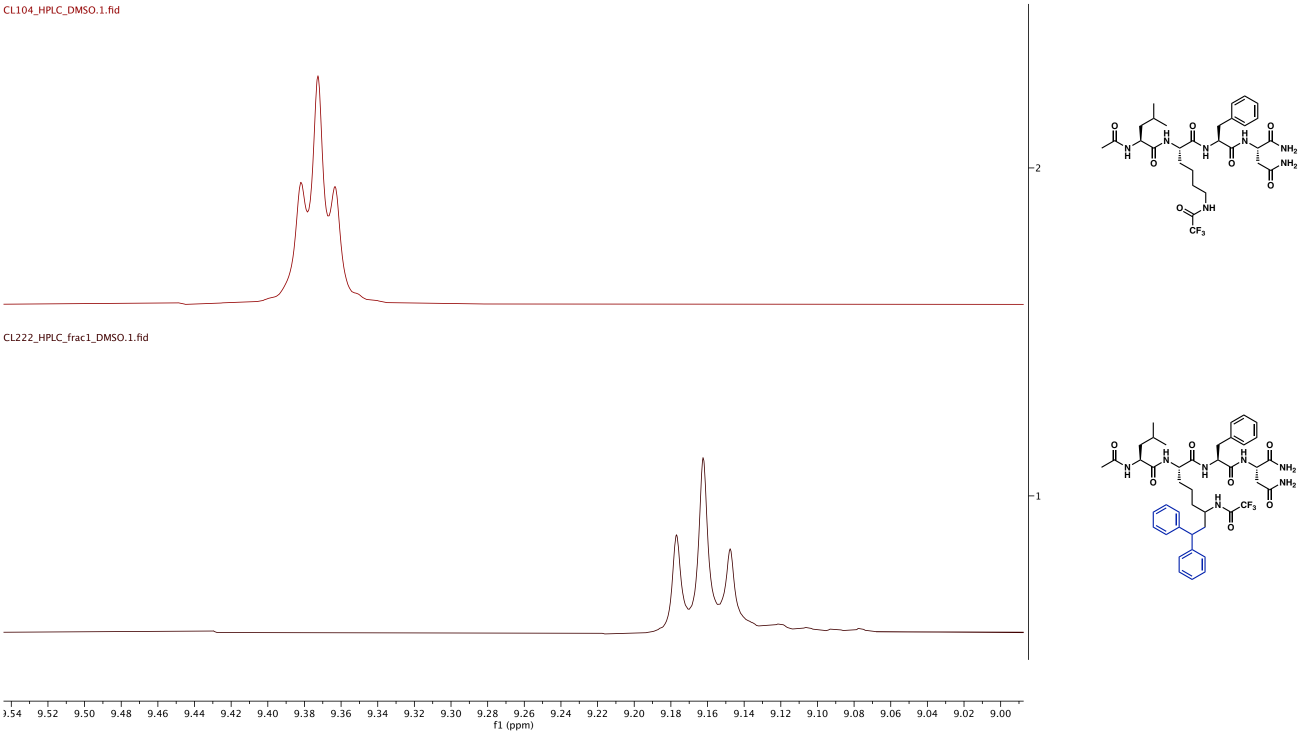

CL104_HPLC_DMSO.1.fid
CL222_HPLC_frac1_DMSO.1.fid
f1 (ppm)

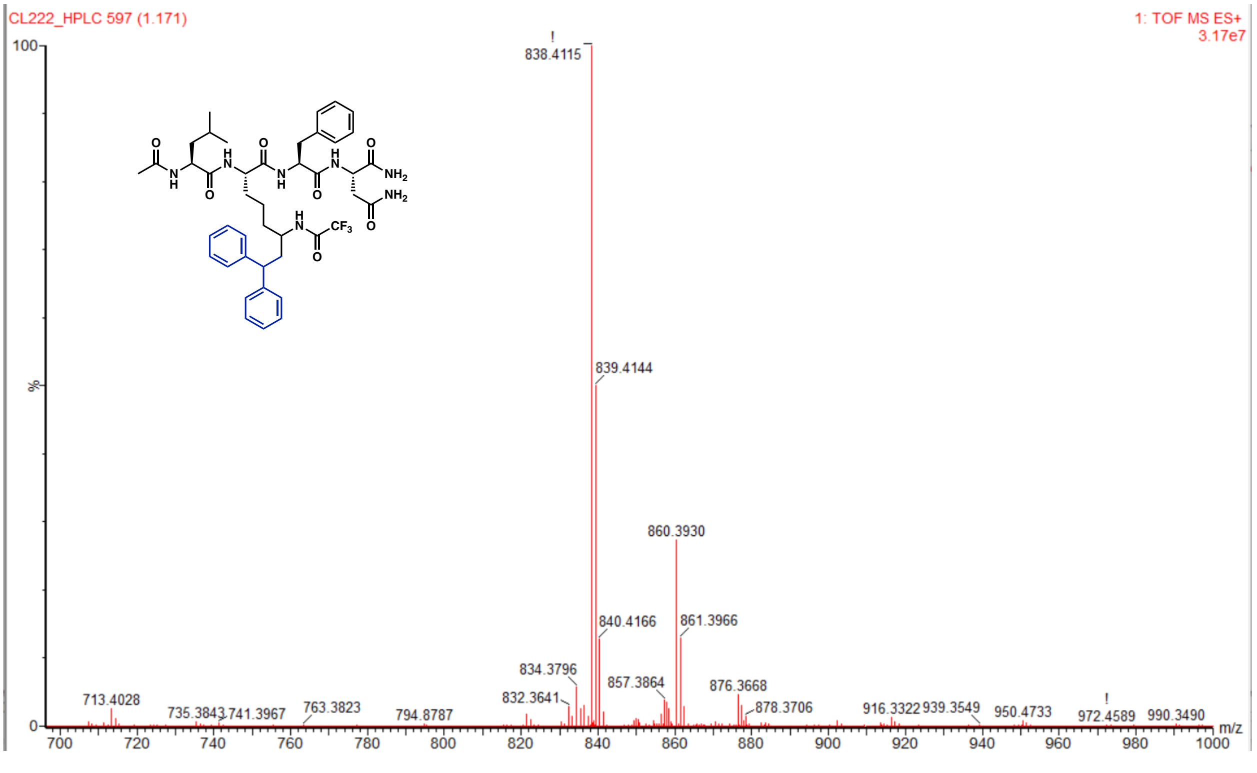

CL222_HPLC 597 (1.171)
1: TOF MS ES+
3.17e7
838.4115
839.4144
860.3930
840.4166
861.3966
834.3796
857.3864
876.3668
832.3641
878.3706
713.4028
735.3843
741.3967
763.3823
794.8787
916.3322
939.3549
950.4733
972.4589
990.3490
m/z
%

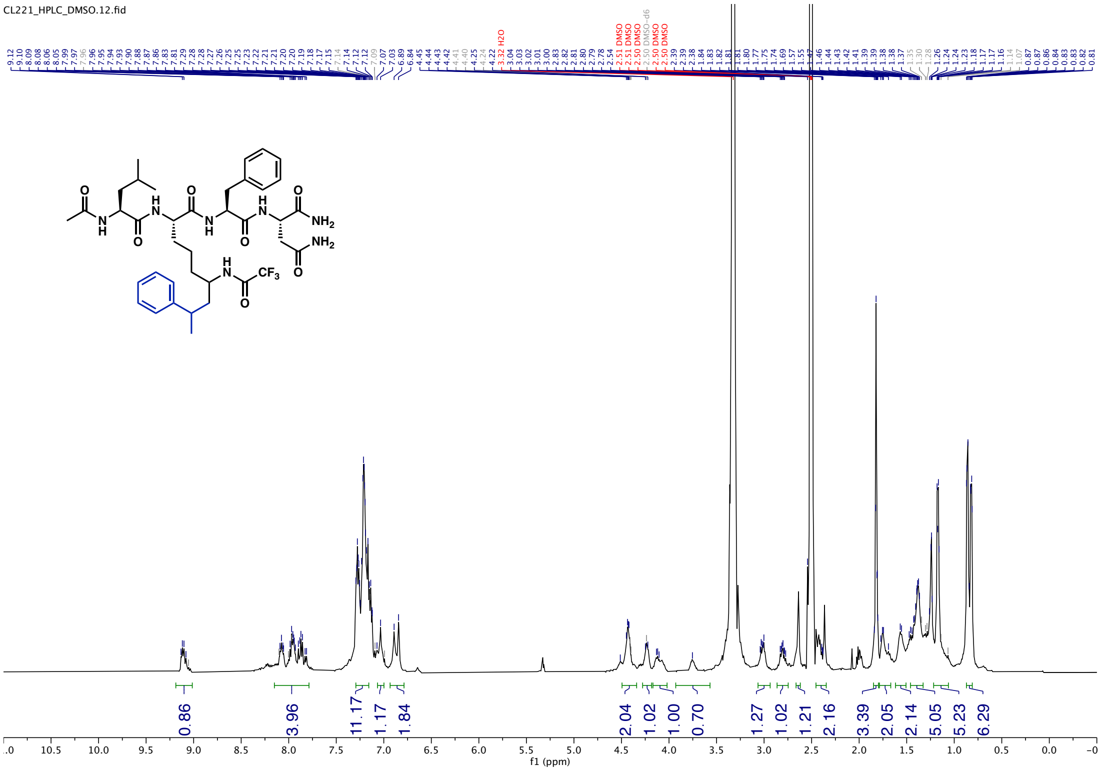
CL221_HPLC_DMSO.12.fid
0.86
3.96
11.17
1.17
1.84
2.04
1.02
1.00
0.70
1.27
1.02
1.21
2.16
3.39
2.05
2.14
5.05
5.23
6.29
f1 (ppm)

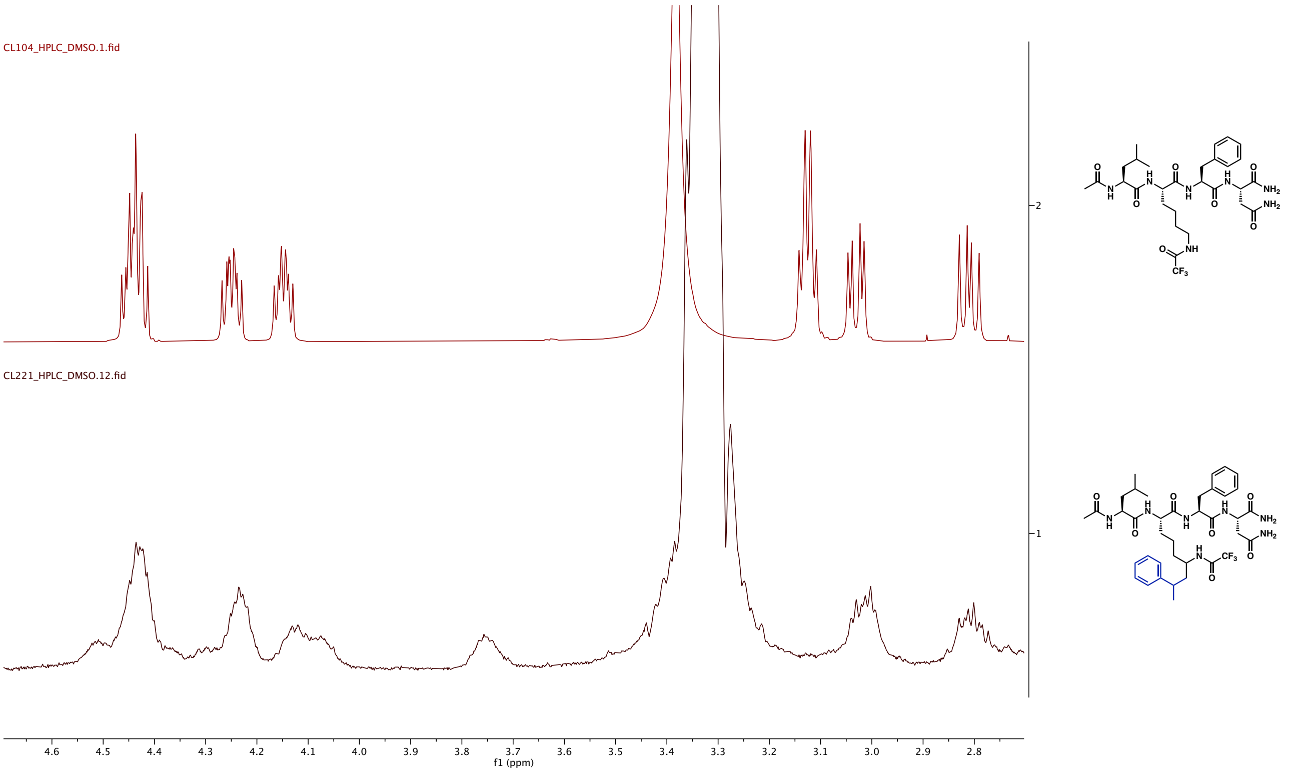
CL104_HPLC_DMSO.1.fid
CL221_HPLC_DMSO.12.fid
f1 (ppm)

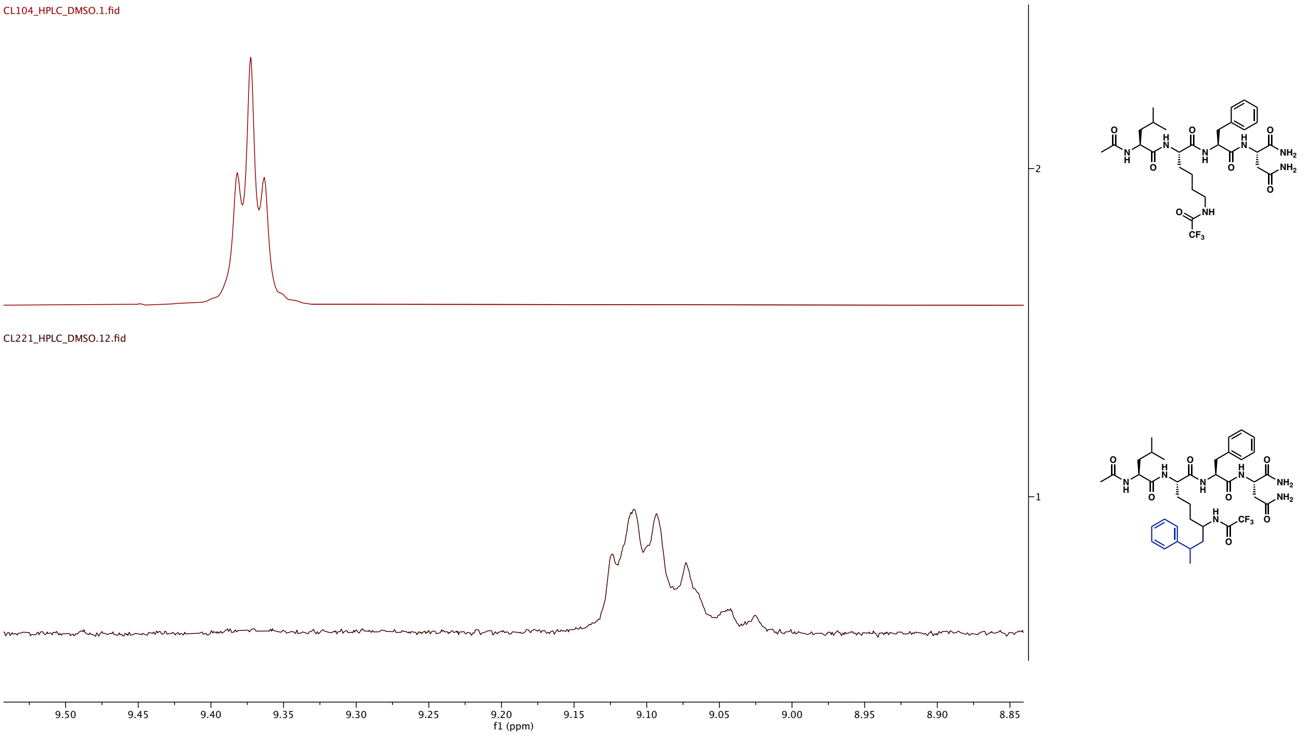

CL104_HPLC_DMSO.1.fid
CL221_HPLC_DMSO.12.fid
9.50
9.45
9.40
9.35
9.30
9.25
9.20
9.15
9.10
9.05
9.00
8.95
8.90
8.85
f1 (ppm)

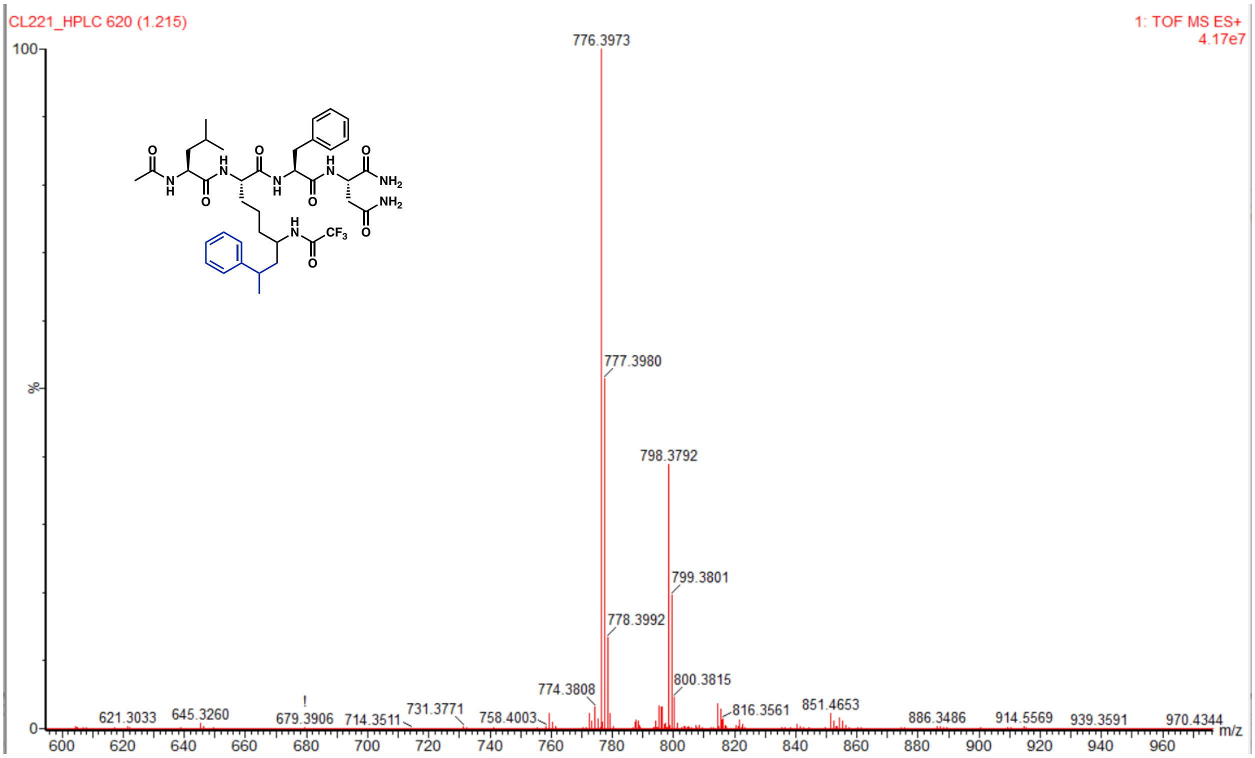

CL221_HPLC 620 (1.215)
1: TOF MS ES+
4.17e7
100
%
0
776.3973
777.3980
798.3792
799.3801
778.3992
800.3815
774.3808
816.3561
851.4653
621.3033
645.3260
679.3906
714.3511
731.3771
758.4003
886.3486
914.5569
939.3591
970.4344
m/z

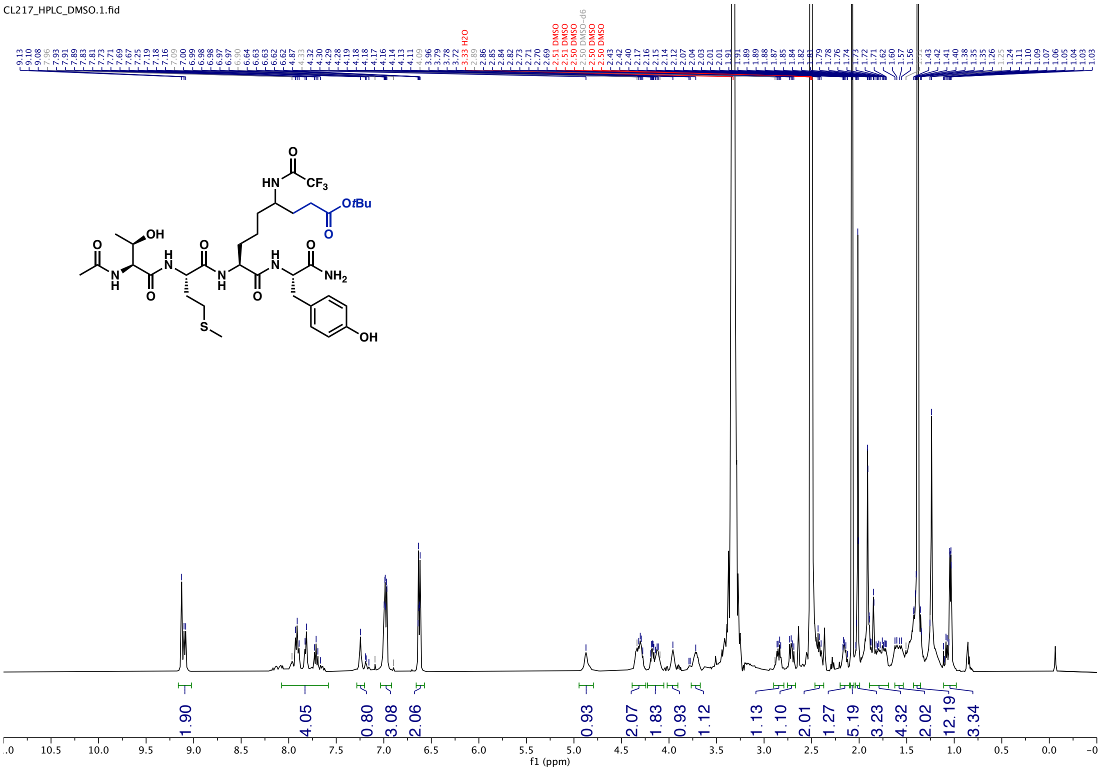
CL217_HPLC_DMSO.1.fid
f1 (ppm)
1.90
4.05
0.80
3.08
2.06
0.93
2.07
1.83
0.93
1.12
1.13
1.10
2.01
1.27
5.19
3.23
4.32
2.02
12.19
3.34

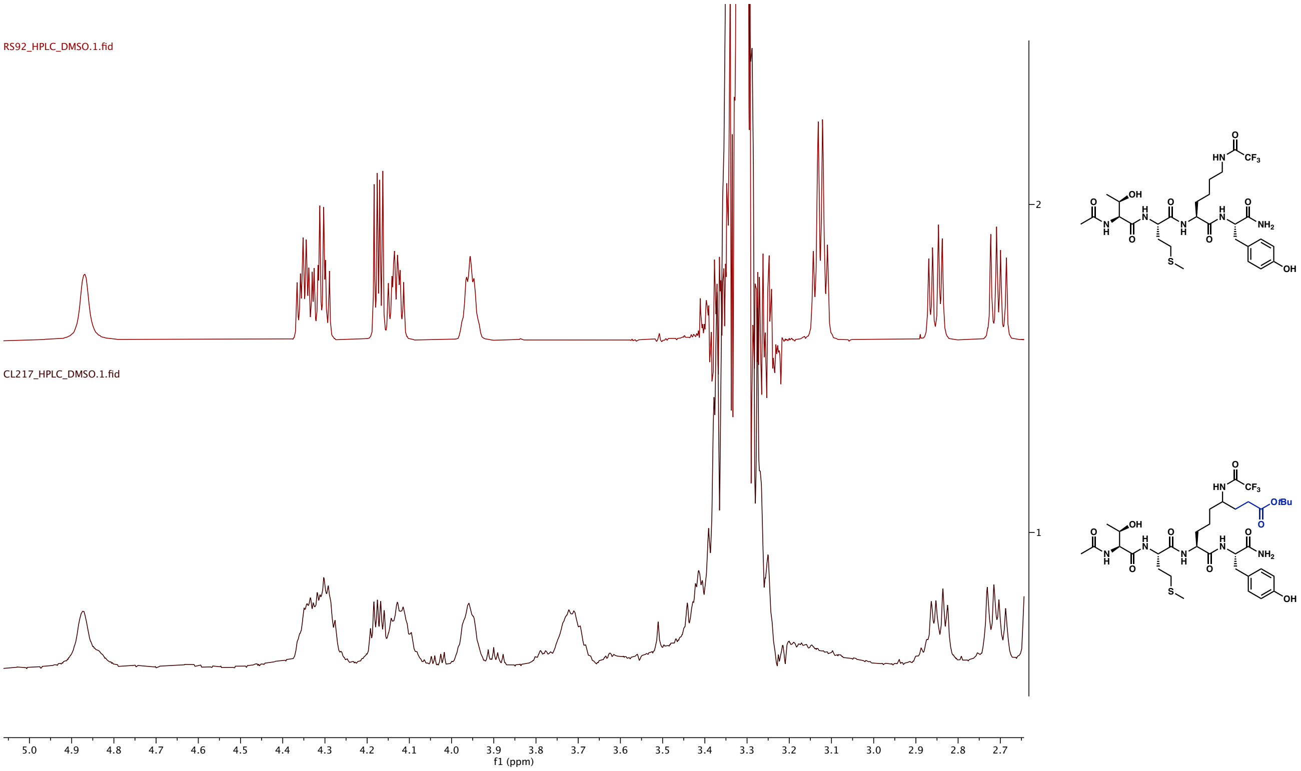
RS92_HPLC_DMSO.1.fid
CL217_HPLC_DMSO.1.fid
f1 (ppm)

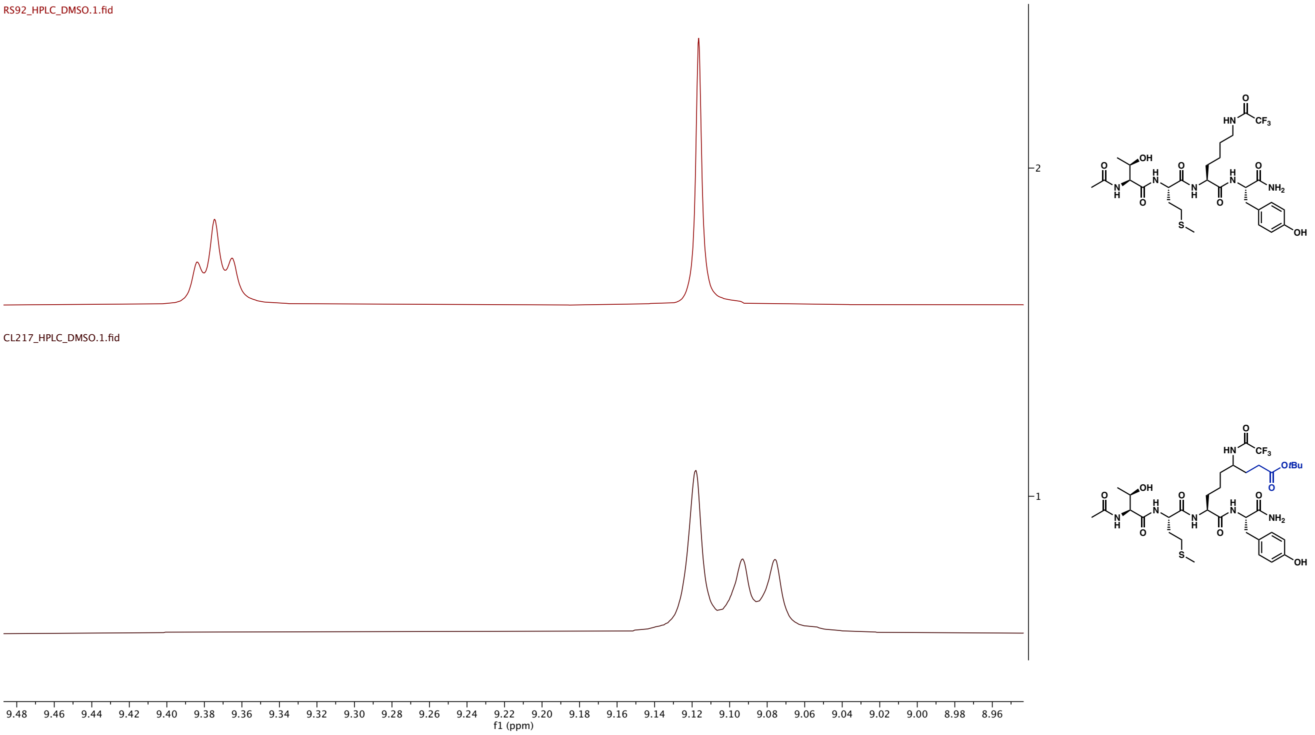
RS92_HPLC_DMSO.1.fid
CL217_HPLC_DMSO.1.fid
2
1
HN
CF3
OH
NH2
OH
S
OtBu
9.48 9.46 9.44 9.42 9.40 9.38 9.36 9.34 9.32 9.30 9.28 9.26 9.24 9.22 9.20 9.18 9.16 9.14 9.12 9.10 9.08 9.06 9.04 9.02 9.00 8.98 8.96
f1 (ppm)

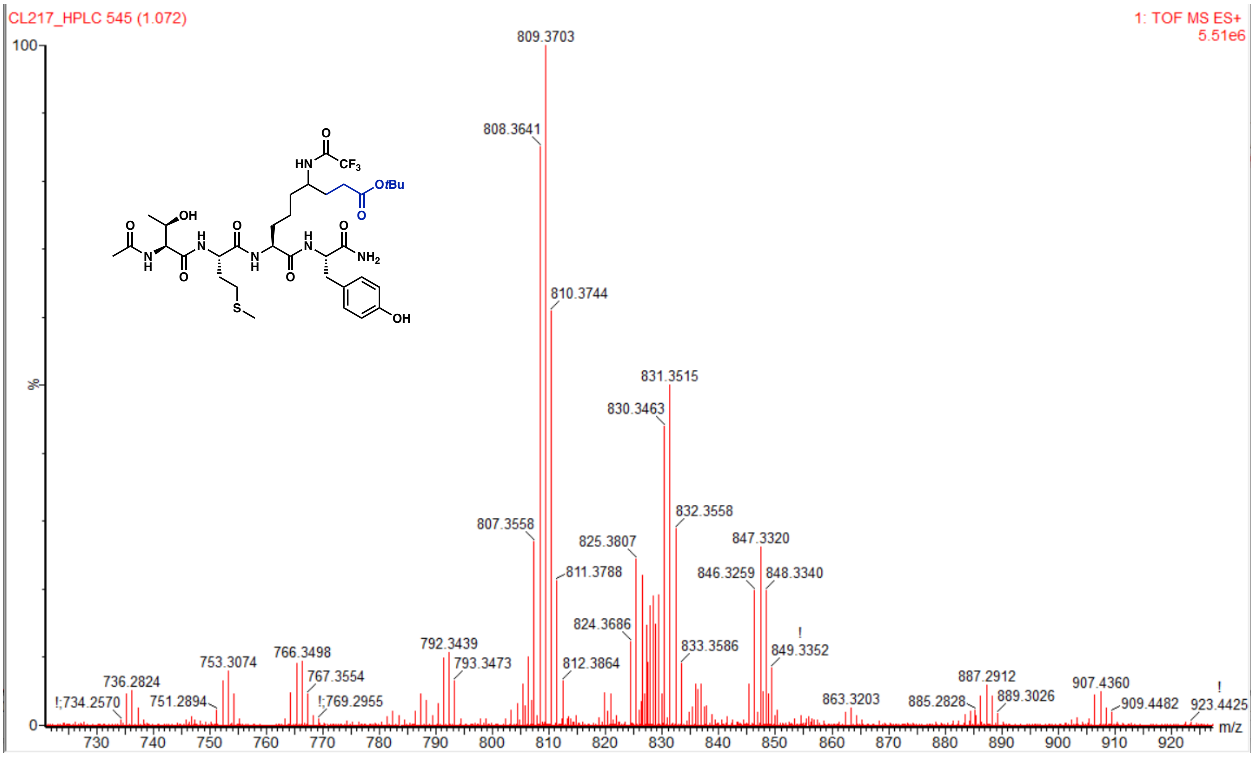
CL217_HPLC 545 (1.072)
1: TOF MS ES+
5.51e6
100
%
0
809.3703
808.3641
810.3744
831.3515
830.3463
807.3558
832.3558
825.3807
847.3320
811.3788
846.3259
848.3340
824.3686
833.3586
849.3352
792.3439
793.3473
812.3864
753.3074
766.3498
767.3554
736.2824
734.2570
751.2894
769.2955
863.3203
885.2828
887.2912
889.3026
907.4360
909.4482
923.4425
730 740 750 760 770 780 790 800 810 820 830 840 850 860 870 880 890 900 910 920
m/z
HN
CF3
OtBu
OH
NH2
OH
S

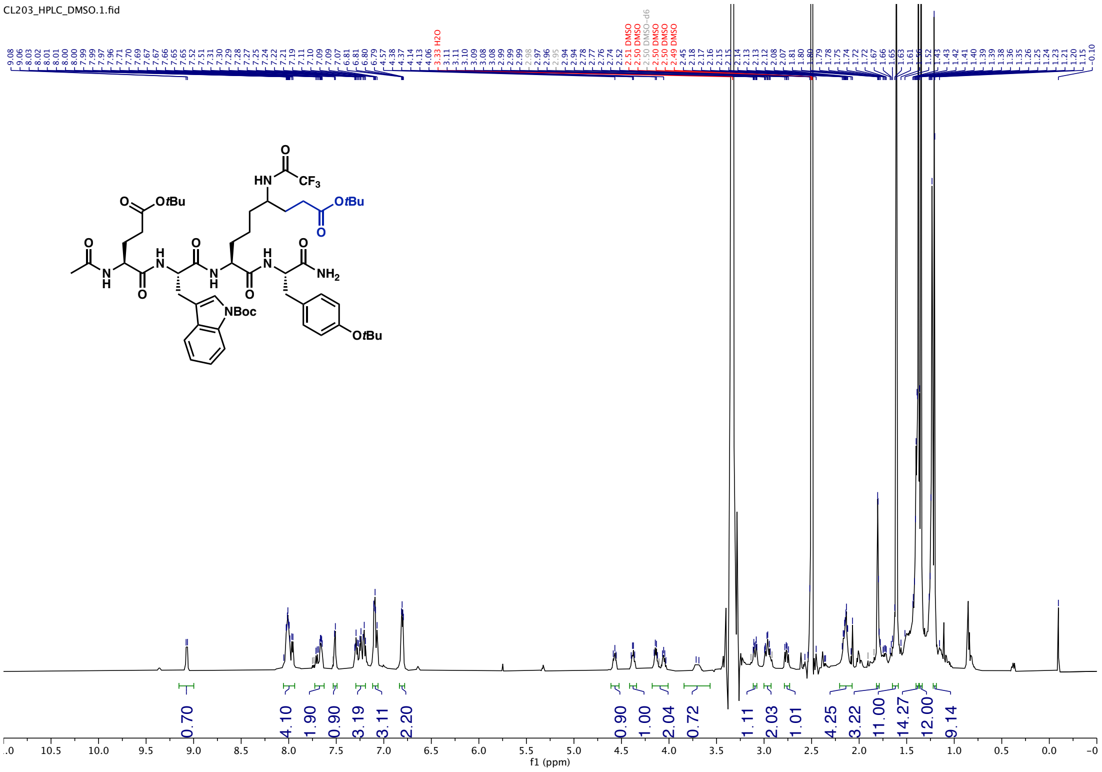
CL203_HPLC_DMSO.1.fid
NBoc
OtBu
NH2
f1 (ppm)

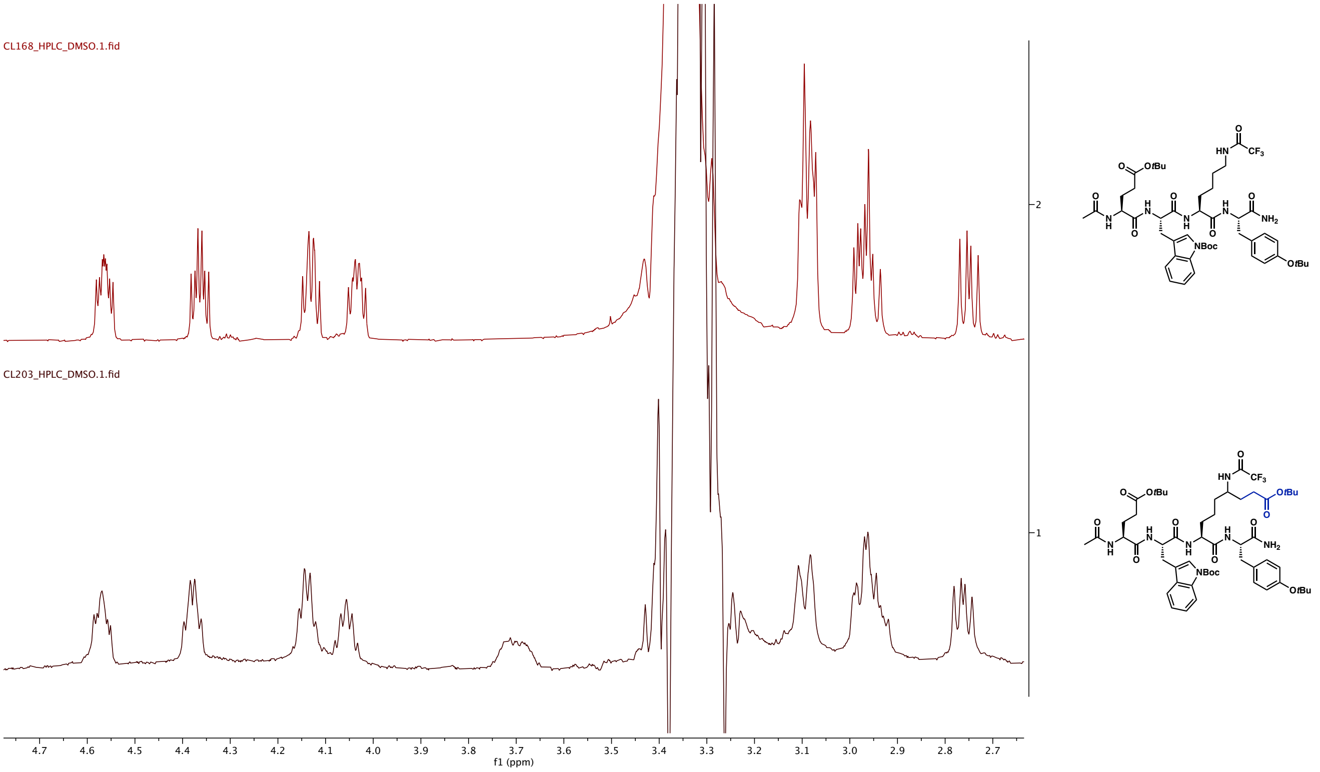
CL168_HPLC_DMSO.1.fid
CL203_HPLC_DMSO.1.fid
f1 (ppm)

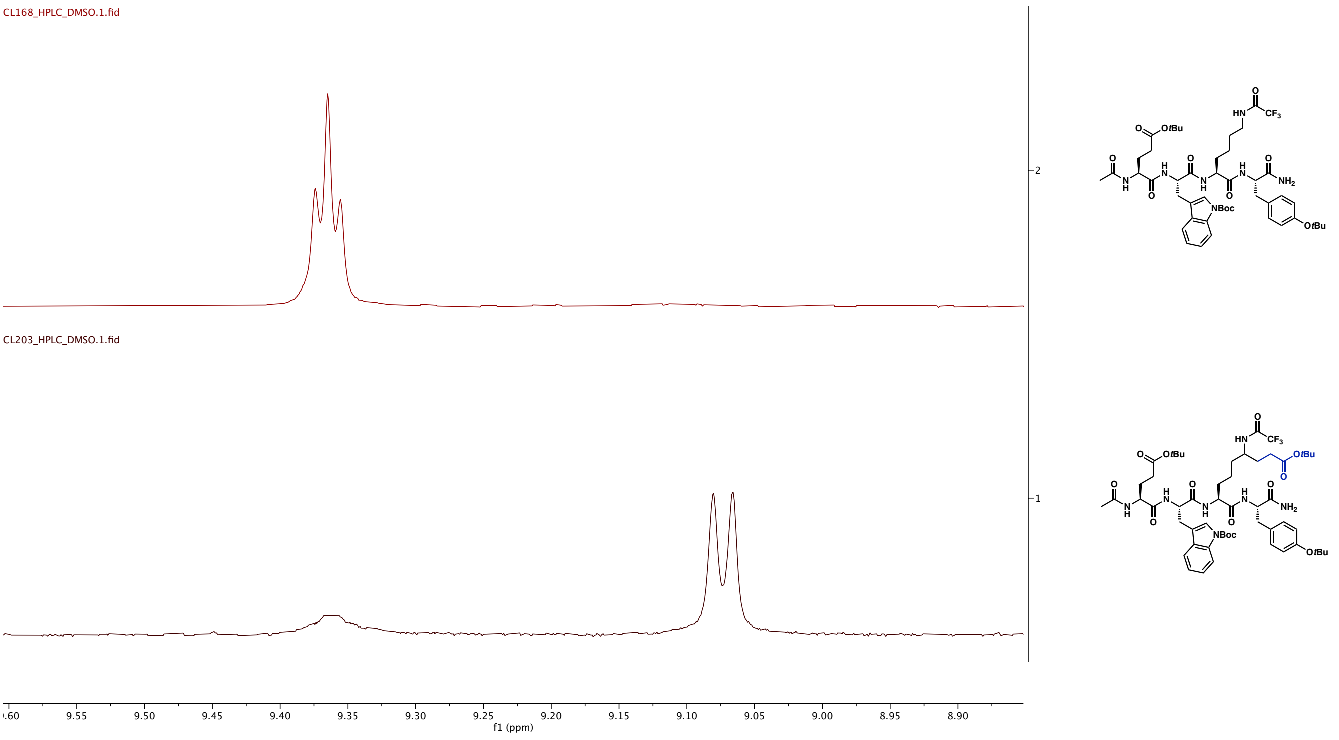

CL168_HPLC_DMSO.1.fid
CL203_HPLC_DMSO.1.fid
f1 (ppm)

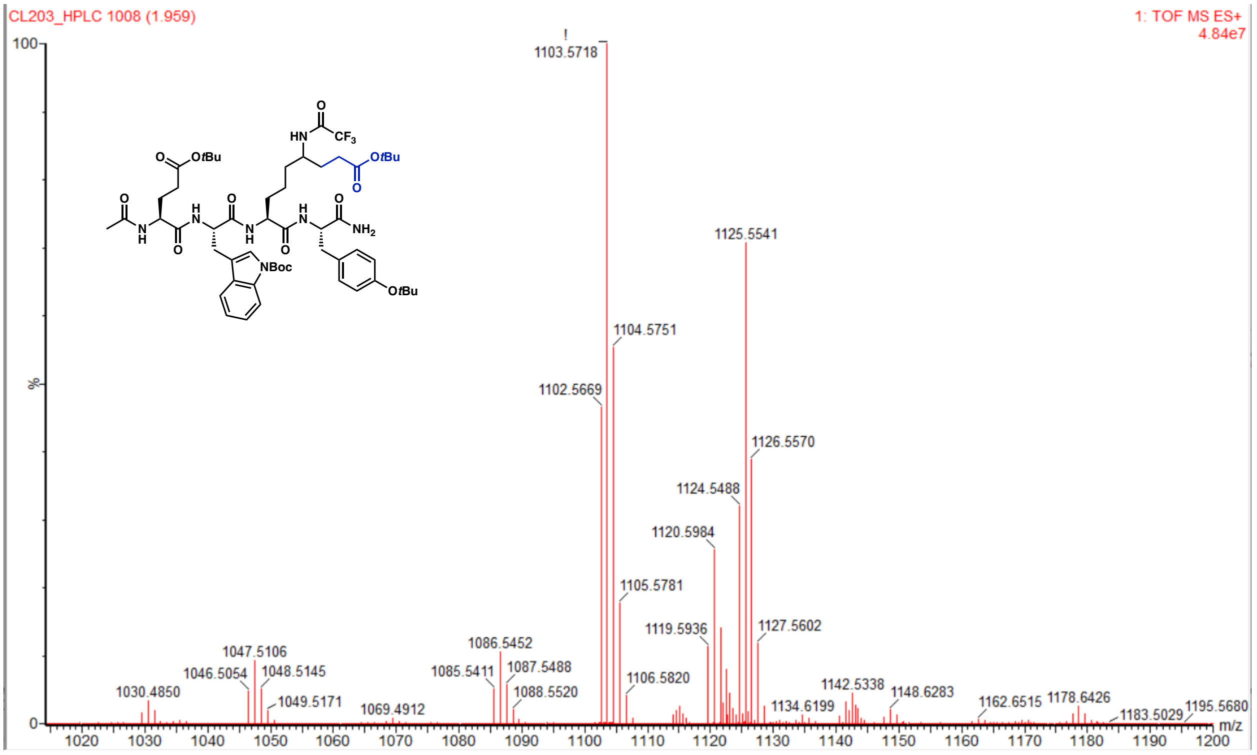

CL203_HPLC 1008 (1.959)
1: TOF MS ES+
4.84e7
1103.5718
1125.5541
1104.5751
1102.5669
1126.5570
1124.5488
1120.5984
1105.5781
1119.5936
1127.5602
1086.5452
1047.5106
1106.5820
m/z

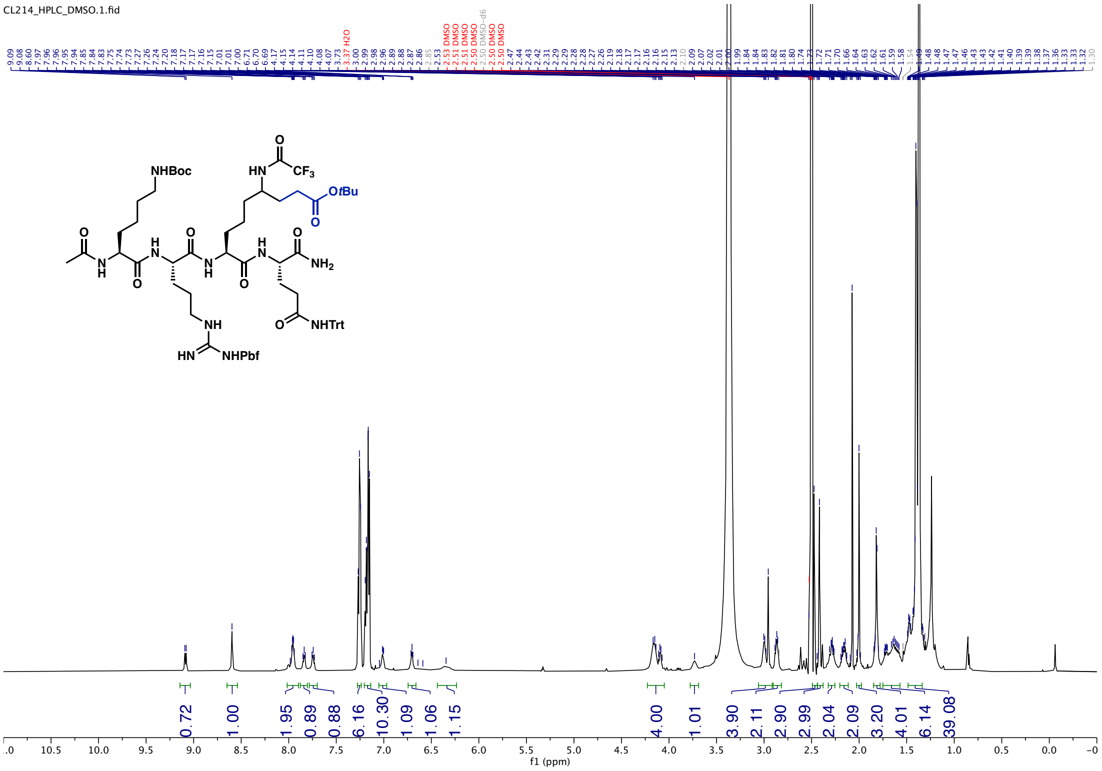
CL214_HPLC_DMSO.1.fid
3.37 H2O
NHBoc
HN
CF3
OtBu
NH2
NHTrt
HN
NHPbf
0.72
1.00
1.95
0.89
0.88
6.16
10.30
1.09
1.06
1.15
4.00
1.01
3.90
2.11
2.90
2.99
2.04
2.09
3.20
4.01
6.14
39.08
f1 (ppm)

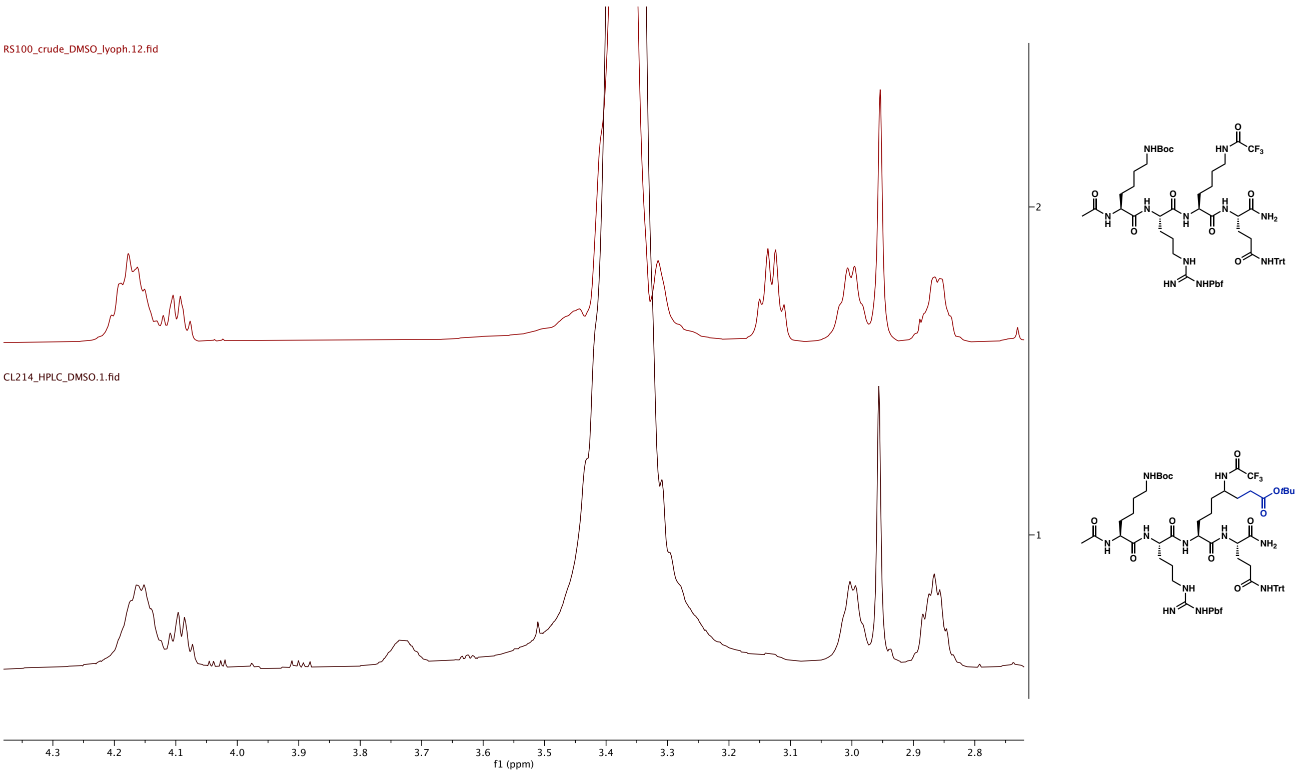
RS100_crude_DMSO_lyoph.12.fid
CL214_HPLC_DMSO.1.fid
f1 (ppm)

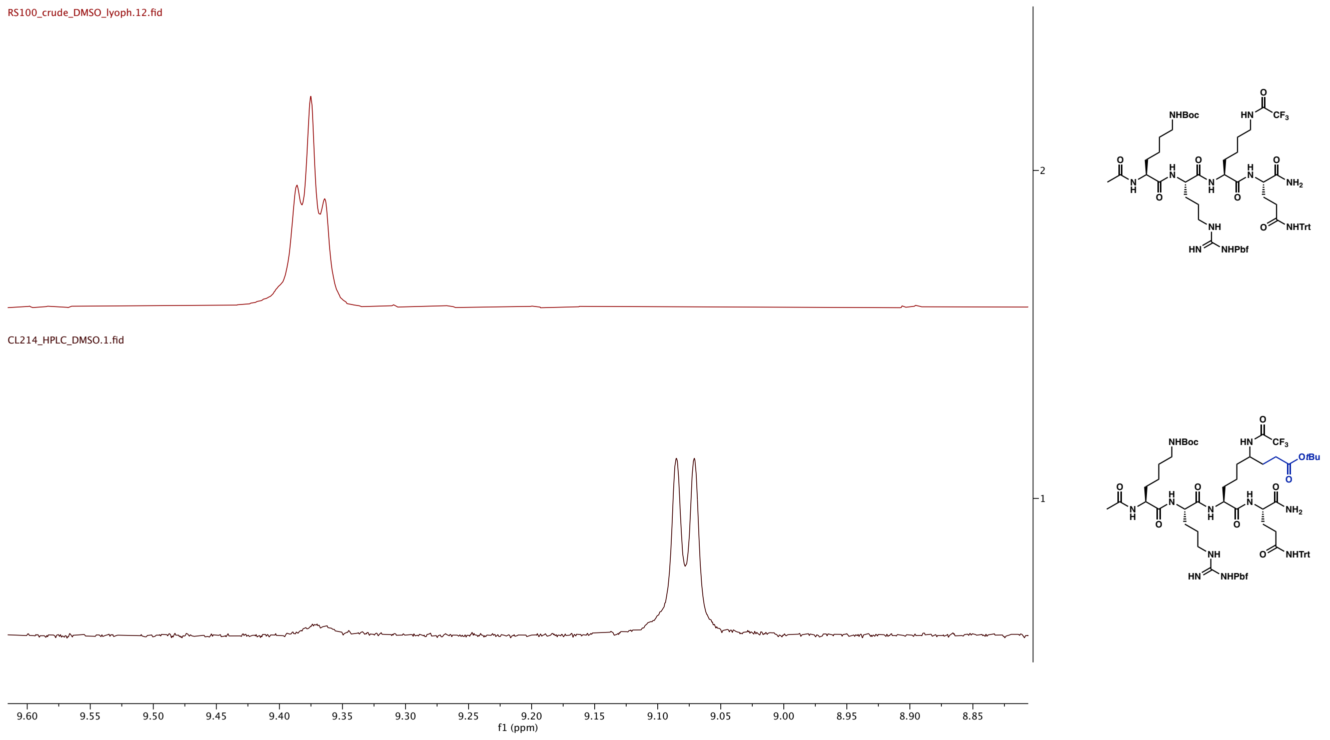

RS100_crude_DMSO_lyoph.12.fid
CL214_HPLC_DMSO.1.fid
9.60
9.55
9.50
9.45
9.40
9.35
9.30
9.25
9.20
9.15
9.10
9.05
9.00
8.95
8.90
8.85
f1 (ppm)
NHBoc
HN
CF3
NH2
NH
HN
NHPbf
NHTrt
OtBu

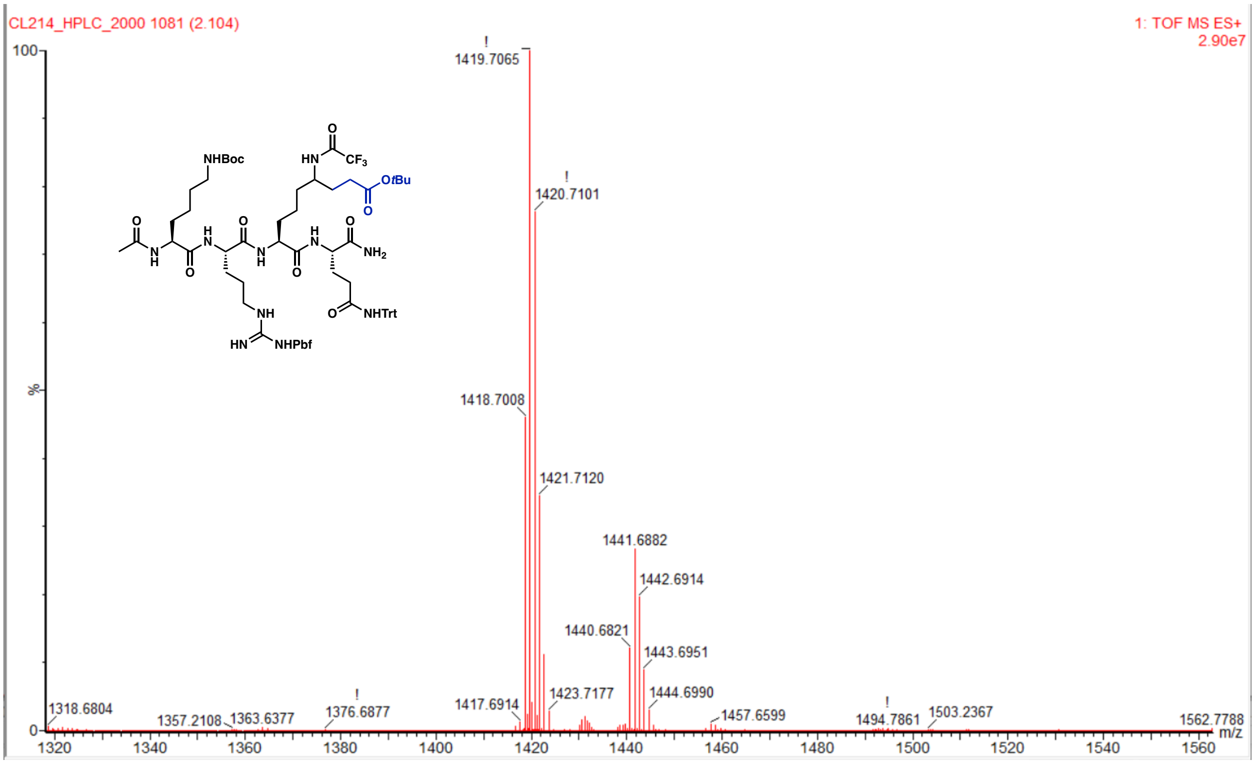

CL214_HPLC_2000 1081 (2.104)
1: TOF MS ES+
2.90e7
100
%
0
1419.7065
1420.7101
1418.7008
1421.7120
1441.6882
1442.6914
1440.6821
1443.6951
1444.6990
1423.7177
1417.6914
1318.6804
1357.2108
1363.6377
1376.6877
1457.6599
1494.7861
1503.2367
1562.7788
m/z
1320
1340
1360
1380
1400
1420
1440
1460
1480
1500
1520
1540
1560
NHBoc
HN
CF3
OtBu
NH2
NH
HN
NHPbf
NHTrt

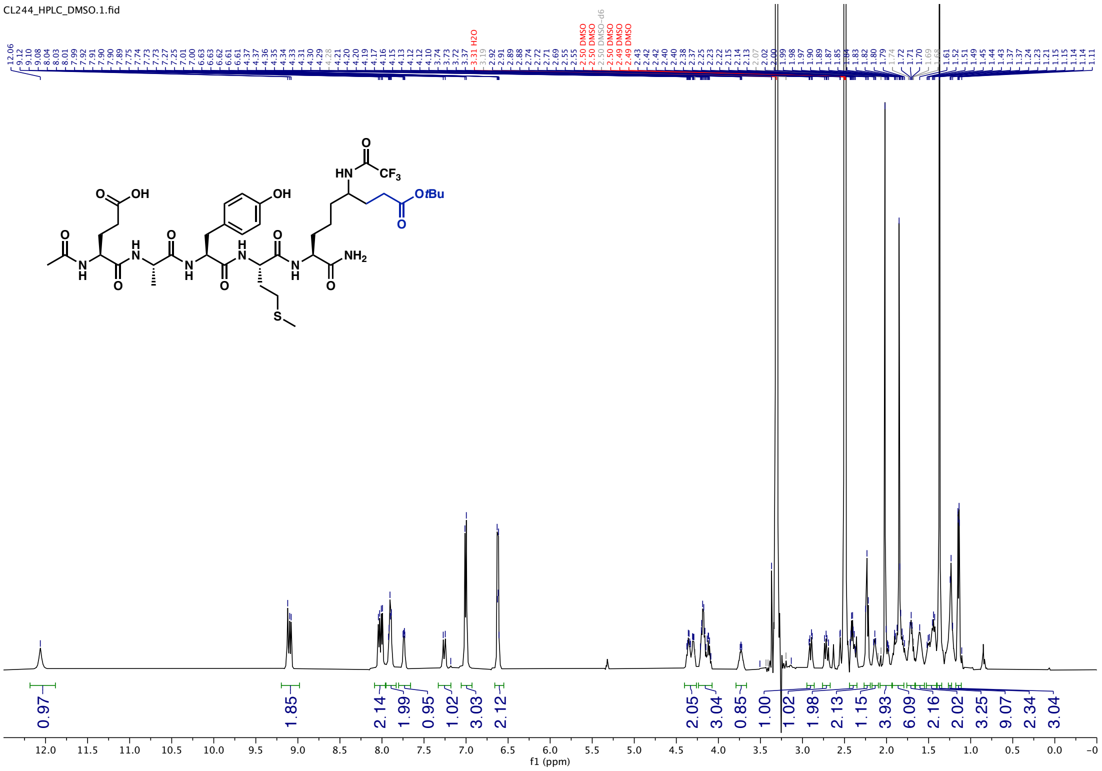
CL244_HPLC_DMSO.1.fid
f1 (ppm)

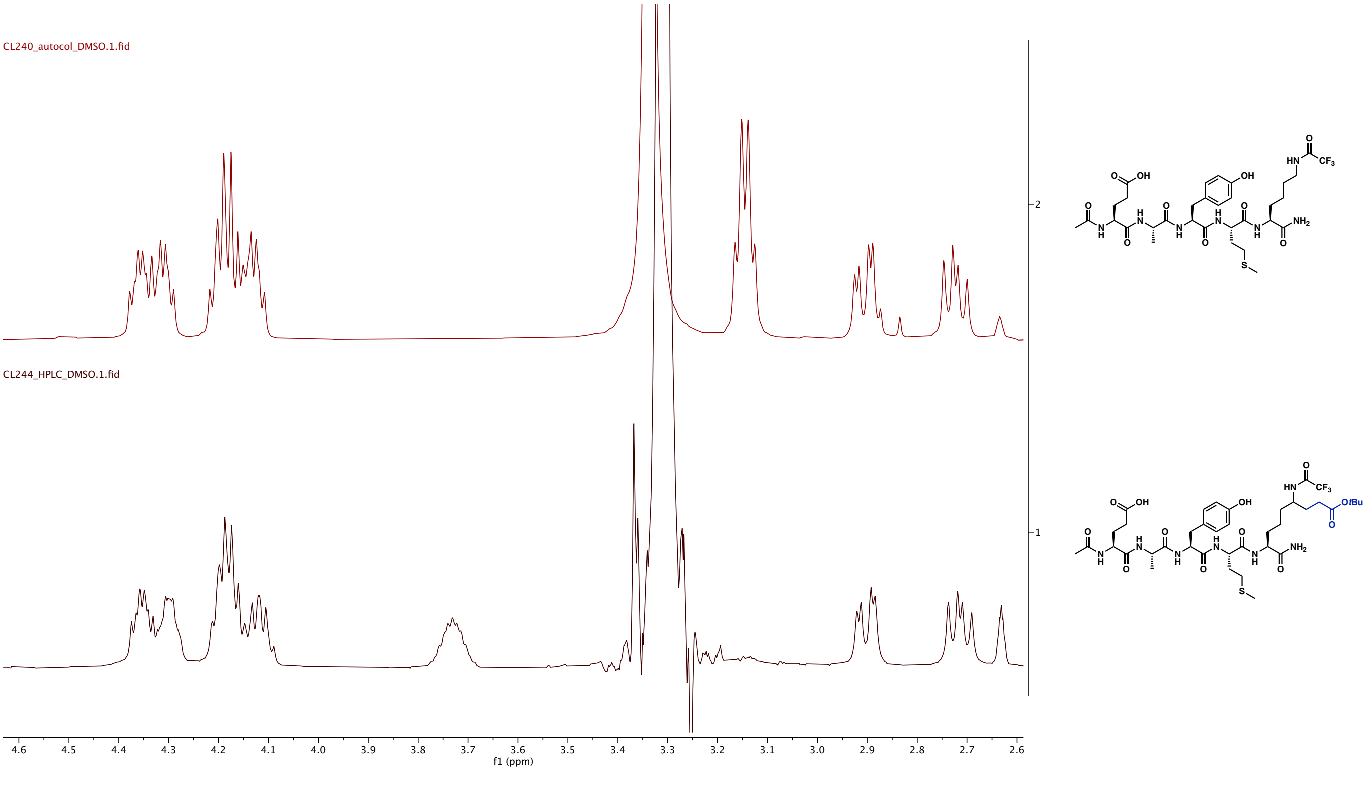
CL240_autocol_DMSO.1.fid
CL244_HPLC_DMSO.1.fid
f1 (ppm)

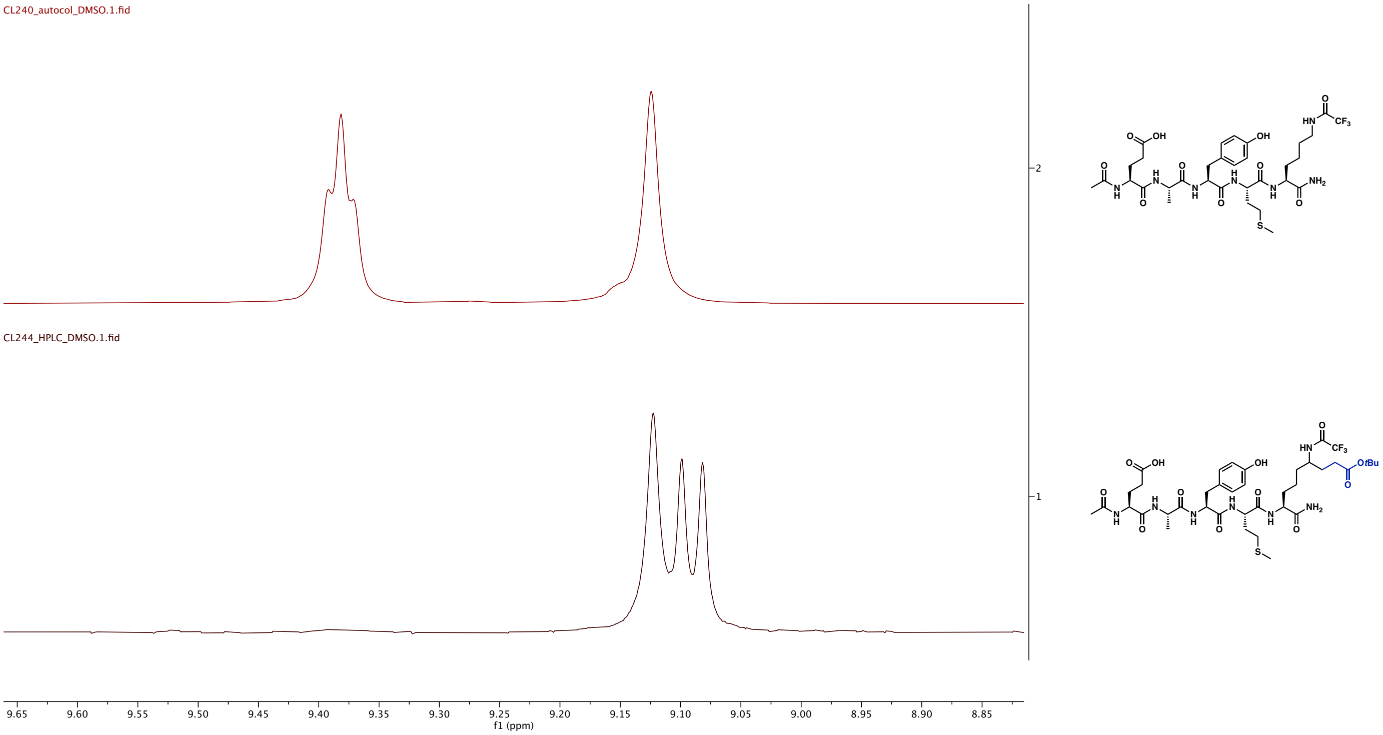
CL240_autocol_DMSO.1.fid
CL244_HPLC_DMSO.1.fid
9.65 9.60 9.55 9.50 9.45 9.40 9.35 9.30 9.25 9.20 9.15 9.10 9.05 9.00 8.95 8.90 8.85
f1 (ppm)
2
1
OH
HN
CF3
NH2
OtBu

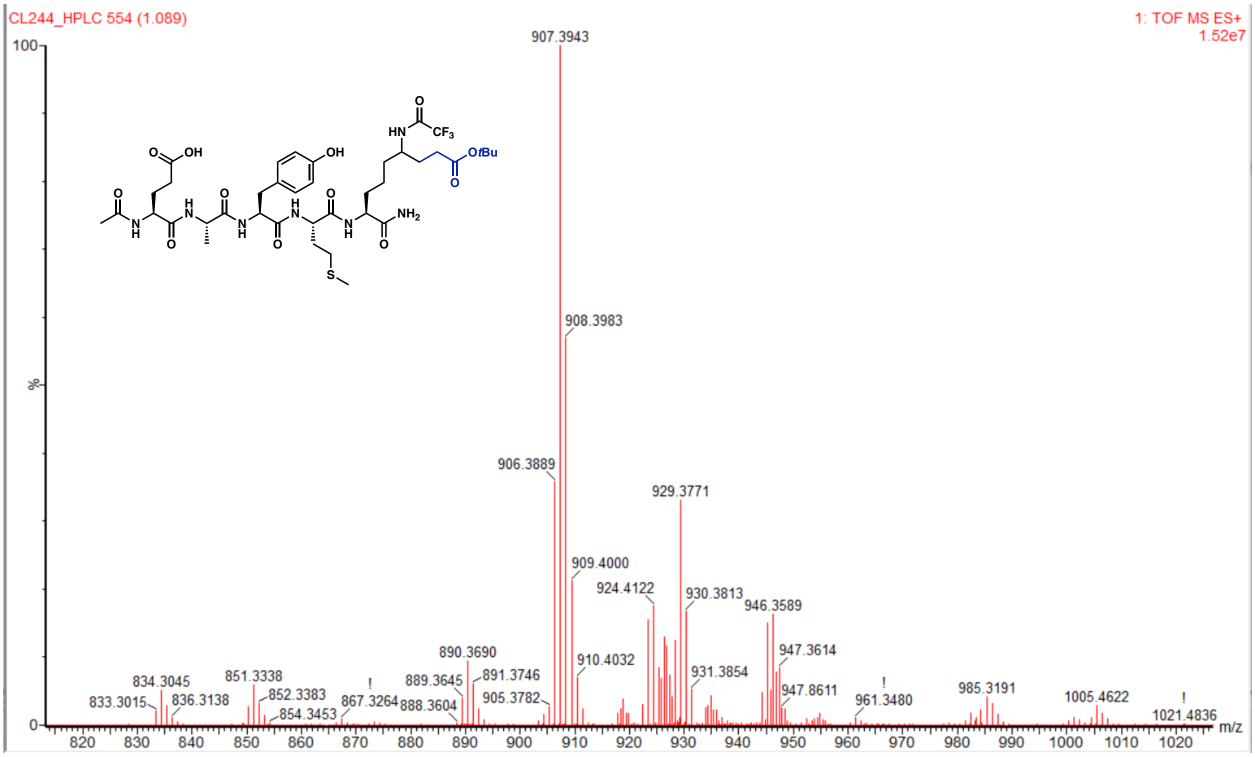
CL244_HPLC 554 (1.089)
1: TOF MS ES+
1.52e7
100
%
0
907.3943
908.3983
906.3889
929.3771
909.4000
924.4122
930.3813
946.3589
890.3690
947.3614
910.4032
931.3854
834.3045
851.3338
889.3645
891.3746
985.3191
833.3015
836.3138
852.3383
867.3264
888.3604
905.3782
947.8611
961.3480
1005.4622
854.3453
1021.4836
820 830 840 850 860 870 880 890 900 910 920 930 940 950 960 970 980 990 1000 1010 1020
m/z
OH
HN
CF3
NH2
OtBu

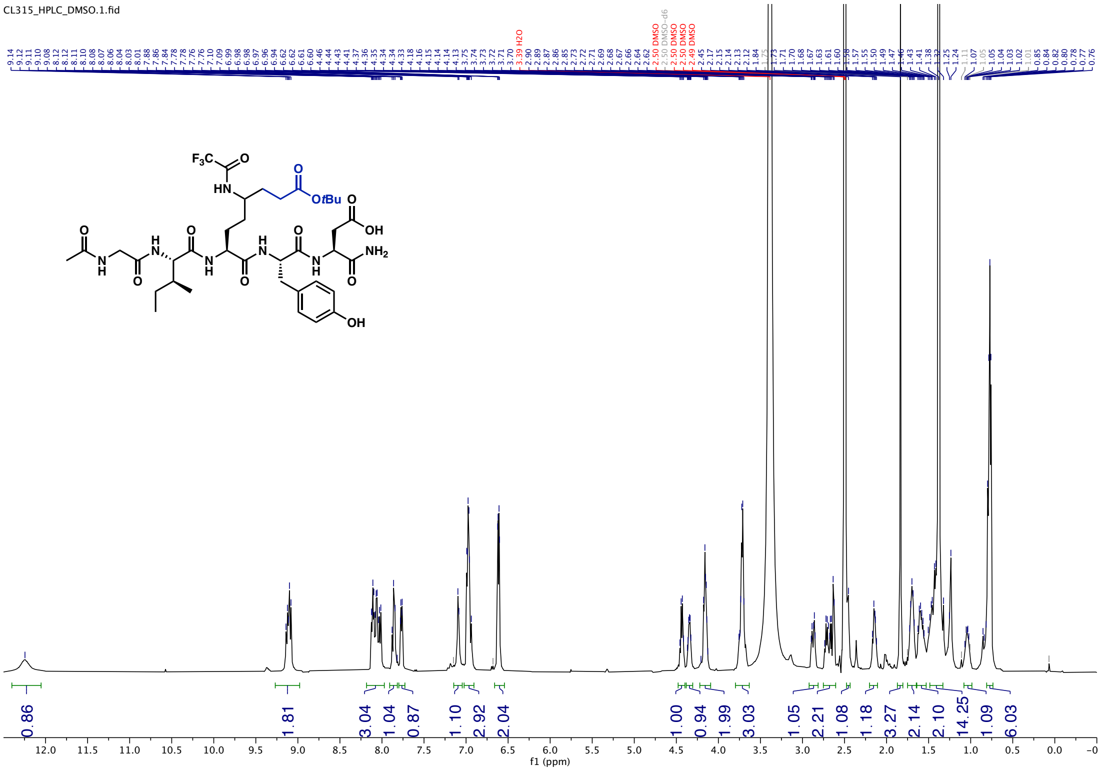
CL315_HPLC_DMSO.1.fid
f1 (ppm)

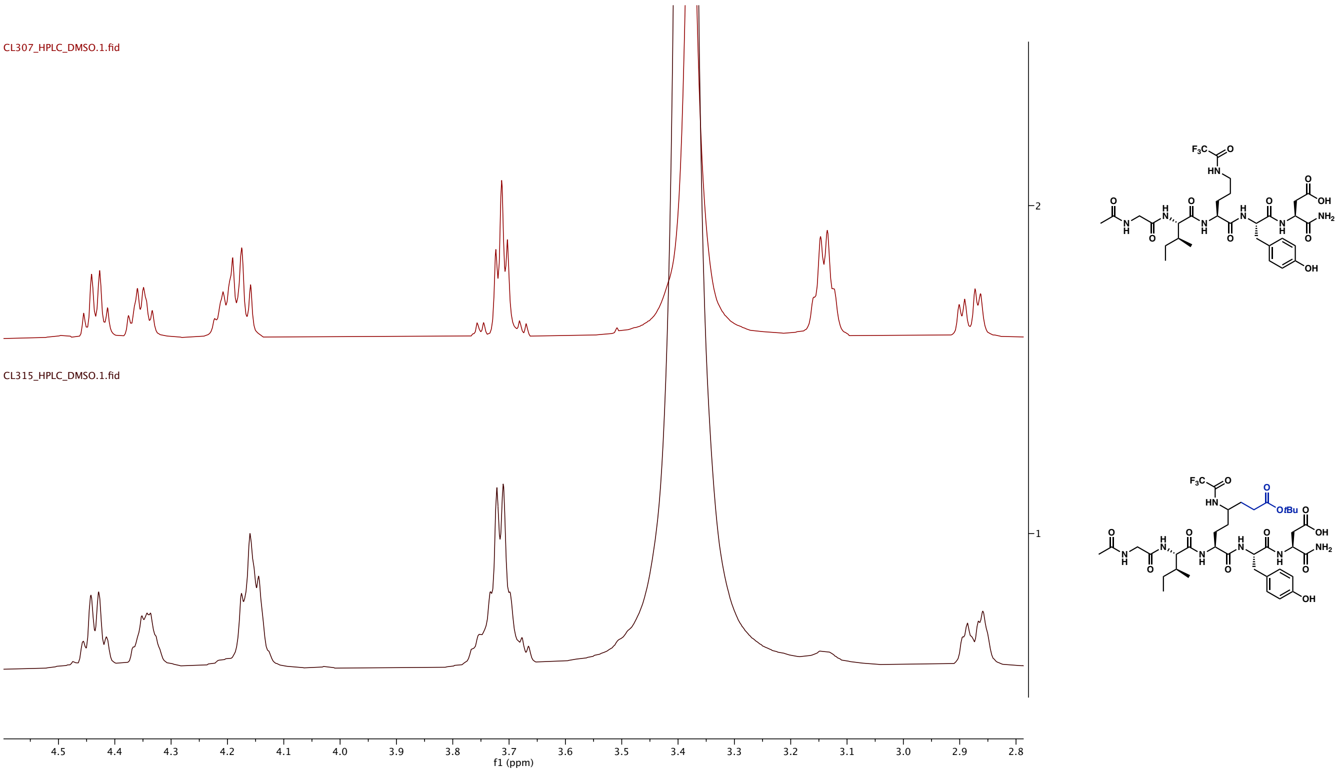
CL307_HPLC_DMSO.1.fid
CL315_HPLC_DMSO.1.fid
f1 (ppm)

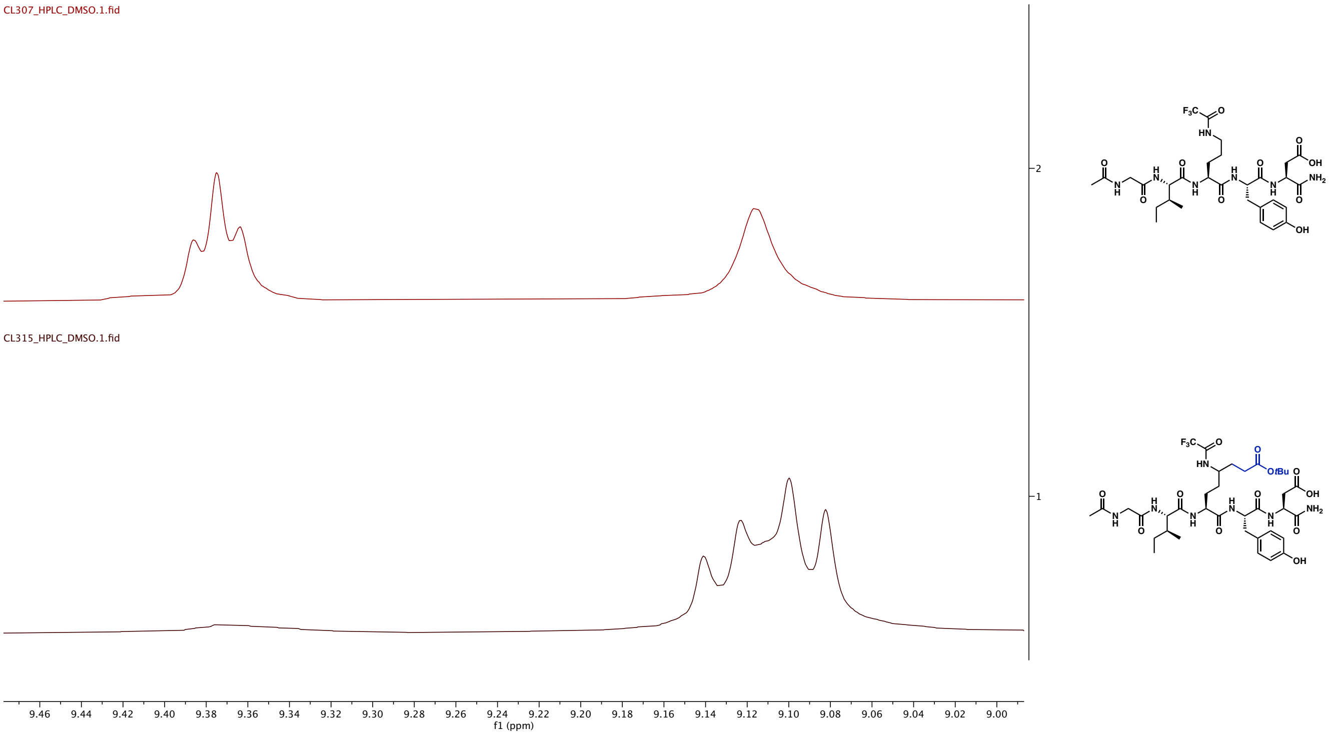
CL307_HPLC_DMSO.1.fid
CL315_HPLC_DMSO.1.fid
f1 (ppm)

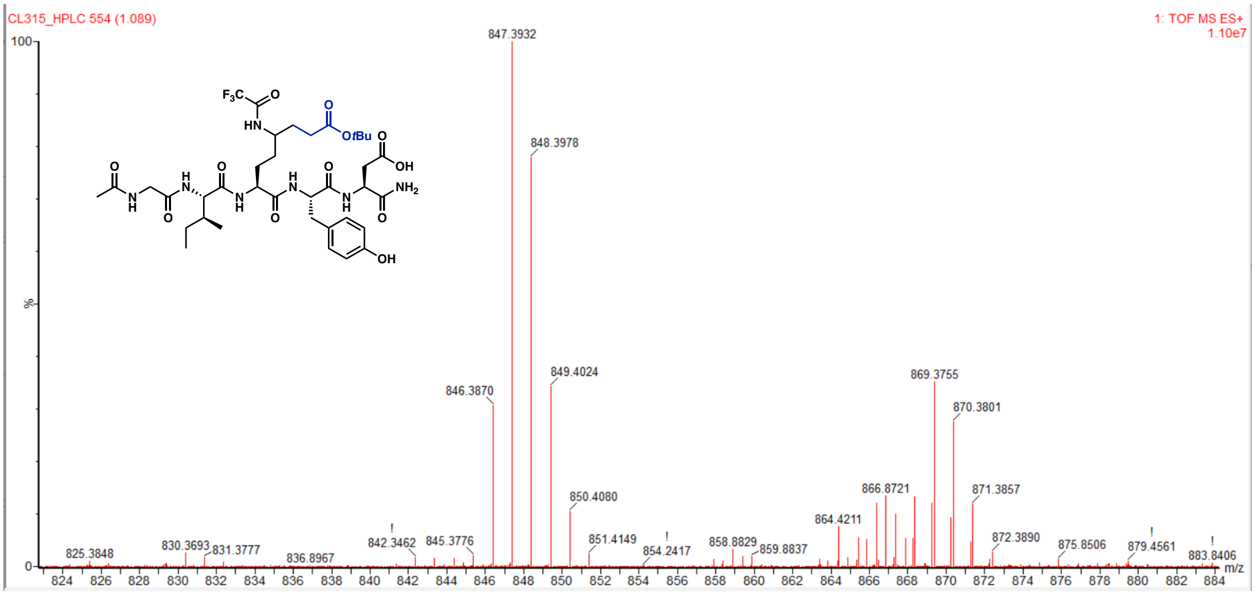
CL315_HPLC 554 (1.089)
1: TOF MS ES+
1.10e7
847.3932
848.3978
849.4024
846.3870
869.3755
870.3801
%
m/z

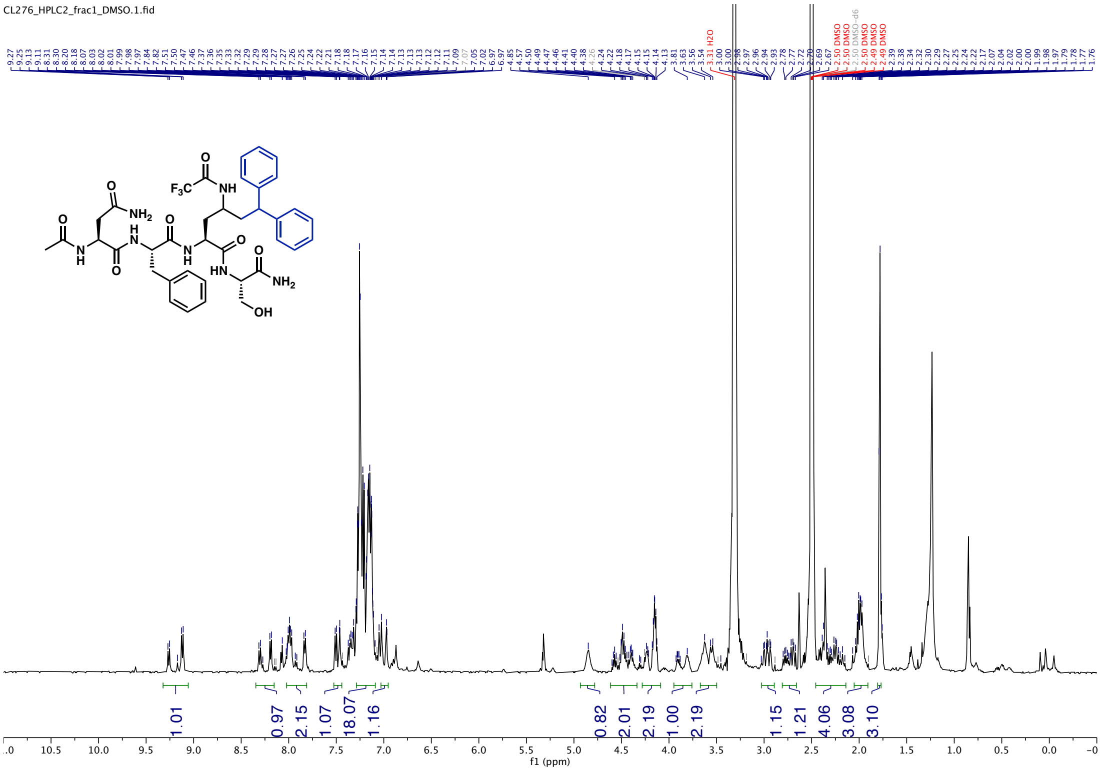
CL276_HPLC2_frac1_DMSO.1.fid
1.01
0.97
2.15
1.07
18.07
1.16
0.82
2.01
2.19
1.00
2.19
1.15
1.21
4.06
3.08
3.10
f1 (ppm)

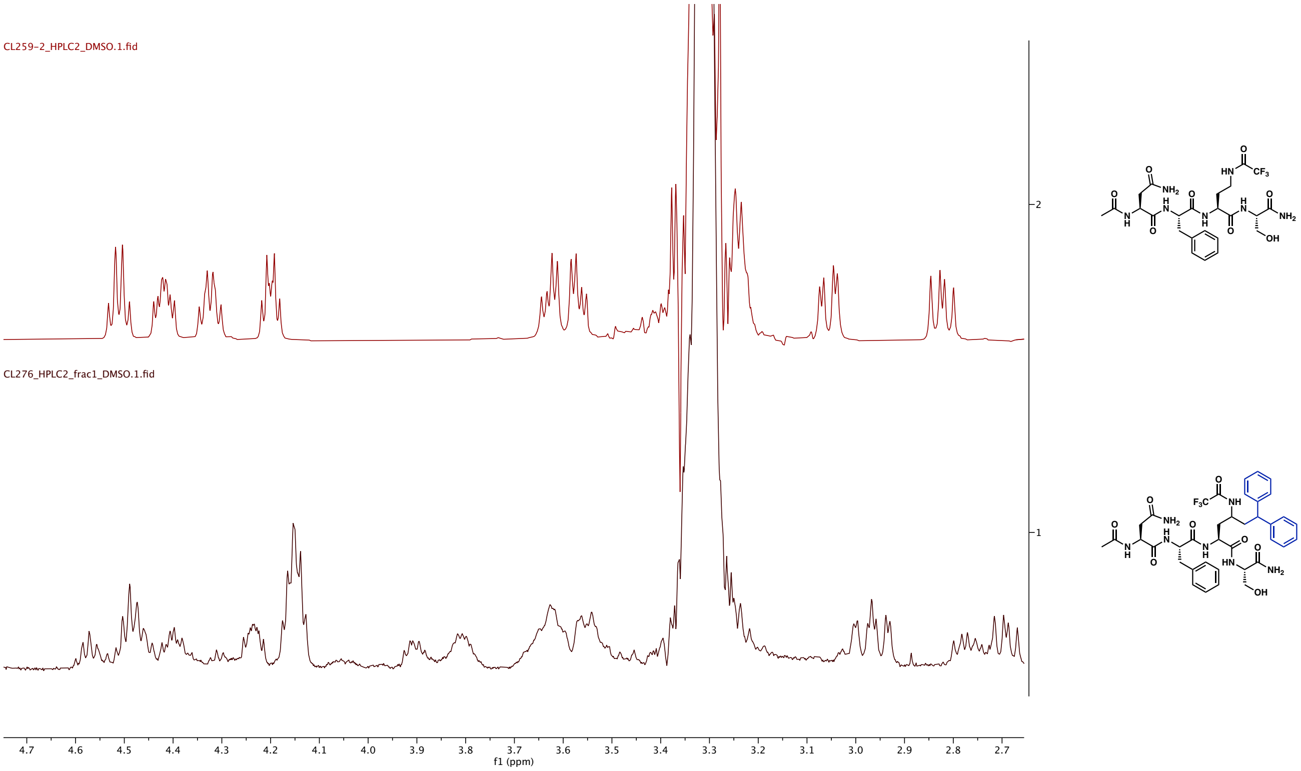
CL259-2_HPLC2_DMSO.1.fid
CL276_HPLC2_frac1_DMSO.1.fid
f1 (ppm)

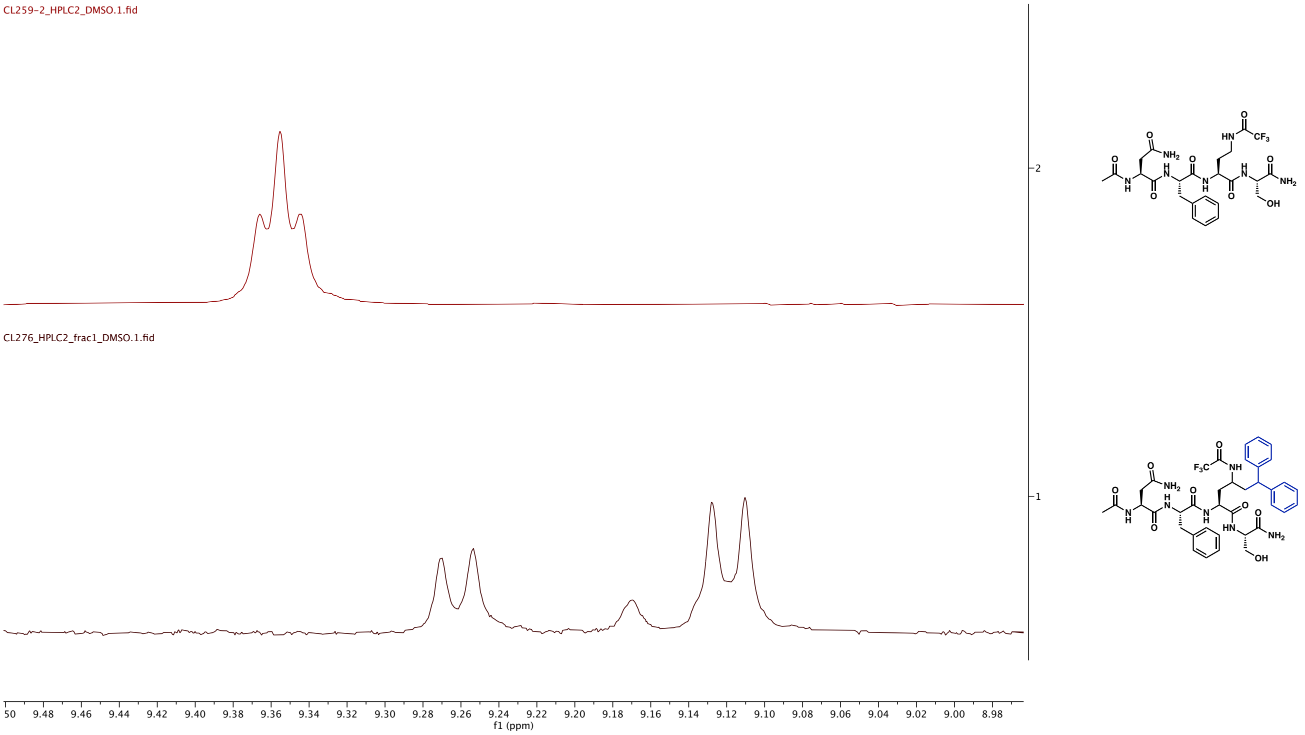
CL259-2_HPLC2_DMSO.1.fid
CL276_HPLC2_frac1_DMSO.1.fid
f1 (ppm)

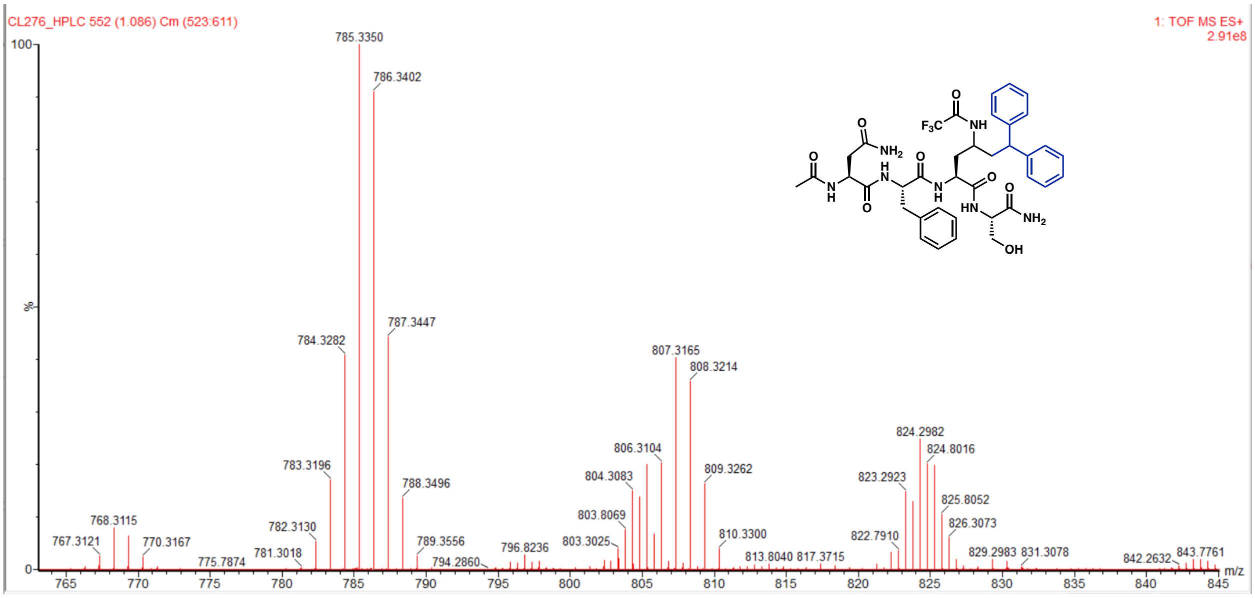
CL276_HPLC 552 (1.086) Cm (523:611)
1: TOF MS ES+
2.91e8
785.3350
786.3402
787.3447
784.3282
807.3165
808.3214
824.2982
824.8016
806.3104
809.3262
783.3196
788.3496
804.3083
823.2923
825.8052
803.8069
826.3073
768.3115
782.3130
810.3300
767.3121
770.3167
789.3556
796.8236
803.3025
822.7910
781.3018
813.8040
817.3715
829.2983
831.3078
775.7874
794.2860
842.2632
843.7761
m/z
%

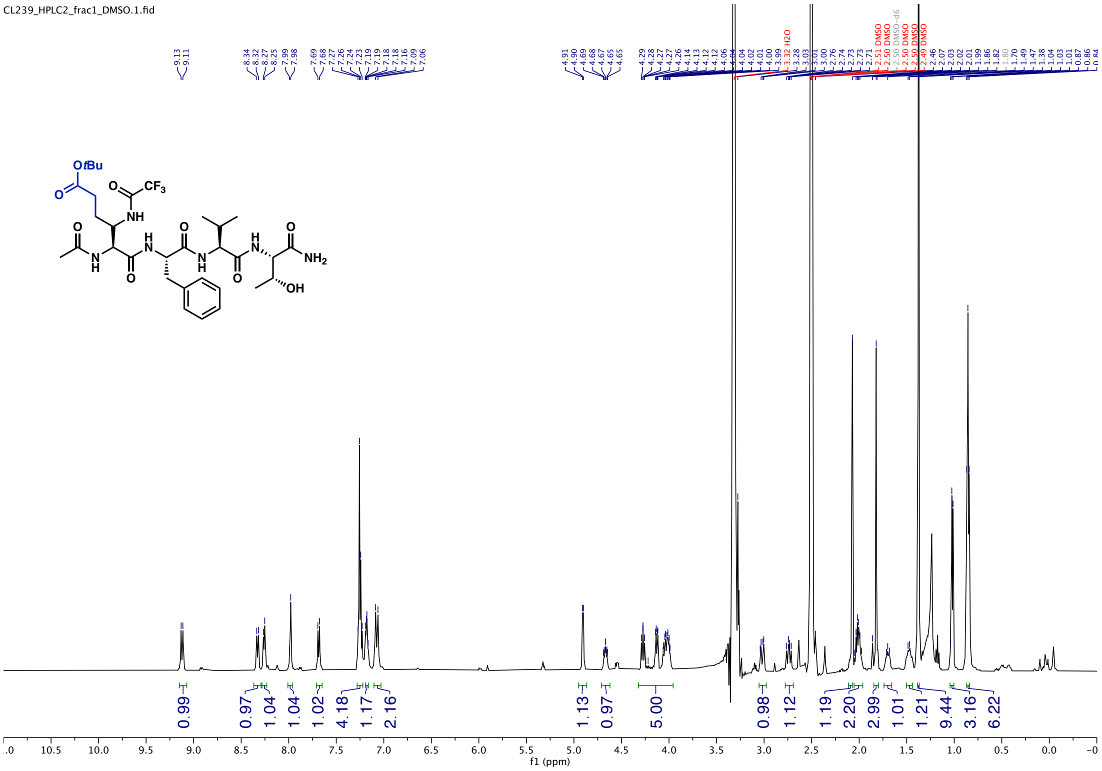
CL239_HPLC2_frac1_DMSO.1.fid
f1 (ppm)

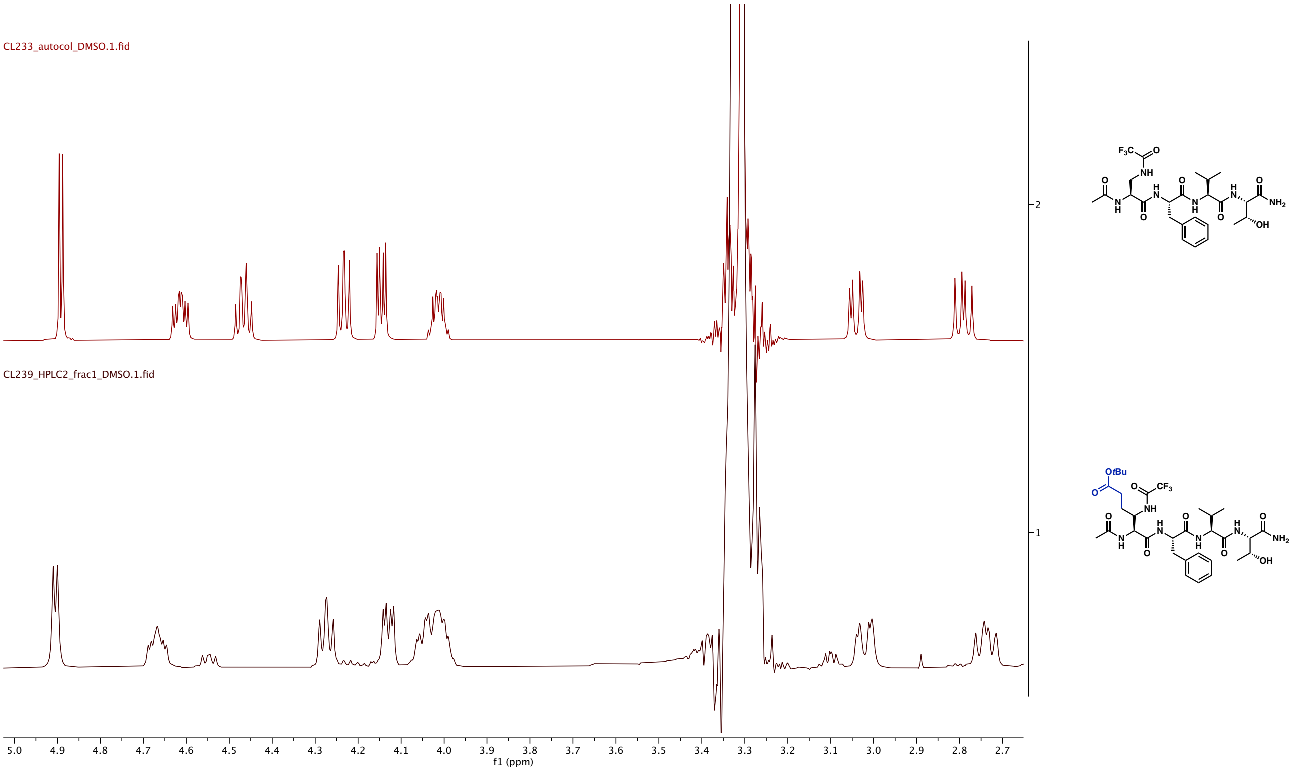
CL233_autocol_DMSO.1.fid
CL239_HPLC2_frac1_DMSO.1.fid
f1 (ppm)

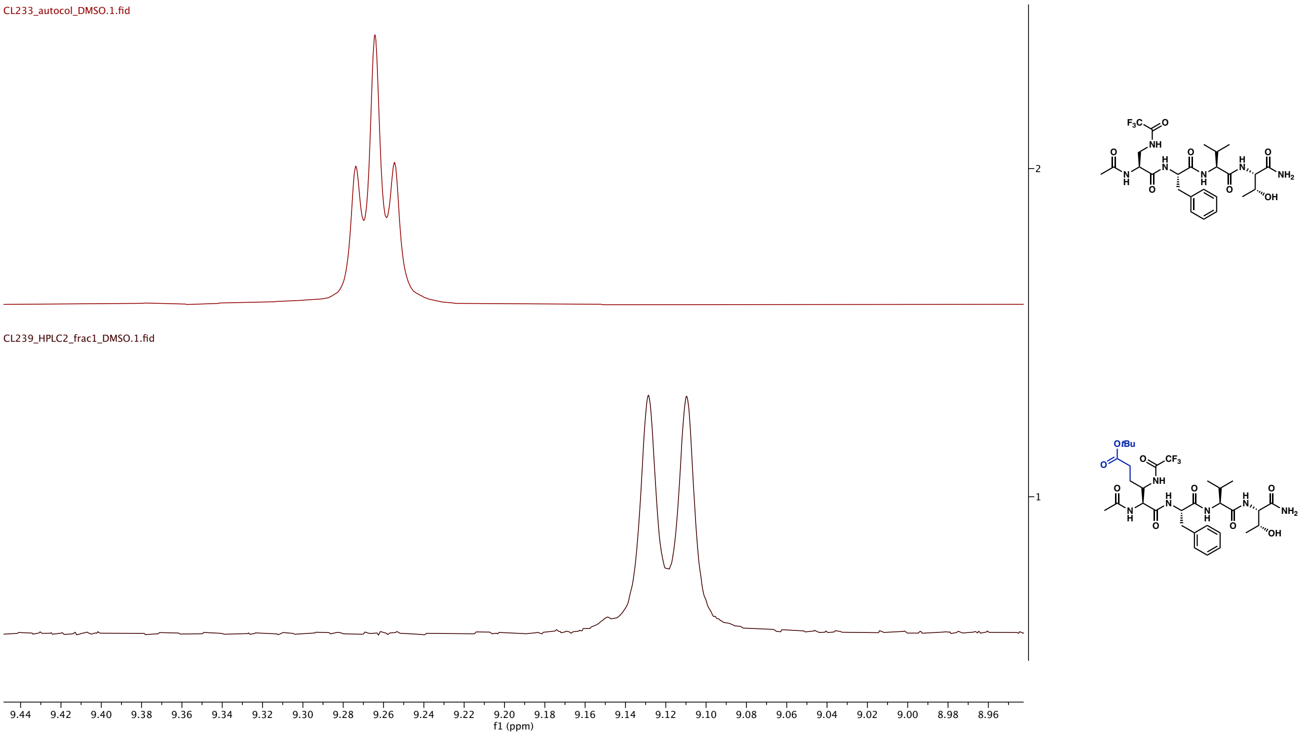
CL233_autocol_DMSO.1.fid
CL239_HPLC2_frac1_DMSO.1.fid
9.44 9.42 9.40 9.38 9.36 9.34 9.32 9.30 9.28 9.26 9.24 9.22 9.20 9.18 9.16 9.14 9.12 9.10 9.08 9.06 9.04 9.02 9.00 8.98 8.96
f1 (ppm)

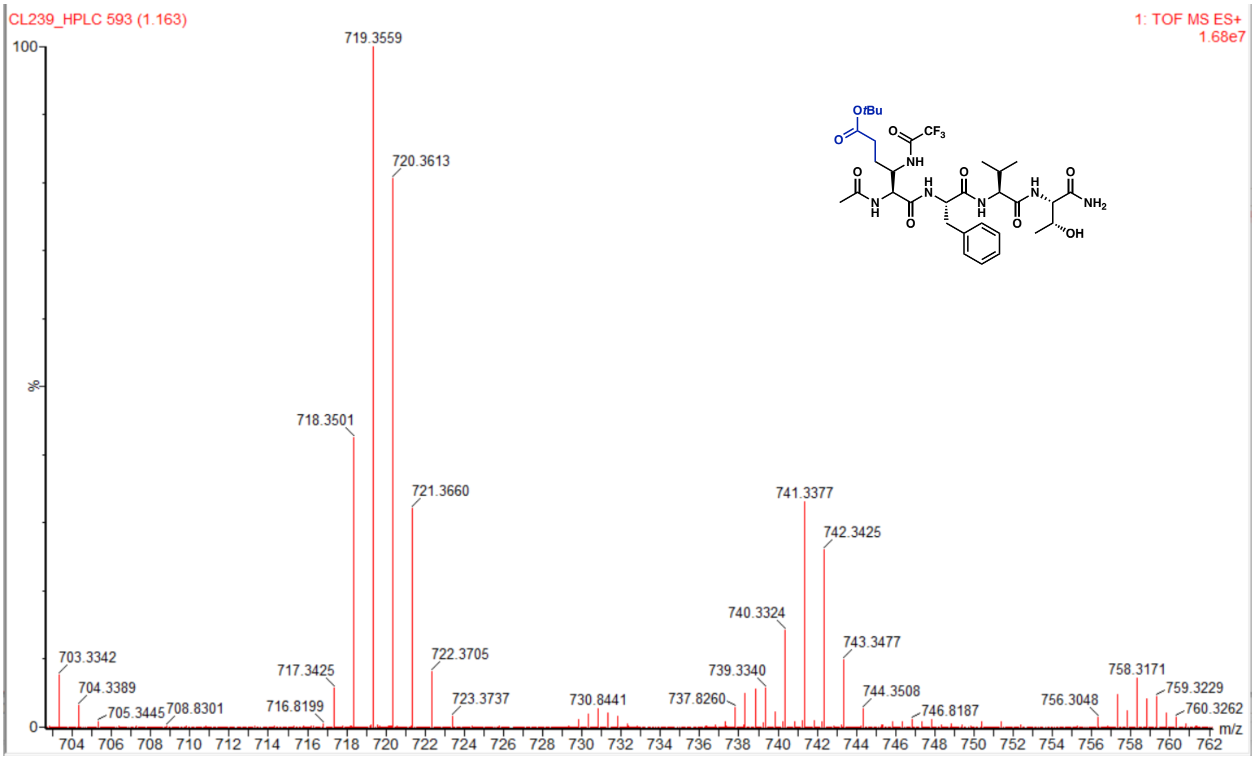
CL239_HPLC 593 (1.163)
1: TOF MS ES+
1.68e7
719.3559
720.3613
718.3501
721.3660
741.3377
742.3425
740.3324
743.3477
739.3340
703.3342
722.3705
717.3425
704.3389
758.3171
759.3229
744.3508
746.8187
756.3048
760.3262
705.3445
708.8301
716.8199
723.3737
730.8441
737.8260
OtBu
CF3
NH2
OH
%
m/z

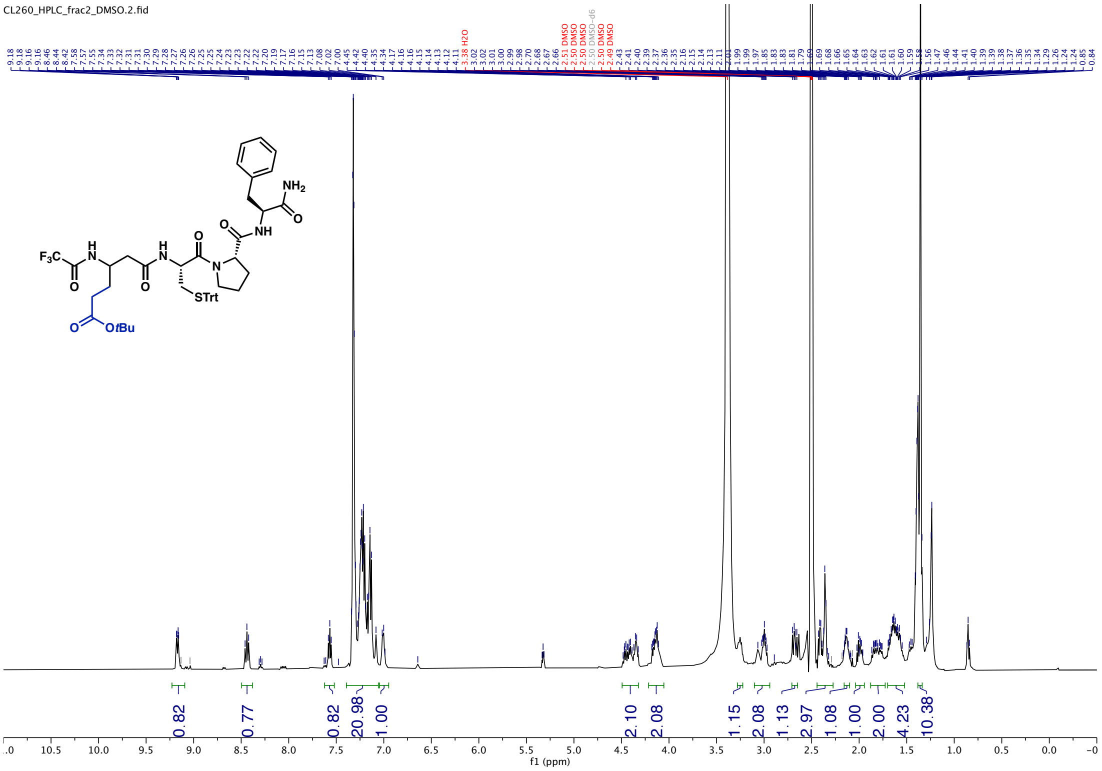

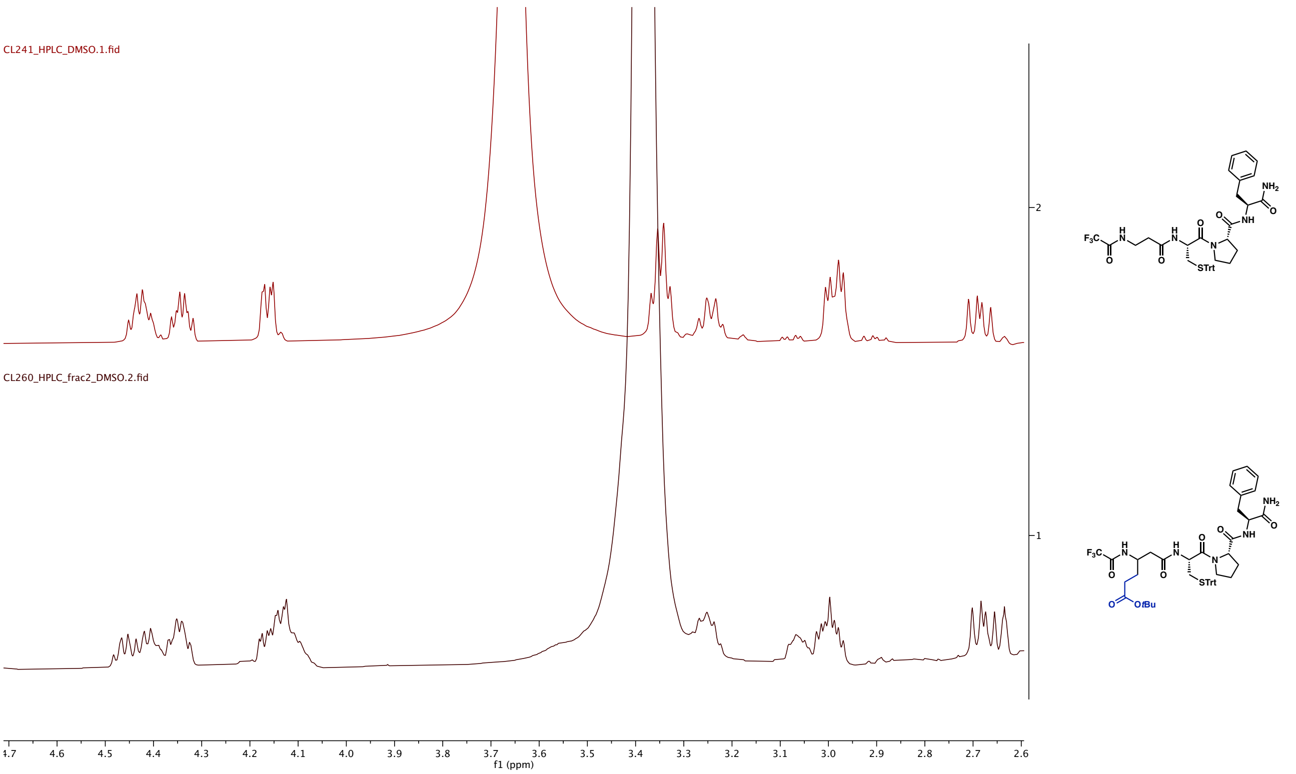

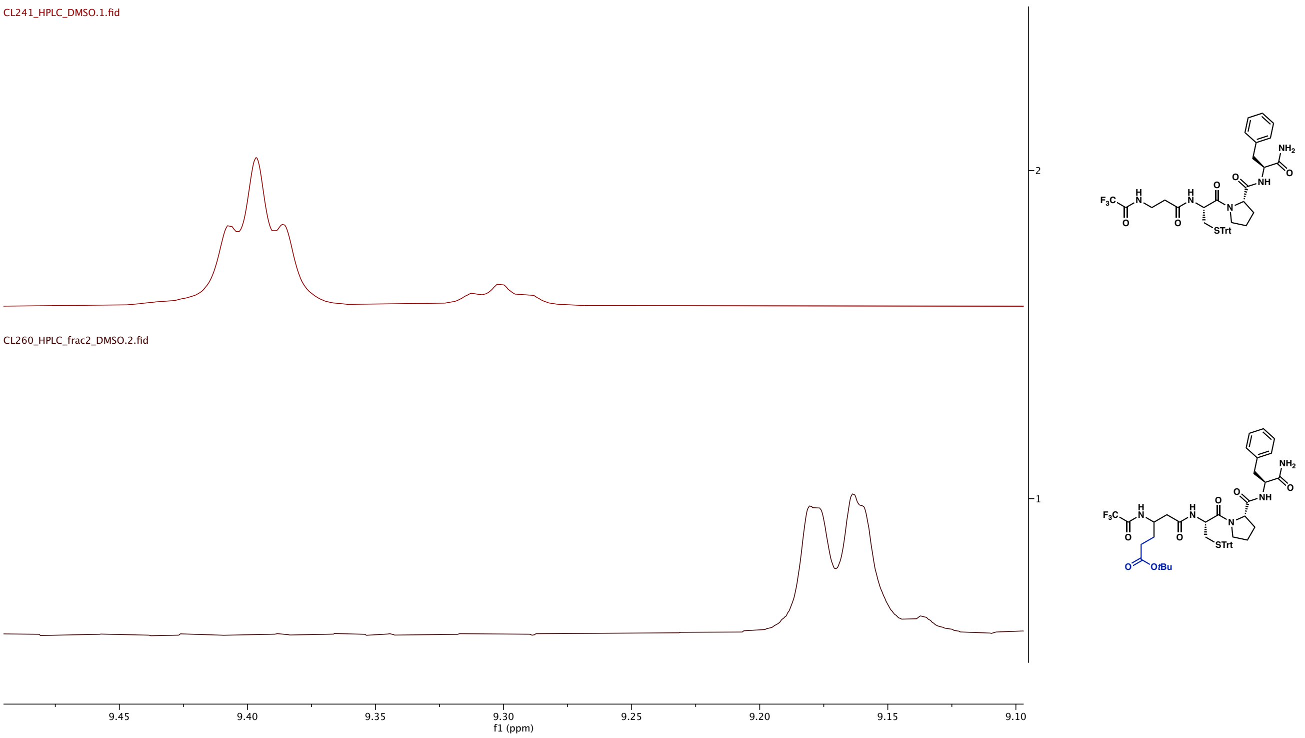
CL241_HPLC_DMSO.1.fid
CL260_HPLC_frac2_DMSO.2.fid
9.45
9.40
9.35
9.30
9.25
9.20
9.15
9.10
f1 (ppm)
STrt
OtBu

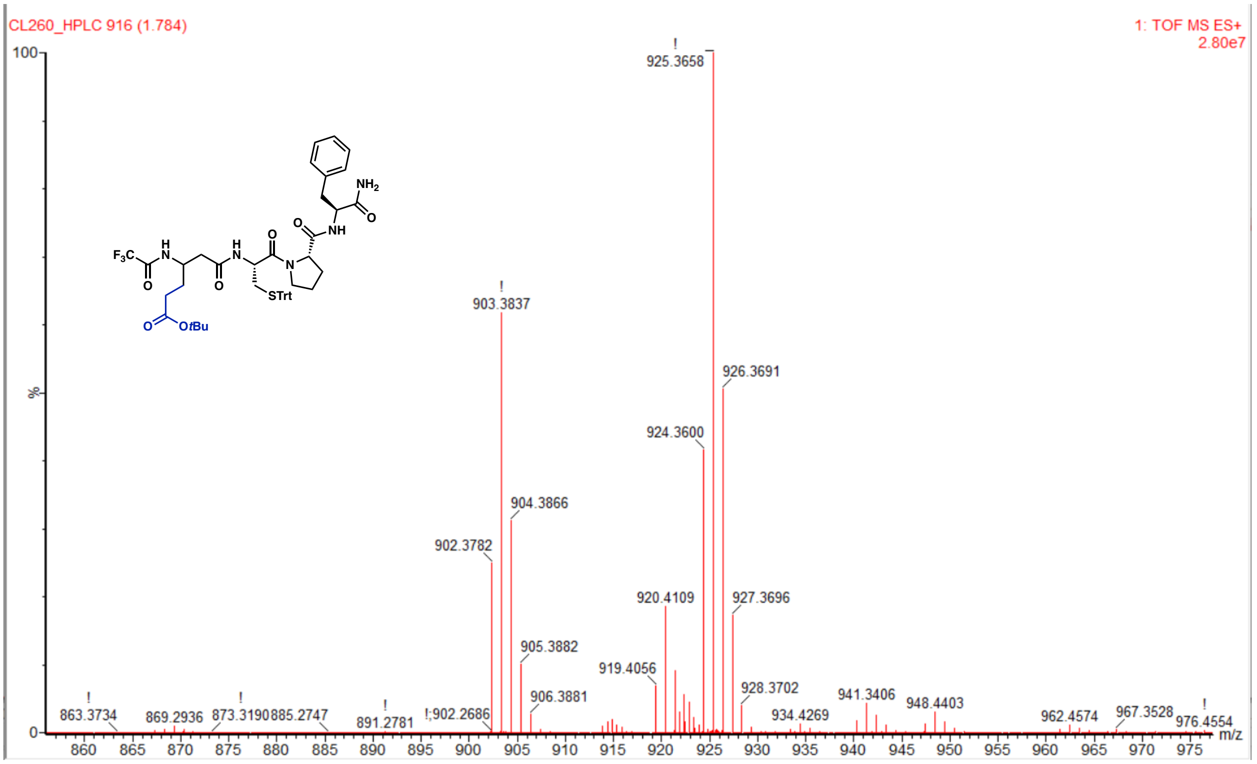
CL260_HPLC 916 (1.784)
1: TOF MS ES+
2.80e7
925.3658
903.3837
926.3691
924.3600
904.3866
902.3782
920.4109
927.3696
905.3882
919.4056
906.3881
928.3702
941.3406
948.4403
863.3734
869.2936
873.3190
885.2747
891.2781
902.2686
934.4269
962.4574
967.3528
976.4554
m/z
%
STrt
OtBu

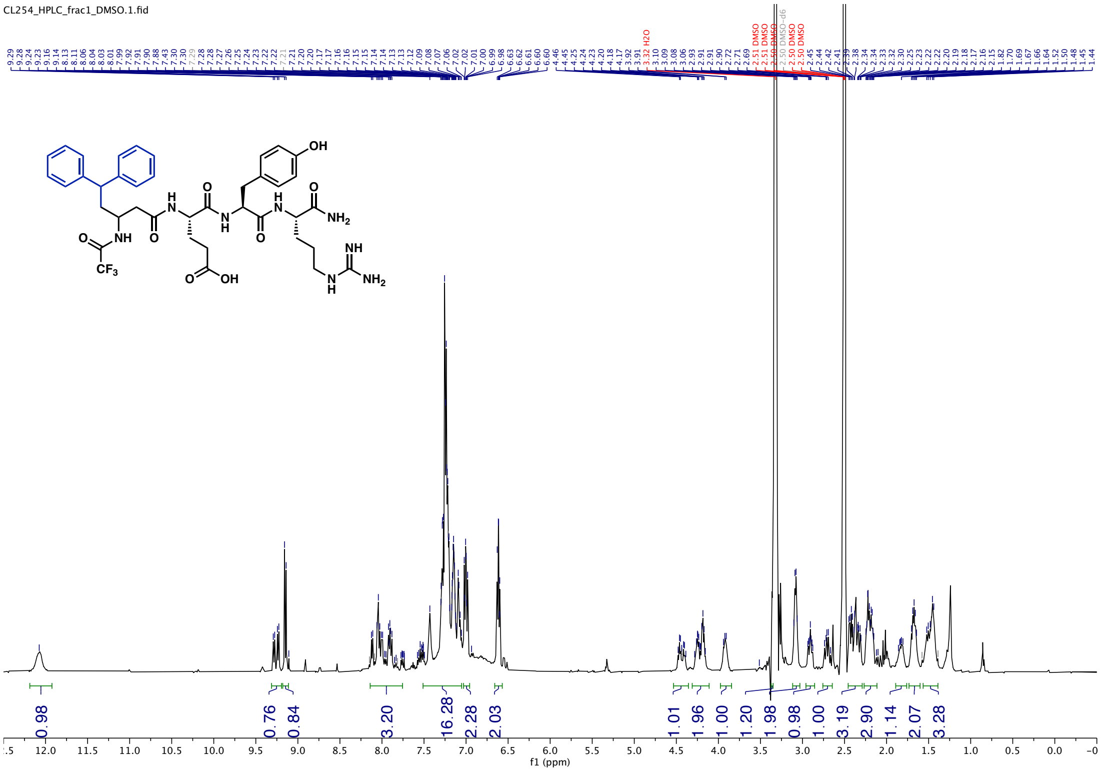
CL254_HPLC_frac1_DMSO.1.fid
f1 (ppm)

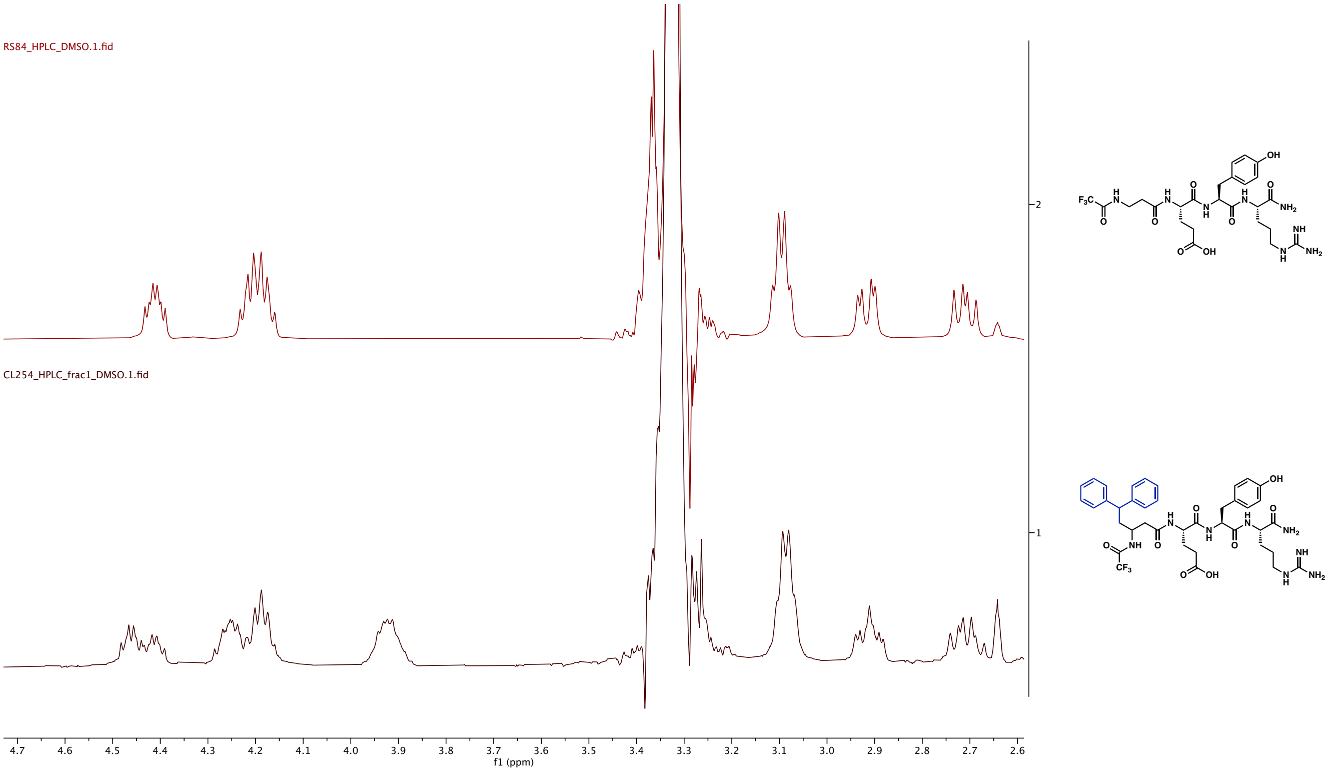
RS84_HPLC_DMSO.1.fid
CL254_HPLC_frac1_DMSO.1.fid
f1 (ppm)

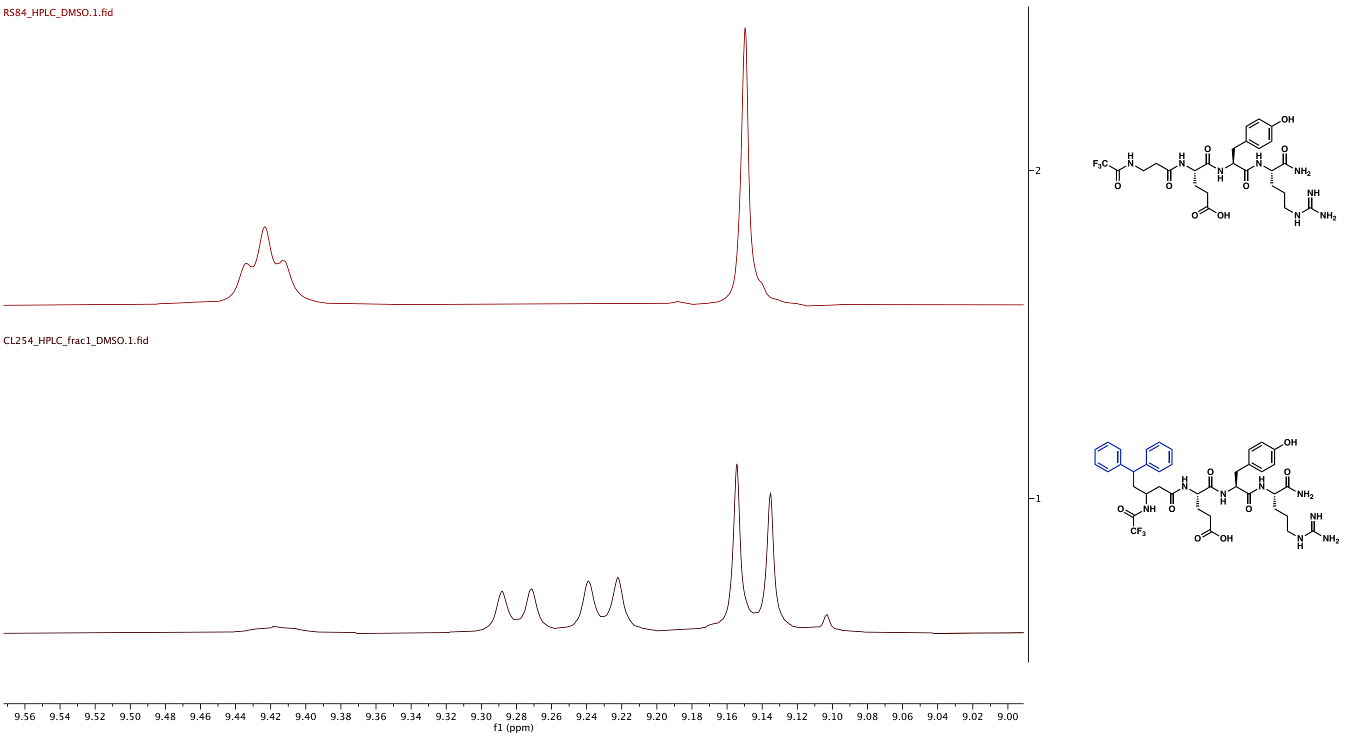
RS84_HPLC_DMSO.1.fid
CL254_HPLC_frac1_DMSO.1.fid
f1 (ppm)

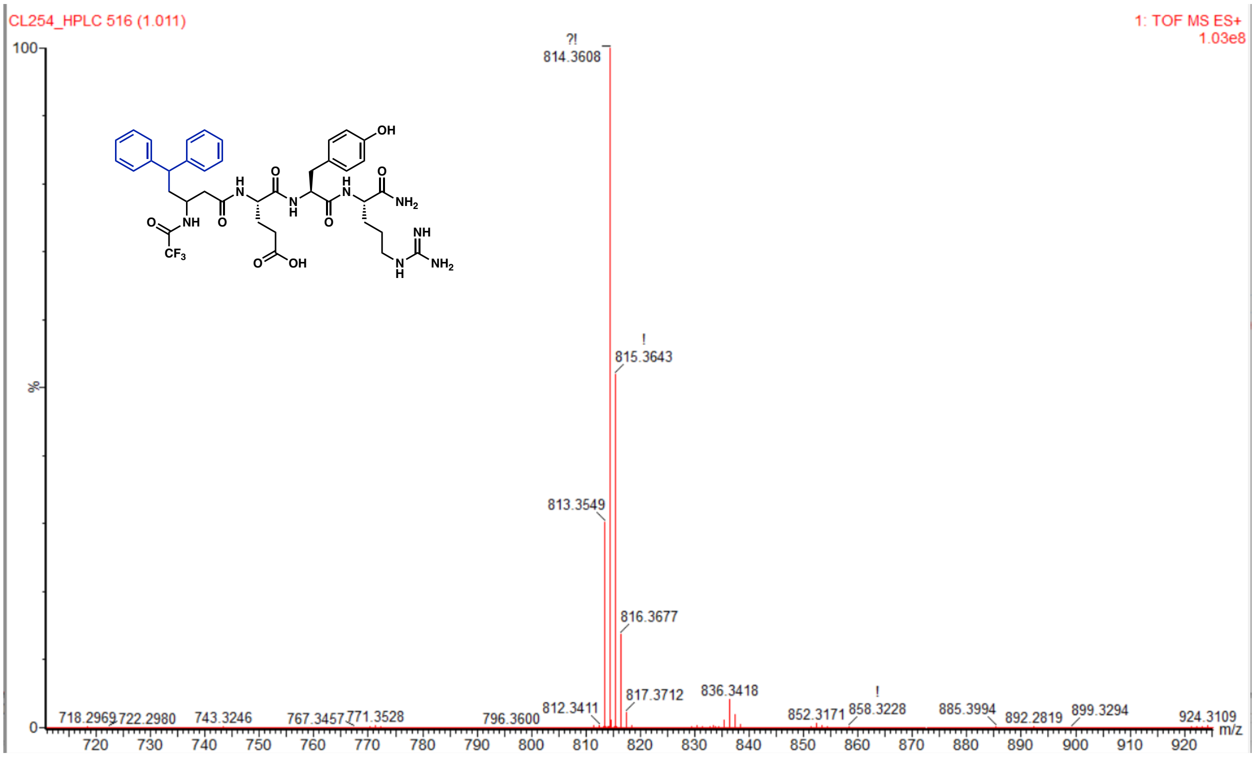
CL254_HPLC 516 (1.011)
1: TOF MS ES+
1.03e8
814.3608
815.3643
813.3549
816.3677
817.3712
836.3418
858.3228
m/z

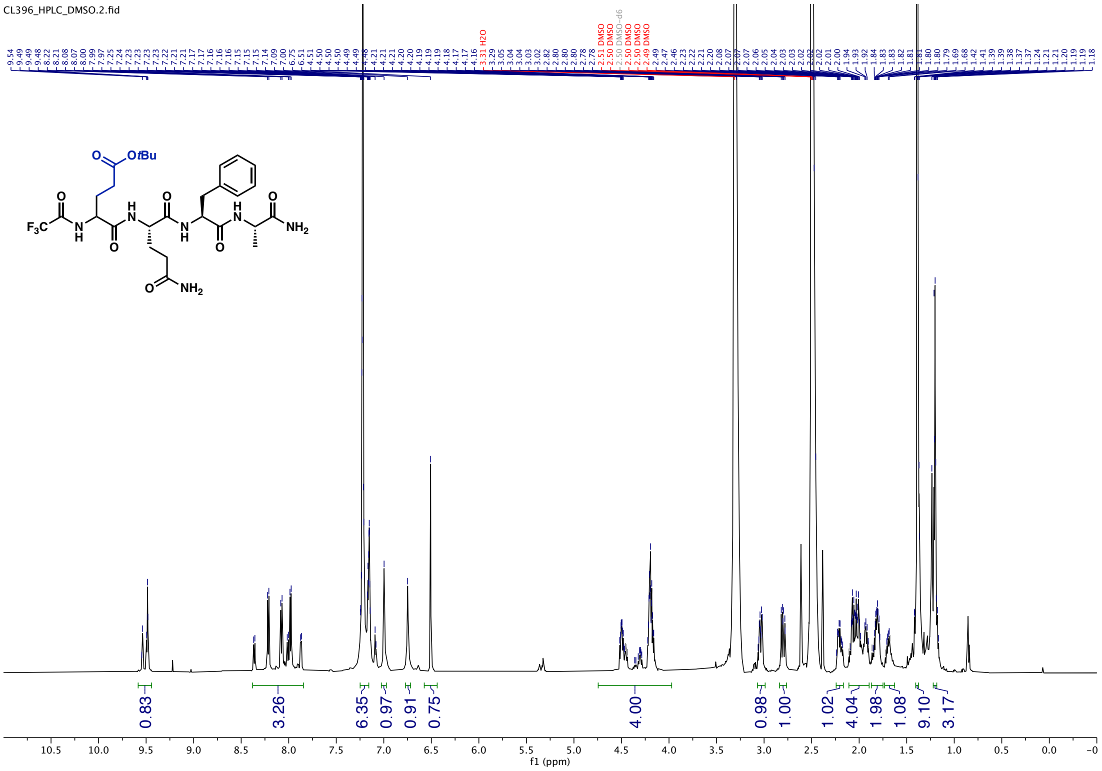
CL396_HPLC_DMSO.2.fid
OtBu
F3C
NH2
f1 (ppm)

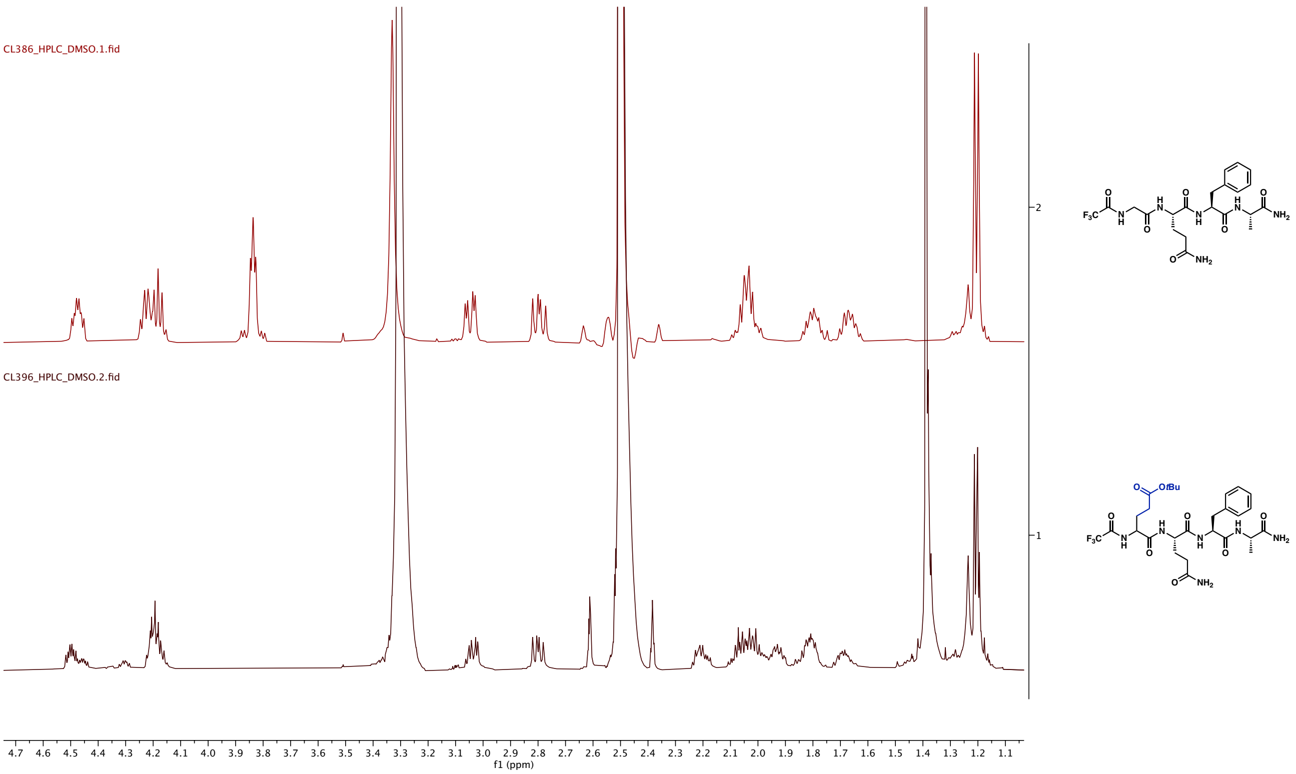
CL386_HPLC_DMSO.1.fid
CL396_HPLC_DMSO.2.fid
F3C
NH2
OtBu
f1 (ppm)

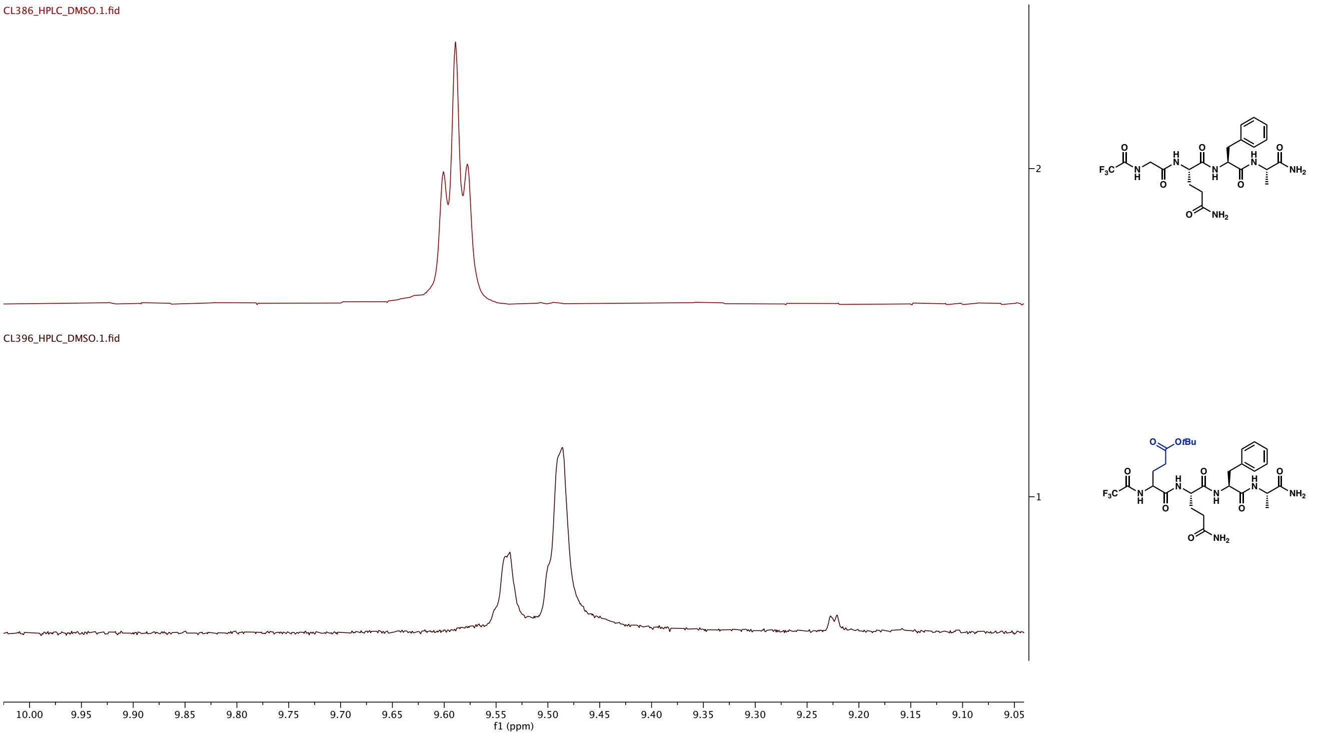
CL386_HPLC_DMSO.1.fid
CL396_HPLC_DMSO.1.fid
f1 (ppm)
O*t*Bu

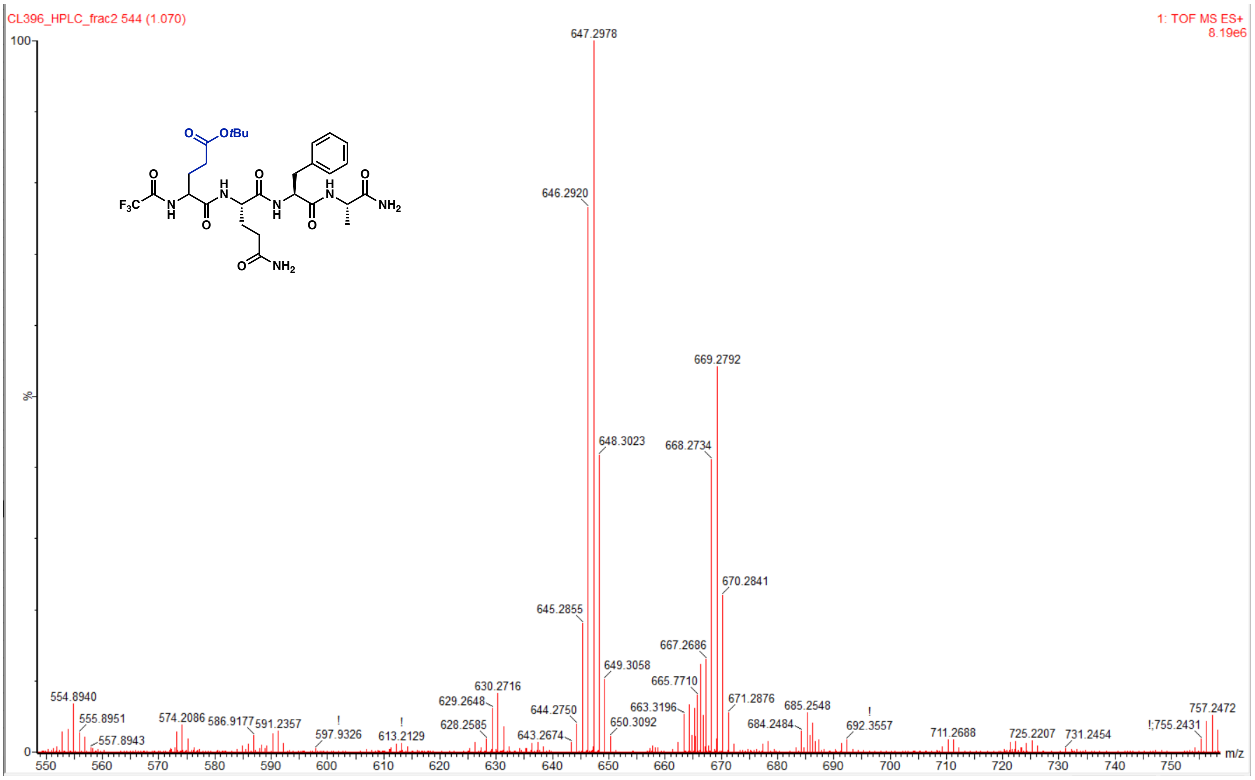
CL396_HPLC_frac2 544 (1.070)
1: TOF MS ES+
8.19e6
647.2978
646.2920
669.2792
648.3023
668.2734
670.2841
645.2855
667.2686
649.3058
665.7710
630.2716
629.2648
554.8940
663.3196
671.2876
685.2548
644.2750
574.2086
586.9177
591.2357
555.8951
650.3092
684.2484
692.3557
757.2472
755.2431
628.2585
597.9326
613.2129
643.2674
711.2688
725.2207
731.2454
557.8943
m/z
%
O*t*Bu

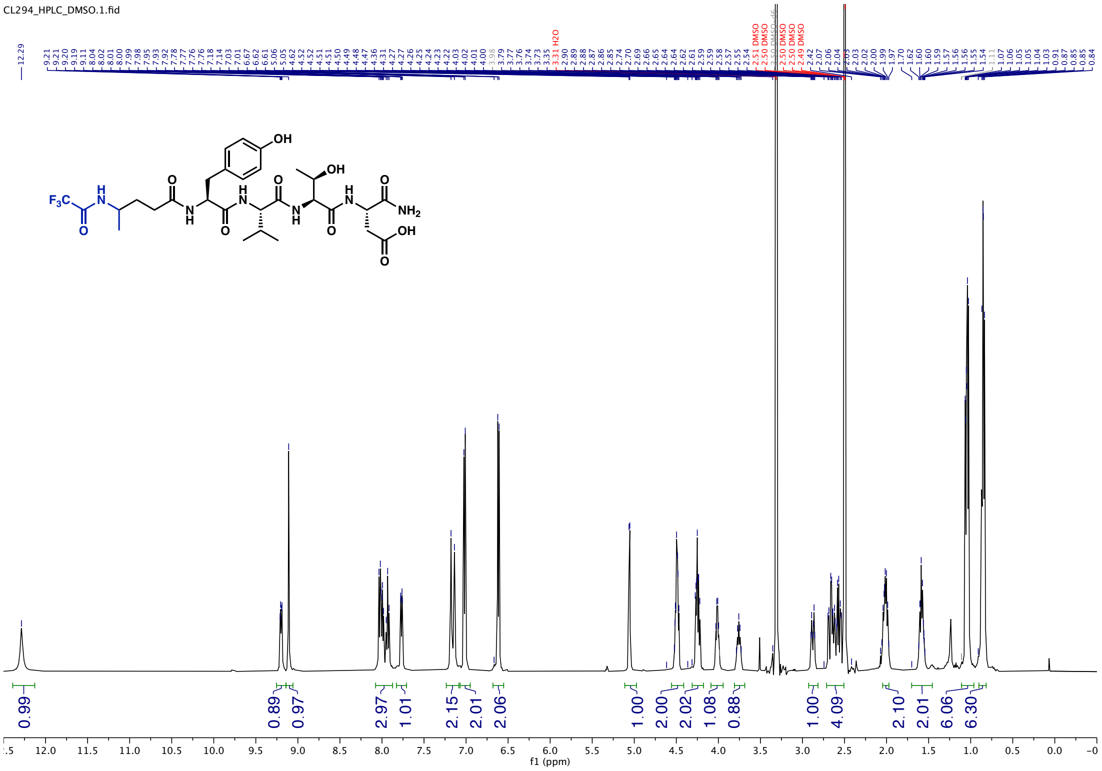
CL294_HPLC_DMSO.1.fid
f1 (ppm)

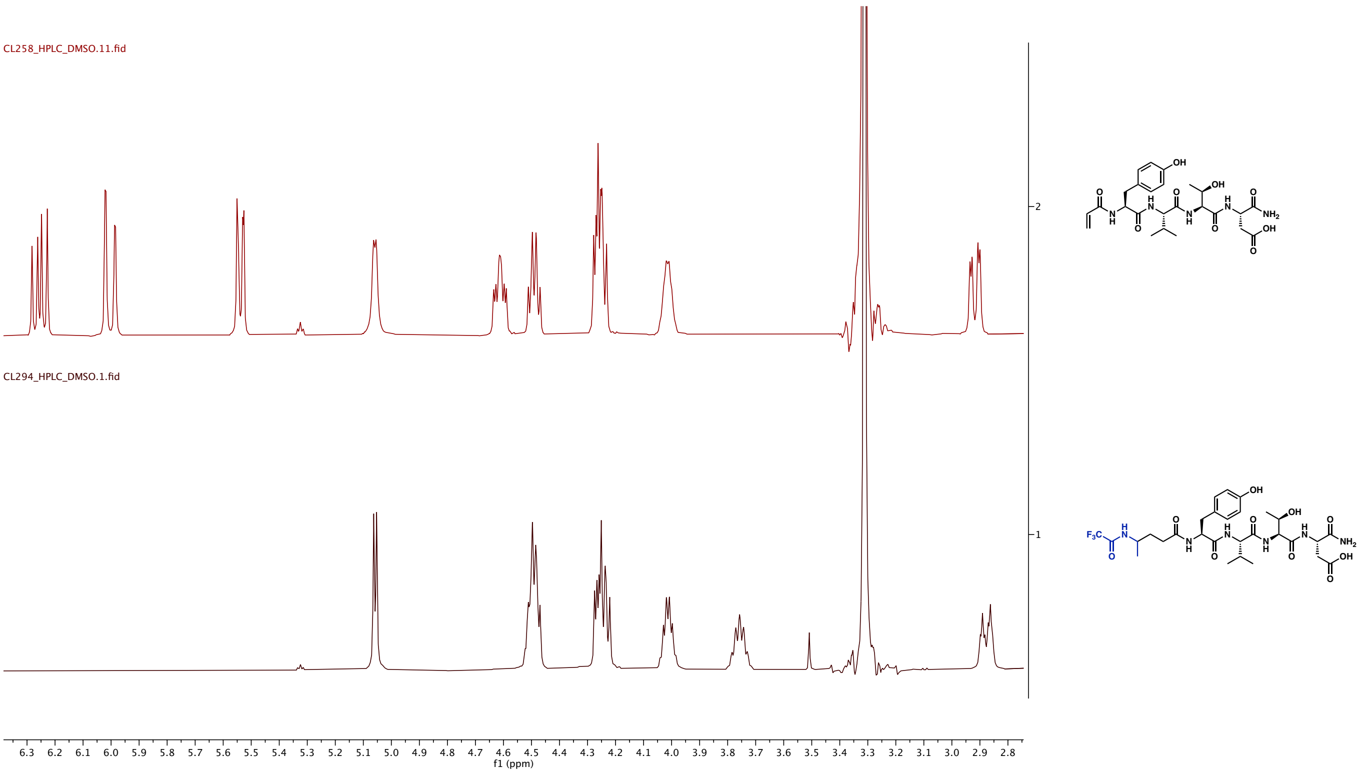
CL258_HPLC_DMSO.11.fid
CL294_HPLC_DMSO.1.fid
f1 (ppm)

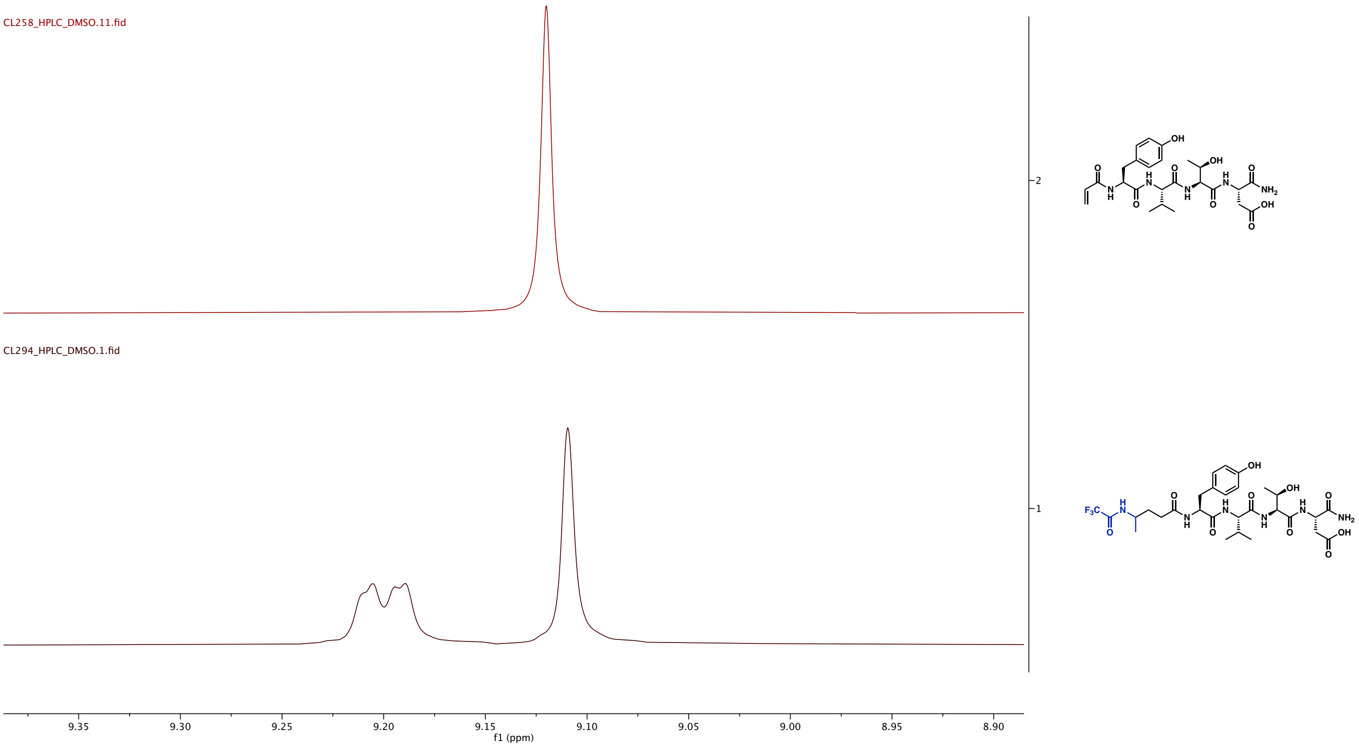

CL258_HPLC_DMSO.11.fid
CL294_HPLC_DMSO.1.fid
9.35
9.30
9.25
9.20
9.15
9.10
9.05
9.00
8.95
8.90
f1 (ppm)

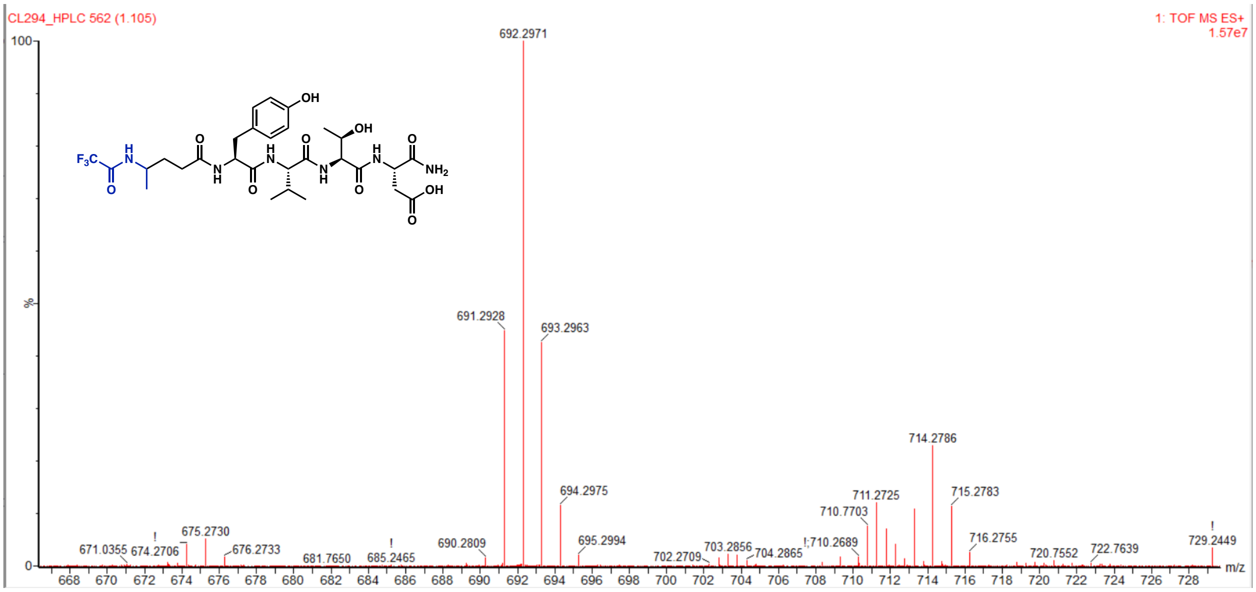

CL294_HPLC 562 (1.105)
1: TOF MS ES+
1.57e7
692.2971
691.2928
693.2963
714.2786
694.2975
711.2725
715.2783
710.7703
695.2994
716.2755
729.2449
675.2730
671.0355
674.2706
676.2733
690.2809
681.7650
685.2465
702.2709
703.2856
704.2865
710.2689
720.7552
722.7639
m/z

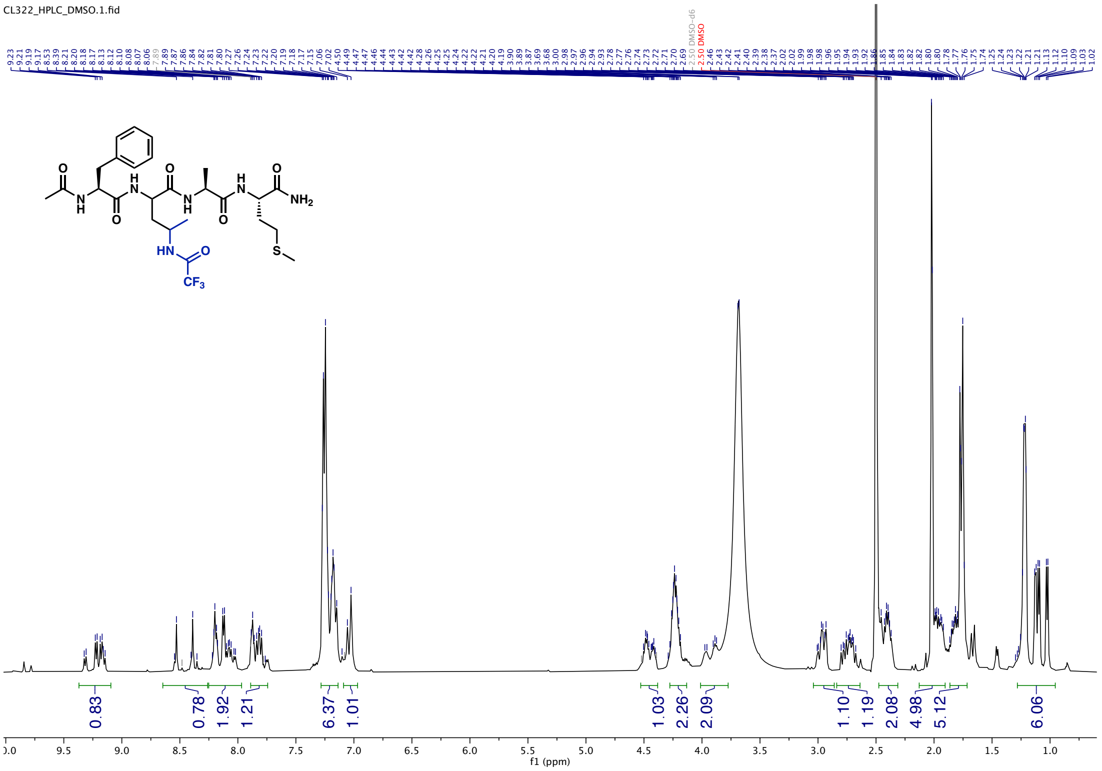
CL322_HPLC_DMSO.1.fid
2.50 DMSO
0.83
0.78
1.92
1.21
6.37
1.01
1.03
2.26
2.09
1.10
1.19
2.08
4.98
5.12
6.06
f1 (ppm)

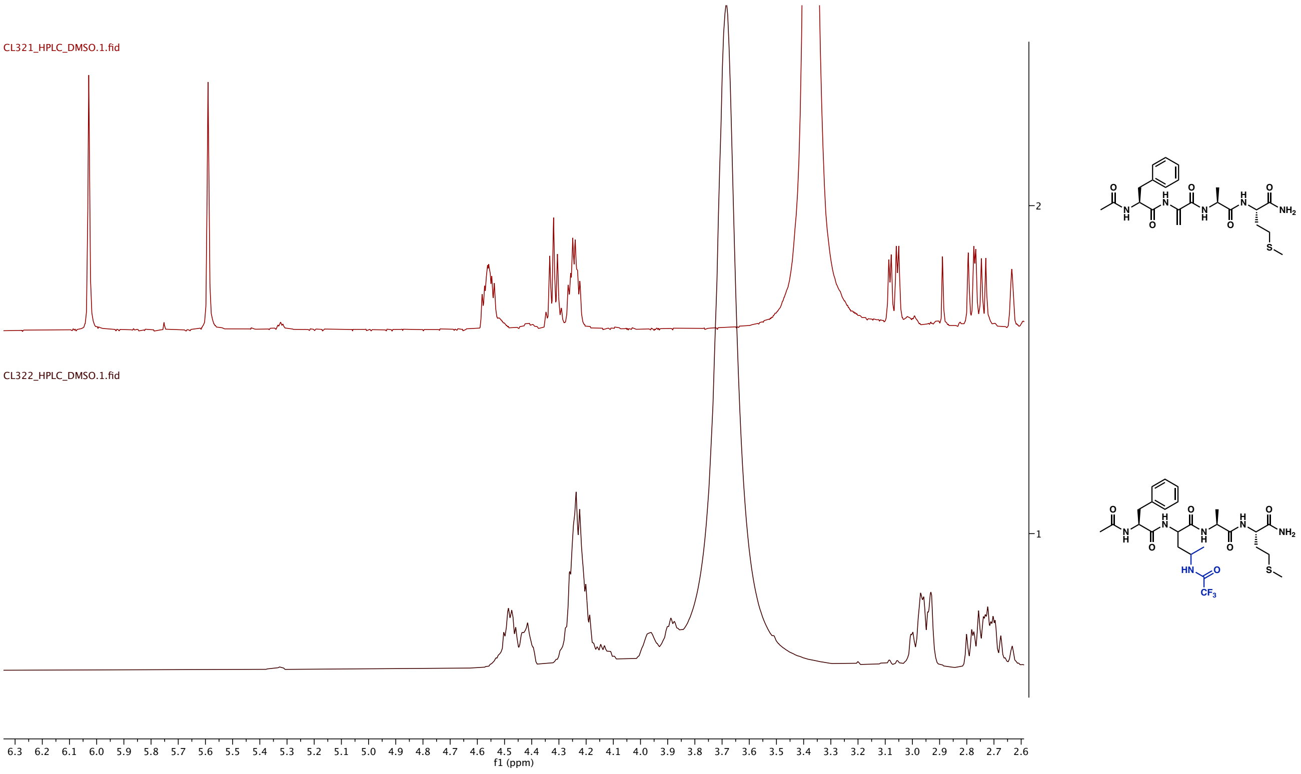
CL321_HPLC_DMSO.1.fid
CL322_HPLC_DMSO.1.fid
f1 (ppm)

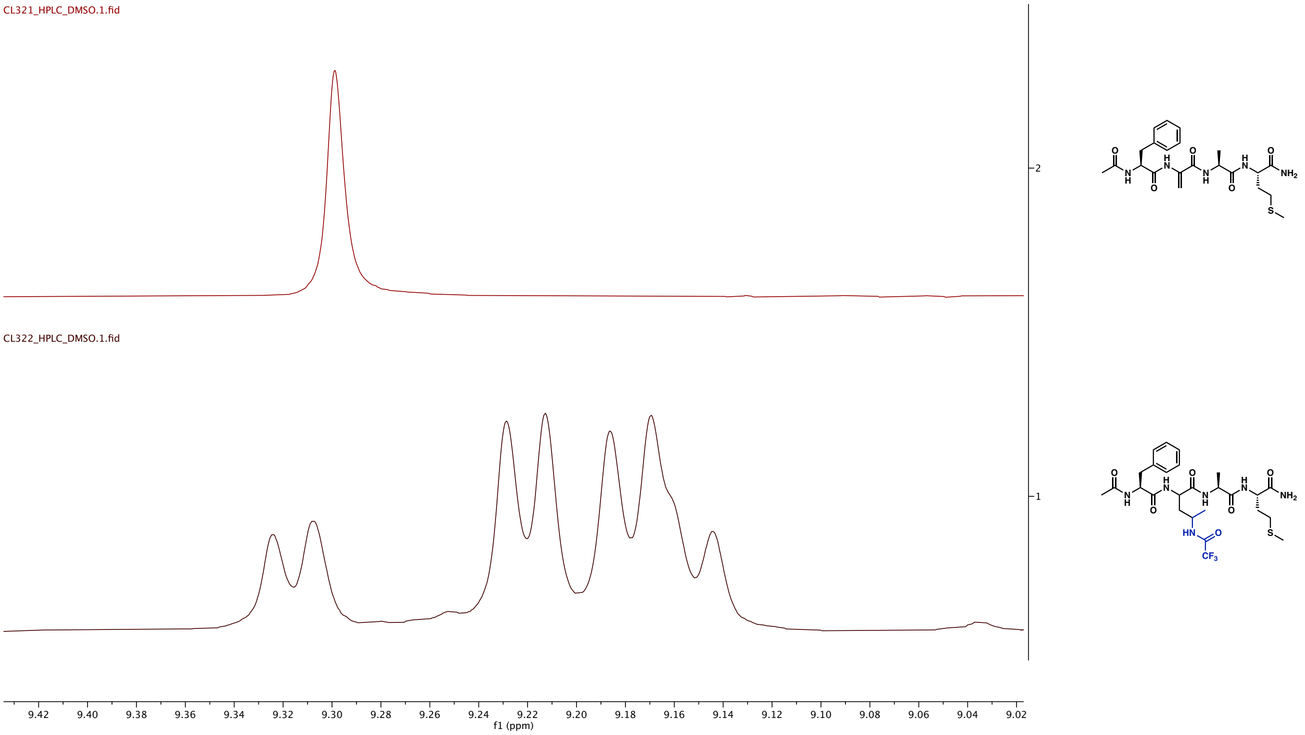

CL321_HPLC_DMSO.1.fid
CL322_HPLC_DMSO.1.fid
f1 (ppm)

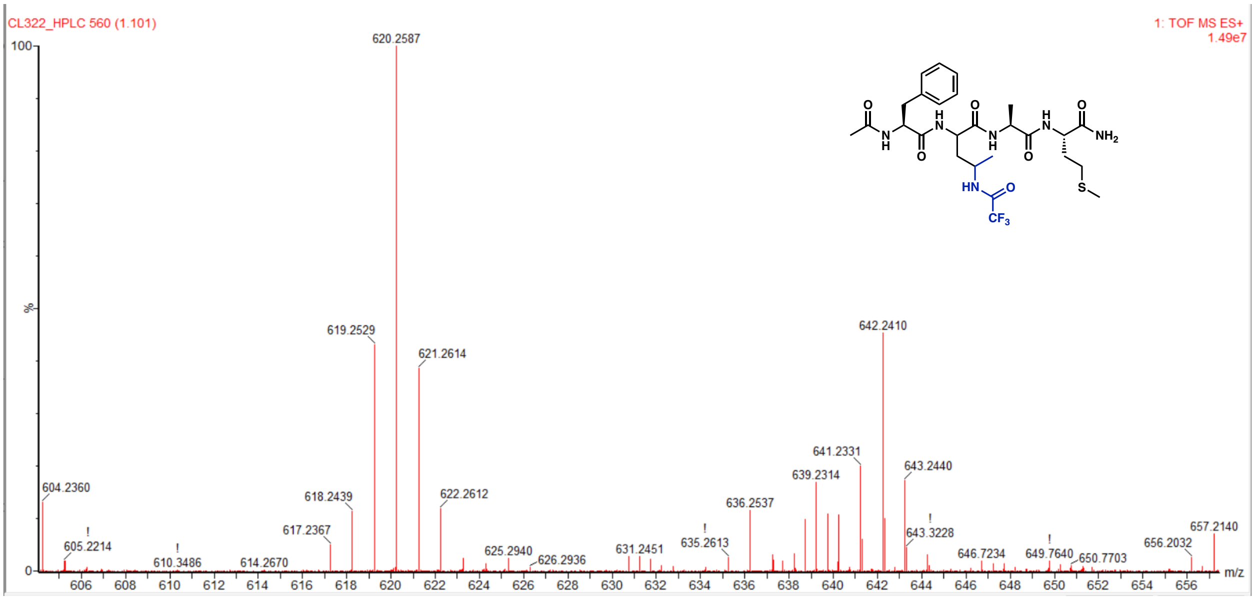

CL322_HPLC 560 (1.101)
1: TOF MS ES+
1.49e7
620.2587
642.2410
619.2529
621.2614
641.2331
643.2440
639.2314
604.2360
618.2439
622.2612
636.2537
617.2367
643.3228
657.2140
605.2214
635.2613
656.2032
625.2940
631.2451
646.7234
649.7640
650.7703
610.3486
614.2670
626.2936
m/z

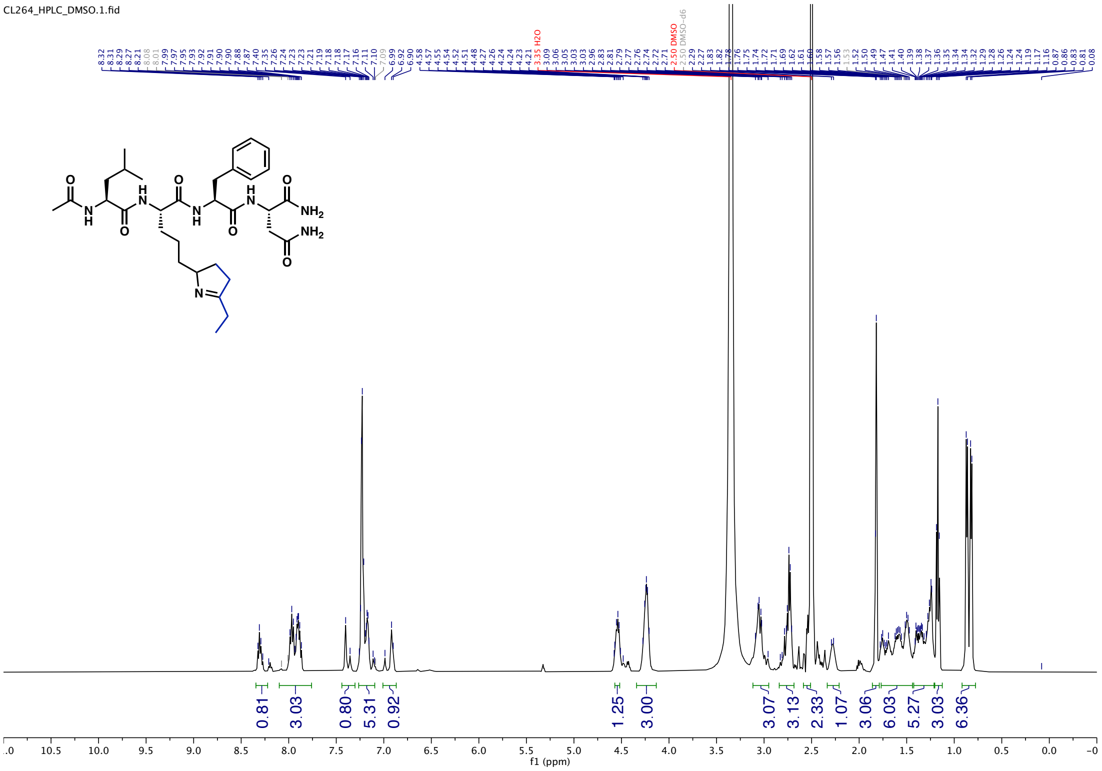
CL264_HPLC_DMSO.1.fid
0.81
3.03
0.80
5.31
0.92
1.25
3.00
3.07
3.13
2.33
1.07
3.06
6.03
5.27
3.03
6.36
f1 (ppm)

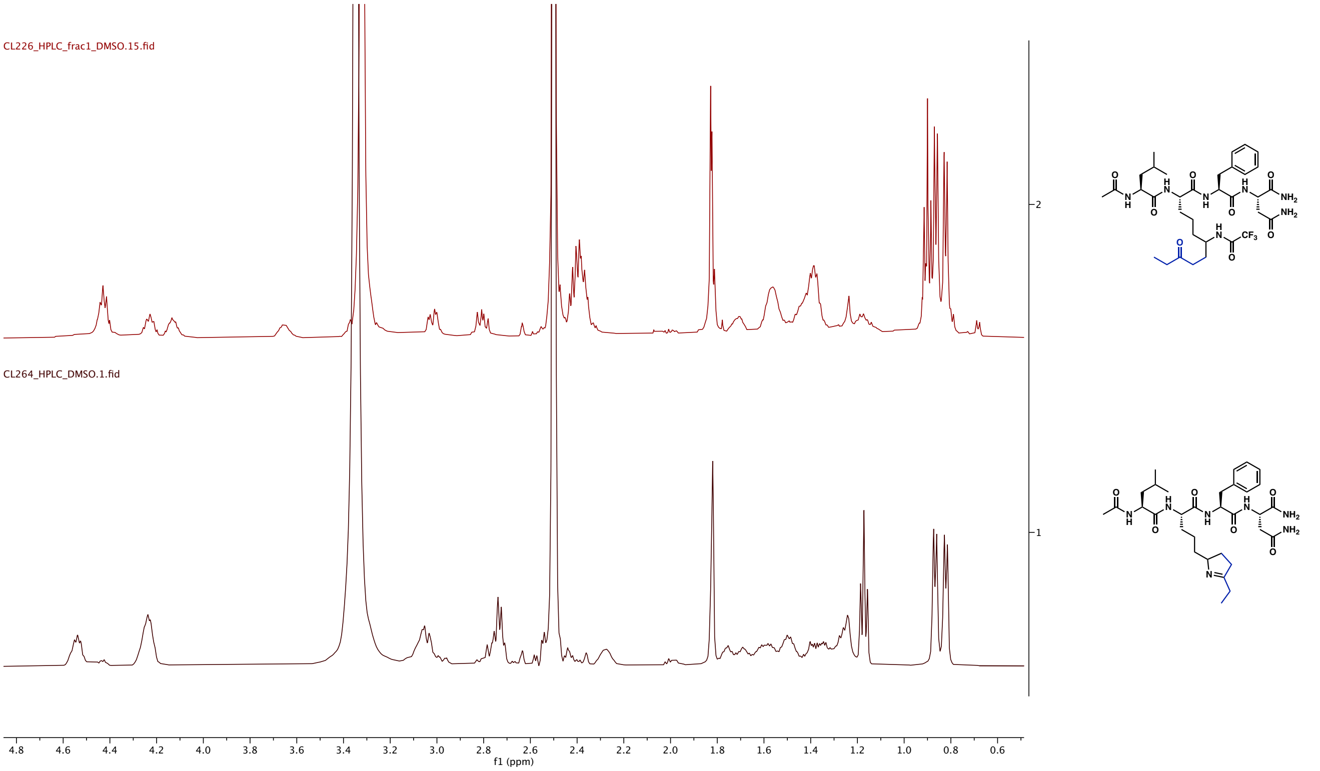
CL226_HPLC_frac1_DMSO.15.fid
CL264_HPLC_DMSO.1.fid
f1 (ppm)

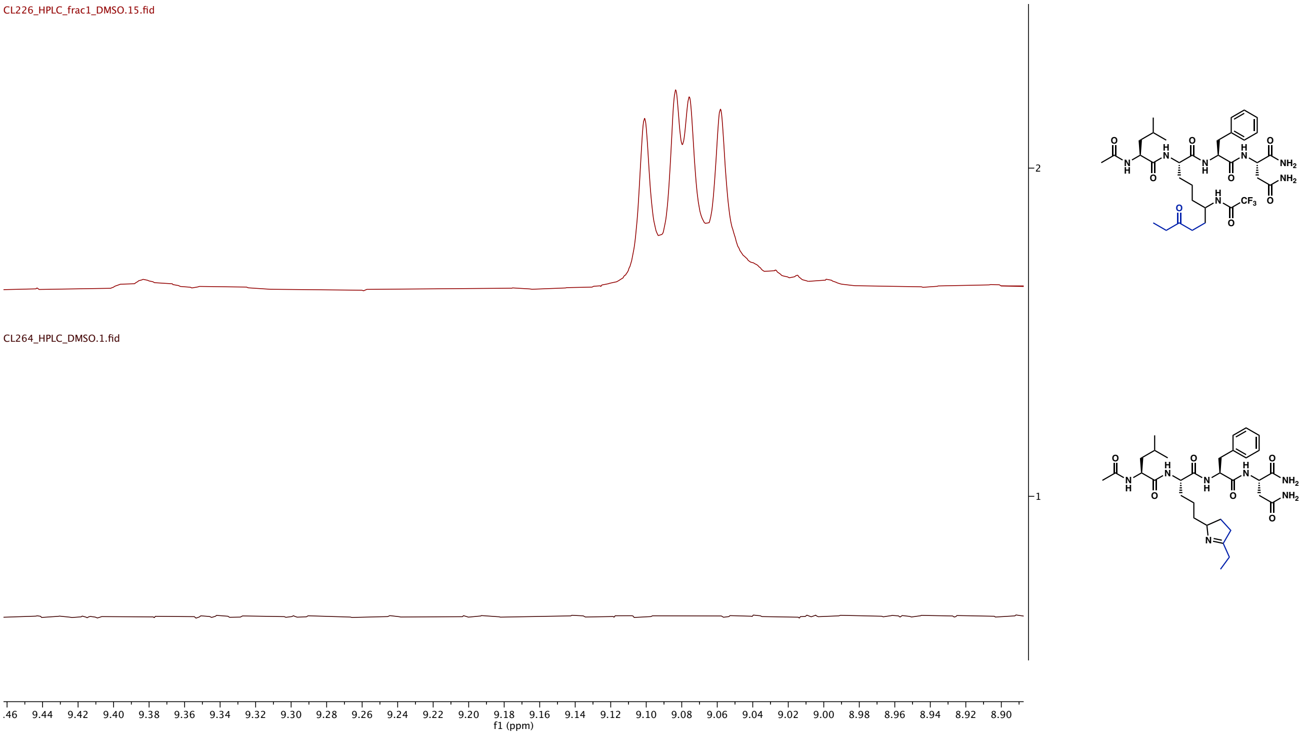
CL226_HPLC_frac1_DMSO.15.fid
CL264_HPLC_DMSO.1.fid
2
1
.46 9.44 9.42 9.40 9.38 9.36 9.34 9.32 9.30 9.28 9.26 9.24 9.22 9.20 9.18 9.16 9.14 9.12 9.10 9.08 9.06 9.04 9.02 9.00 8.98 8.96 8.94 8.92 8.90
f1 (ppm)

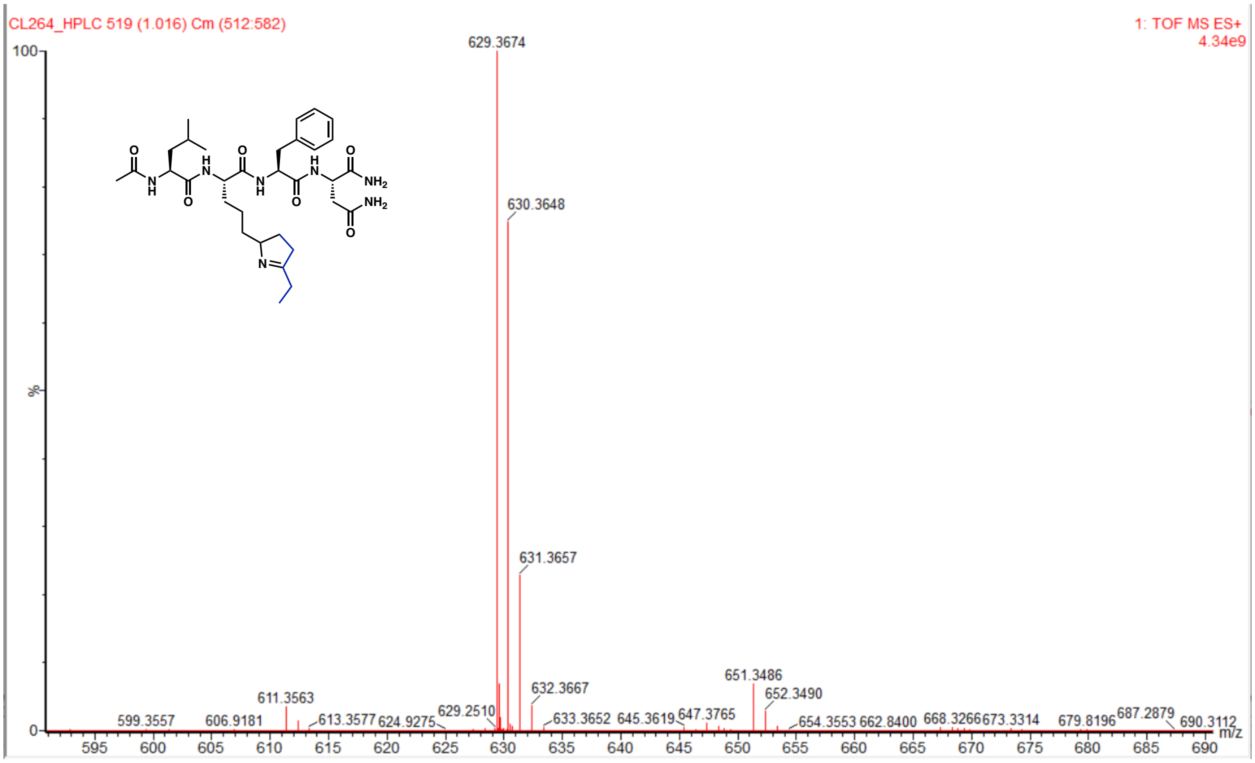
CL264_HPLC 519 (1.016) Cm (512:582)
1: TOF MS ES+
4.34e9
629.3674
630.3648
631.3657
632.3667
651.3486
652.3490
611.3563
599.3557
606.9181
613.3577
624.9275
629.2510
633.3652
645.3619
647.3765
654.3553
662.8400
668.3266
673.3314
679.8196
687.2879
690.3112
m/z
%

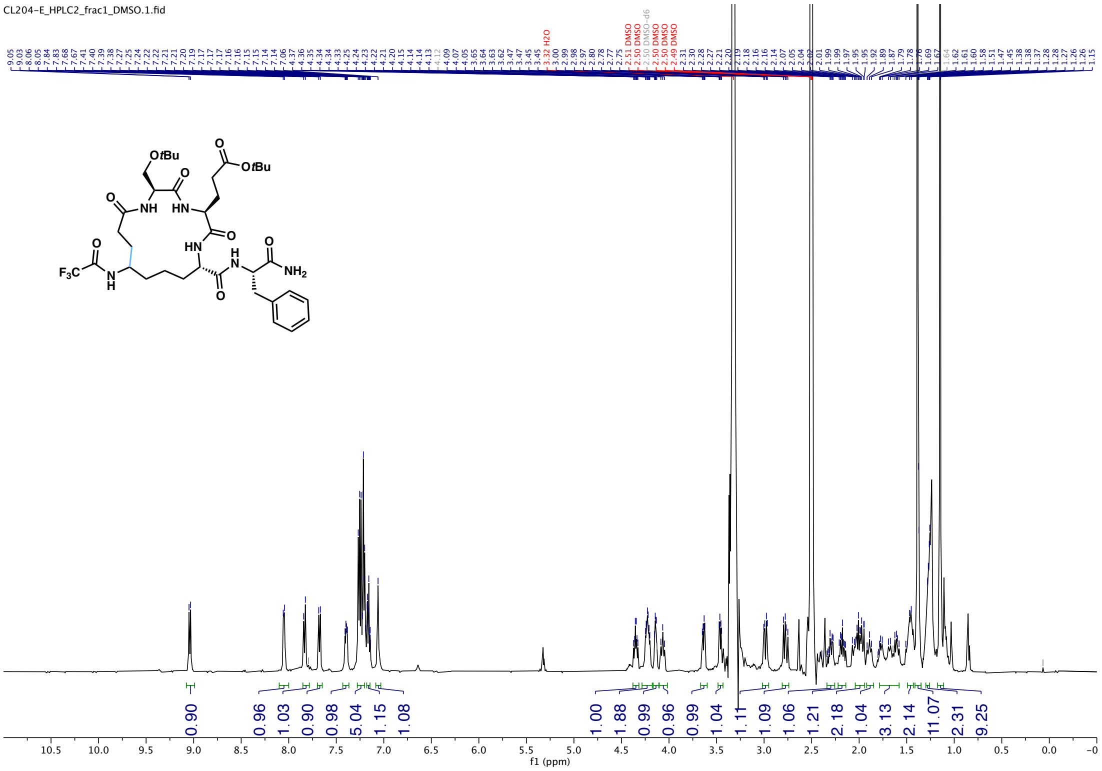
CL204-E_HPLC2_frac1_DMSO.1.fid
f1 (ppm)

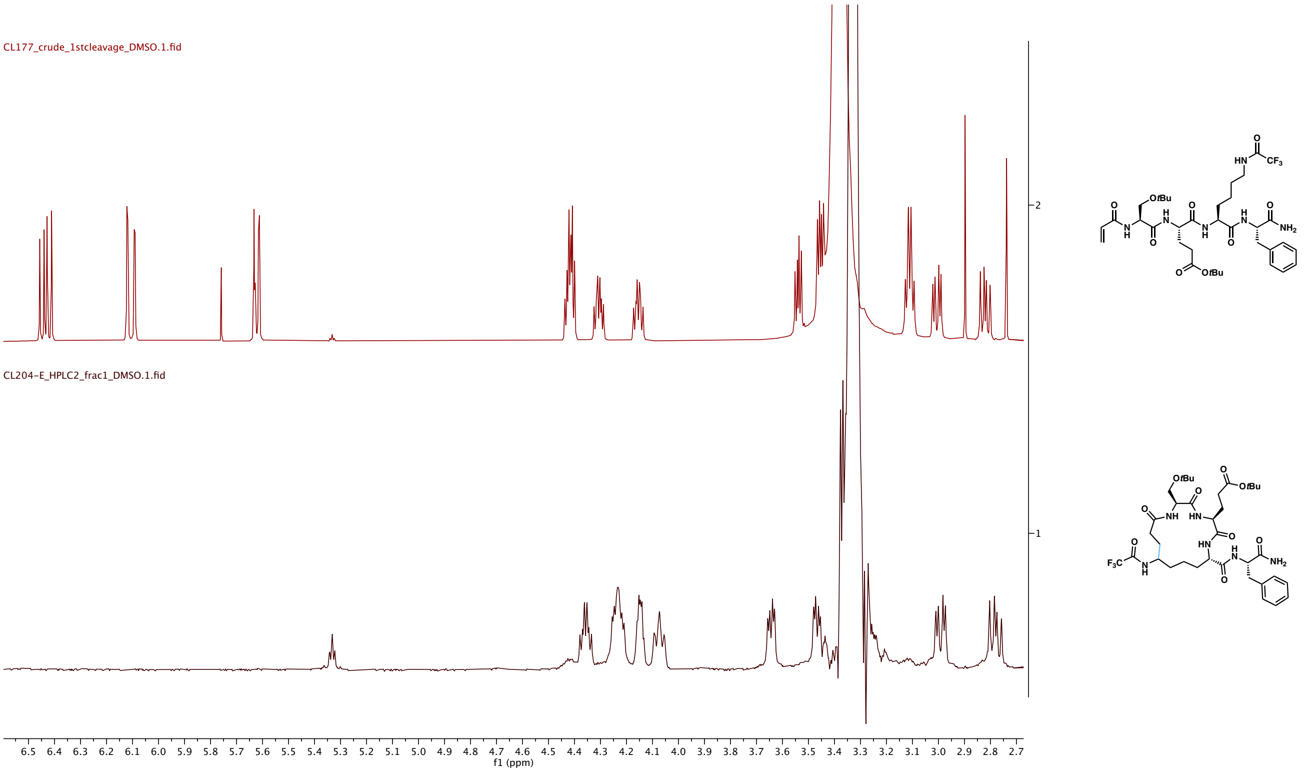
CL177_crude_1stcleavage_DMSO.1.fid
CL204-E_HPLC2_frac1_DMSO.1.fid
f1 (ppm)

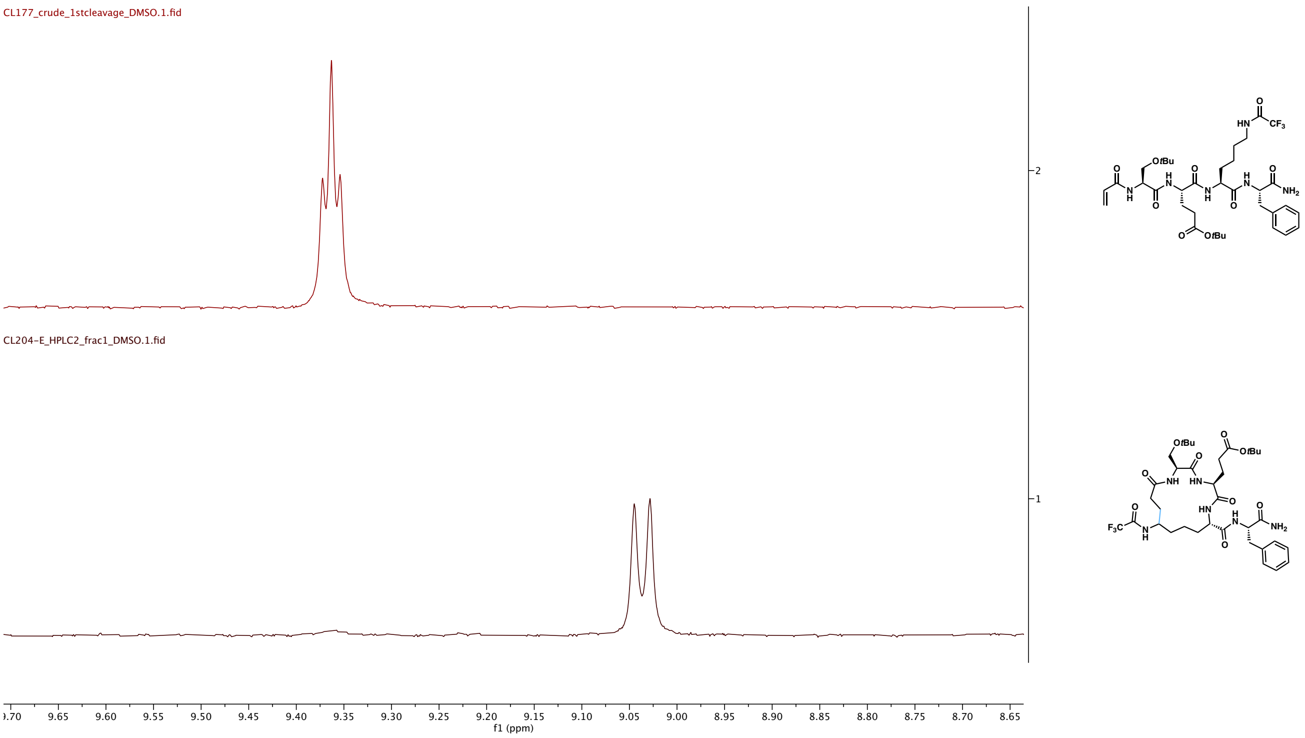

CL177_crude_1stcleavage_DMSO.1.fid
CL204-E_HPLC2_frac1_DMSO.1.fid
f1 (ppm)

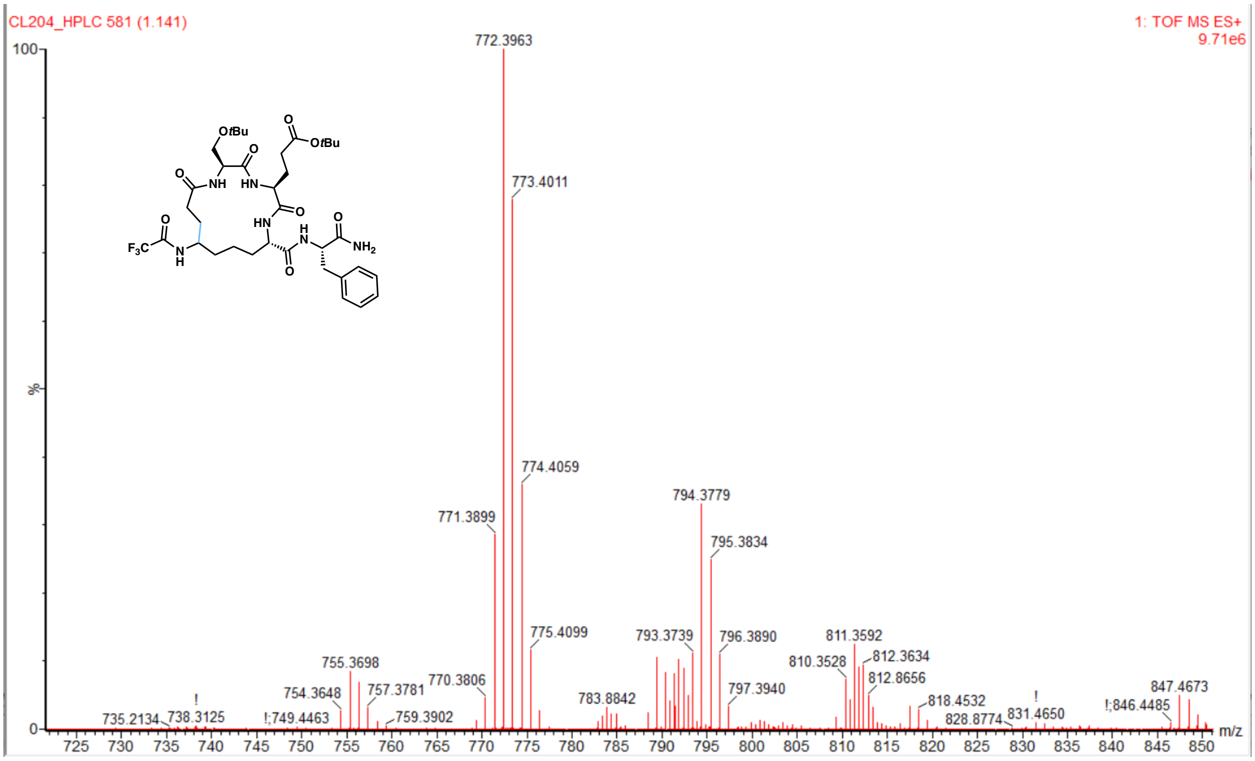

CL204_HPLC 581 (1.141)
1: TOF MS ES+
9.71e6
772.3963
773.4011
774.4059
771.3899
794.3779
795.3834
775.4099
793.3739
796.3890
811.3592
810.3528
812.3634
812.8656
755.3698
770.3806
757.3781
754.3648
797.3940
783.8842
818.4532
847.4673
846.4485
831.4650
828.8774
759.3902
749.4463
738.3125
735.2134
m/z

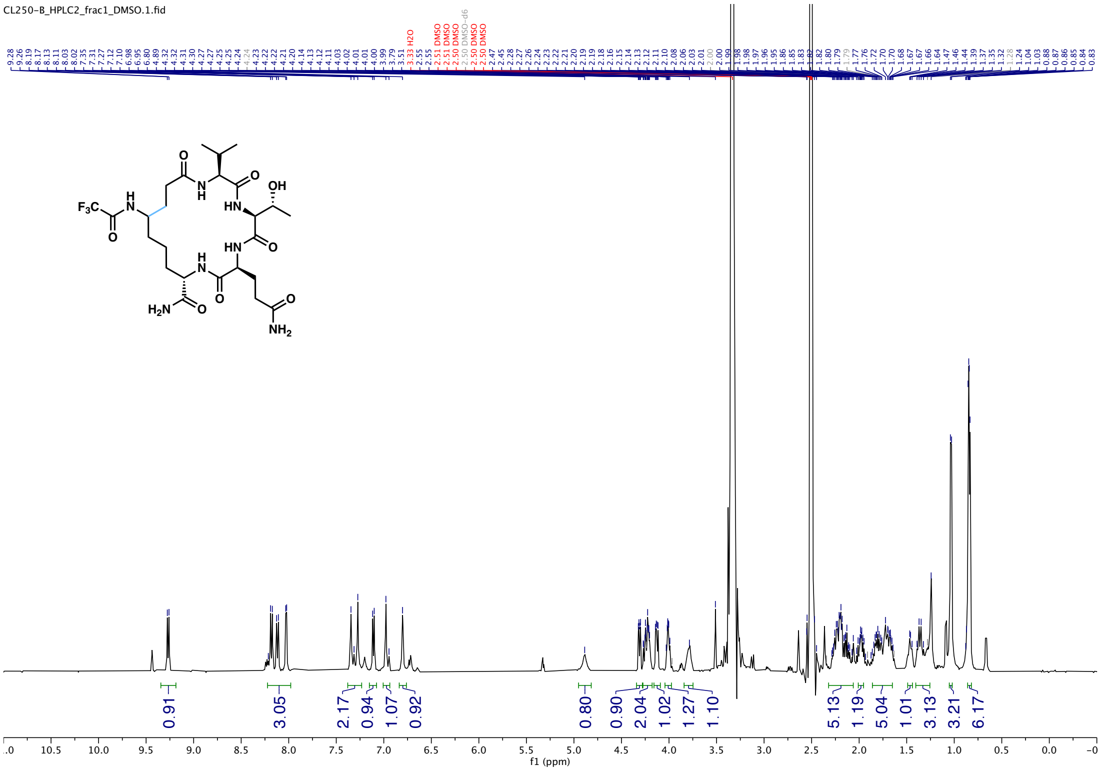
CL250-B_HPLC2_frac1_DMSO.1.fid
0.91
3.05
2.17
0.94
1.07
0.92
0.80
0.90
2.04
1.02
1.27
1.10
5.13
1.19
5.04
1.01
3.13
3.21
6.17
f1 (ppm)

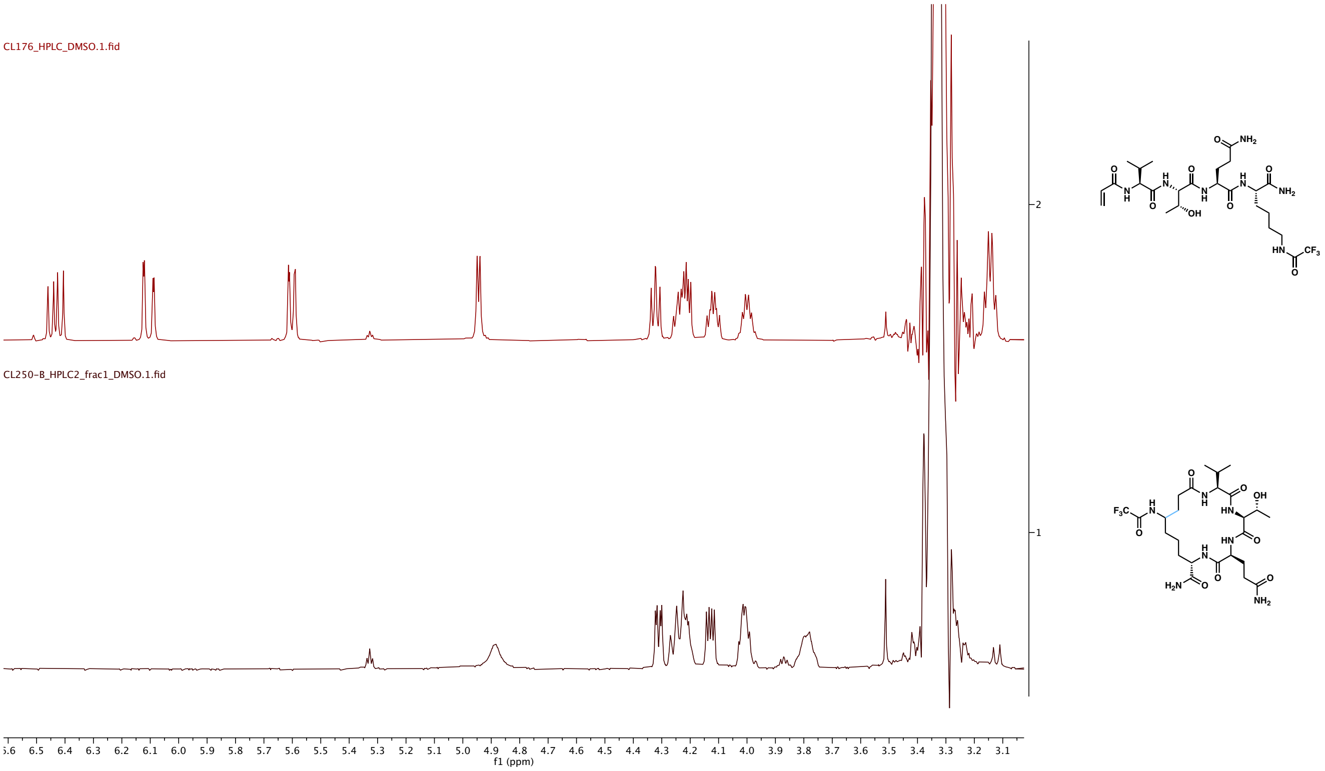
CL176_HPLC_DMSO.1.fid
CL250-B_HPLC2_frac1_DMSO.1.fid
f1 (ppm)

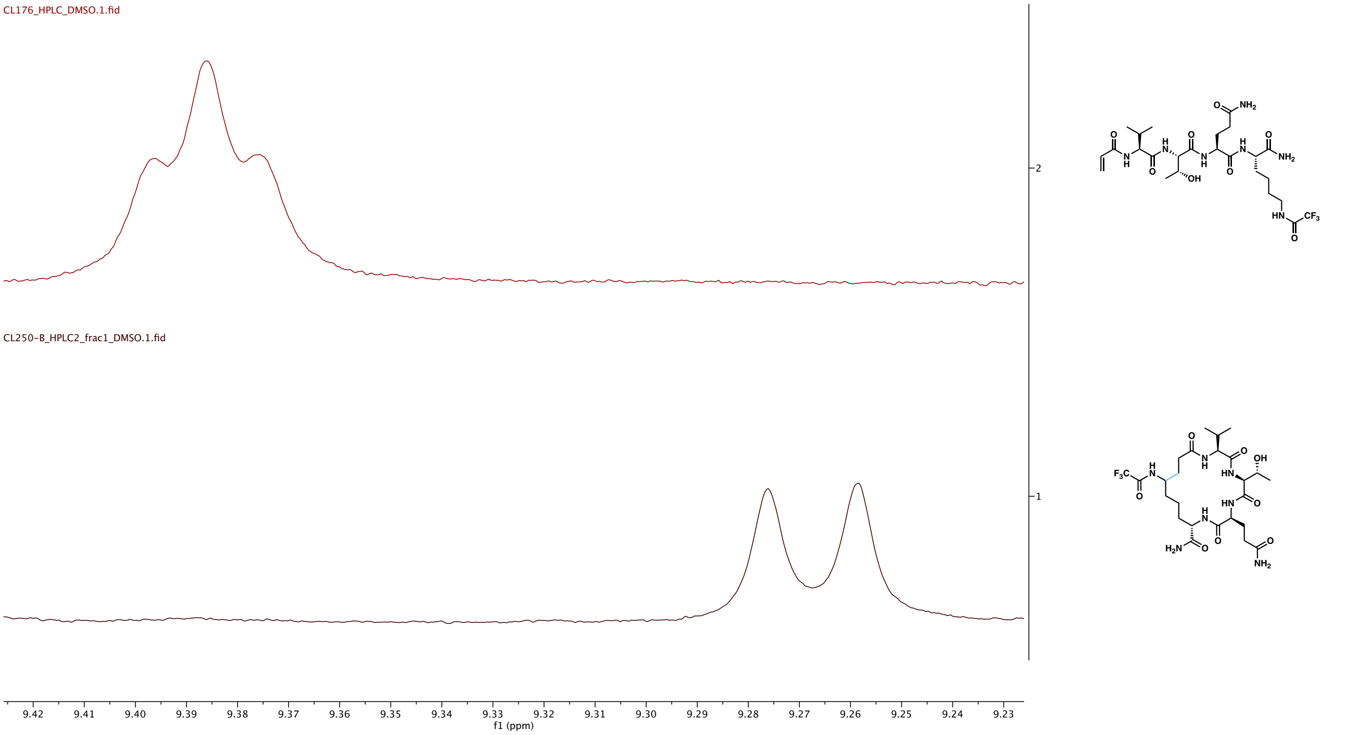
CL176_HPLC_DMSO.1.fid
CL250-B_HPLC2_frac1_DMSO.1.fid
9.42 9.41 9.40 9.39 9.38 9.37 9.36 9.35 9.34 9.33 9.32 9.31 9.30 9.29 9.28 9.27 9.26 9.25 9.24 9.23
f1 (ppm)
2
1

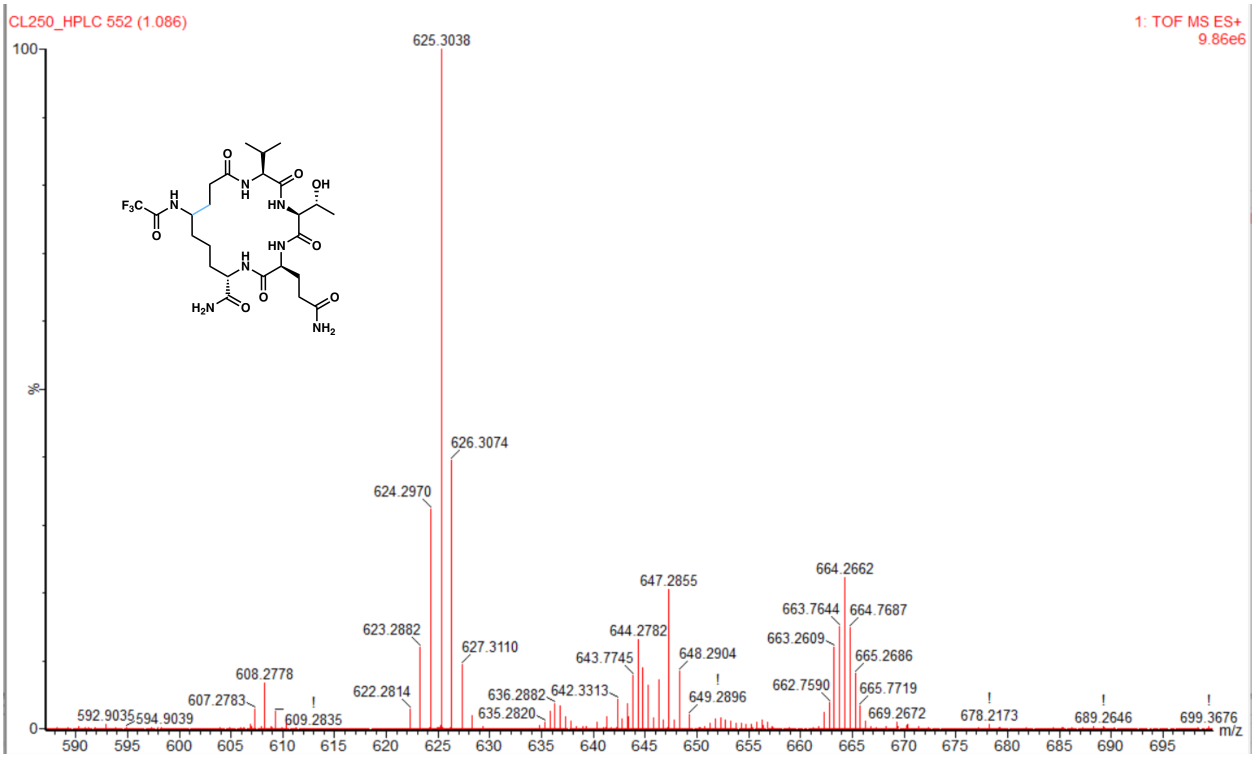
CL250_HPLC 552 (1.086)
1: TOF MS ES+
9.86e6
625.3038
626.3074
624.2970
647.2855
664.2662
663.7644
664.7687
663.2609
665.2686
644.2782
643.7745
648.2904
623.2882
627.3110
608.2778
607.2783
622.2814
636.2882
642.3313
635.2820
649.2896
662.7590
665.7719
669.2672
678.2173
689.2646
699.3676
592.9035
594.9039
609.2835
%
100
0
590 595 600 605 610 615 620 625 630 635 640 645 650 655 660 665 670 675 680 685 690 695
m/z

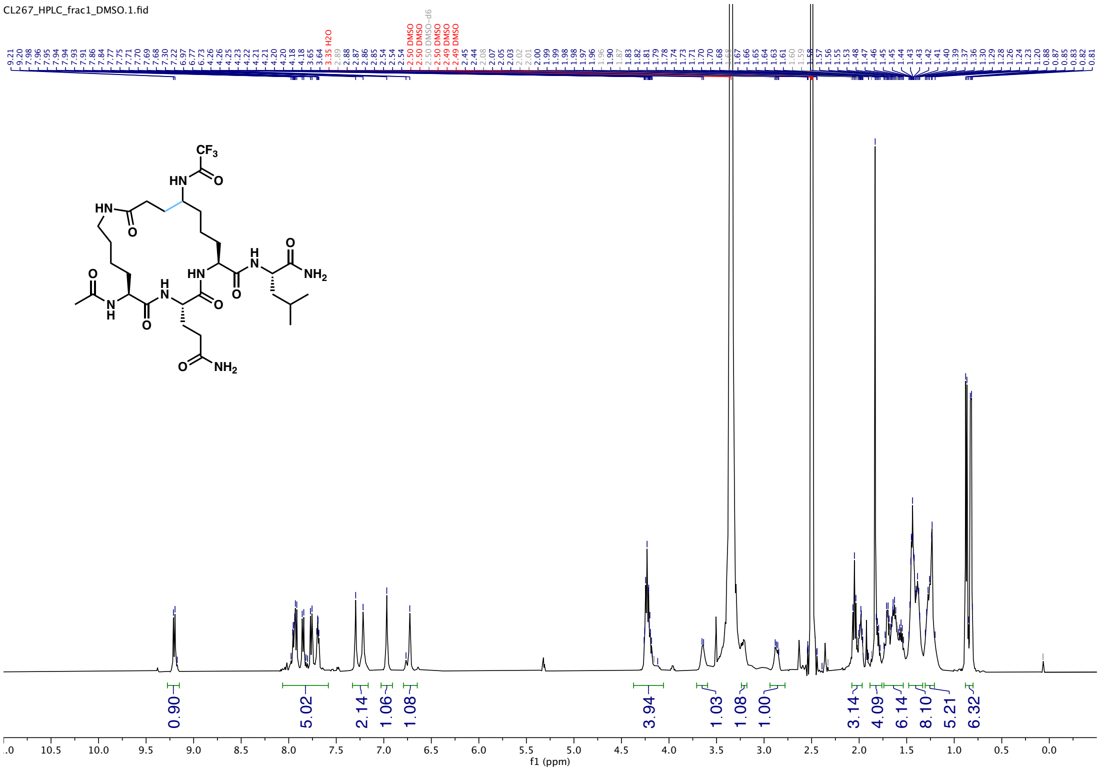
CL267_HPLC_frac1_DMSO.1.fid
0.90
5.02
2.14
1.06
1.08
3.94
1.03
1.08
1.00
3.14
4.09
6.14
8.10
5.21
6.32
f1 (ppm)

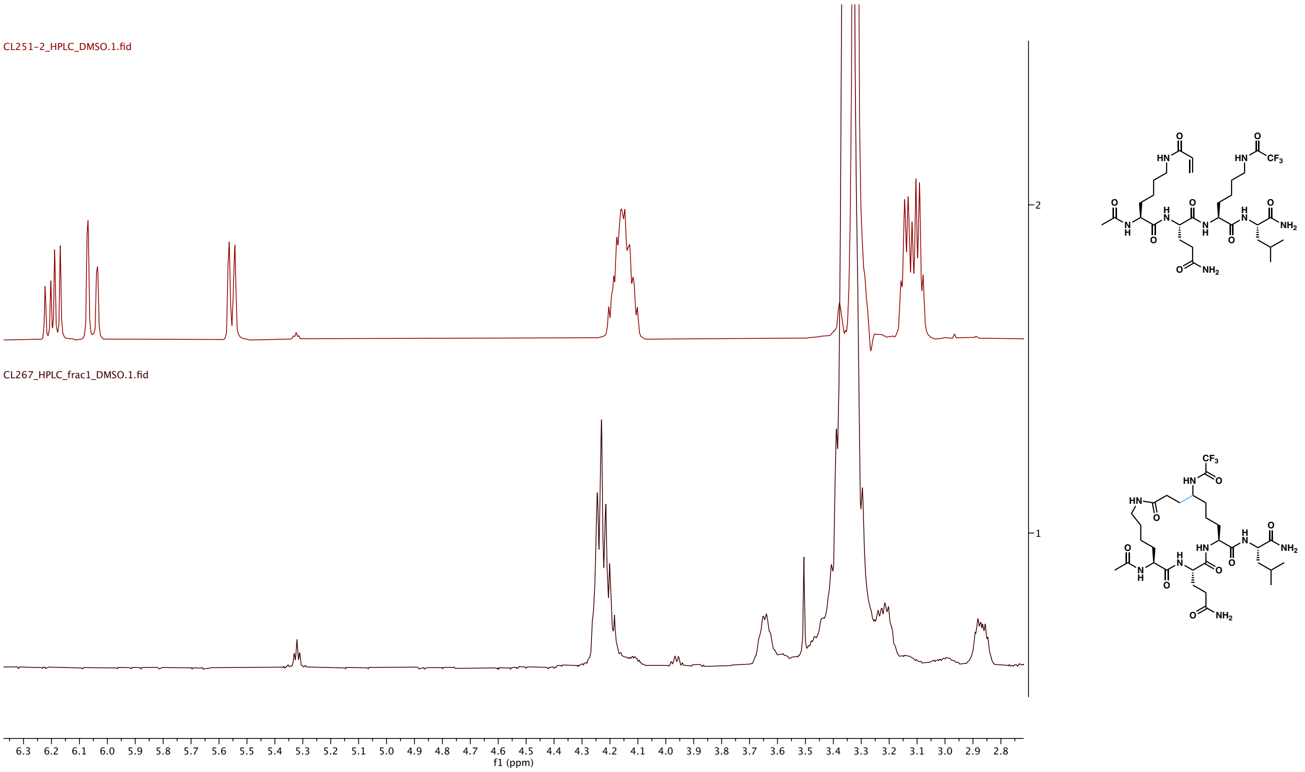
CL251-2_HPLC_DMSO.1.fid
CL267_HPLC_frac1_DMSO.1.fid
f1 (ppm)

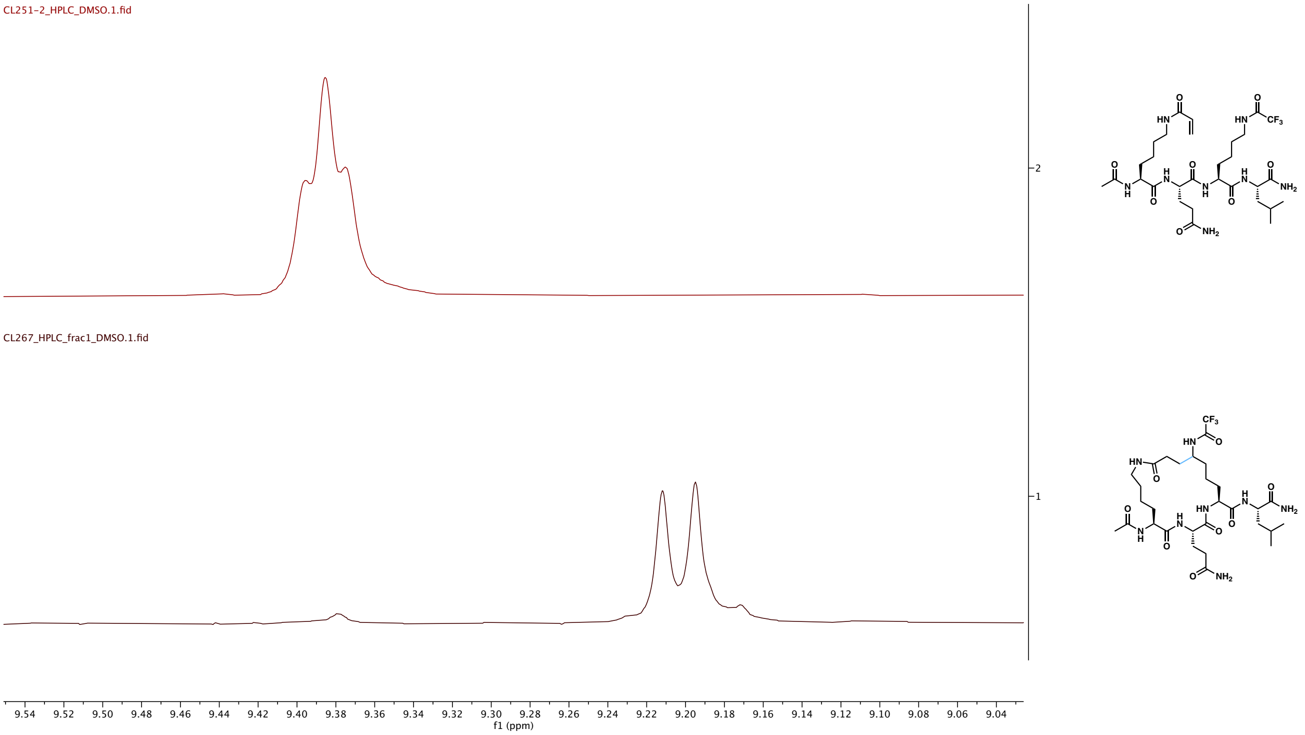
CL251-2_HPLC_DMSO.1.fid
CL267_HPLC_frac1_DMSO.1.fid
9.54
9.52
9.50
9.48
9.46
9.44
9.42
9.40
9.38
9.36
9.34
9.32
9.30
9.28
9.26
9.24
9.22
9.20
9.18
9.16
9.14
9.12
9.10
9.08
9.06
9.04
f1 (ppm)

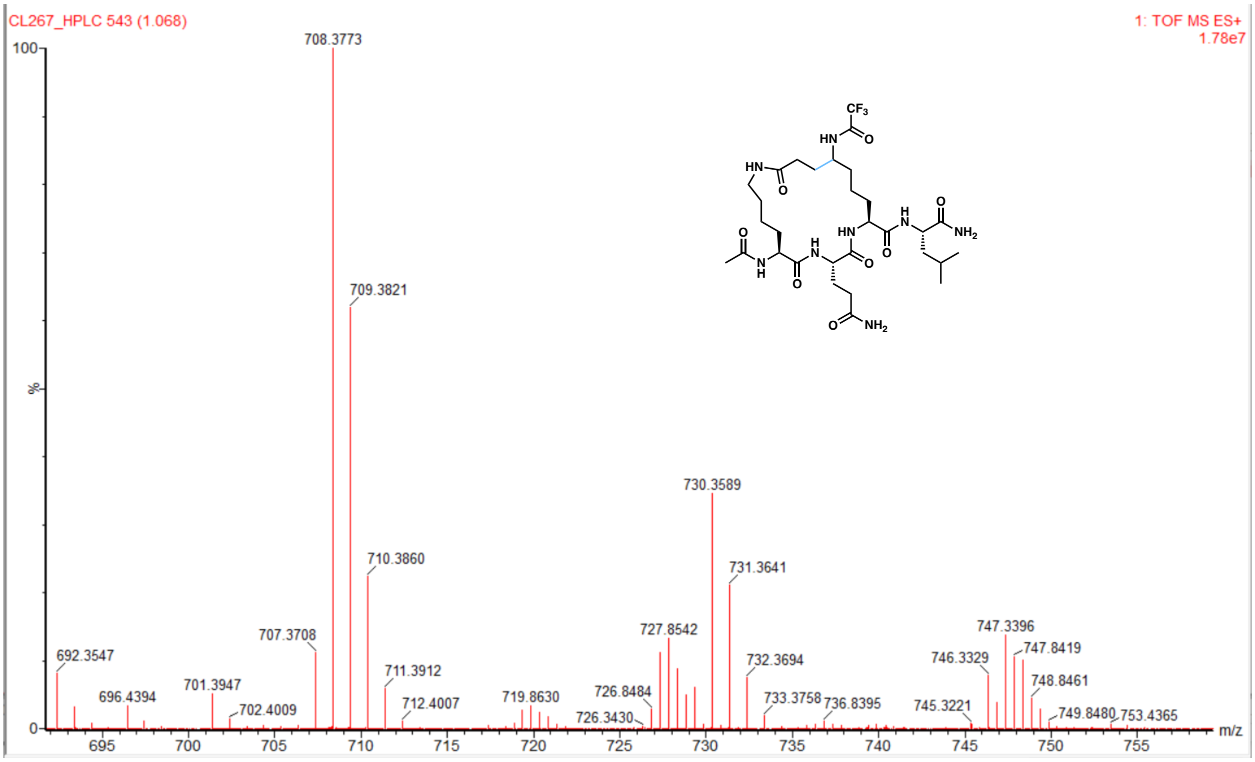
CL267_HPLC 543 (1.068)
1: TOF MS ES+
1.78e7
100
%
0
708.3773
709.3821
710.3860
730.3589
731.3641
707.3708
727.8542
747.3396
747.8419
746.3329
692.3547
732.3694
711.3912
748.8461
701.3947
696.4394
719.8630
726.8484
712.4007
733.3758
736.8395
745.3221
702.4009
726.3430
749.8480
753.4365
695
700
705
710
715
720
725
730
735
740
745
750
755
m/z

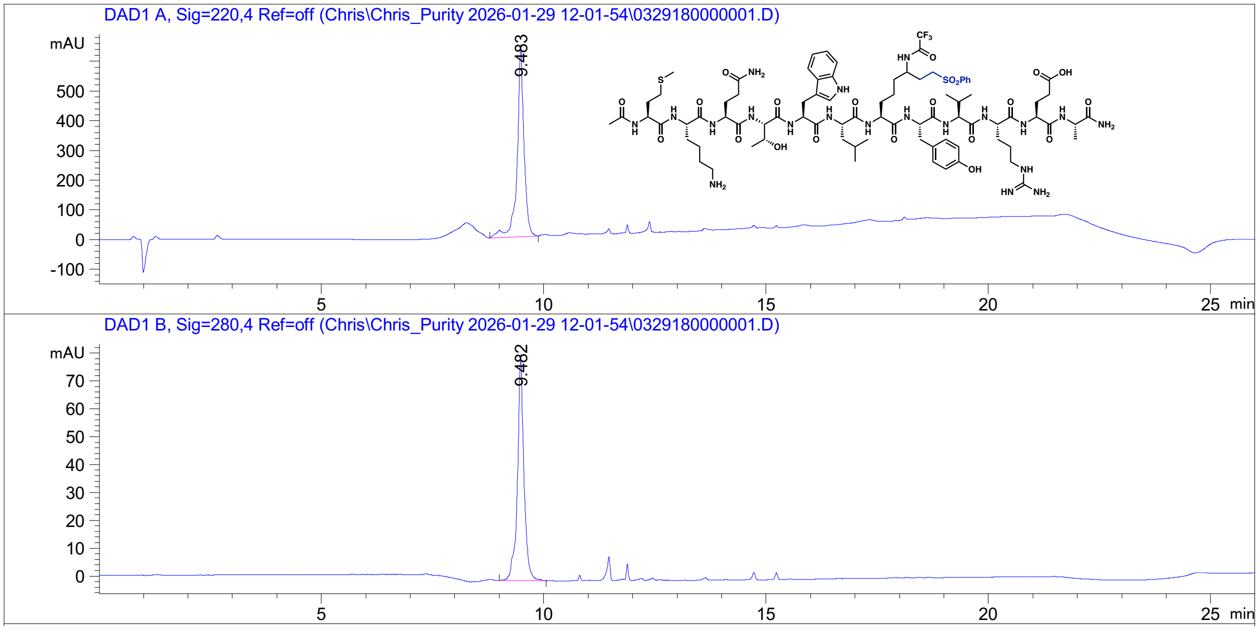

DAD1 A, Sig=220,4 Ref=off (Chris\Chris_Purity 2026-01-29 12-01-54\0329180000001.D)
mAU
9.483
min
DAD1 B, Sig=280,4 Ref=off (Chris\Chris_Purity 2026-01-29 12-01-54\0329180000001.D)
9.482

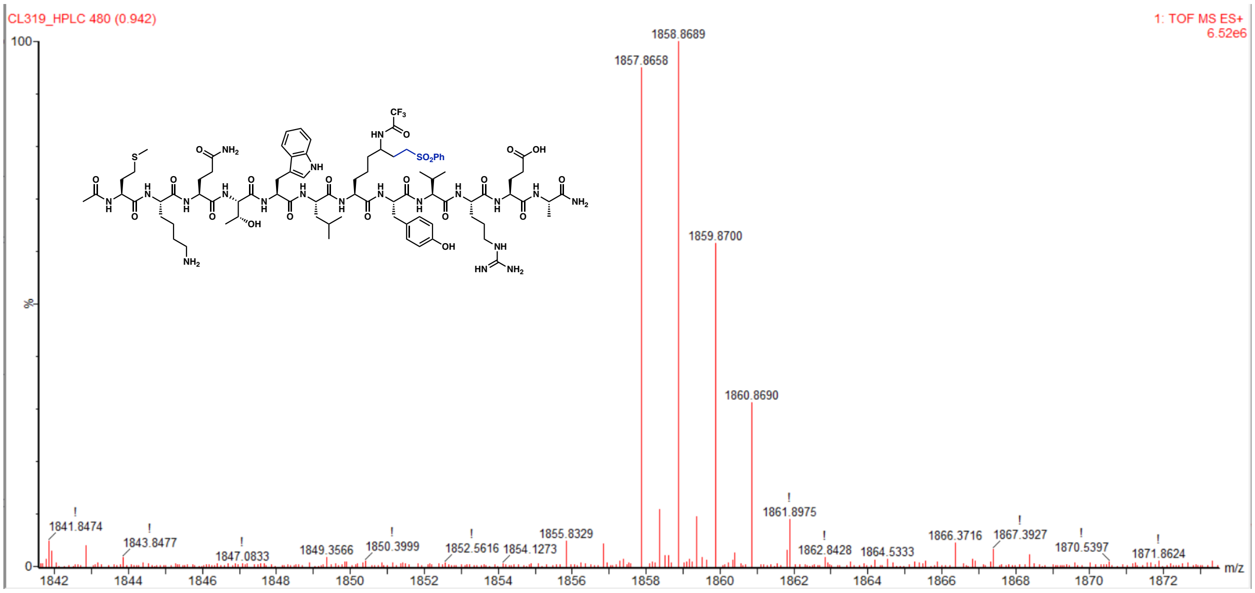

CL319_HPLC 480 (0.942)
1: TOF MS ES+
6.52e6
1858.8689
1857.8658
1859.8700
1860.8690
1861.8975
m/z

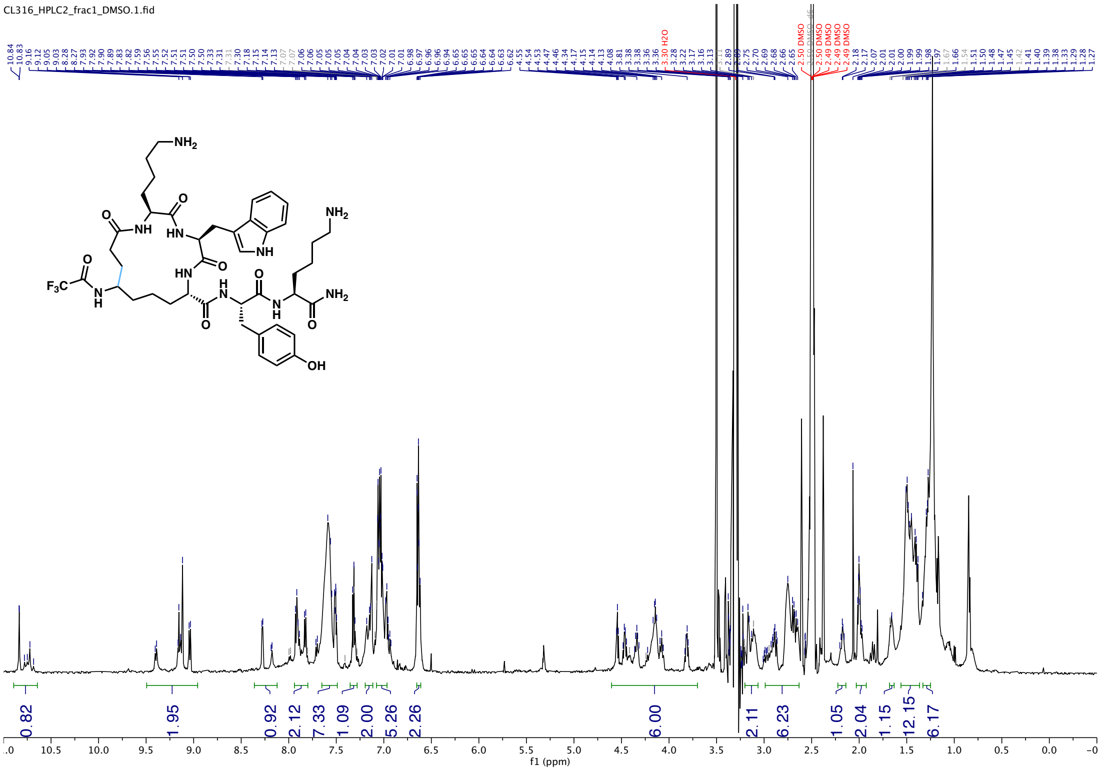
CL316_HPLC2_frac1_DMSO.1.fid
0.82
1.95
0.92
2.12
7.33
1.09
2.00
5.26
2.26
6.00
2.11
6.23
1.05
2.04
1.15
12.15
6.17
f1 (ppm)

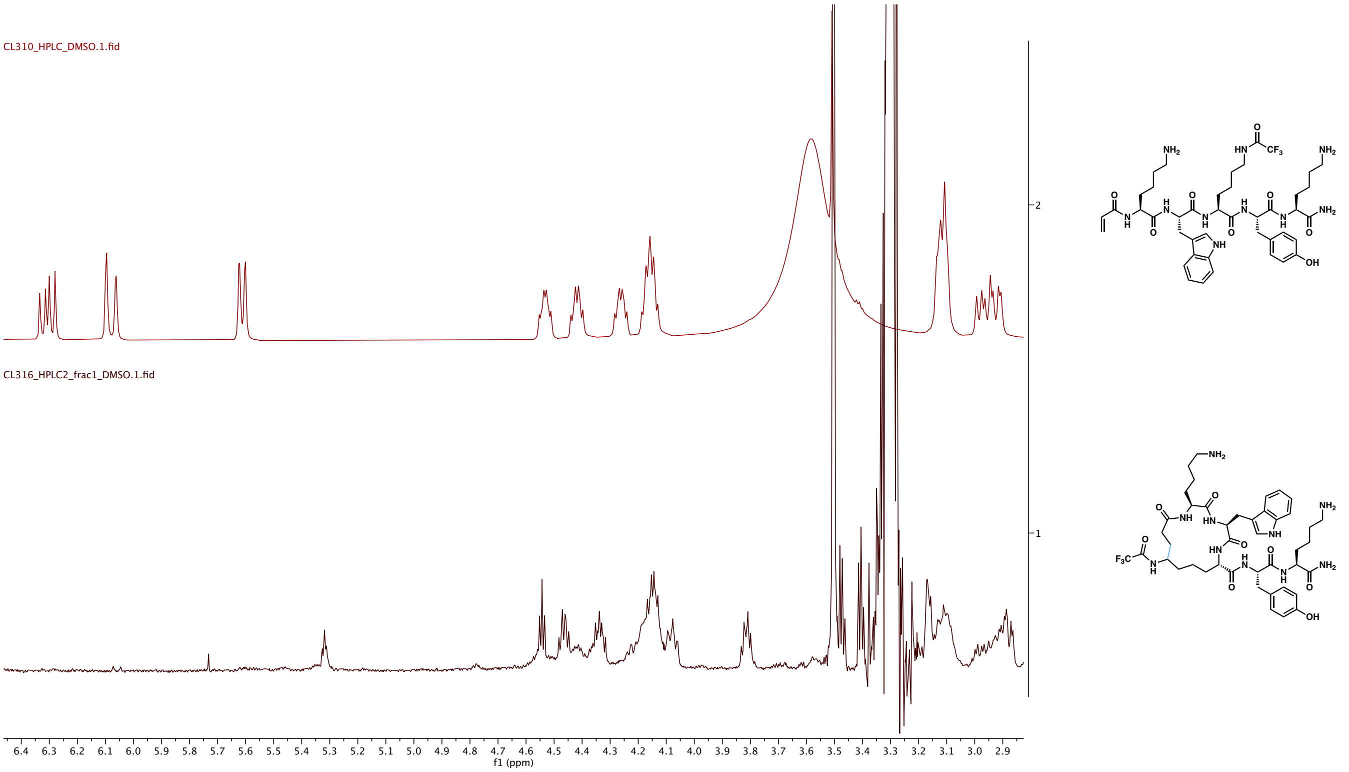
CL310_HPLC_DMSO.1.fid
CL316_HPLC2_frac1_DMSO.1.fid
f1 (ppm)

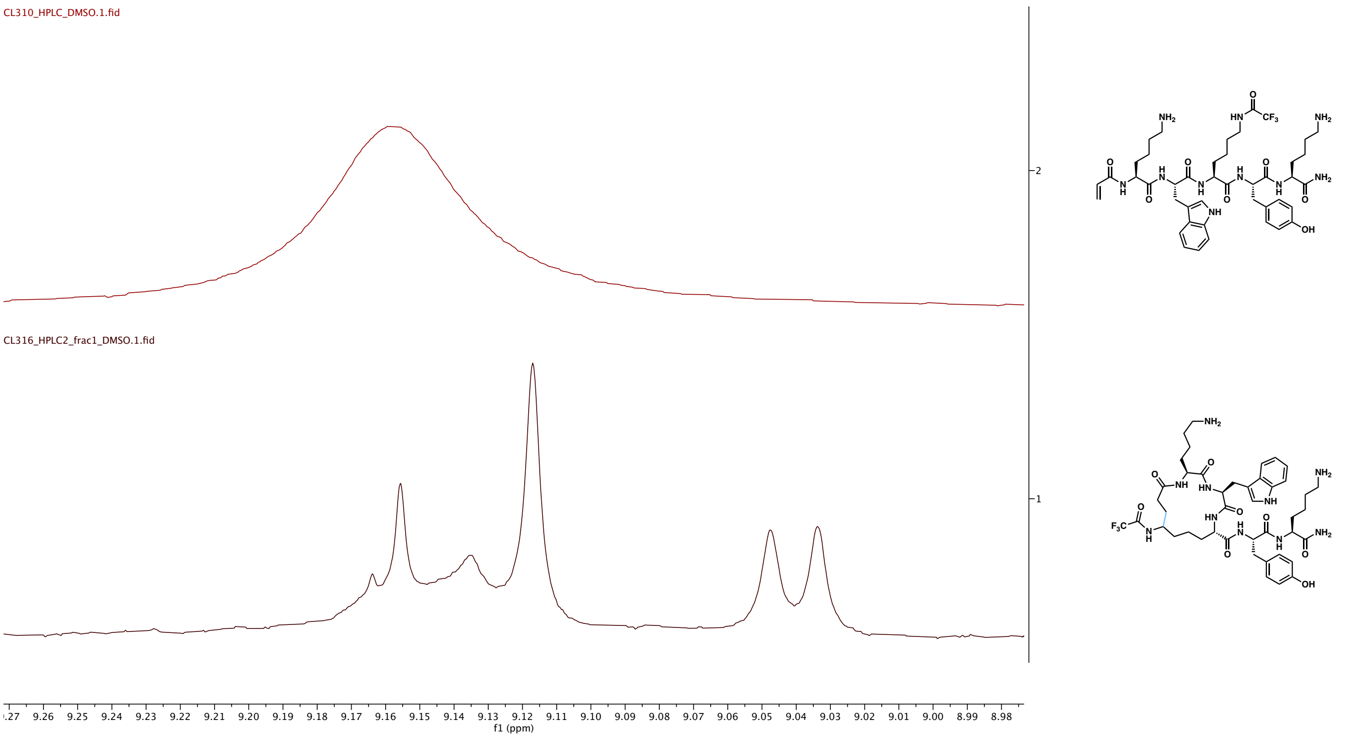
CL310_HPLC_DMSO.1.fid
CL316_HPLC2_frac1_DMSO.1.fid
f1 (ppm)

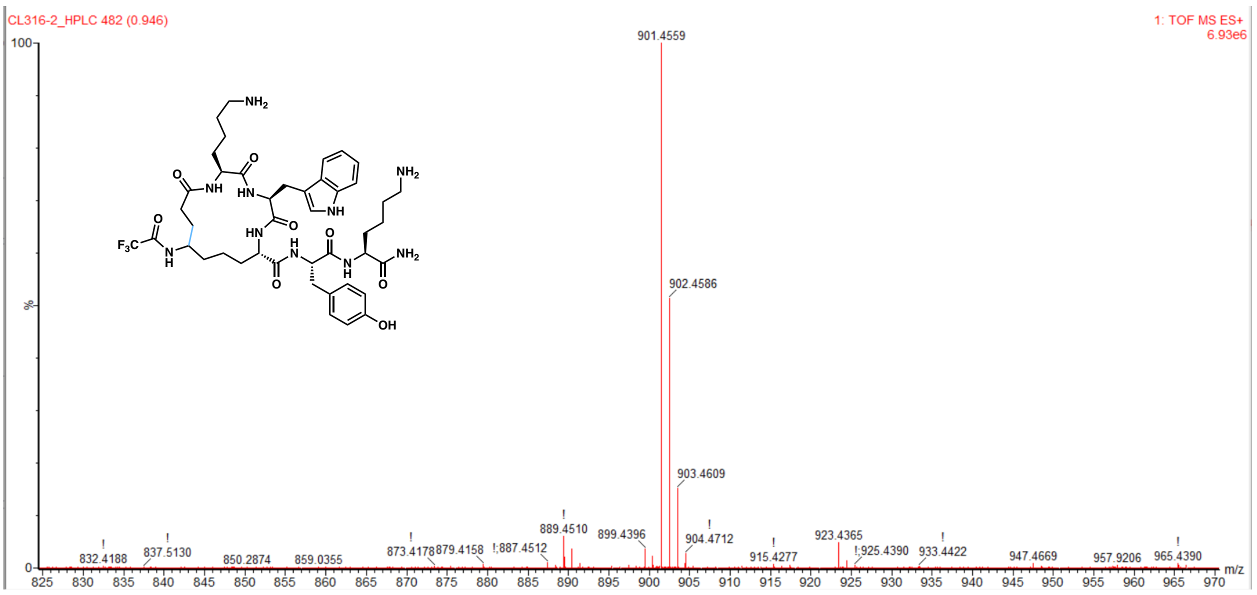
CL316-2_HPLC 482 (0.946)
1: TOF MS ES+
6.93e6
901.4559
902.4586
903.4609
889.4510
899.4396
904.4712
923.4365
m/z

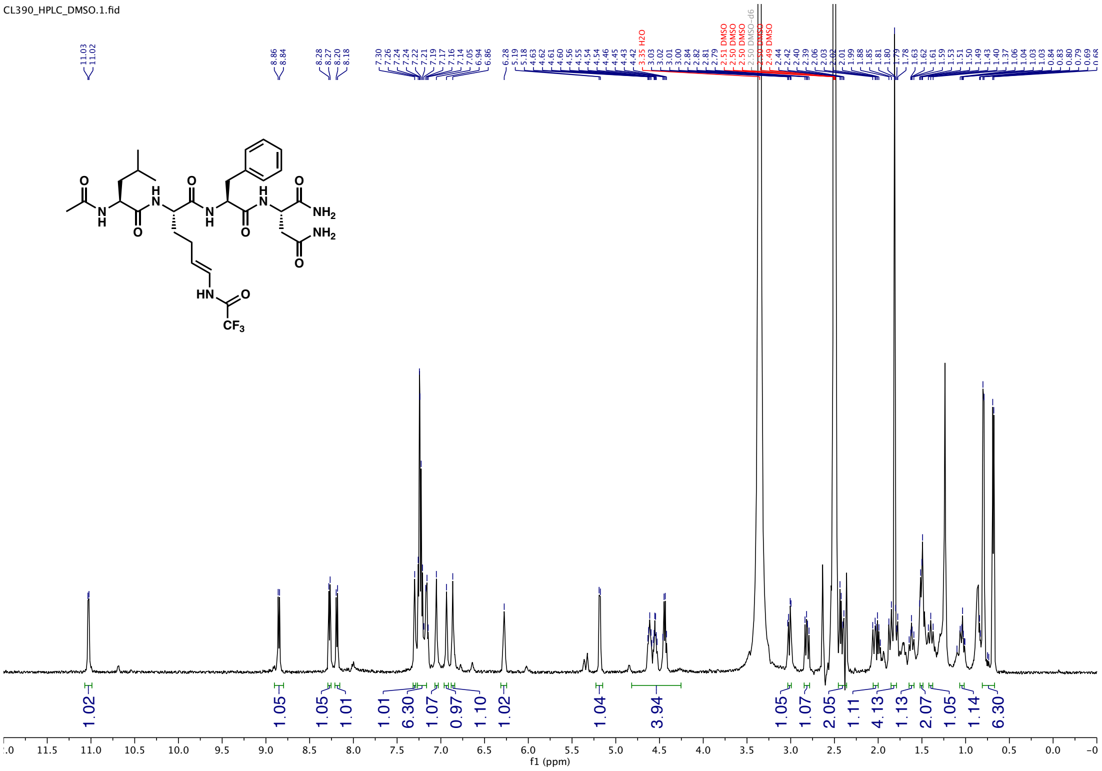
CL390_HPLC_DMSO.1.fid
f1 (ppm)

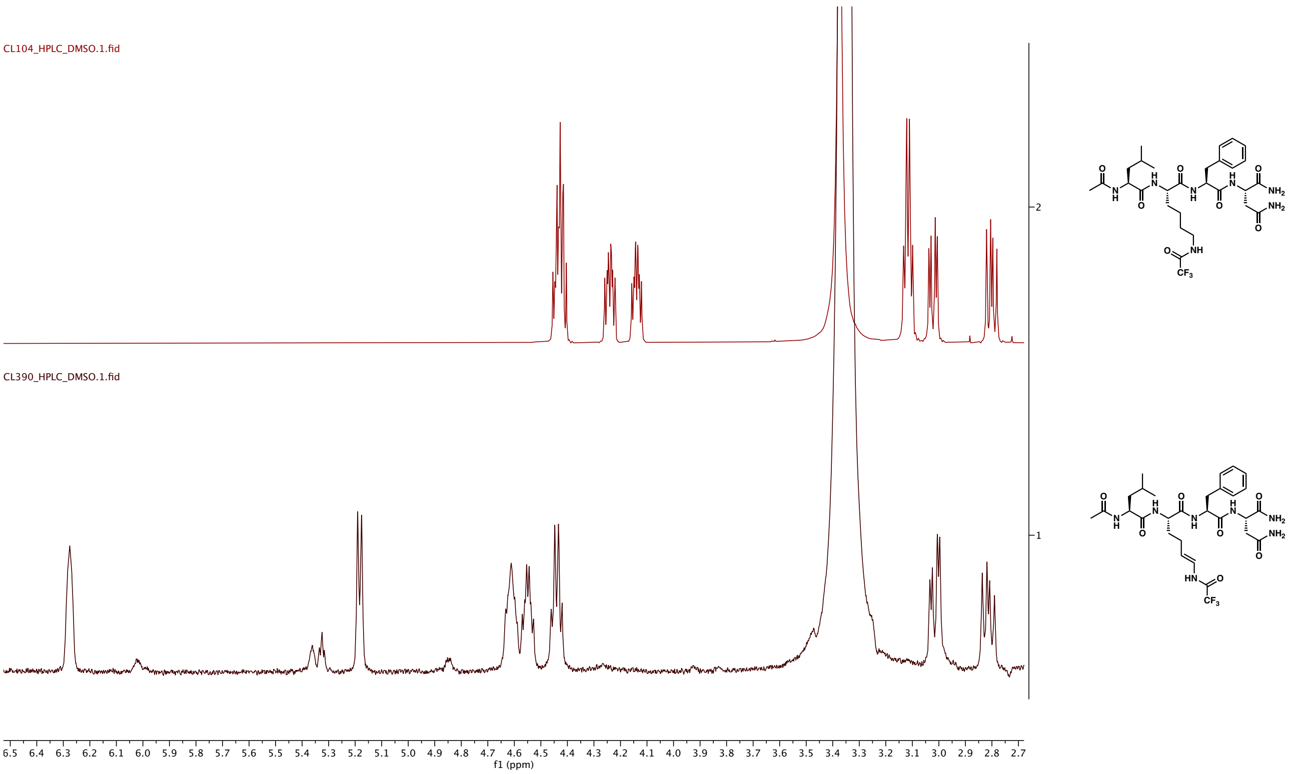
CL104_HPLC_DMSO.1.fid
CL390_HPLC_DMSO.1.fid
f1 (ppm)

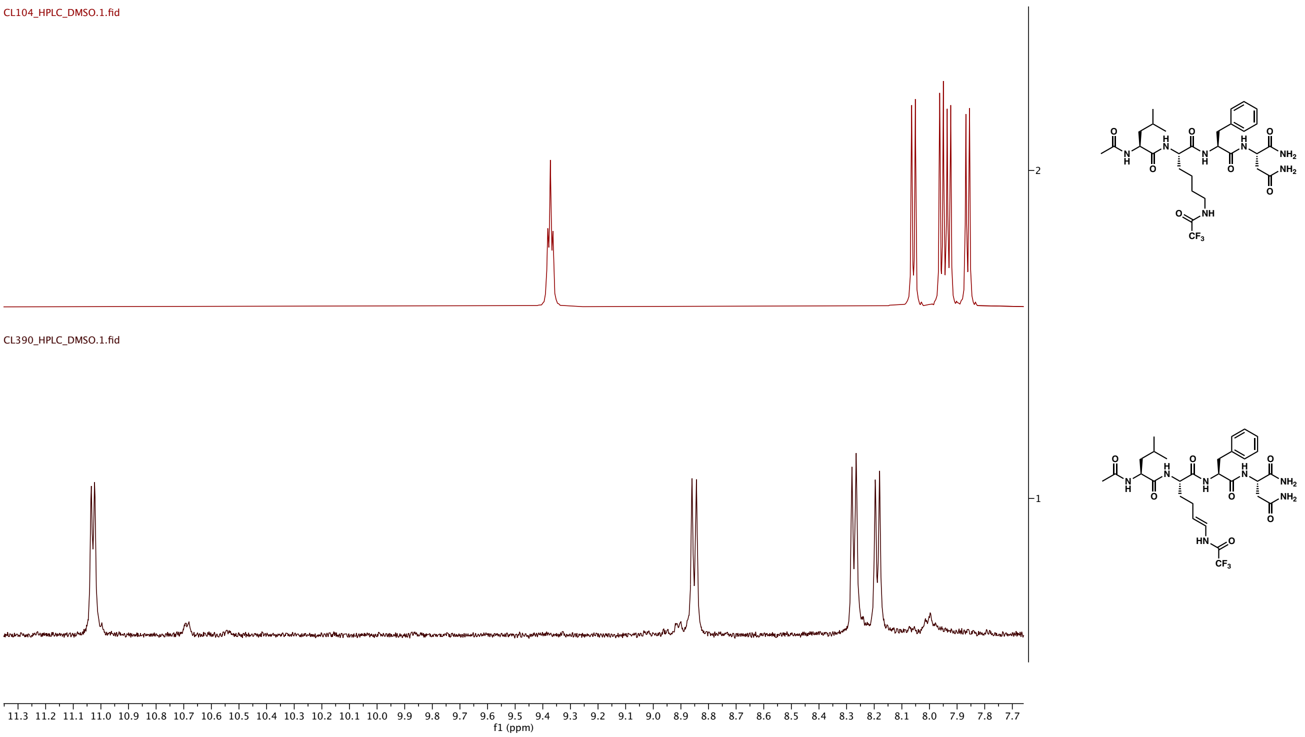

CL104_HPLC_DMSO.1.fid
CL390_HPLC_DMSO.1.fid
f1 (ppm)

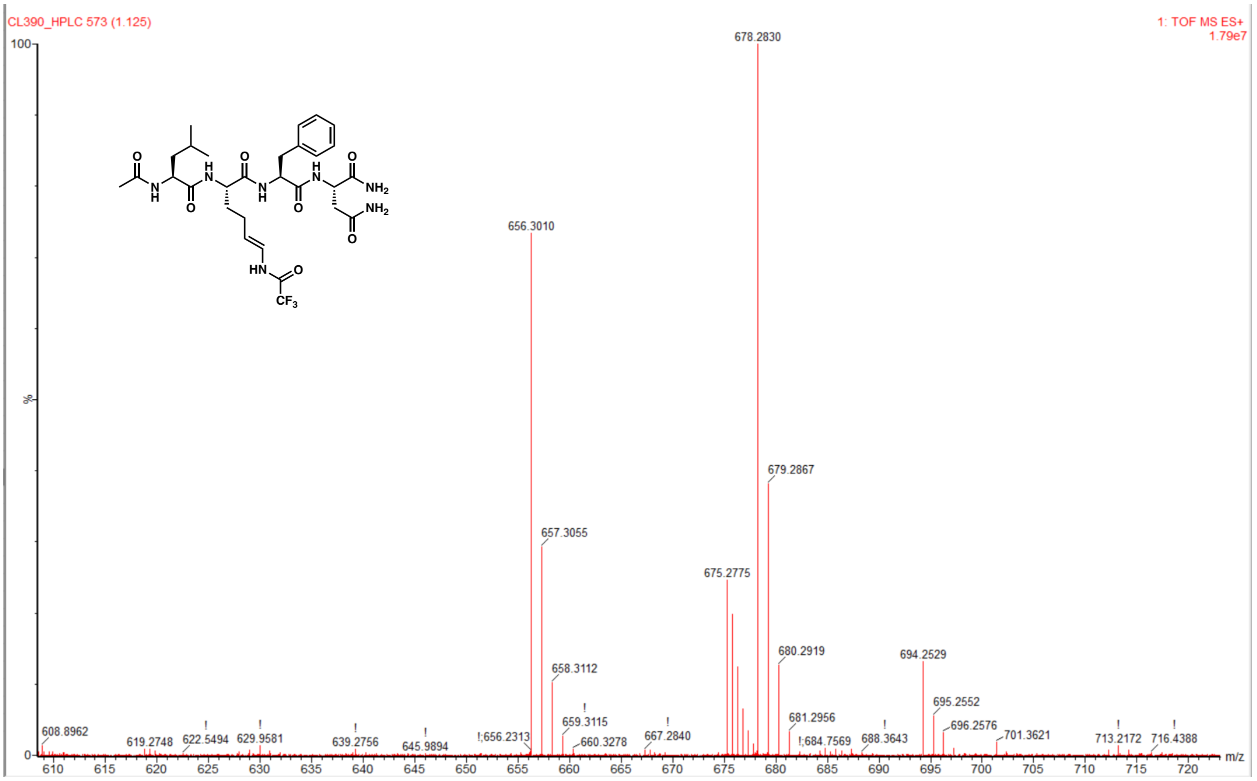

CL390_HPLC 573 (1.125)
1: TOF MS ES+
1.79e7
678.2830
656.3010
679.2867
657.3055
675.2775
680.2919
694.2529
658.3112
695.2552
659.3115
681.2956
696.2576
608.8962
701.3621
713.2172
716.4388
m/z

8. References

(1) Lamartina, C. W.; Chartier, C. A.; Hirano, J. M.; Shah, N. H.; Rovis, T. Crafting Unnatural Peptide Macrocycles via Rh(III)-Catalyzed Carboamidation. *J. Am. Chem. Soc.* **2024**, *146* (30), 20868–20877.

(2) Qi, X.; Jambu, S.; Ji, Y.; Belyk, K. M.; Panigrahi, N. R.; Arora, P. S.; Strotman, N. A.; Diao, T. Late-Stage Modification of Oligopeptides by Nickel-Catalyzed Stereoselective Radical Addition to Dehydroalanine. *Angew. Chem. Int. Ed.* **2022**, *61* (48), e202213315.
